# Diastereoselective ZnCl_2_-Mediated Joullié–Ugi Three-Component Reaction for the Preparation of Phosphorylated *N*-Acylaziridines from 2*H*-Azirines

**DOI:** 10.3390/molecules29051023

**Published:** 2024-02-27

**Authors:** Julene Allende, Iurre Olaizola, Ana M. Ochoa de Retana, Francisco Palacios, Jesús M. de los Santos

**Affiliations:** Department of Organic Chemistry I, Faculty of Pharmacy, Lascaray Research Center, University of the Basque Country (UPV/EHU), Paseo de la Universidad 7, 01006 Vitoria, Spain; julene.allende@ehu.eus (J.A.); yurre.olaizola@ehu.eus (I.O.); anamaria.ochoaderetana@ehu.eus (A.M.O.d.R.); francisco.palacios@ehu.eus (F.P.)

**Keywords:** *N*-acylaziridine, 2*H*-azirine, Joullié–Ugi reaction, oxazol derivatives

## Abstract

We disclose a direct approach to the diastereoselective synthesis of phosphorus substituted *N*-acylaziridines based on a one-pot ZnCl_2_-catalyzed Joullié–Ugi three-component reaction of phosphorylated 2*H*-azirines, carboxylic acids and isocyanides. Hence, this robust protocol offers rapid access to an array of *N*-acylaziridines in moderate-to-good yields and up to 98:2 dr for substrates over a wide scope. The relevance of this synthetic methodology was achieved via a gram-scale reaction and the further derivatization of the nitrogen-containing three-membered heterocycle. The diastereo- and regioselective ring expansion of the obtained *N*-acylaziridines to oxazole derivatives was accomplished in the presence of BF_3_·OEt_2_ as an efficient Lewid acid catalyst.

## 1. Introduction

Multicomponent reactions (MCRs) are highly convergent processes that can be used to create new potent bioactive molecules. On the basis of green chemistry [1], MCRs benefit from efficiency, energy, time and atom economies, use environmentally favorable conditions and decrease the amount of byproducts and/or waste [2,3,4,5,6,7,8]. In addition, MCRs provide one of the most attractive prospects concerning complexity and diversity for the preparation of chemical libraries compared with other traditional synthetic organic methods. A large number of MCRs have been developed and are currently in a promising place in the chemist’s toolbox of sustainable synthetic methodologies.

Among them, the more versatile isocyanide-based MCRs form the backbone of today’s MCRs, such as the Ugi four-component reaction (U-4CR), which comprises the condensation of primary amines, carbonyl compounds, carboxylic acids and isocyanides to furnish dipeptide-like structures [9,10,11,12,13]. As the U-4CR mechanism occurred via the in situ formation of an imine intermediate, the employment of a cyclic imine instead of the amine and the carbonyl components in the Ugi protocol is a simple concept that supplies the robust Joullié–Ugi three-component reaction (JU-3CR). The Ugi variant was first reported by Joullié et al. [14,15] in 1982, and it is an appealing synthetic methodology not only because nitrogen-containing heterocycles can be directly attained in a single step, but also because of its greater stereochemical control [16]. The ring strain and the impossibility of imine *E*/*Z* isomerization contribute to a greater diastereoselectivity. Likewise, Joullié–Ugi adducts are privileged structures of interest in medicinal chemistry due to the synergistic effect of peptidic moieties linked to the nitrogen heterocycles leading to products with unique pharmaceutical activities [17,18,19,20].

**Figure 1:**
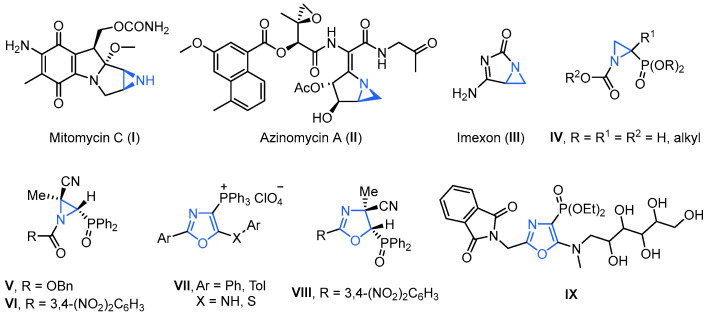
Aziridine-containing natural products and selected examples of phosphorus-substituted oxazole derivatives with high potential for medical applications.

Due to a better understanding of the benefits of covalent binding mechanisms and to the FDA support of effective and innocuous covalent drugs, there is great interest in covalent binding therapeutics [21,22,23]. Targeted covalent inhibitors are designed by incorporating an electrophile into a ligand that would bind the target protein. The integrated electrophile, acting as “warheads” [24], including ketone, α,β-unsaturated carbonyl, nitrile, ester, epoxide or aziridine, binds irreversibly to endogenous nucleophilic functionalities, including lysine, tyrosine, serine, cysteine and threonine, among others, on the target protein, introducing a covalent interaction. In this regard, aziridines, well known for their robust alkylating properties, possess the potential to function as potent covalent drugs by virtue of their ability to serve as DNA cross-linking agents. This is achieved through the nucleophilic ring opening of the three-membered nitrogen-containing heterocycle [25]. Aziridines are an important class of synthetic targets because they often exhibit a broad range of biological activities; for example, aziridine-containing mitomycin C (**I**) [26], azinomycin A (**II**) [27] and imexon (**III**) [28] show antitumor activity (Figure 1). Other natural aziridines, also known as aziridine alkaloids, display antibacterial and antimicrobial activity against selected microorganisms. For instance, aziridine-2-phosphonates **IV** have been claimed to show antibacterial properties [29] (Figure 1). We have recently reported the synthesis of phosphorus-substituted *N*-acylaziridines **V** [30] and **VI** [31], which exhibited a very good cytotoxic effect inhibiting the growth of human tumor cell line A549 (adenocarcinomic human alveolar basal epithelial cells). Recently, oxazoles have been emerged as the critical pharmacophore for various biological and medicinal applications. They serve as the key structural motif in numerous naturally occurring compounds and exhibit a broad spectrum of pharmacological properties, such as anti-cancer, anti-tubercular, anti-bacterial, anti-fungal, anti-parasitic and anti-viral properties, among others [32]. Hence, they can be utilized as primary building blocks in the pharmaceutical sector for the synthesis of several drugs. Likewise, from a biological perspective, organophosphorus compounds are very interesting due to their ability to modify the reactivity of heterocycles and regulate essential biological functions [33]. Thus, the anticancer agents 1,3-oxazol-4-ylphosphonium perchlorates **VII** [34], or the oxazole-phosphine oxide derivative **VIII** [31], and the oxazol-4-yl-phosphonate derivative **IX** displaying interesting anti-human cytomegalovirus (HCMV) properties [35], are only some examples of phosphorus-substituted oxazole derivatives with high potential for medical applications.

**Scheme 1:**
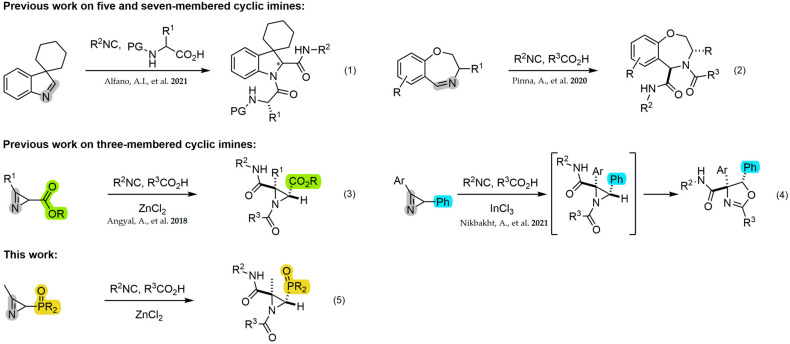
JU-3CR on three, five and seven-membered cyclic imines [39,42,43,44].

Many of the reported JU-3CR examples in the literature primarily employ 5, 6 or 7-membered cyclic imines for the preparation of pyrrolidine [36,37], thiazolidine [38], indolines [39,40] (Scheme 1, Equation (1)), piperidine [36,41] or oxazepine [42] peptidomimetics (Scheme 1, Equation (2)). However, only two examples have been reported in relation to the use of 2*H*-azirines as cyclic imines in the three-component Joullié–Ugi reaction for the synthesis of *N*-acylaziridines. Kanizsai et al. [43] first described the Lewis acid-promoted version of the Joullié–Ugi reaction using 2*H*-azirine-2-carboxylates for the diastereoselective synthesis of *N*-acylaziridines-2-carboxamide derivatives (Scheme 1, Equation (3)). Furthermore, a domino JU-3CR/diastereoselective ring expansion reaction was applied in the preparation of oxazolines from 2-phenyl-2*H*-azirines [44] (Scheme 1, Equation (4)).

**Figure 2:**
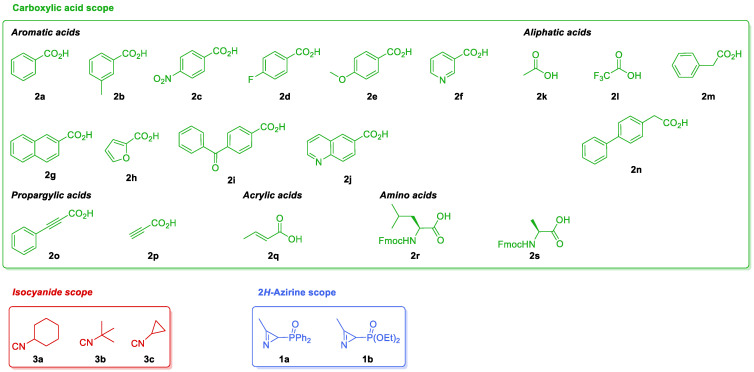
The scope of phosphorus-substituted 2*H*-azirines **1**, carboxylic acids **2** and isocyanides **3** tested in the JU-3CR.

We have been previously involved in the chemistry of phosphorus-substituted 2*H*-azirines for the synthesis of phosphorylated cyanoaziridines [30] and their ring expansion [31], hybrid molecules such as azirino[2,1-*b*]benzo[*e*][1,3]oxazines [45] or α-aminophosphonic acid derivatives [46]. In continuation of our previous research works, as depicted in (Scheme 1, Equation (5)), here, we describe a diastereoselective approach to phosphorylated *N*-acylaziridine 2-carboxamide derivatives through the JU-3CR using phosphorus-substituted 2*H*-azirines such as 3-membered cyclic imines, carboxylic acids and isocyanides (Figure 2).

## 2. Results

As outlined in Table 1, we started our investigation with the optimization of the three-component reaction conditions of 2*H*-azirine phosphine oxide **1a**, benzoic acid (**2a**) and cyclohexyl isocyanide (**3a**) in THF at 60 °C. The Joullié–Ugi reaction without a catalyst led to the obtention of only a 10% yield of *N*-acylaziridine phosphine oxide **4a** (entry 1). It is well known that the activation of 2*H*-azirines by Lewis acids may significantly enhance their reactivity [47,48]. Thus, we next explored the Lewis or Brønsted acid-mediated JU-3CR. Only a 14% yield of **4a** could be achieved when PTSA was used as the Brønsted acid catalyst in this process (entry 2). Trifluoromethanesulfonic acid (TfOH, entry 3) showed moderate catalytic activity since the reaction proceeded smoothly in THF at 60 °C to give the product **4a** in a 35% yield. Increasing the amount of TfOH from 10 mol% to 25 mol% did not improve the yield of compound **4a**. The JU-3CR was carried out in the presence of different Lewis acids. For instance, Ti(O*^i^*Pr)_4_, Sc(OTf)_3_, InCl_3_, BF_3_·OEt_2_, MgBr_2_ and ZnCl_2_·H_2_O were not suitable for the current reaction since compound **4a** could not be detected, and only the starting 2*H*-azirine **1a** or decomposition products were recovered instead (entries 4–9). In general, the most active catalyst for the JU-3CR of **1a** with carboxylic acid **2a** and isocyanide **3a** was found to be ZnCl_2_ (Table 1, entry 10), which is consistent with literature reports of other JU-3CRs involving 2*H*-azirine-2-carboxylates [43] Therefore, the use of ZnCl_2_ (25 mol%, entry 10) resulted in the formation of *N*-acylaziridine **4a** in a 60% chemical yield, together with a small amount of the pyrazine that proceed from the thermal treatment of the corresponding 2*H*-azirine phosphine oxide **1a** [49]. This process yielded product **4a** in a diastereomeric ratio of 96:4.

The effect of the solvent on the JU-3CR was also tested. The ZnCl_2_ catalyst in this process was incompatible with some solvents such as MeOH or MeCN (entries 11 and 12), and in both cases, no reaction product was observed. The degree of consumption of the starting 2*H*-azirine **1a** was found to depend on reaction temperature. In the reaction conducted at –10 °C (entry 13), a significant amount of unreacted 2*H*-azirine **1a** was recovered and the expected product **4a** was obtained in a low yield. The JU-3CR was found to work better when the reaction was carried out at room temperature, and the almost complete conversion of the 2*H*-azirine **1a** was then observed (entry 14). Finally, different amounts of ZnCl_2_ were examined, which showed that decreasing the amount of ZnCl_2_ (10 mol%, entry 15) led to the desired product **4a** in a low yield together with α-ketamide derived from the nucleophilic addition of the carboxylic acid to 2*H*-azirine **1a** [50]. Increasing the amount of ZnCl_2_ up to 30 mol% (entry 16) did not affect the yield of compound **4a**.

**Scheme 2:**
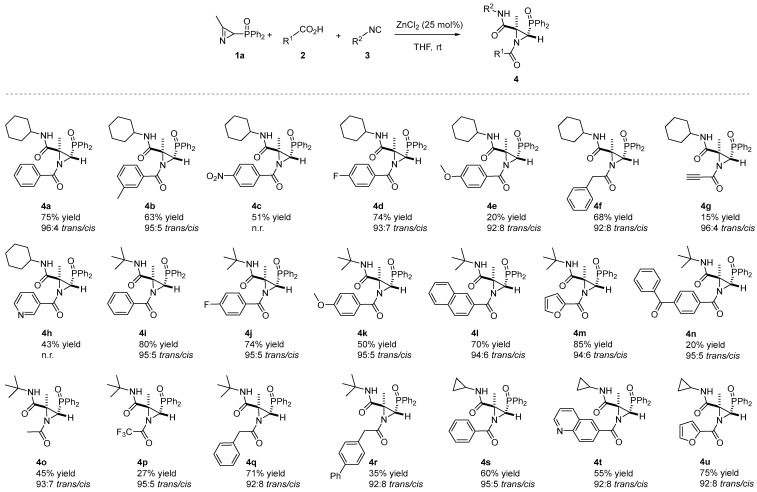
Substrate scope of the *N*-acylaziridine phosphine oxides **4** in the Joullié–Ugi three-component reaction. See the Supporting Information for experimental details. Reactions were carried out on a 0.5 mmol scale; 2*H*-azirine **1a** (0.5 mmol), carboxylic acid **2** (1.3 eq.), isocyanide **3** (1.3 eq.), ZnCl_2_ (25 mol%) and solvent (0.5 mL). Diastereomeric ratio (*trans*/*cis*) determined from the crude reaction mixture via ^1^H NMR. It was not possible to determine the diastereomeric ratio of compounds **4c** and **4h** as the crude reaction NMR spectra were not clean enough.

Given that the results of this preliminary investigation seemed to define ZnCl_2_ as the best catalyst in the JU-3CR in THF and room temperature as the best reaction condition, we adopted these conditions for further studies. Then, a range of *N*-acylaziridine phosphine oxides **4** with diverse substitution patterns (Figure 2) on the aziridine ring were prepared. A considerable selection of aromatic carboxylic acid partners **2** were well tolerated in the JU-3CR with **1a** and **3** as coupling partners (Scheme 2). Both electron-donating (OMe) and electron-withdrawing groups (F, NO_2_) at the *para*-phenyl position of aromatic carboxylic acid **2** yielded desired products **4c**, **4d**, **4e**, **4j** and **4k** in 20–74% yields and very good diastereoselectivities. Among them, 4-fluor derivatives **4d** and **4j** were achieved with the best yields (74%).

The electron-donating group (Me) at the *meta*-phenyl position of aromatic carboxylic acid also furnished *N*-acylaziridine **4b** in a 63% yield (Scheme 2). Other aromatic carboxylic acids such as 2-naphthoic acid **2g** or 4-benzoylbenzoic acid **2i** were selected as suitable candidates for this transformation, providing products **4** in moderate-to-good yields. Even heteroaromatic carboxylic acids were well tolerated in the Joullié–Ugi three-component reaction. For instance, nicotinic acid **2f**, 2-furoic acid **2h** and or quinoline-6-carboxylic acid **2j** gave desired products **4h**, **4m**, **4t** and **4u** in 43–85% yields. Phenylacetic acid **2m** yielded *N*-acylaziridines **4f** and **4q** in a 68 and 71% yield, respectively. However, worse yields of *N*-acylaziridines **4o**, **4p** and **4r**, derived, respectively, from acetic acid **2k**, trifluoroacetic acid **2l** and 4-biphenylacetic acid **2n** were attained (Scheme 2). Moreover, propargylic acids, such as propiolic acid **2p**, can also be subjected to the JU-3CR to give compound **4g** in a low yield but with high diastereoselectivity (96:4 *trans*:*cis* dr). This confirms the strength of the carboxylic acid scope in the JU-3CR.

In order to assess the applicability of the JU-3CR, we next investigated the scope of this process with regard to the isocyanide partner. Besides the cyclohexyl isocyanide (**3a**), other aliphatic isocyanides such as *tert*-butyl isocyanide (**3b**) or cyclopropyl isocyanide (**3c**) have been tested in the JU-3CR. All the isocyanides studied are well tolerated, giving the *N*-azylaziridines **4** in a moderate-to-good yield (see Scheme 2). The isocyanide partner does not affect the outcome of this protocol. For instance, compare the chemical yields of **4a**, **4i** and **4s** (60–80% yields), **4d** and **4j** (74% yield) or **4m** and **4u**, which proceeded smoothly in 85 and 75% yields (Scheme 2).

A careful examination of the spectroscopic data of the crude reaction mixture of *N*-acylaziridine **4a**, showed two well-resolved doublets in the ^1^H NMR spectrum corresponding to the H3 methine proton of the aziridine ring, which is consistent with the presence of two diastereoisomers. The major diastereoisomer appears at *δ*_H_ ~ 3.81 ppm with the coupling constant ^2^*J*_PH_ = 24.0 Hz, and the minor one at *δ*_H_ ~ 3.35 ppm with a lower coupling constant of ^2^*J*_PH_ = 21.7 Hz in a ratio of 96:4. Substrates **4** were extensively characterized on the basis of their ^1^H, ^13^C, ^31^P, ^19^F NMR and 2D NMR spectra and HRMS (see the Supporting Information). The most characteristic signals for *N*-acylaziridine **4a** (major diastereoisomer) in the ^1^H NMR spectrum are the two well-resolved doublets at *δ*_H_ ~ 5.76 ppm (^3^*J*_HH_ = 8.1 Hz) and *δ*_H_ ~ 3.81 ppm with a coupling constant of ^2^*J*_PH_ = 24.0 Hz, corresponding to the NH of amide group and the H3 methine proton of the aziridine ring, respectively. A singlet at *δ*_H_ ~ 1.96 ppm was attributed to the methyl group. In the ^13^C NMR spectrum, the formation of compound **4a** is evident from the presence of two carbonyl groups at *δ*_C_ ~ 176.2 (^3^*J*_PC_ = 3.3 Hz) and 165.6 ppm, while the quaternary carbon C2 appears as a doublet at *δ*_C_ ~ 49.6 ppm (^2^*J*_PC_ = 3.0 Hz) and the methine carbon C3 shows a chemical shift at *δ*_C_ ~ 42.0 ppm with a large coupling constant (^1^*J*_PC_ = 100.4 Hz).

**Figure 3:**
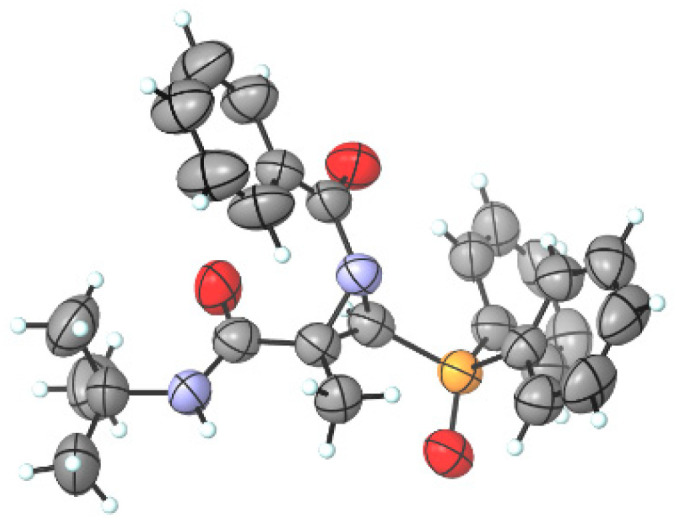
ORTEP diagram of functionalized *N*-acylaziridine phosphine oxide **4i** (H, white; C, grey; O, red; N, blue; P, orange) (2*S*,3*S* enantiomer shown).

Since it was not possible to assign the stereochemistry of *N*-acylaziridines **4** via ^1^H and ^13^C NMR, their structure has been unambiguously determined via X-ray diffraction analysis [51,52,53], establishing the *trans*-relationship between the amide group at the C2 position and the phosphorus moiety at the C3 position of the major diastereoisomer **4i**. The CIF data are presented in the Supporting Information, and the ORTEP drawing of **4i** is shown in Figure 3.

**Scheme 3:**
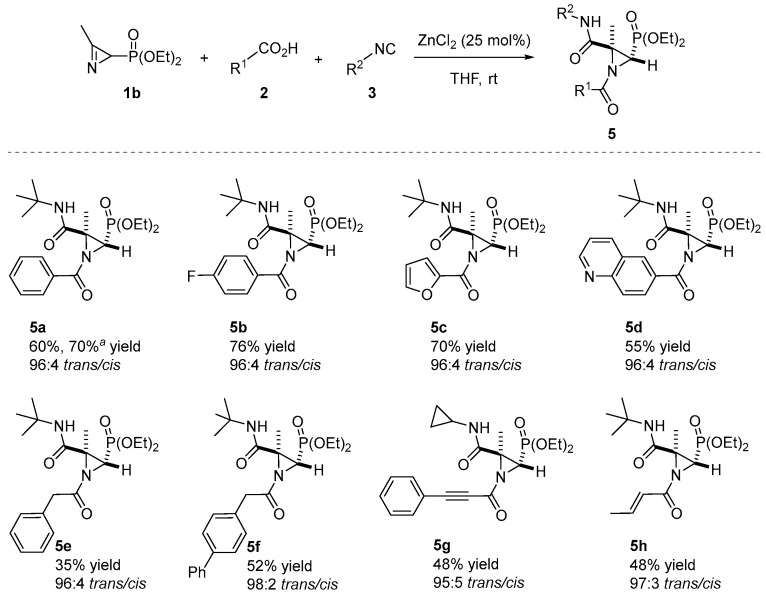
Substrate scope of the *N*-acylaziridine phosphonates **5** in the Joullié–Ugi three-component reaction. See the Supporting Information for experimental details. Reactions were carried out on a 0.5 mmol scale; 2*H*-azirine **1b** (0.5 mmol), carboxylic acid **2** (1.3 eq.), isocyanide **3** (1.3 eq.), ZnCl_2_ (25 mol%) and solvent (0.5 mL). *^a^* Yield of isolated compound **5a** after crystallization, at a 4 mmol scale. The diastereomeric ratio (*trans*/*cis*) was determined from the crude reaction mixture via ^1^H NMR.

The ZnCl_2_-catalyzed Joullié–Ugi three-component reaction was extended to the use of 2*H*-azirine phosphonate **1b**. Then, 2*H*-azirine **1b** reacted with a series of carboxylic acids **2** and isocyanides **3** in THF at room temperature in the presence of ZnCl_2_ (25 mol%). As outlined in Scheme 3, aromatic (**2a** and **2d**), heteroaromatic (**2h** and **2j**), aliphatic (**2m** and **2n**), propargylic (**2o**) and acrylic (**2q**) acids are allowable in the JU-3CR, yielding the desired *N*-acylaziridine phosphonates **5** in chemical yields ranging from 35 to 76%.

**Scheme 4:**
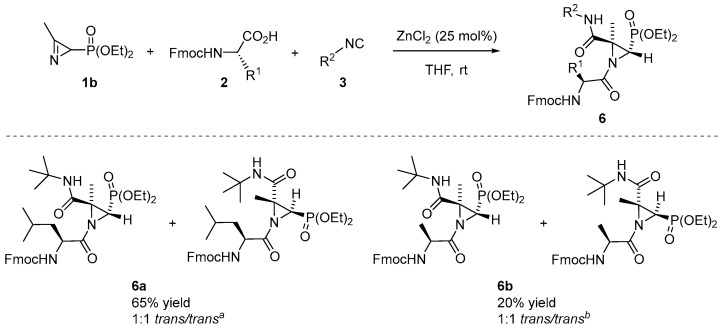
Substrate scope of phosphorylated aziridine peptidomimetics **6** through the Joullié–Ugi three-component reaction using *N*-Fmoc amino acids. See the Supporting Information for experimental details. Reactions were carried out on a 1 mmol scale; 2*H*-azirine **1b** (1 mmol), Fmoc-protected amino acid **2r** or **2s** (1.3 eq.), isocyanide **3b** (1.3 eq.), ZnCl_2_ (25 mol%) and solvent (1 mL). *^a^* Diastereomeric ratio (*trans*/*trans*) determined from the crude reaction mixture via ^1^H NMR. The diastereomeric ratio between *trans*- and *cis*-diastereoisomers is approximately 92:8. *^b^* The diastereomeric ratio (*trans*/*trans*) determined from the crude reaction mixture via ^1^H NMR. The diastereomeric ratio between *trans* and *cis*-diastereoisomers could not be determined.

Encouraged by the abovementioned obtained results of the Joullié–Ugi three-component reaction between phosphorylated 2*H*-azirines **1**, carboxylic acids **2** and isocyanides **3**, we further investigated the substrate scope using *N*-Fmoc-protected amino acids as carboxylic acid partners for the preparation of phosphorylated aziridine peptidomimetics. To our delight, it was found that the reaction proceeded smoothly when 2*H*-azirine phosphonate **1b** reacted with *tert*-butyl isocyanide (**3b**) and Fmoc-Leu (**2r**, R^1^ = *^i^*Bu) in the standard conditions to yield derivative **6a** in a 65% isolated yield (Scheme 4). The ^1^H NMR spectrum of the crude reaction mixture confirmed the presence of two well-resolved doublets at *δ*_H_ = 3.03 and 2.90 ppm with a coupling constant of ^2^*J*_PH_ = 18.6 and 18.4 Hz, respectively, for the H3 methine proton of the aziridine ring of both *trans*-diastereoisomers, in a ratio of 1:1: Conversely, another doublet appeared at *δ*_H_ = 2.71 ppm with a lower coupling constant of ^2^*J*_PH_ = 14.7 Hz corresponding to H3 of the *cis*-diastereoisomer, while the fourth doublet corresponding to the other *cis*-diastereoisomer appeared overlapped in the range of *δ*_H_ = 2.83–2.75 ppm. The diastereomeric ratio between *trans-* and *cis*-diastereoisomers is approximately 92:8. After purification via flash-chromatography, it was possible to identity both *trans*-diastereoisomers of **6a**. The Joullié–Ugi reaction between 2*H*-azirine **1b**, *tert*-butyl isocyanide (**3b**) and Fmoc-Ala (**2s**, R^1^ = Me) using 25 mol% of ZnCl_2_ in THF and at room temperature led to the formation of derivative **6b** in a lower yield (Scheme 4). Via the ^1^H NMR of the crude compound, it was possible to determine the 1:1 ratio between both *trans*-diastereoisomers. Nevertheless, in this case, determining the diastereomeric ratio (*trans*/*cis*) was infeasible.

The gram-scale synthesis of phosphorylated *N*-acylaziridines was accomplished, as shown in Scheme 3. The use of 4.0 mmol of 2*H*-azirine phosphonate **1b**, under the JU standard conditions, gave *N*-acylaziridine phosphonate **5a** in a 70% yield (1.11 g) after recrystallization.

**Scheme 5:**
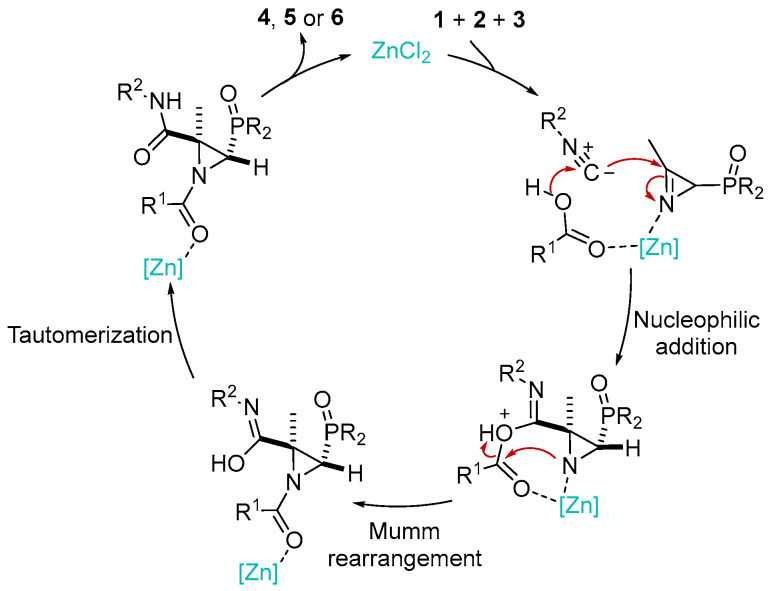
Plausible mechanism for the synthesis of phosphorus substituted *N*-Acylaziridines **4**, **5** and **6** through the Joullié–Ugi three-component reaction.

Scheme 5 outlines a plausible mechanism for the JU-3CR. This process carried out in a polar solvent, suggesting the formation of polar intermediates, is compatible with a stepwise mechanism. The addition of Lewis acids (in our case ZnCl_2_) increases the electrophilicity of the iminic C–N double bond in 2*H*-azirine **1**. Thus, the electrophilic imine and nucleophilic carboxylic acid **2** add to the carbon atom of isocyanide **3**. The amino group of the adduct thus formed promotes the irreversible Mumm rearrangement in the presence of a zinc catalyst and the intramolecular acylation of the amine nitrogen atom, which after subsequent hydroxylimine → amide tautomerization leads to phosphorylated *N*-acylaziridines **4**, **5** or **6**.

**Scheme 6:**
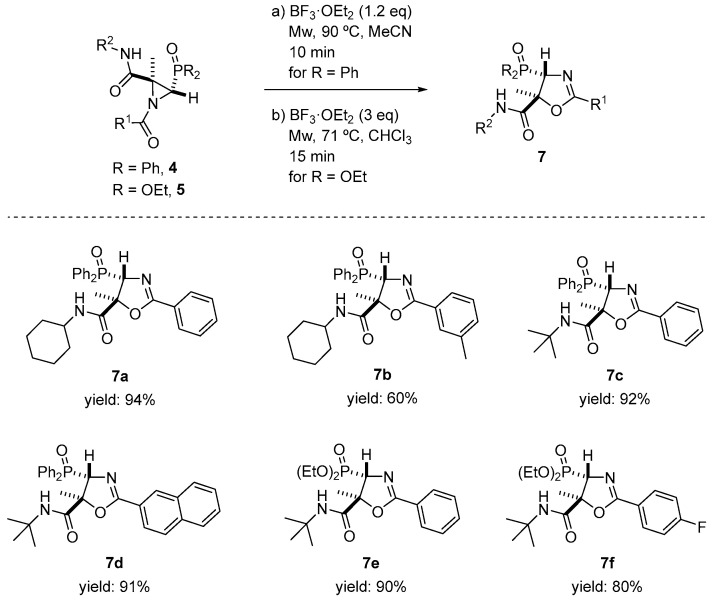
Phosphorus-substituted oxazole derivatives **7** through the ring expansion of *N*-acylaziridines **4** or **5**. See the Supporting Information for experimental details. Reactions were carried out on a 0.12–0.80 mmol scale; *N*-acylaziridine **4** or **5** (0.12–0.80 mmol), BF_3_·OEt_2_ (1.2 eq. for *N*-acylaziridines **4** derived from phosphine oxide and 3 eq. for *N*-acylaziridines **5** derived from phosphonate) and solvent (20 mL/mmol).

We performed further derivatization in order to illustrate the utility of the Joullié–Ugi adducts. Thus, we explored the isomerization reaction of *N*-acylaziridines **4** and **5** to oxazole derivatives. For this purpose, and taking into account that the regio- and stereochemical outcomes of these rearrangements strongly depend on the reaction conditions, as well as the substitution pattern of the *N*-acylaziridine, we started exploring thermal conditions for the ring opening of compounds **4** and **5**. Thus, phosphorus-substituted *N*-acylaziridine **4a** was heated in refluxing CHCl_3_. Under these conditions, the corresponding oxazole derivative was not observed, and the unreacted starting substrate was recovered instead. Next, the rearrangement of **4a** was also tested under nucleophilic conditions [31,54,55,56,57]. When **4a** reacted with 0.2 equivalents of NaI in THF at 60 °C, as in the previous case, no satisfactory results were attained, observing only decomposition products. Likewise, the isomerization of the *N*-acylaziridine to oxazole derivative under mild acidic conditions was examined. Compound **4a** was treated with both Brønsted acids, including *p*-toluenesulfonic acid (PTSA), and Lewis acids, including BF_3_·OEt_2_. Only the use of BF_3_·OEt_2_ gave satisfactory results. Hence, when *N*-acylaziridine **4a** reacted in the presence of 1.2 equivalents of BF_3_·OEt_2_ in MeCN at 90 °C and under microwave irradiation for 10 min, the formation of 4-diphenylphosphoryl-4,5-dihydrooxazole-5-carboxamide **7a** was achieved in a very good yield and in a regio- and diastereoselective fashion (Scheme 6).

Spectroscopic data confirmed the isomerization of *N*-acylaziridine **4a** into oxazole derivative **7a**. While the ^1^H NMR spectrum of **4a** shows a signal for the methyl group at *δ*_H_ = 1.96 ppm and the methine hydrogen resonates at *δ*_H_ = 3.81 ppm as a well-resolved doublet (^2^*J*_PH_ = 24 Hz, see above), in dihydrooxazole-5-carboxamide **7a**, these signals appear at *δ*_H_ = 1.63 and 5.32 ppm as a singlet and a well-resolved doublet with a much lower coupling constant (^2^*J*_PH_ = 6.0 Hz), respectively. Similarly, other *N*-acylaziridines derived from phosphine oxide **4b**, **4i** and **4l** reacted with BF_3_·OEt_2_ under the same reaction conditions, providing 60, 92 and 91% yields of oxazole derivatives **7b**–**d** (Scheme 6). This synthetic methodology was extended to the use of *N*-acylaziridines **5** derived from phosphonate. Thus, the ring expansion of **5a** and **5b** easily occurred via the slight excess of BF_3_·OEt_2_ (3 equivalents) in CHCl_3_ at 71 °C and under microwave irradiation for 15 min, to obtain regio- and diasteroselective **7e** and **7f** in high yields (Scheme 6).

**Figure 4:**
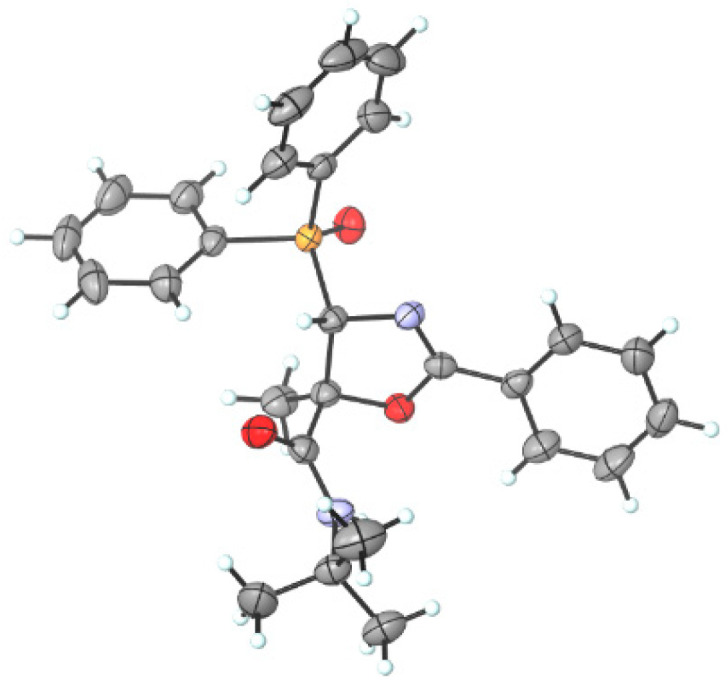
ORTEP diagram of functionalized diphenylphosphoryl oxazole derivative **7a** (H, white; C, grey; O, red; N, blue; P, orange) (4*S*,5*S* enantiomer shown).

Since it was not possible to assign the stereochemistry of oxazole derivatives **7** via ^1^H and ^13^C NMR, their structure has been unambiguously determined via X-ray diffraction analysis [51,52,53], establishing not only the regioselectivity of the isomerization process, but also the *anti*-relationship between the amide group at the C5 position and the phosphorus moiety at the C4 position of **7a** (Figure 4).

**Scheme 7:**
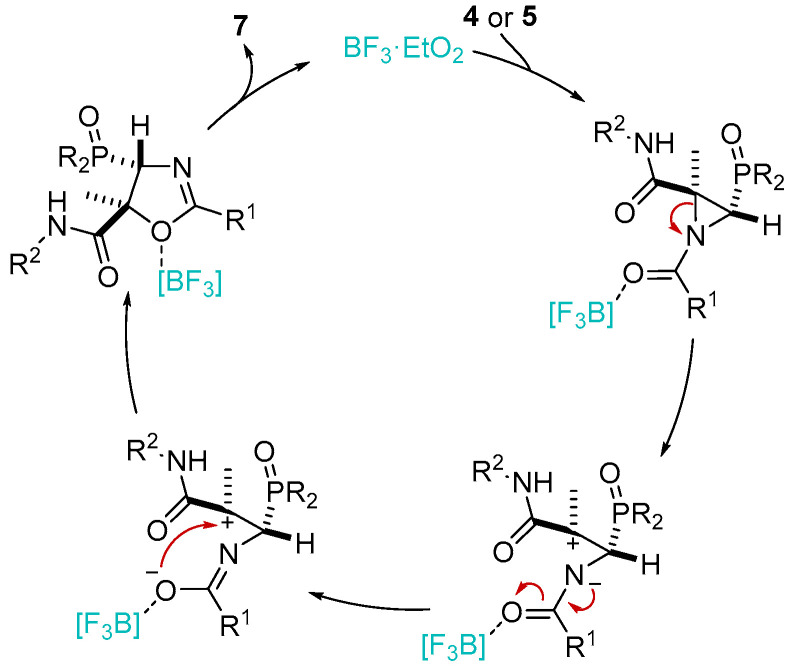
Rational mechanism for the stereospecific and regioselective ring expansion of *N*-acylaziridines **4** or **5** to oxazole derivatives **7**.

A reasonable mechanism that would explain the formation of **7** is exemplified in Scheme 7. First, BF_3_·OEt_2_ would activate the carbonyl group of *N*-acylaziridine **4** or **5**, thus assisting the ring-opening reaction, through the N–C2 bond of *N*-acylaziridine, with the concomitant generation of the most stable carbocation. This intermediate enables the ring expansion of *N*-acylaziridine **4** or **5** to oxazole derivative **7**, as the only regio- and diastereoisomer.

## 3. Materials and Methods

### 3.1. General Experimental Information

Solvents for extraction and chromatography were reagent-grade. All solvents used in reactions were freshly distilled and dried over 4 Å molecular sieves before use. Unless otherwise mentioned, all other solvents and chemicals were purchased from commercial vendors and recrystallized or distilled as necessary, or used without further purification. All reactions were performed under an atmosphere of dry nitrogen. The reaction progress was monitored via ^31^P NMR or analytical thin-layer chromatography (TLC) performed on precoated Merck silica gel 60 F_254_ TLC aluminum plates, and spot-visualized with UV light or permanganate stain. Melting points were uncorrected. ^1^H (400 MHz), ^13^C (100 MHz), ^19^F (376 MHz) and ^31^P NMR (160 MHz) spectra were recorded using a Bruker Avance 400 (400 MHz) spectrometer in CDCl_3_ at room temperature. Chemical shifts (*δ*) are reported in parts per million (ppm) with the internal chloroform signal at 7.26 ppm as a standard for ^1^H, the internal chloroform signal at 77.2 ppm as a standard for ^13^C, the external fluorotrichloromethane (CFCl_3_) signal at 0.0 ppm for ^19^F or the external H_3_PO_4_ (50%) signal at 0.0 ppm as a standard for ^31^P NMR spectra. All coupling constants (*J*) values are reported in Hz. ^19^F and ^13^C NMR spectra were recorded in a broadband decoupled mode from hydrogen nuclei. Distortionless enhanced polarization transfer (DEPT) supported peak assignments for ^13^C NMR. Data for 1H NMR spectra are reported for the following: chemical shift, multiplicity, coupling constant and integration. Multiplicity abbreviations are reported as s = singlet; d = doublet; t = triplet; q = quartet; m = multiplet; dd = double doublet; bs = broad singlet. IR spectra were measured using a Nicolet iS10 Termo Scientific spectrometer using an attenuated total reflectance technique (ATR). Absorbance frequencies are given at maximum of intensity in cm^–1^. High-resolution mass spectra (HRMS) were obtained via the positive-ion electrospray ionization (ESI) method with a time of flight Q-TOF method. Data are reported in the form *m*/*z* (intensity relative to base = 100). 2*H*-Azirine phosphine oxide **1a** [58] and phosphonate **1b** [59] were prepared according to procedures in the literature and characterized using NMR spectra.

### 3.2. Experimental Procedure and Characterization Data for N-Acylaziridine Phosphine Oxide ***4***

In a flame-dried flask, the corresponding carboxylic acid **2** (0.65 mmol, 1.3 eq.), isocyanide **3** (0.65 mmol, 1.3 eq.) and 1M diethyl ether solution of ZnCl_2_ (0.12 mL; 0.12 mmol; 0.25 eq.) were added to 0.5 mL of dry THF. Then, 2*H*-azirine phosphine oxide **1a** (0.50 mmol, 1 eq.) was added at room temperature. The reaction mixture was stirred until TLC showed the disappearance of 2*H*-azirine **1a** (1–24 h). The solvent was removed under vacuum, and the residue was dissolved in dichloromethane (5 mL) and washed with water (2 × 5 mL). The organic layer was dried over anhydrous MgSO_4_ and filtered, and the solvent was evaporated under reduced pressure. The resulting residue was purified via flash column chromatography (SiO_2_, hexanes/AcOEt) to yield compounds **4**.

(2S*,3S*)-1-Benzoyl-*N*-cyclohexyl-3-(diphenylphosphoryl)-2-methylaziridine-2-carboxamide (**4a**) (182 mg, 75%) was obtained as a white solid from carboxylic acid **2a** (79 mg, 0.65 mmol), isocyanide **3a** (81 µL mg, 0.65 mmol) and 2*H*-azirine **1a** (127 mg, 0.50 mmol), as described in the general procedure. The crude product was purified via flash column chromatography (SiO_2_, AcOEt/hexanes 50:50) to give the title compound **4a**. mp 249–251 °C; IR (neat) *v_max_* 3281, 3070, 2923, 2848, 1685, 1651, 1549, 1285, 1193 cm^–1^; ^1^H NMR (400 MHz, CDCl_3_) *δ* 8.01–7.95 (m, 4H, ArH), 7.84–7.82 (m, 2H, ArH), 7.62–7.47 (m, 7H, ArH), 7.40–7.37 (m, 2H, ArH), 5.76 (d, ^2^*J*_HH_ = 8.2 Hz, 1H, HC-NH), 3.81 (d, ^2^*J*_PH_ = 24.0 Hz, 1H, CH-P), 3.50–3.40 (m, 1H, HC-NH), 1.96 (s, 3H, CH_3_), 1.76–1.73 (m, 1H, *^c^*Hex), 1.63–1.59 (m, 1H, *^c^*Hex), 1.53–1.45 (m, 2H, *^c^*Hex), 1.27–0.98 (m, 5H, *^c^*Hex), 0.73–0.63 (m, 1H, *^c^*Hex) ppm; ^13^C {1H} NMR (100 MHz, CDCl_3_) *δ* 176.9 (d, ^3^*J*_PC_ = 3.1 Hz), 165.6), 134.2, 132.6 (d, ^1^*J*_PC_ = 103.4 Hz, C_quat_), 132.5, 132.4, 132.4, 132.3, 132.3, 131.8, 131.7, 131.2, 131.1, 129.1, 129.0, 128.8, 128.6, 128.5, 49.7 (d, ^2^*J*_PC_ = 2.7 Hz, C_quat_), 49.1, 42.2 (d, ^1^*J*_PC_ = 101.0 Hz), 32.5, 32.4, 25.4, 24.7, 24.6, 15.5 ppm; ^31^P NMR (160 MHz, CDCl_3_) *δ* 24.1 ppm; ESI-HRMS (CI) *m*/*z* calculated for C_29_H_32_N_2_O_3_P ([M + H]^+^), 487.2151; found 487.21567.

(2S*,3S*)-*N*-Cyclohexyl-3-(diphenylphosphoryl)-2-methyl-1-(3-methylbenzoyl)aziridine-2-carboxamide (**4b**) (158 mg, 63%) was obtained as a white solid from carboxylic acid **2b** (88 mg, 0.65 mmol), isocyanide **3a** (81 µL, 0.65 mmol) and 2*H*-azirine **1a** (127 mg, 0.50 mmol), as described in the general procedure. The crude product was purified via flash column chromatography (SiO_2_, AcOEt/hexane 50:50) to give the title compound **4b**. mp 201–203 °C; IR (neat) *v_max_* 3303, 3056, 2939, 1679, 1643, 1538, 1293, 1191 cm^–1^; ^1^H NMR (400 MHz, CDCl_3_) *δ* 7.90–7.85 (m, 4H, ArH), 7.59 (s, 1H, ArH), 7.59–7.39 (m, 7H, ArH), 7.21–7.13 (m, 2H, ArH), 5.81 (d, ^3^*J*_HH_ = 8.2 Hz, 1H, HC-NH), 3.78 (d, ^2^*J*_PC_ = 24.2 Hz, 1H, CH-P), 3.42–3.33 (m, 1H, HC-NH), 2.26 (s, 3H, CH_3_), 1.86 (s, 3H, CH_3_), 1.65–1.63 (m, 1H, *^c^*Hex), 1.52–1.49 (m, 1H, *^c^*Hex), 1.43–1.36 (m, 2H, *^c^*Hex), 1.18–0.90 (m, 5H, *^c^*Hex), 0.70–0.61 (m, 1H, *^c^*Hex) ppm; ^13^C {1H} NMR (100 MHz, CDCl_3_) *δ* 176.2 (d, ^3^*J*_PC_ = 3.3 Hz), 165.6, 138.2, 133.9, 133.2, 132.3, 132.2, 132.2, 132.1, 131.7, 131.6 131.1, 131.0, 129.1, 129.0, 128.9, 128.7, 128.6, 128.2, 125.5, 49.6 (d,^2^ *J*_PC_ = 3.0 Hz), 49.0, 42.0 (d, ^1^*J*_PC_ = 100.4 Hz), 32.5, 32.4, 25.3, 24.7, 24.6, 21.4, 15.4 ppm; ^31^P NMR (160 MHz, CDCl_3_) *δ* 23.9 ppm; ESI-HRMS (CI) *m*/*z* calculated for C_30_H_34_N_2_O_3_P ([M + H]^+^), 501.2307; found 501.2308.

(2S*,3S*)-*N*-Cyclohexyl-3-(diphenylphosphoryl)-2-methyl-1-(4-nitrobenzoyl)aziridine-2-carboxamide (**4c**) (135 mg, 51%) was obtained as a white solid from carboxylic acid **2c** (108 mg, 0.65 mmol), isocyanide **3a** (81 µL, 0.65 mmol) and 2*H*-azirine **1a** (127 mg, 0.50 mmol), as described in the general procedure. The crude product was purified via flash column chromatography (SiO_2_, AcOEt/hexane 50:50) to give the title compound **4c**. mp 254–256 °C; IR (neat) *v_max_* 3281, 3078, 2923, 1737, 1687, 1649, 1596, 1554, 1501, 1390, 1285, 1193 cm^–1^; ^1^H NMR (400 MHz, CDCl_3_) *δ* 8.20 (d, ^3^*J*_HH_ = 8.7 Hz, 2H, ArH), 7.96–7.78 (m, 6H, ArH), 7.59–7.48 (m, 6H, ArH), 5.88 (d, ^3^*J*_HH_ = 7.8 Hz, 1H, HC-NH), 3.68 (d, ^2^*J*_PH_ = 23.5 Hz, 1H, CH-P), 3.49–3.38 (m, 1H, HC-NH), 1.95 (s, 3H, CH_3_), 1.73 (d, ^3^*J*_HH_ = 12.2 Hz, 1H, *^c^*Hex), 1.60 (d, ^3^*J*_HH_ = 14.4 Hz, 1H, *^c^*Hex), 1.52–1.45 (m, 2H, *^c^*Hex), 1.34 (d, ^3^*J*_HH_ = 12.4, 1H, *^c^*Hex), 1.23–1.03 (m, 4H, *^c^*Hex), 0.83–0.75, 1H, *^c^*Hex) ppm; ^13^C {1H} NMR (100 MHz, CDCl_3_) *δ* 175.2 (d, ^3^*J*_PC_ = 3.2 Hz), 165.7, 150.0, 139.8, 132.6, 136.6, 132.6, 132.5, 132.2 (d, ^1^*J*_PC_ = 103.9 Hz), 131.6, 131.5, 131.3 (d, ^1^*J*_PC_ = 105.2 Hz), 131.1, 131.0, 129.3, 129.2, 129.1, 128.9, 128.8. 123.7, 50.0 (d, ^2^*J*_PC_ = 2.73 Hz), 49.4, 42.7 (d, ^1^*J*_PC_ = 99.2 Hz), 32.6, 32.5, 25.3, 24.7, 24.6, 15.3 ppm; ^31^P NMR (160 MHz, CDCl_3_) *δ* 23.5 ppm; ESI-HRMS (CI) *m*/*z* calculated for C_29_H_31_N_3_O_5_P ([M + H]^+^), 532.2001; found 532.1980.

(2S*,3S*)-*N*-Cyclohexyl-3-(diphenylphosphoryl)-1-(4-fluorobenzoyl)-2-methylaziridine-2-carboxamide (**4d**) (186 mg, 74%) was obtained as a white solid from carboxylic acid **2d** (91 mg, 0.65 mmol), isocyanide **3a** (81 µL, 0.65 mmol) and 2*H*-azirine **1a** (127 mg, 0.50 mmol), as described in the general procedure. The crude product was purified via flash column chromatography (SiO_2_, AcOEt/hexane 50:50) to give the title compound **4d**. mp 255–257 °C; IR (neat) *v_max_* 3284, 3018, 2939, 1688, 1650, 1590, 1286, 1102 cm^–1^; ^1^H NMR (400 MHz, CDCl_3_) *δ* 7.96–7.91 (m, 4H, ArH), 7.82 (dd, ^3^*J*_HH_ = 8.8, ^4^*J*_HH_ = 5.4 Hz, 2H), 7.60–7.46 (m, 6H, ArH), 7.04 (t, ^3^*J*_HH_ = 8.6 Hz, ^3^*J*_HF_ = 8.6 Hz, 2H, ArH), 5.77 (d, ^3^*J*_HH_ = 8.2 Hz, 1H, HC-NH), 3.75 (d, ^2^*J*_PH_ = 24.0 Hz, 1H, CH-P), 3.48 (m, 1H, HC-NH), 1.93 (s, 3H, CH_3_), 1.75–1.72 (m, 1H, *^c^*Hex), 1.62–1.59 (m, 1H, *^c^*Hex), 1.53–1.48 (m, 2H, *^c^*Hex), 1.22–1.17 (m, 2H, *^c^*Hex), 1.12–1.00 (m, 3H, *^c^*Hex), 0.79–0.70 (m, 1H, *^c^*Hex) ppm; ^13^C {1H}NMR (100 MHz, CDCl_3_) *δ* 175.7 (d, ^3^*J*_PC_ = 3.2 Hz), 165.6, 165.4 (d, ^1^*J*_CF_ = 253.3 Hz), 132.5 (d, ^1^*J*_PC_ = 103.6 Hz, C_quat_), 132.4, 132.4, 132.4, 132.3, 132.2, 131.7, 131.1, 131.0, 130.9, 130.8, 130.6 (d, ^4^*J*_CF_ = 3.0 Hz), 129.1, 129.0, 128.8, 115.7, 115.5, 49.7 (d, ^2^*J*_PC_ = 3.0 Hz), 49.0, 42.2 (d, ^1^*J*_PC_ = 100.4 Hz), 32.5, 32.5, 25.4, 24.7, 24.6, 15.4 ppm; ^31^P NMR (160 MHz, CDCl_3_) *δ* 24.4 ppm; ^19^F NMR (376 MHz, CDCl_3_) *δ* –106.5 ppm; ESI-HRMS (CI) *m*/*z* calculated for C_29_H_31_FN_2_O_3_P ([M + H]^+^), 505.2056; found 505.2043.

(2S*,3S*)-*N*-Cyclohexyl-3-(diphenylphosphoryl)-1-(4-methoxybenzoyl)-2-methylaziridine-2-carboxamide (**4e**) was (51 mg, 20%) obtained as a white solid from carboxylic acid **2e** (100 mg, 0.65 mmol), isocyanide **3a** (81 µL, 0.65 mmol) and 2*H*-azirine **1a** (127 mg, 0.50 mmol), as described in the general procedure. The crude product was purified via flash column chromatography (SiO_2_, AcOEt/hexane 50:50) to give the title compound **4e**. mp 251–252 °C; IR (neat) *v_max_* 3278, 3075, 2923, 1685, 1651, 1596, 1549, 1282, 1196, 1121 cm^–1^; ^1^H NMR (400 MHz, CDCl_3_) *δ* 7.96–7.92 (m, 4H, ArH), 7.77 (d, ^3^*J*_HH_ = 8.9 Hz, 2H, ArH), 7.57–7.44 (m, 6H, ArH), 6.84 (d, ^3^*J*_HH_ = 8.9 Hz, 2H, ArH), 5.82 (d, ^3^*J*_HH_ = 7.9 Hz, 1H, HC-NH), 3.80 (s, 3H, OCH_3_), 3.76 (d, ^1^*J*_PC_ = 24.2 Hz, 1H, CH-P), 3.51–3.40 (m, 1H, HC-NH), 1.92 (s, 3H, CH_3_), 1.73–1.69 (m, 1H, *^c^*Hex), 1.59–1.56 (m, 1H, *^c^*Hex), 1.59–1.47 (m, 2H, *^c^*Hex), 1.31–0.96 (m, 5H, *^c^*Hex), 0.77–0.68 (m, 1H, *^c^*Hex) ppm; ^13^C {1H} NMR (100 MHz, CDCl_3_) *δ* 176.0 (d, ^3^*J*_PC_ = 3.3 Hz), 165.6, 163.1, 133.2, 132.3, 132.3, 132.2, 132.2, 131.8, 131.7, 131.3, 131.1, 131.0, 130.5, 129.0, 128.9, 128.7, 128.6, 126.7, 113.7, 55.5, 49.7 (d, ^2^*J*_PC_ = 2.9 Hz, C_quat_), 49.0, 41.9 (d, ^1^*J*_PC_ = 101.0 Hz), 32.5, 32.4, 25.4, 24.7, 24.6, 15.5 ppm; ^31^P NMR (160 MHz, CDCl_3_) *δ* 24.0 ppm; ESI-HRMS (CI) *m*/*z* calculated for C_30_H_34_N_2_O_4_P ([M + H]^+^), 517.2256; found 517.2257.

(2S*,3S*)-*N*-Cyclohexyl-3-(diphenylphosphoryl)-2-methyl-1-(2-phenylacetyl)aziridine-2-carboxamide (**4f**) (170 mg, 68%) was obtained as a white solid from carboxylic acid **2m** (88 mg, 0.65 mmol), isocyanide **3a** (81 µL, 0.65 mmol) and 2*H*-azirine **1a** (127 mg, 0.50 mmol), as described in the general procedure. The crude product was purified via flash column chromatography (SiO_2_, AcOEt/hexane 60:40) to give the title compound **4f**. mp 245–247 °C; IR (neat) *v_max_* 3311, 3056, 2978, 1691, 1653, 1524, 1254, 1182 cm^–1^; ^1^H NMR (400 MHz, CDCl_3_) *δ* 7.97–7.91 (m, 2H, ArH), 7.85–7.80 (m, 2H, ArH), 7.54–7.41 (m, 6H, ArH), 7.22–7.18 (m, 3H, ArH), 7.12 (dd, ^3^*J*_HH_ = 7.6, ^4^*J*_HH_ = 1.8, 2H, ArH), 5.99 (bs, 1H, HC-NH), 3.74–3.63 (m, 1H, HC-NH), 3.68 (d, ^2^*J*_HH_ = 16.4 Hz, 1H, CH_2_), 3.57 (d, ^2^*J*_HH_ = 16.5 Hz, 1H, CH_2_), 3.40 (d, ^2^*J*_PH_ = 23.7 Hz, 1H, CH-P), 1.91–1.87 (m, 1H, *^c^*Hex), 1.83–1.79 (m, 1H, *^c^*Hex), 1.70–1.57 (m, 3H, *^c^*Hex), 1.39–1.25 (m, 2H, *^c^*Hex), 1.31 (s, 3H, CH_3_), 1.19–1.09 (m, 3H, *^c^*Hex); ^13^C {1H} NMR (100 MHz, CDCl_3_) *δ* 180.8 (d, ^3^*J*_PC_ = 2.1 Hz), 166.8, 134.2, 133.2, 132.3, 132.3, 132.2, 132.1, 131.7, 131.6, 131.2 (d, ^1^*J*_PC_ = 103.5 Hz) 131.0, 130.7, 130.1, 129.0, 128.9, 128.6, 128.5, 128.4, 126.9, 49.5, 49.1 (d, ^2^*J*_PC_ = 2.9 Hz), 44.4, 42.2 (d, ^1^*J*_PC_ = 101.3 Hz), 32.9, 32.7, 25.3, 24.9, 24.8, 24.7, 14.8 ppm; ^31^P NMR (160 MHz, CDCl_3_) *δ* 24.4 ppm; ESI-HRMS (CI) *m*/*z* calculated for C_30_H_34_N_2_O_3_P ([M + H]^+^), 501.2307; found 501.2290.

(2S*,3S*)-*N*-Cyclohexyl-3-(diphenylphosphoryl)-2-methyl-1-propioloylaziridine-2-carboxamide (**4g**) (33 mg, 15%) was obtained as a pale orange solid from carboxylic acid **2p** (40 µL, 0.65 mmol), isocyanide **3a** (81 µL, 0.65 mmol) and 2*H*-azirine **1a** (127 mg, 0.50 mmol), as described in the general procedure. The crude product was purified via flash column chromatography (SiO_2_, AcOEt/hexane 50:50) to give the title compound **4g**. mp 152–153 °C; IR (neat) *v_max_* 3270, 3059, 2923, 2098, 1674, 1658, 1587, 1371, 1188 cm^–1^; ^1^H NMR (400 MHz, CDCl_3_) *δ* 7.91–7.86 (m, 4H, ArH), 7.57–7.51 (m, 6H, ArH), 6.06 (d, ^3^*J*_HH_ = 8.1 Hz, 1H, HC-NH), 3.81–3.75 (m, 1H, HC-NH), 3.67 (d, ^2^*J*_PH_ = 23.9 Hz, 1H, CH-P), 2.80 (s, 1H, C≡CH), 1.93–1.92 (m, 2H, *^c^*Hex), 1.85 (s, 3H, CH_3_), 1.77–1.73 (m, 2H, *^c^*Hex), 1.68–1.58 (m, 1H, *^c^*Hex), 1.41–1.26 (m, 2H, *^c^*Hex), 1.25–1.11 (m, 3H, *^c^*Hex) ppm; ^13^C {1H} NMR (100 MHz, CDCl_3_) *δ* 165.5, 160.3, 132.7, 132.6, 132.5, 132.5, 131.9 (d, ^1^*J*_PC_ = 103.4 Hz), 131.8, 131.7, 131.2 (d, ^1^*J*_PC_ = 105.3 Hz), 131.1, 131.0, 129.2, 129.0, 128.8, 128.7, 76.7, 49.8, 49.3 (d, ^2^*J*_PC_ = 2.2 Hz), 43.9 (d, ^1^*J*_PC_ = 98.0 Hz), 33.2, 32.8, 25.5, 24.9, 24.9, 14.7 ppm; ^31^P NMR (160 MHz, CDCl_3_) *δ* 22.8 ppm; ESI-HRMS (CI) *m*/*z* calculated for C_25_H_28_N_2_O_3_P ([M + H]^+^), 435.1838; found 435.1838.

(2S*,3S*)-*N*-Cyclohexyl-3-(diphenylphosphoryl)-2-methyl-1-nicotinoylaziridine-2-carboxamide (**4h**) (104 mg, 43%) was obtained as a white solid from carboxylic acid **2f** (80 mg, 0.65 mmol), isocyanide **3a** (81 µL, 0.65 mmol) and 2*H*-azirine **1a** (127 mg, 0.50 mmol), as described in the general procedure. The crude product was purified via flash column chromatography (SiO_2_, AcOEt/hexane 50:50) to give the title compound **4h**. mp 234–236 °C; IR (neat) *v_max_* 3275, 3078, 3053, 2925, 1690, 1651, 1543, 1318, 1199, 1127 cm^–1^; ^1^H NMR (400 MHz, CDCl_3_) *δ* 8.95 (s, 1H, ArH), 8.68 (d, ^3^*J*_HH_ = 4.60 Hz, 1H, ArH), 8.14 (d, ^3^*J*_HH_ = 8.0 Hz, 1H, ArH), 7.96–7.90 (m, 4H, ArH), 7.57–7.48 (m, 6H, ArH), 7.34 (dd, ^3^*J*_HH_ = 7.7 Hz, ^3^*J*_HH_ = 4.60 Hz, 1H, ArH), 5.86 (bs, 1H, HC-NH), 3.76 (d, ^2^*J*_PH_ = 23.5 Hz, 1H, CH-P), 3.45–3.37 (m, 1H, HC-NH), 1.95 (s, 3H, CH_3_), 1.72 (d, ^3^*J*_HH_ = 11.7 Hz, 1H, *^c^*Hex), 1.61–1.57 (m, 1H, *^c^*Hex), 1.51–1.45 (m, 2H, *^c^*Hex), 1.27–0.99 (m, 5H, *^c^*Hex), 0.78–0.69 (m, 1H, *^c^*Hex) ppm; ^13^C {1H} NMR (100 MHz, CDCl_3_) *δ* 175.4 (d, ^3^*J*_PC_ = 3.4 Hz), 165.5, 152.9, 149.4, 132.5, 132.5, 132.5, 132.4, 132.4 (d, ^1^*J*_PC_ = 103.8 Hz), 131.7, 131.4 (d, ^1^*J*_PC_ = 105.4 Hz), 131.6, 131.1, 131.0, 130.0), 129.1, 129.0, 128.8, 128.7, 123.6, 50.0, 49.4, 42.5 (d, ^1^*J*_PC_ = 100.1 Hz), 32.5, 32.5, 25.3, 24.7, 24.6, 15.3 ppm; ^31^P NMR (160 MHz, CDCl_3_) *δ* 23.7 ppm; ESI-HRMS (CI) *m*/*z* calculated for C_28_H_31_N_3_O_3_P ([M + H]^+^), 488.2103; found 488.2102.

(2S*,3S*)-1-Benzoyl-*N*-(*tert*-butyl)-3-(diphenylphosphoryl)-2-methylaziridine-2-carboxamide (**4i**) (184 mg, 80%) was obtained as a white solid from carboxylic acid **2a** (79 mg, 0.65 mmol), isocyanide **3b** (79 µL, 0.65 mmol) and 2*H*-azirine **1a** (127 mg, 0.50 mmol), as described in the general procedure. The crude product was purified via flash column chromatography (SiO_2_, AcOEt/hexane 50:50) to give the title compound **4i**. mp 177–179 °C; IR (neat) *v_max_* 3319, 3050, 2967, 2923, 1699, 1682, 1699, 1601, 1535, 1232, 1190 cm^–1^; ^1^H NMR (400 MHz, CDCl_3_) *δ* 8.03–7.93 (m, 4H, ArH), 7.80–7.78 (m, 2H, ArH), 7.59–7.45 (m, 7H, ArH), 7.36 (t, ^3^*J*_HH_ = 7.6 Hz, 2H, ArH), 5.66 (s, 1H, NH), 3.80 (d, ^2^*J*_PH_ = 23.6 Hz, 1H, CH-P), 1.89 (s, 3H, CH_3_), 0.98 (s, 9H, *^t^*Bu) ppm; ^13^C {1H} NMR (100 MHz, CDCl_3_) *δ* 177.1 (d, ^3^*J*_PC_ = 3.1 Hz), 165.3, 134.3, 132.9 (d, ^1^*J*_PC_ = 103.5 Hz), 132.5, 132.3, 132.3, 132.2, 132.2, 131.8, 131.7, 131.6 (d, ^1^*J*_PC_ = 105.3 Hz) 131.2, 131.1, 129.0, 128.9, 128.7, 128.6, 128.5, 128.4, 52.1, 50.0 (d, ^2^*J*_PC_ = 3.0 Hz, C_quart_), 42.3 (d, ^1^*J*_PC_ = 101.3 Hz), 28.1, 15.8 ppm; ^31^P NMR (160 MHz, CDCl_3_) 23.9 ppm; *δ* ESI-HRMS (CI) *m*/*z* calculated for C_27_H_30_N_2_O_3_P ([M + H]^+^), 461.1994; found 461.1995.

2S*,3S*-*N*-(*tert*-Butyl)-3-(diphenylphosphoryl)-1-(4-fluorobenzoyl)-2-methylaziridine-2-carboxamide (**4j**) (177mg, 74%) was obtained as a white solid from carboxylic acid **2d** (91 mg, 0.65 mmol), isocyanide **3b** (76 µL, 0.65 mmol) and 2*H*-azirine **1a** (127 mg, 0.50 mmol), as described in the general procedure. The crude product was purified via flash column chromatography (SiO_2_, AcOEt/hexane 50:50) to give the title compound **4j**. mp 221–223 °C; IR (neat) *v_max_* 3297, 3053, 2964, 1685, 1665, 1524, 1282, 1196 cm^–1^; ^1^H NMR (400 MHz, CDCl_3_) *δ* 8.01–7.91 (m, 4H, ArH), 7.83–7.79 (m, 2H, ArH), 7.59–7.44 (m, 6H, ArH), 7.04 (t, ^3^*J*_HH_ = 8.6 Hz, ^3^*J*_HF_ = 8.6 Hz, 2H, ArH), 5.69 (s, 1H, NH), 3.77 (d, ^2^*J*_PH_ = 23.5 Hz, 1H, CH-P), 1.89 (s, 3H, CH_3_), 1.03 (s, 9H, *^t^*Bu) ppm; ^13^C {1H} NMR (100 MHz, CDCl_3_) *δ* 176.0 (d, ^3^*J*_PC_ = 2.8 Hz), 165.4 (d, ^1^*J*_CF_ = 253.3 Hz), 165.3, 133.3, 132.4, 132.3, 132.3, 132.1, 131.8, 131.7, 131.2, 131.1, 131.0, 130.9, 130.8 (d, ^4^*J*_CF_ = 3.0 Hz), 129.1, 129.0, 128.8, 128.7, 115.6 (d, ^2^*J*_CF_ = 21.9 Hz, ArC), 52.3, 50.1, 42.2 (d, ^1^*J*_PC_ = 101.0 Hz, 28.2, 15.7 ppm; ^31^P NMR (160 MHz, CDCl_3_) 24.0 ppm; ^19^F NMR (376 MHz, CDCl_3_) *δ* –106.5 ppm; ESI-HRMS (CI) *m*/*z* calculated for C_27_H_29_FN_2_O_3_P ([M + H]^+^), 479.1900; found, 479.1904.

(2S*,3S*)-*N*-(*tert*-Butyl)-3-(diphenylphosphoryl)-1-(4-methoxybenzoyl)-2-methylaziridine-2-carboxamide(**4k**) (123 mg, 50%) was obtained as a white solid from carboxylic acid **2e** (100 mg, 0.65 mmol), isocyanide **3b** (76 µL, 0.65 mmol) and 2*H*-azirine **1a** (127 mg, 0.50 mmol), as described in the general procedure. The crude product was purified via flash column chromatography (SiO_2_, AcOEt/hexane 45:55) to give the title compound **4k**. mp > 275 °C; IR (neat) *v_max_* 3250, 2961, 1671, 1660, 1601, 1066 cm^–1^; ^1^H NMR (400 MHz, CDCl_3_) *δ* 8.01–7.92 (m, 4H, ArH), 7.77 (d, ^3^*J*_HH_ = 8.9 Hz, 2H, ArH), 7.54–7.45 (m, 6H, ArH), 6.85 (d, ^3^*J*_HH_ = 8.9 Hz, 2H, ArH), 5.67 (s, 1H, NH), 3.82 (s, 3H, CH_3_), 3.79 (d, ^2^*J*_PH_ = 23.8 Hz, 1H, CH-P), 1.89 (s, 3H, CH_3_), 1.01 (s, 9H, *^t^*Bu) ppm; ^13^C {1H} NMR (100 MHz, CDCl_3_) *δ* 176.2 (d, ^3^*J*_PC_ = 3.3 Hz), 165.3, 163.1 (C_quat_), 133.0 (d, ^1^*J*_PC_ = 103.3 Hz), 132.3, 132.2, 132.2, 131.8, 131.7 (ArC), 131.2 (C_quat_), 131.2, 131.1, 130.6, 129.0, 128.9, 128.7, 128.6, 126.9, 113.7, 55.6, 52.1, 50.1 (d, ^2^*J*_PC_ = 3.1 Hz, C_quat_), 42.0 (d, ^1^*J*_PC_ = 101.5 Hz), 28.2, 15.8 ppm; ^31^P NMR (160 MHz, CDCl_3_) *δ* 24.1 ppm ESI-HRMS (CI) *m*/*z* calculated for C_28_H_32_N_2_O_4_P ([M + H]^+^), 491.2100; found 491.2101.

(2S*,3S*)-1-(2-Naphthoyl)-*N*-(*tert*-butyl)-3-(diphenylphosphoryl)-2-methylaziridine-2-carboxamide (**4l**) (178 mg, 70%) was obtained as a white solid from carboxylic acid **2g** (112 mg, 0.65 mmol), isocyanide **3b** (76µL, 0.65 mmol) and 2*H*-azirine **1a** (127 mg, 0.50 mmol), as described in the general procedure. The crude product was purified via flash column chromatography (SiO_2_, AcOEt/hexane 50:50) to give the title compound **4l**. mp 265–267 °C; IR (neat) *v_max_* 3381, 3061, 2970, 1682, 1662, 1529, 1293, 1196 cm^–1^; 1H NMR (400 MHz, CDCl_3_) *δ* 8.33 (s, 1H, ArH), 8.04–7.93 (m, 4H, ArH), 7.88–7.81 (m, 4H, ArH), 7.58–7.43 (m, 8H, ArH) 5.66 (s, 1H, NH), 3.84 (d, ^2^*J*_PH_ = 23.6 Hz, 1H, CH-P), 1.98 (s, 3H, CH_3_), 0.88 (s, 9H, *^t^*Bu) ppm; ^13^C {1H} NMR (100 MHz, CDCl_3_) *δ* 177.2 (d, ^3^*J*_PC_ = 3.3 Hz), 165.4, 135.4, 133.0 (d, ^1^*J*_PC_ = 103.6 Hz), 132.5, 132.4, 132.3, 132.3, 132.2, 131.8, 131.7, 131.2, 131.1, 129.6, 129.3, 129.1, 129.0, 128.7, 128.6, 128.3, 128.2, 127.9, 126.8, 124.7, 51.1, 50.2 (d, ^2^*J*_PC_ = 2.9 Hz), 42.4 (d, ^1^*J*_PC_ = 100.5 Hz), 28.1, 15.7 ppm; ^31^P NMR (160 MHz, CDCl_3_) *δ* 23.9 ppm; ESI-HRMS (CI) *m*/*z* calculated for C_31_H_32_N_2_O_3_P [M + H]^+^), 511.2151; found 511.2152.

(2S*,3S*)-*N*-(*tert*-Butyl)-3-(diphenylphosphoryl)-1-(furan-2-carbonyl)-2-methylaziridine-2-carboxamide (**4m**) (191 mg, 85%) was obtained as a white solid from carboxylic acid **2h** (73 mg, 0.65 mmol), isocyanide **3b** (76 µL, 0.65 mmol) and 2*H*-azirine **1a** (127 mg, 0.50 mmol), as described in the general procedure. The crude product was purified via flash column chromatography (SiO_2_, AcOEt/hexane 45:55) and recrystallized from diethyl ether to give the title compound **4m**. mp 222–224 °C; IR (neat) *v_max_* 3317, 3056, 2959, 1674, 1576, 1296, 1118 cm^–1^; ^1^H NMR (400 MHz, CDCl_3_) *δ* 8.02–7.91 (m, 4H, ArH), 7.59–7.45 (m, 6H), 7.31 (dd, ^3^*J*_HH_ = 1.7 Hz, ^4^*J*_HH_ = 0.8 Hz, 1H), 7.14 (dd, ^3^*J*_HH_ = 3.5, ^4^*J*_HH_ = 0.9 Hz, 1H), 6.44 (dd, ^3^*J*_HH_ = 3.5 Hz, ^3^*J*_HH_ = 1.7 Hz, 1H), 5.85 (s, 1H, NH), 3.69 (d, ^2^*J*_PH_ = 23.3 Hz, 1H, CH-P), 1.86 (s, 3H, CH_3_), 1.18 (s, 9H, *^t^*Bu) ppm; ^13^C {1H} NMR (100 MHz, CDCl_3_) *δ* 166.4 (d, ^3^*J*_PC_ = 3.5 Hz), 166.0, 148.7, 145.0, 133.0 (d, ^1^*J*_PC_ = 103.5 Hz), 132.3, 132.2, 132.1, 131.8, 131.7, 131.1, 131.0, 129.0, 128.9, 128.7, 128.6, 116.5, 112.3, 52.0, 49.8 (d, ^2^*J*_PC_ = 3.2 Hz, C_quat_), 42.3 (d, ^1^*J*_PC_ = 101.0 Hz), 28.3, 15.6 ppm; ^31^P NMR (160 MHz, CDCl_3_) *δ* 23.6 ppm; ESI-HRMS (CI) *m*/*z* calculated for C_25_H_28_N_2_O_4_P ([M + H]^+^), 451.1787; found 451.1789.

(2S*,3S*)-1-(4-Benzoylbenzoyl)-*N*-(*tert*-butyl)-3-(diphenylphosphoryl)-2-methylaziridine-2-carboxamide (**4n**) (57 mg, 20%) was obtained as a white solid from carboxylic acid **2i** (147 mg, 0.65 mmol), isocyanide **3b** (76 µL, 0.65 mmol) and 2*H*-azirine **1b** (127 mg, 0.50 mmol), as described in the general procedure. The crude product was purified via flash column chromatography (SiO_2_, AcOEt/hexane 50:50) to give the title compound **4n**. mp 103–105 °C; IR (neat) *v_max_* 3385, 3062, 2974, 1694, 1656, 1524, 1273, 1188 cm^–1^; ^1^H NMR (400 MHz, CDCl_3_) *δ* 8.02–7.88 (m, 6H, ArH), 7.78–7.74 (m, 4H, ArH), 7.63–7.46 (m, 9H, ArH), 5.71 (s, 1H, NH), 3.78 (d, ^2^*J*_PH_ = 23.3 Hz, 1H, CH-P), 1.91 (s, 3H, CH_3_), 1.04 (s, 9H, *^t^*Bu) ppm; ^13^C {1H} NMR (100 MHz, CDCl_3_) *δ* 196.0, 176.5 (d, ^3^*J*_PC_ = 3.3 Hz), 165.5, 140.8, 137.5, 137.1, 133.0, 132.7 (d, ^1^*J*_PC_ = 103.7 Hz), 132.5, 132.4, 132.4, 132.3, 131.7, 131.6, 131.5 (d, ^1^*J*_PC_ = 105.4 Hz), 131.1, 131.0, 130.2, 129.9, 129.1, 129.0, 128.8, 128.7, 128.6, 128.3, 52.3, 50.1 (d, ^3^*J*_PC_ = 2.8 Hz), 42.7 (d, ^1^*J*_PC_ = 100.2 Hz), 28.2, 15.6 ppm; ^31^P NMR (160 MHz, CDCl_3_) *δ* 25.7 ppm; ESI-HRMS (CI) *m*/*z* calculated for C_34_H_34_N_2_O_4_P ([M + H]^+^), 565.2256; found 565.2252.

(2S*,3S*)-1-Acetyl-*N*-(*tert*-butyl)-3-(diphenylphosphoryl)-2-methylaziridine-2-carboxamide (**4o**) (89 mg, 45%) was obtained as a white solid from carboxylic acid **2k** (38 µL, 0.65 mmol), isocyanide **3b** (76 µL, 0.65 mmol) and 2*H*-azirine **1a** (127 mg, 0.50 mmol), as described in the general procedure. The crude product was purified via flash column chromatography (SiO_2_, AcOEt/hexane 50:50) and recrystallized from diethyl ether to give the title compound **4o**. mp 195–197 °C; IR (neat) *v_max_* 3358, 3056, 2984, 1699, 1665, 1540, 1274, 1116 cm^–1^; ^1^H NMR (400 MHz, CDCl_3_) *δ* 8.06–7.76 (m, 4H, ArH), 7.69–7.35 (m, 6H, ArH), 5.90 (s, 1H, NH), 3.46 (d, ^2^*J*_PH_ = 24.8 Hz, 1H, CH-P), 1.94 (s, 3H, CH_3_), 1.76 (s, 3H, CH_3_), 1.33 (s, 9H, *^t^*Bu) ppm; ^13^C {1H} NMR (100 MHz, CDCl_3_) *δ* 179.8 (d, ^3^*J*_PC_ = 2.9 Hz), 166.5, 132.5 (d, ^1^*J*_PC_ = 103.5 Hz), 132.4, 132.3 131.7, 131.7 (d, ^1^*J*_PC_ = 104.3 Hz), 131.6, 131.1, 130.0, 129.1, 129.0, 128.8, 128.7, 52.4, 48.4 (d, ^2^*J*_PC_ = 3.3 Hz), 42.9 (d, ^1^*J*_PC_ = 100.4 Hz), 28.6, 24.3, 15.4 ppm; ^31^P NMR (160 MHz, CDCl_3_) *δ* 23.8 ppm; ESI-HRMS (CI) *m*/*z* calculated for C_22_H_28_N_2_O_3_P ([M + H]^+^), 399.1838; found 399.1837.

(2S*,3S*)-*N*-(*tert*-Butyl)-3-(diphenylphosphoryl)-2-methyl-1-(2,2,2-trifluoroacetyl)aziridine-2-carboxamide (**4p**) (61 mg, 27%,) was obtained as a white solid from carboxylic acid **2l** (50 µL, 0.65 mmol), isocyanide **3b** (76 µL, 0.65 mmol) and 2*H*-azirine **1a** (127 mg, 0.50 mmol), as described in the general procedure. The crude product was purified via flash column chromatography (SiO_2_, AcOEt/hexane 75:25) to give the title compound **4p**. mp 172–174 °C; IR (neat) *v_max_* 3346, 3059, 2921, 1732, 1664, 1538, 1399, 1204, 1138 cm^–1^; ^1^H NMR (400 MHz, CDCl_3_) *δ* 7.95–7.90 (m, 2H, ArH), 7.85–7.79 (m, 2H, ArH), 7.58–7.45 (m, 6H, ArH), 5.91 (s, 1H, NH), 3.42 (d, ^2^*J*_PH_ = 21.0 Hz, 1H, CH-P), 1.75 (s, 3H, CH_3_), 1.29 (s, 9H, *^t^*Bu) ppm; ^13^C {1H} NMR (100 MHz, CDCl_3_) *δ* 167.3 (qd, ^2^*J*_CF_ = 38.2 Hz, ^3^*J*_PC_ = 3.4 Hz), 166.1, 132.7, 132.7, 132.7, 132.6, 132.1 (d, ^1^*J*_PC_ = 104.7 Hz), 131.4, 130.9, 130.8, 129.9, 129.2, 129.1, 128.9, 128.8, 115.37 (q, ^1^*J*_CF_ = 287.7 Hz), 55.9, 52.6 (d, ^2^*J*_PC_ = 3.5 Hz, C_quat_), 41.8 (d, ^1^*J*_PC_ = 98.3 Hz), 28.3, 14.8 ppm; ^19^F NMR (376 MHz, CDCl_3_) *δ* –75.5 ppm; ^31^P NMR (160 MHz, CDCl_3_) *δ* 23.4 ppm; ESI-HRMS (CI) *m*/*z* calculated for C_22_H_25_F_3_N_2_O_3_P ([M + H]^+^), 453.1555; found 453.1543.

(2S*,3S*)-*N*-(*tert*-Butyl)-3-(diphenylphosphoryl)-2-methyl-1-(2-phenylacetyl)aziridine-2-carboxamide (**4q**) (158 mg, 71%) was obtained as a white solid from carboxylic acid **2m** (88 mg, 0.65 mmol), isocyanide **3b** (76 µL mg, 0.65 mmol) and 2*H*-azirine **1a** (127 mg, 0.50 mmol), as described in the general procedure. The crude product was purified via flash column chromatography (SiO_2_, AcOEt/hexane 50:50) to give the title compound **4q**. mp: 236–238 °C; IR (neat) *v_max_* 3286, 3056, 2956, 1693, 1657, 1549, 1293, 1188 cm^–1^; ^1^H NMR (400 MHz, CDCl_3_) *δ* 8.01 (m, 2H, ArH), 7.91–7.77 (m, 2H, ArH), 7.59–7.41 (m, 6H, ArH), 7.27–7.21 (m, 3H, ArH), 7.16 (d, ^3^*J*_HH_ = 7.5, 2H, ArH), 5.68 (s, 1H, NH), 3.71 (d, ^2^*J*_HH_ = 16.5 Hz, 1H, CH_2_), 3.61 (d, ^2^*J*_HH_ = 16.5 Hz, 1H, CH_2_), 3.41 (d, ^2^*J*_PH_ = 23.5 Hz, 1H, CH-P), 1.35 (s, 9H, *^t^*Bu), 1.32 (s, 3H, CH_3_) ppm; ^13^C {1H} NMR (100 MHz, CDCl_3_) *δ* 180.8 (d, ^3^*J*_PC_ = 2.7 Hz), 166.6, 134.2, 133.3, 132.8 (d, ^1^*J*_PC_ = 103.5 Hz), 132.2, 132.2, 132.0, 132.0, 131.6, 131.5, 131.3 (d, ^1^*J*_PC_ = 104.8 Hz), 130.9, 130.9, 130.1, 128.9, 128.8, 128.5, 128.4, 128.4, 126.9, 52.3, 49.3 (d, ^2^*J*_PC_ = 3.5 Hz, C_quat_), 44.4, 42.2 (d, ^1^*J*_PC_ = 101.4 Hz), 28.5, 15.0 ppm; ^31^P NMR (160 MHz, CDCl_3_) *δ* 23.9 ppm; ESI-HRMS (CI) *m*/*z* calculated for C_28_H_32_N_2_O_3_P ([M + H]^+^), 475.2151; found 475.21341.

(2S*,3S*)-1-(2-([1,1’-Biphenyl]-4-yl)acetyl)-*N*-(*tert*-butyl)-3-(diphenylphosphoryl)-2-methylaziridine-2-carboxamide (**4r**) (96 mg, 35%) was obtained as a white solid from carboxylic acid **2n** (137 mg, 0.65 mmol), isocyanide **3b** (76 µL, 0.65 mmol) and 2*H*-azirine **1a** (127 mg, 0.50 mmol), as described in the general procedure. The crude product was purified via flash column chromatography (SiO_2_, AcOEt/hexane 50:50) to give the title compound **4r**. mp 256–258 °C; IR (neat) *v_max_* 3363, 3056, 2977, 1698, 1675, 1533, 1197 cm^–1^; ^1^H NMR (400 MHz, CDCl_3_) *δ* 8.02–7.97 (m, 2H, ArH), 7.88–7.83 (m, 2H, ArH), 7.56–7.41 (m, 12H, ArH), 7.34 (t, ^3^*J*_HH_ = 7.3 Hz, 1H, ArH), 7.22 (d, ^3^*J*_HH_ = 7.9 Hz, 2H, ArH), 5.74 (s, 1H, NH), 3.71 (d, ^2^*J*_HH_ = 16.6 Hz, 1H, CH_2_) 3.64 (d, ^2^*J*_HH_ = 16.6 Hz, 1H, CH_2_), 3.41 (d, ^2^*J*_PC_ = 21.9 Hz, 1H, CH-P), 1.40 (s, 3H, CH_3_), 1.38 (s, 9H, *^t^*Bu) ppm; ^13^C {1H} NMR (100 MHz, CDCl_3_) *δ* 181.0 (d, ^3^*J*_PC_ = 2.9 Hz), 166.8, 141.0, 140.0, 133.4, 132.4, 132.4, 132.2, 132.2, 131.8, 131.7, 131.3 (d, ^1^*J*_PC_ = 104.9 Hz), 131.1, 131.0, 130.6, 129.0, 128.9, 128.9, 128.7, 128.6, 127.4, 127.2, 127.2, 52.5, 49.6 (d, ^2^*J*_PC_ = 3.6 Hz), 44.2, 42.3 (d, ^1^*J*_PC_ = 101.3 Hz), 28.7, 15.2 ppm; ^31^P NMR (160 MHz, CDCl_3_) *δ* 24.2 ppm; ESI-HRMS (CI) *m*/*z* calculated for C_34_H_36_N_2_O_3_P ([M + H]^+^), 551.2464; found 551.2459.

(2S*,3S*)-1-Benzoyl-*N*-cyclopropyl-3-(diphenylphosphoryl)-2-methylaziridine-2-carboxamide (**4s**) (133 mg, 60%) was obtained as a white solid from carboxylic acid **2a** (79 mg, 0.65 mmol), isocyanide **3c** (43 µL, 0.65 mmol) and 2*H*-azirine **1a** (127 mg, 0.50 mmol), as described in the general procedure. The crude product was purified via flash column chromatography (SiO_2_, AcOEt/hexane 20:80) to give the title compound **4s**. mp 203–205 °C; IR (neat) *v_max_* 3256, 3059, 2923, 1674, 1579, 1299, 1182 cm^–1^; ^1^H NMR (400 MHz, CDCl_3_) *δ* 8.04–7.88 (m, 4H, ArH), 7.81 (d, ^3^*J*_HH_ = 7.5 Hz, 2H, ArH), 7.64–7.44 (m, 7H, ArH), 7.38 (t, ^3^*J*_HH_ = 7.6 Hz, 2H, ArH), 6.30 (s, 1H, HC-NH) 3.80 (d, ^2^*J*_PC_ = 23.8 Hz, 1H, CH-P), 2.46–2.29 (m, 1H, HC-NH), 1.93 (s, 3H, CH_3_), 0.68–0.43 (m, 2H, CH_2_), 0.40–0.21 (m, 1H, CH_2_), 0.00 to –0.17 (m, 1H, CH_2_) ppm; ^13^C {1H} NMR (100 MHz, CDCl_3_) *δ* 176.7 (d, ^3^*J*_PC_ = 3.2 Hz), 168.0, 134.1, 132.6 (d, ^1^*J*_PC_ = 103.5 Hz), 132.6, 132.4, 132.4, 132.3, 132.3, 132.1, 131.7, 131.6, 131.1, 131.1, 129.1, 129.0, 128.8, 128.6, 128.5, 128.5, 49.5 (d, ^2^*J*_PC_ = 3.0 Hz, C_quat_), 42.4 (d, ^1^*J*_PC_ = 100.5 Hz), 23.1, 15.4, 6.5, 6.4 ppm; ^31^P NMR (160 MHz, CDCl_3_) *δ* 26.4 ppm; ESI-HRMS (CI) *m*/*z* calculated for C_26_H_26_N_2_O_3_P ([M + H]^+^), 445.1681; found 445.1682.

(2S*,3S*)-*N*-Cyclopropyl-3-(diphenylphosphoryl)-2-methyl-1-(quinoline-6-carbonyl)aziridine-2-carboxamide (**4t**) (136 mg, 55%) obtained as a white solid from carboxylic acid **2j** (112 mg, 0.65 mmol), isocyanide **3c** (43 µL, 0.65 mmol) and 2*H*-azirine **1a** (127 mg, 0.50 mmol), as described in the general procedure. The crude product was purified via flash column chromatography (SiO_2_, AcOEt/hexane 20:80) to give the title compound **4t**. mp 212–214 °C; IR (neat) *v_max_* 3195, 3045, 2953, 1674, 1660, 1551, 1290, 1177 cm^–1^; ^1^H NMR (400 MHz, CDCl_3_) *δ* 8.98 (d, ^4^*J*_HH_ = 3.0 Hz, 1H, ArH), 8.37 (s, 1H, ArH), 8.16 (d, ^3^*J*_HH_ = 9.5 Hz, 1H, ArH), 8.08–8.03 (m, 2H, ArH), 7.98–7.86 (m, 4H, ArH), 7.60–7.48 (m, 4H, ArH), 7.46–7.41 (m, 3H, ArH), 6.20 (d, ^2^*J*_HH_ = 22.1 Hz, 1H, HC-NH), 3.78 (d, ^2^*J*_PC_ = 23.7 Hz, 1H, CH-P), 2.41–2.34 (m, 1H, HC-NH), 0.62–0.44 (m, 2H, CH_2_), 0.29–0.23 (m, 1H, CH_2_), 0.04 to –0.10 (m, 1H, CH_2_); ^13^C {1H} NMR (100 MHz, CDCl_3_) *δ* 176.1 (d, ^3^*J*_PC_ = 3.1 Hz), 168.3, 152.5, 149.9, 137.5, 132.5 (d, ^1^*J*_PC_ = 103.5 Hz), 132.5, 132.5, 132.4, 132.4, 132.2, 131.5 (d, ^1^*J*_PC_ = 105.3 Hz), 129.9, 129.7, 129.2, 129.0, 128.8, 128.7, 128.1, 127.6, 122.0, 49.7 (d, ^3^*J*_PC_ = 2.8 Hz, C_quat_), 42.5 (d, ^1^*J*_PC_ = 99.9 Hz), 23.2, 15.3, 6.6, 6.6 ppm; ^31^P NMR (160 MHz, CDCl_3_) *δ* 23.8 ppm; ESI-HRMS (CI) *m*/*z* calculated for C_29_H_27_N_3_O_3_P ([M + H]^+^), 496.1790; found 496.1788.

(2S*,3S*)-*N*-Cyclopropyl-3-(diphenylphosphoryl)-1-(furan-2-carbonyl)-2-methylaziridine-2-carboxamide (**4u**) (162 mg, 75%) was obtained as a white solid from carboxylic acid **2h** (73 mg, 0.65 mmol), isocyanide **3c** (43 µL, 0.65 mmol) and 2*H*-azirine **1a** (127 mg, 0.50 mmol), as described in the general procedure. The crude product was purified via flash column chromatography (SiO_2_, AcOEt/hexane 80:20) to give the title compound **4u**. mp 127–129 °C; IR (neat) *v_max_* 3236, 3114, 2961, 1674, 1579, 1290, 1171 cm^–1^; ^1^H NMR (400 MHz, CDCl_3_) *δ* 7.98–7.91 (m, 4H, ArH), 7.59–7.46 (m, 6H, ArH), 7.27 (d, ^4^*J*_HH_ = 1.0 Hz, 1H, ArH), 7.15 (dd, ^3^*J*_HH_ = 3.5 Hz, ^4^*J*_HH_ = 0.8 Hz, 1H, ArH), 6.46 (dd, ^3^*J*_HH_ = 3.6 Hz, ^3^*J*_HH_ = 1.7 Hz, 1H, ArH), 6.24 (s, 1H, HC-NH), 3.70 (d, ^2^*J*_PH_ = 23.6 Hz, 1H, CH-P), 2.60–2.54 (m, 1H, HC-NH), 1.90 (s, 3H, CH_3_), 0.76–0.64 (m, 2H, CH_2_), 0.49–0.42 (m, 1H, CH_2_), 0.32–0.26 (m, 1H, CH_2_) ppm; ^13^C {1H} NMR (100 MHz, CDCl_3_) *δ* 168.6, 165.8 (d, ^3^*J*_PC_ = 3.4 Hz), 148.3, 145.0, 132.5 (d, ^1^*J*_PC_ = 103.5 Hz), 132.3, 132.3, 132.2, 132.1, 131.7, 131.6, 131.0, 130.9, 129.0, 128.8, 128.6, 128.5, 116.7, 112.3, 48.9, 42.5 (d, ^1^*J*_PC_ = 99.9 Hz), 29.7, 23.2, 15.1, 6.6, 6.6 ppm; ^31^P NMR (160 MHz, CDCl_3_) *δ* 24.6 ppm; ESI-HRMS (CI) *m*/*z* calculated for C_24_H_24_N_2_O_4_P ([M + H]^+^), 435.1474; found 434.1463.

### 3.3. Experimental Procedure and Characterization Data for N-Acylaziridine Phosphonates ***5***

In a flame-dried flask, the corresponding carboxylic acid **2** (0.65 mmol, 1.3 eq.), isocyanide **3** (0.65 mmol, 1.3 eq.) and a 1M diethyl ether solution of ZnCl_2_ (0.12 mL, 0.12 mmol, 0.25 eq.) were added to 0.5 mL of dry THF. Then, 2*H*-azirine phosphonate **1b** (0.50 mmol, 1 eq.) was added at room temperature. The reaction mixture was stirred until TLC showed the disappearance of 2*H*-azirine **1b** (1–24 h). The solvent was removed under vacuum, and the residue was dissolved in dichloromethane (5 mL) and washed with water (2 × 5 mL). The organic layer was dried over anhydrous MgSO_4_ and filtered, and the solvent was evaporated under reduced pressure. The resulting residue was purified via flash column chromatography (SiO_2_, hexanes/AcOEt) to yield compounds **5**.

Diethyl ((2S*,3S*)-1-benzoyl-3-(*tert*-butylcarbamoyl)-3-methylaziridin-2-yl)phosphonate (**5a**) (118 mg, 60%) was obtained as a white solid from carboxylic acid **2a** (79 mg, 0.65 mmol), isocyanide **3b** (76 µL, 0.65 mmol) and 2*H*-azirine **1b** (96 mg, 0.50 mmol), as described in the general procedure. The crude product was purified via flash column chromatography (SiO_2_, AcOEt/hexane 45:55) to give the title compound **5a**. mp 140–141 °C; IR (neat) *v_max_* 3306, 3071, 2966, 1688, 1665, 1633, 1236 cm^–1^; ^1^H NMR (400 MHz, CDCl_3_) *δ* 7.83 (d, ^3^*J*_HH_ = 7.3 Hz, 2H, ArH), 7.48 (d, ^3^*J*_HH_ = 7.3 Hz, 1H, ArH), 7.39 (t, ^3^*J*_HH_ = 7.5 Hz, 2H, ArH), 5.75 (s, 1H, NH), 4.33–4.10 (m, 4H, OCH_2_CH_3_), 3.25 (d, ^2^*J*_PC_ = 17.2 Hz, 1H, CH-P), 1.94 (s, 3H, CH_3_), 1.39–1.35 (m, 6H, OCH_2_CH_3_), 1.01 (s, 9H, *^t^*Bu) ppm; ^13^C {1H} NMR (100 MHz, CDCl_3_) *δ* 176.3 (d, ^3^*J*_PC_ = 4.7 Hz), 165.2, 134.1, 132.5, 128.5, 128.4, 63.6 (d, ^2^*J*_PC_ = 6.9 Hz), 62.6 (d, ^2^*J*_PC_ = 6.9 Hz), 52.1, 48.4 (d, ^2^*J*_PC_ = 2.8 Hz), 38.8 (d, ^1^*J*_PC_ = 202.0 Hz), 28.1, 16.5 (d, ^3^*J*_PC_ = 6.5 Hz), 16.4 (t, ^3^*J*_PC_ = 6.5 Hz), 15.4 ppm; ^31^P NMR (160 MHz, CDCl_3_) *δ* 17.3 ppm; ESI-HRMS (CI *m*/*z* calcd. For C_19_H_30_N_2_O_5_P ([M + H]^+^), 397.1892; found 397.1891.

Diethyl ((2S*,3S*)-3-(*tert*-butylcarbamoyl)-1-(4-fluorobenzoyl)-3-methylaziridin-2-yl)phosphonate (**5b**) (158 mg, 76%) was obtained as a needle-shaped crystal from carboxylic acid **2d** (91 mg, 0.65 mmol), isocyanide **3b** (76 µL, 0.65 mmol) and 2*H*-azirine **1b** (96 mg, 0.50 mmol), as described in the general procedure. The crude product was purified via flash column chromatography (SiO_2_, AcOEt/hexane 50:50) to give the title compound **5b**. mp 99–101 °C; IR (neat) *v_max_* 3391, 2982, 1686, 1602, 1525, 1288, 1240 cm^–1^; ^1^H NMR (400 MHz, CDCl_3_) *δ* 7.85 (dd, ^2^*J*_HH_ = 8.8 Hz, ^3^*J*_HF_ = 5.4 Hz, 2H, ArH), 7.08 (t, ^2^*J*_HF_ = 8.6 Hz, ^2^*J*_HH_ = 8.6 Hz, 2H, ArH), 5.76 (s, 1H, NH), 4.42–4.05 (m, 4H, OCH_2_CH_3_), 3.24 (d, ^2^*J*_PC_ = 17.1 Hz, 1H, CH-P), 1.94 (s, 3H, CH_3_), 1.55–1.20 (m, 6H, OCH_2_CH_3_), 1.06 (s, 9H, *^t^*Bu) ppm; ^13^C {1H} NMR (100 MHz, CDCl_3_) *δ* 175.2 (d, ^3^*J*_PC_ = 4.7 Hz), 165.4 (d, ^1^*J*_CF_ = 253.4 Hz), 165.2, 130.9 (d, ^3^*J*_CF_ = 9.1 Hz, 130.5 (d, ^4^*J*_CF_ = 3.0 Hz), 115.5 (d, ^2^*J*_CF_ = 21.8 Hz), 63.6 (d, ^2^*J*_PC_ = 6.1 Hz), 62.7 (d, ^2^*J*_PC_ = 6.6 Hz), 52.2, 48.4 (d, ^2^*J*_PC_ = 2.8 Hz), 38.7 (d, ^1^*J*_PC_ = 202.4 Hz), 28.2, 16.5 (d, ^2^*J*_PC_ = 6.6 Hz), 16.5 (d, ^2^*J*_PC_ = 6.6 Hz) 15.4 ppm; ^31^P NMR (160 MHz, CDCl_3_) *δ* 17.1 ppm; ^19^F NMR (376 CDCl_3_) *δ* –106.4 ppm; ESI-HRMS (CI) *m*/*z* calcd. For C_19_H_29_FN_2_O_5_P ([M + H]^+^), 415.1798; found 415.1806.

Diethyl ((2S*,3S*)-3-(*tert*-butylcarbamoyl)-1-(furan-2-carbonyl)-3-methylaziridin-2-yl)phosphonate (**5c**) (135 mg, 70%) was obtained as a white solid from carboxylic acid **2h** (73 mg, 0.65 mmol), isocyanide **3b** (76 µL, 0.65 mmol) and 2*H*-azirine **1b** (96 mg, 0.50 mmol), as described in the general procedure. The crude product was purified via flash column chromatography (SiO_2_, AcOEt/hexane 50:50) to give the title compound **5c**. mp 94–96 °C; IR (neat) *v_max_* 3246, 3062, 2986, 1701, 1679, 1652, 1448, 1188, 1121 cm^–1^; ^1^H NMR (400 MHz, CDCl_3_) *δ* 7.44–7.42 (m, 1H, ArH), 7.13–7.11 (m, 1H, ArH), 6.59–6.11 (m, 1H, ArH), 5.97 (s, 1H, NH), 4.40–3.69 (m, 4H, OCH_2_CH_3_), 3.12 (d, ^2^*J*_PC_ = 18.2 Hz, 1H, CH-P), 1.89 (s, 3H, CH_3_), 1.35 (t, ^3^*J*_HH_ = 7.1 Hz, 6H, OCH_2_CH_3_), 1.15 (s, 9H, *^t^*Bu) ppm; ^13^C {1H} NMR (100 MHz, CDCl_3_) *δ* 165.7 (d, ^3^*J*_PC_ = 5.1 Hz), 165.7 (d, ^3^*J*_PC_ = 1.1 Hz), 148.4, 145.2, 116.5, 112.2, 63.8 (d, ^2^*J*_PC_ = 6.4 Hz), 63.0 (d, ^2^*J*_PC_ = 6.5 Hz), 52.1, 48.5 (d, ^2^*J*_PC_ = 3.0 Hz), 39.2 (d, ^1^*J*_PC_ = 203.7 Hz), 28.2, 16.3 (d, ^3^*J*_PC_ = 5.0 Hz), 16.2 (d, ^3^*J*_PC_ = 5.1 Hz), 15.3 ppm; ^31^P NMR (160 MHz, CDCl_3_) *δ* 16.9 ppm; ESI-HRMS (CI) *m*/*z* calcd. for C_17_H_28_N_2_O_6_P ([M + H]^+^), 387.1685; found 387.1668.

Diethyl ((2S*,3S*)-3-(*tert*-butylcarbamoyl)-3-methyl-1-(quinoline-6-carbonyl)aziridin-2-yl)phosphonate (**5d**) (123 mg, 55%) was obtained as a white solid from carboxylic acid **2j** (112 mg, 0.65 mmol), isocyanide **3b** (76 µL, 0.65 mmol) and 2*H*-azirine **1b** (96 mg, 0.50 mmol), as described in the general procedure. The crude product was purified via flash column chromatography (SiO_2_, CH_2_Cl_2_/MeOH 99:1) to give the title compound **5d**. mp 152–153 °C; IR (neat) *v_max_* 3412, 2922, 1676, 1665, 1528, 1287, 1157 cm^–1^; ^1^H NMR (400 MHz, CDCl_3_) *δ* 8.99 (dd, ^3^*J*_HH_ = 4.2 Hz, ^4^*J*_HH_ = 1.7 Hz, 1H, ArH), 8.42 (dd, ^4^*J*_HH_ = 1.8 Hz, ^4^*J*_HH_ = 0.9 Hz, 1H), 8.26–8.23 (m, 1H, ArH), 8.13–8.08 (m, 2H, ArH), 7.46 (ddd, ^3^*J*_HH_ = 8.3 Hz, ^3^*J*_HH_ = 4.2 Hz, ^4^*J*_HH_ = 1.0 Hz, 1H, ArH), 5.76 (s, 1H, NH), 4.30–4.23 (m, 4H, OCH_2_CH_3_), 3.32 (d, ^2^*J*_PC_ = 17.0 Hz, 1H, CH-P), 2.02 (s, 3H, CH_3_), 1.42–1.38 (m, 6H, OCH_2_CH_3_), 0.95 (s, 9H, *^t^*Bu) ppm; ^13^C {1H} NMR (100 MHz, CDCl_3_) *δ* 175.9 (d, ^3^*J*_PC_ = 4.7 Hz), 165.3, 152.5, 149.9, 137.4, 132.1, 129.9, 129.7, 128.1, 127.6, 122.0, 63.7 (d, ^2^*J*_PC_ = 6.2 Hz), 62.7 (d, ^2^*J*_PC_ = 6.6 Hz), 52.2, 48.6 (d, ^2^*J*_PC_ = 2.8 Hz), 39.2 (d, ^1^*J*_PC_ = 201.9 Hz), 28.2, 16.6, (d, ^3^*J*_PC_ = 6.6 Hz), 16.5, (d, ^3^*J*_PC_ = 6.9 Hz), 15.5 ppm; ^31^P NMR (160 MHz, CDCl_3_) *δ* 17.0 ppm; ESI-HRMS (CI) *m*/*z* calcd. for C_22_H_31_N_3_O_5_P ([M + H]^+^), 448.2001; found 448.1994.

Diethyl ((2S*,3S*)-3-(*tert*-butylcarbamoyl)-3-methyl-1-(2-phenylacetyl)aziridin-2-yl)phosphonate (**5e**) (71 mg, 35%) was obtained as a white crystalline solid from carboxylic acid **2m** (88 mg, 0.65 mmol), isocyanide **3b** (76 µL, 0.65 mmol) and 2*H*-azirine **1b** (96 mg, 0.50 mmol), as described in the general procedure. The crude product was purified via flash column chromatography (SiO_2_, AcOEt/hexane 50:50) to give the title compound **5e**. mp 124–126 °C; IR (neat) *v_max_* 3312, 3061, 2985, 1688, 1669, 1565, 1236, 1028 cm^–1^; ^1^H NMR (400 MHz, CDCl_3_) *δ* 7.43–6.85 (m, 5H, ArH), 5.76 (s, 1H, NH), 4.37–4.09 (m, 4H, OCH_2_CH_3_), 3.73 (d, ^2^*J*_HH_ = 16.3, 1H, CH_2_), 3.66 (d, ^2^*J*_HH_ = 16.4 Hz, 1H, CH_2_), 2.89 (d, ^2^*J*_PC_ = 18.3 Hz, 1H, CH-P), 1.49 (s, 3H, CH_3_), 1.42–1.22 (m, 15H), 1.32 (s, 9H, *^t^*Bu) ppm; ^13^C {1H} NMR (100 MHz, CDCl_3_) *δ* 180.1 (d, ^3^*J*_PC_ = 4.2 Hz), 166.6, 134.3, 130.2, 128.6, 127.1, 63.5 (d, ^2^ *J*_PC_ = 6.3 Hz), 62.7 (d, ^2^*J*_PC_ = 6.5 Hz), 52.3, 48.0 (d, ^2^*J*_PC_ = 3.3 Hz), 44.4, 38.8 (d, ^1^*J*_PC_ = 201.5 Hz), 28.6, 16.3 (d, ^3^*J*_PC_ = 6.3 Hz), 16.3 (d, ^3^*J*_PC_ = 7.8, Hz), 14.8 (CH_3_) ppm; ^31^P NMR (160 MHz, CDCl_3_) *δ* 16.7 ppm; ESI-HRMS (CI) *m*/*z* calcd. for C_20_H_32_N_2_O_5_P^+^ ([M + H]^+^), 411.2049; found 411.2046.

Diethyl ((2S*,3S*)-1-(2-([1,1’-biphenyl]-4-yl)acetyl)-3-(*tert*-butylcarbamoyl)-3-methylaziridin-2-yl)phosphonate (**5f**) (126 mg, 52%) was obtained as a white solid from carboxylic acid **2n** (137 mg, 0.65 mmol), isocyanide **3b** (76 µL, 0.65 mmol) and 2*H*-azirine **1b** (96 mg, 0.50 mmol), as described in the general procedure. The crude product was purified via flash column chromatography (SiO_2_, AcOEt/hexane 50:50) to give the title compound **5f**. mp 150–152 °C; IR (neat) *v_max_* 3361, 3056, 2975, 1699, 1654, 1526, 1252 cm^–1^; ^1^H NMR (400 MHz, CDCl_3_) *δ* 7.56 (dd, ^3^*J*_HH_ = 10.7 Hz, ^3^*J*_HH_ = 7.8 Hz, 4H, ArH), 7.43 (t, ^3^*J*_HH_ = 7.6 Hz, 2H, ArH), 7.34 (d, ^3^*J*_HH_ = 8.1 Hz, 3H, ArH), 5.77 (s, 1H, NH), 4.25–4.15 (m, 4H, OCH_2_CH_3_), 3.75 (d, ^2^*J*_HH_ = 16.3 Hz, 1H, CH_2_), 3.69 (d, ^2^*J*_HH_ = 16.3 Hz, 1H, CH_2_), 2.91 (d, ^2^*J*_PC_ = 18.2 Hz, 1H, CH-P), 1.55 (s, 3H, CH_3_), 1.37–1.33 (m, 6H, OCH_2_CH_3_) 1.36 (s, 9H, *^t^*Bu) ppm; ^13^C {1H} NMR (100 MHz, CDCl_3_) *δ* 180.1 (d, ^3^*J*_PC_ = 4.3 Hz), 166.6, 140.9, 140.1, 133.4, 130.6, 128.9, 127.4, 127.2, 127.0, 63.5 (d, ^2^*J*_PC_ = 6.3 Hz), 62.7 (d, ^2^*J*_PC_ = 6.5 Hz), 52.4, 47.9 (d, ^2^*J*_PC_ = 3.3 Hz), 44.0, 38.9 (d, ^1^*J*_PC_ = 201.3 Hz), 28.7, 16.5 (d, ^3^*J*_PC_ = 6.2 Hz), 16.5 (d, ^3^*J*_PC_ = 6.3 Hz), 14.9 (CH_3_) ppm; ^31^P NMR (160 MHz, CDCl_3_) *δ* 16.6 ppm; ESI-HRMS (CI) *m*/*z* calcd. for C_26_H_36_N_2_O_5_P ([M + H]^+^), 487.2362; found 487.2340.

Diethyl ((2S*,3S*)-3-(cyclopropylcarbamoyl)-3-methyl-1-(3-phenylpropioloyl)aziridin-2-yl)phosphonate (**5g**) (96 mg, 48%) was obtained as a white wax from carboxylic acid **2o** (95 mg, 0.65 mmol), isocyanide **3b** (76 µL, 0.65 mmol) and 2*H*-azirine **1b** (96 mg, 0.50 mmol), as described in the general procedure. The crude product was purified via flash column chromatography (SiO_2_, AcOEt/hexane 60:40) to give the title compound **5g**. mp 133–135 °C; IR (neat) *v_max_* 3332, 2987, 2204, 1681, 1672, 1538, 1286, 1187, 1016 cm^–1^; ^1^H NMR (400 MHz, CDCl_3_) *δ* 7.55–7.53 (m, 2H, ArH), 7.45–7.41 (m, 1H, ArH), 7.38–7.33 (m, 2H, ArH), 6.56 (d, ^3^*J*_HH_ = 2.9 Hz, 1H, HC-NH), 4.29–4.17 (m, 4H, OCH_2_CH_3_), 3.26 (d, ^2^*J*_PC_ = 17.1 Hz, 1H, CH-P), 2.75–2.69 (m, 1H, HC-NH), 1.86 (s, 3H, CH_3_), 1.36–1.32 (m, 6H, OCH_2_CH_3_), 0.79–0.74 (m, 2H, CH_2_), 0.58–0.54 (m, 2H, CH_2_) ppm; ^13^C {1H} NMR (100 MHz, CDCl_3_) *δ* 167.9, 161.0 (d, ^3^*J*_PC_ = 5.6 Hz), 133.0, 130.8, 128.7, 119.9, 89.3, 83.1, 63.6 (d, ^2^ *J*_PC_ = 6.3 Hz), 62.8 (d, ^2^*J*_PC_ = 6.5 Hz), 48.1 (^2^*J*_PC_ = 2.04 Hz) 40.8 (d, ^1^*J*_PC_ = 200.6 Hz), 23.6, 16.5 (d, ^3^*J*_PC_ = 6.1 Hz), 14.6, 6.7, 6.6 ppm; ^31^P NMR (160 MHz, CDCl_3_) *δ* 15.9 ppm; ESI-HRMS (CI) *m*/*z* calcd. for C_20_H_26_N_2_O_5_P ([M + H]^+^), 405.1579; found 405.1570.

Diethyl ((2S*,3S*)-1-((*E*)-but-2-enoyl)-3-(*tert*-butylcarbamoyl)-3-methylaziridin-2-yl)phosphonate (**5h**) (96 mg, 48%) was obtained as an oil from carboxylic acid **2q** (56 mg, 0.65 mmol), isocyanide **3b** (76 µL, 0.65 mmol) and 2*H*-azirine **1b** (96 mg, 0.50 mmol), as described in the general procedure. The crude product was purified via flash column chromatography (SiO_2_, AcOEt/hexane 50:50) to give the title compound **5h**. R_f_: 0.50 (AcOEt); IR (neat) *v_max_* 3362, 2977, 1689, 1645, 1523, 1288, 1244, 1192, 1027 cm^–1^; ^1^H NMR (400 MHz, CDCl_3_) *δ* 6.90 (dq, ^3^*J*_HH_ = 15.5 Hz, ^3^*J*_HH_ = 6.9 Hz, 1H, CH_3_CH=CH) 6.00 (s, 1H, NH), 5.95 (dd, ^3^*J*_HH_ = 15.5 Hz, ^4^*J*_HH_ = 1.7 Hz, 1H, CH_3_CH=CH) 4.38–4.14 (m, 4H, OCH_2_CH_3_), 3.03 (d, ^2^*J*_PC_ = 17.1 Hz, 1H, CH-P), 1.88 (dd, ^3^*J*_HH_ = 6.9 Hz, ^4^*J*_HH_ = 1.7 Hz, 3H, CH_3_-CH=), 1.89 (s, 3H, CH_3_), 1.43–1.33 (m, 6H, OCH_2_CH_3_), 1.34 (s, 9H, *^t^*Bu) ppm; ^13^C {1H} NMR (100 MHz, CDCl_3_) *δ* 173.6 (d, ^3^*J*_PC_ = 4.8 Hz), 165.9, 143.4, 125.4, 63.3 (d, ^2^*J*_PC_ = 6.3 Hz), 62.6 (d, ^2^*J*_PC_ = 6.5 Hz), 52.1, 47.3 (d, ^2^*J*_PC_ = 2.8 Hz), 38.7 (d, ^1^*J*_PC_ = 200.8 Hz), 28.5, 18.1, 16.4 (d, ^3^*J*_PC_ = 6.0 Hz), 16.4 (d, ^3^*J*_PC_ = 6.2, Hz), 14.9 ppm; ^31^P NMR (160 MHz, CDCl_3_) *δ* 17.1 ppm; ESI-HRMS (CI) *m*/*z* calcd. for C_16_H_30_N_2_O_5_P ([M + H]^+^), 361.1892; found 361.1878.

### 3.4. Gram Scale Procedure of N-Acylaziridine ***5a***

In a flame-dried flask, benzoic acid **2a** (635 mg, 5.2 mmol, 1.3 eq.), *tert*-butyl isocyanide **3b** (588 µL, 5.2 mmol, 1.3 eq.) and a 1M diethyl ether solution of ZnCl_2_ (1.0 mL, 1 mmol, 0.25 eq.) were added to 4 mL of dry THF. Then, 2*H*-azirine phosphonate **1b** (764 mg, 4 mmol, 1 eq.) was added at room temperature. The reaction mixture was stirred until TLC showed the disappearance of 2*H*-azirine **1b** (4 h). The solvent was removed under vacuum, and the residue was dissolved in dichloromethane (15 mL) and washed with saturated NaHCO_3_ solution (15 mL) and water (2 × 15 mL). The organic layer was dried over anhydrous MgSO_4_ and filtered, and the solvent was evaporated under reduced pressure. The resulting residue was recrystallized from pentane to yield the desired *N*-acylaziridine **5a** (1.11 g, 70%).

### 3.5. Experimental Procedure and Characterization Data for Phosphorylated Aziridine Peptidomimetics ***6***

In a flame-dried flask, corresponding amino acid **2r** or **2s** (1.3 mmol, 1.3 eq.), *tert-*butyl isocyanide **3b** (1.3 mmol, 1.3 eq.) and a 1M diethyl ether solution of ZnCl_2_ (0.25 mL, 0.25 mmol, 0.25 eq.) were added to 1 mL of dry THF. Then, 2*H*-azirine phosphonate **1b** (1 mmol, 1 eq.) was added at room temperature. The reaction mixture was stirred until TLC showed the disappearance of 2*H*-azirine **1b** (6 h). The solvent was removed under vacuum, and the residue was dissolved in dichloromethane (5 mL) and washed with water (2 × 5 mL). The organic layer was dried over anhydrous MgSO_4_ and filtered, and the solvent was evaporated under reduced pressure. The resulting residue was purified via flash column chromatography (SiO_2_, hexanes/AcOEt) to yield the title compounds.

(9*H*-Fluoren-9-yl)methyl ((*S*)-1-((2*S*,3*S*)-2-(*tert*-butylcarbamoyl)-3-(diethoxyphosphoryl)-2-methylaziridin-1-yl)-4-methyl-1-oxopentan-2-yl)carbamate and (9*H*-fluoren-9-yl)methyl ((*S*)-1-((2*R*,3*R*)-2-(*tert*-butylcarbamoyl)-3-(diethoxyphosphoryl)-2-methylaziridin-1-yl)-4-methyl-1-oxopentan-2-yl)carbamate (**6a**) (407 mg, 65%) yielded (50/50) of a mixture of two diastereoisomers, **6a**_A_ and **6a**_B_, obtained as oils from amino acid **2r** (459 mg, 1.3 mmol), *tert-*butyl isocyanide **3b** (152 µL, 1.3 mmol) and 2*H*-azirine **1b** (191 mg, 1 mmol), as described in the general procedure. The crude product was purified via flash column chromatography (SiO_2_, AcOEt/hexane 40:60) to give the title compound as a mixture of two diastereoisomers (**6a**_A_ and **6a**_B_). R_f_: 0.60 (AcOEt); IR (neat) *v_max_* 3444, 3328, 3067, 2961, 1701, 1668, 1521, 1257, 1218, 1016 cm^–1^; ^1^H NMR (400 MHz, CDCl_3_) *δ* 7.75 (d, ^3^*J*_HH_ = 7.5 Hz, 4H, ArH)_A+B_, 7.60–7.58 (m, 1H, 4H, ArH)_A+B_, 7.39 (t, ^3^*J*_HH_ = 7.4 Hz, 4H, ArH)_A+B_, 7.32–7.28 (m, ^3^*J*_HH_ = 7.4 Hz, 4H, ArH)_A+B_, 5.99 (s, 1H, NH)_A_, 5.89 (s, 1H, NH)_B_, 5.28 (d, ^3^*J*_HH_ = 8.7 Hz, NH_Fmoc_)_A_, 5.11 (d, ^3^*J*_HH_ = 9.2 Hz, NH_Fmoc_)_B_, 4.49–4.18 (m, 16H, CH_α_ + OCH_2_CH_3_ + CH_2Fmoc_ + CH_Fmoc_)_A+B_, 2.99 (d, ^2^*J*_PH_ = 18.1 Hz, 1H, CH-P)_B_, 2.85 (d, ^2^*J*_PH_ = 17.7 Hz, 1H, CH-P)_A_, 1.80–1.57 (m, 12H, CH_3_ + CHCH_2_)_A+B_, 1.38–1.29 (m, 30H, OCH_2_CH_3 +_ *^t^*Bu)_A+B_, 0.97–0.93 (m, 12H, CH_3_)_A+B_ ppm; ^13^C {1H} NMR (100 MHz, CDCl_3_) *δ* 181.8 (d, ^3^*J*_PC_ = 4.3 Hz)_A_, 181.7 (d, ^3^*J*_PC_ = 4.3 Hz,)_B_, 166.7_A_, 166.0_B_, 155.8_A_, 155.7_B_, 143.9_B_, 143.8_B_, 143.8_A_, 141.4_B_, 141.3_A_, 141.3_B_, 141.3_A_, 127.8_B_, 127.7_A_, 127.7_B_, 127.6_A_, 127.1_A_, 127.1_A_, 127.1_B_, 127.0_B_, 125.1_A_, 125.0_B_, 124.9_B_, 120.1_B_, 120.0_B_, 119.9_A_, 66.8_B_, 66.6A, 63.5 (d, *^2^J*_PC_ = 6.6 Hz)_A_, 63.3 (d, ^2^*J*_PC_ = 6.3 Hz)_B_, 62.7 (d, ^2^*J*_PC_ = 6.4 Hz)_A_, 62.7 (d, ^2^*J*_PC_ = 6.0 Hz)_B_, 54.9_A_, 54.2_B_, 52.4_A_, 52.3_B_, 48.8 (d, ^2^*J*_PC_ = 3.3 Hz)_A_, 48.4 (d, ^2^*J*_PC_ = 3.2 Hz)_B_, 47.2, 47.2, 42.4_A_, 42.3_B_, 38.62 (d, ^1^*J_PC_* = 201.8 Hz)_A_, 37.83 (d, ^1^*J_PC_* = 201.1, Hz)_B_, 28.5_B_, 28.4_A_, 24.6_A_, 24.5_B_, 23.4_B_, 23.2_A_, 21.8_A_, 21.6_B_, 16.4_A+B_, 15.2_A_, 15.1_B_ ppm; ^31^P NMR (160 MHz, CDCl_3_) *δ* **6a**_B_ 16.9, **6a**_A_ 16.2 ppm; ESI-HRMS (CI) *m*/*z* calcd. for C_33_H_47_N_3_O_7_P ([M + H]^+^), 628.3152; found 628.3143.

(9*H*-Fluoren-9-yl)methyl ((*S*)-1-((2*S*,3*S*)-2-(*tert*-butylcarbamoyl)-3-(diethoxyphosphoryl)-2-methylaziridin-1-yl)-1-oxopropan-2-yl)carbamate and (9*H*-fluoren-9-yl)methyl ((*S*)-1-((2*R*,3*R*)-2-(*tert*-butylcarbamoyl)-3-(diethoxyphosphoryl)-2-methylaziridin-1-yl)-1-oxopropan-2-yl)carbamate (**6b**) (114 mg, 20%) yielded (50/50) of a mixture of two diastereoisomers, **6b**_A_ and **6b**_B_, obtained as oils from amino acid **2s** (405 mg, 1.3 mmol), *tert-*butyl isocyanide **3b** (152 µL, 1.3 mmol) and 2*H*-azirine **1b** (191 mg, 1 mmol), as described in the general procedure. The crude product was purified via flash column chromatography (SiO_2_, AcOEt/hexane 40:60) to give the title compound as a mixture of two diastereoisomers (**6b**_A_ and **6b**_B_). R_f_: 0.60 (AcOEt); IR (neat) *v_max_* 3450, 3278, 3075, 2925, 1682, 1651, 1288, 1196 cm^–1^;^1^H NMR (400 MHz, CDCl_3_) *δ* 7.76–7.74 (m, 4H, ArH), 7.59–7.57 (m, 4H, ArH), 7.41–7.37 (m, 4H, ArH), 7.33–7.27 (m, 4H, ArH), 6.01 (s, 1H, NH), 5.90 (s, 1H, NH), 5.47 (d, ^3^*J*_HH_ = 7.7 Hz, 1H, NH_Fmoc_), 5.33 (d, ^3^*J*_HH_ = 7.8 Hz, 1H, NH_Fmoc_), 4.48–4.32 (m, 16H, CH_α_ + OCH_2_CH_3_ + CH_2Fmoc_ + CH_Fmoc_), 2.99 (d, ^2^*J*_PC_ = 18.1 Hz, 1H, CH-P), 2.87 (d, ^2^*J*_PC_ = 17.8 Hz, 1H, CH-P), 1.72 (s, 3H, CH_3_), 1.68 (s, 3H, CH_3_), 1.46 (d, ^3^*J*_HH_ = 6.9 Hz, 3H, CH_3_), 1.41 (d, ^3^*J*_HH_ = 7.0 Hz, 3H, CH_3_), 1.37–1.35 (m, 12H, OCH_2_CH_3_) 1.33 (s, 9H, *^t^*Bu), 1.30 (s, 9H, *^t^*Bu) ppm; ^13^C {1H} NMR (100 MHz, CDCl_3_) *δ* 182.1 (d, ^3^*J*_PC_ = 4.4 Hz), 181.6 (d, ^3^*J*_PC_ = 4.1 Hz), 166.7, 166.0, 155.7, 155.5, 155.5, 144.0, 143.9, 143.9, 141.5, 141.4, 141.3, 127.9, 127.8, 127.8, 127.7, 127.2, 127.1, 127.1, 127.1, 125.1, 125.1, 125.1, 124.9, 120.2, 120.1, 120.0, 120.0, 66.9, 66.8, 63.7 (d, ^2^*J*_PC_ = 6.4 Hz), 63.5 (d, ^2^*J*_PC_ = 6.3 Hz), 52.6, 52.5, 52.3, 51.7, 49.0, 48.3 (d, ^2^*J*_PC_ = 3.1 Hz), 47.2, 38.8 (d, ^1^*J*_PC_ = 202.7 Hz), 37.9 (d, ^1^*J*_PC_ = 201.8 Hz), 28.6, 28.4, 19.5, 18.3, 16.5, 16.4, 16.4, 16.4, 16.3, 16.3, 16.3, 15.3, 15.0 ppm; ^31^P NMR (160 MHz, CDCl_3_) *δ* 16.8, 16.1 ppm; ESI-HRMS (CI) *m*/*z* calcd. for C_30_H_41_N_3_O_7_P ([M + H]^+^), 586.2682; found 586.2659.

### 3.6. Experimental Procedure and Characterization Data for Phosphorus Substituted Oxazole Derivatives ***7***

Method A: Corresponding Joullié–Ugi adduct **4** derived from phosphine oxide (1 eq.) was dissolved in 20 mL/mmol of acetonitrile, and BF_3_·OEt_2_ (1.2 eq.) was added. The solution was heated under microwave irradiation at 90 °C for 10 min. The solvent was evaporated, and the crude product was diluted with AcOEt, washed with saturated NaHCO_3_ (5 mL) and extracted with AcOEt (3 × 5 mL). The organic layer was dried over anhydrous MgSO_4_ and filtered, and the solvent was evaporated under reduced pressure. The resulting residue was purified via flash column chromatography (SiO_2_, hexanes/AcOEt) or via crystallization to yield the corresponding oxazole derivative. Method B: Corresponding Joullié–Ugi adduct **5** derived from phosphonate (1 eq.) was dissolved in 20 mL/mmol of chloroform, and BF_3_·OEt_2_ (3 eq.) was added. The solution was heated under microwave irradiation at 71 °C for 15 min. The solvent was evaporated, and the crude product was diluted with AcOEt, washed with saturated NaHCO_3_ (5 mL) and extracted with AcOEt (3 × 5 mL). The organic layer was dried over anhydrous MgSO_4_ and filtered, and the solvent was evaporated under reduced pressure. The resulting residue was purified via flash column chromatography (SiO_2_, hexanes/AcOEt) to yield the corresponding oxazole derivative.

(4S*,5S*)-*N*-Cyclohexyl-4-(diphenylphosphoryl)-5-methyl-2-phenyl-4,5-dihydrooxazole-5-carboxamide (**7a**) (105 mg, 94%) was obtained as a white solid from Joullié–Ugi adduct **4a** (111 mg, 0.23 mmol) and BF_3_·OEt_2_ (35 µL, 0.28 mmol), as described in the general procedure in method A. The crude product was purified via flash column chromatography (SiO_2_, AcOEt/hexane 50:50) to give title compound **7a**. mp 209–211 °C; IR (neat) *v_max_* 3325, 2936, 1659, 1589, 1251, 1218, 1151 cm^–1^; ^1^H NMR (400 MHz, CDCl_3_) *δ* 8.15–8.10 (m, 2H, ArH), 7.97–7.90 (m, 4H, ArH), 7.56–7.40 (m, 9H, ArH), 6.91 (d, ^3^*J*_HH_ = 7.9 Hz, 1H, HC-NH), 5.32 (d, ^2^*J*_PC_ = 6.0 Hz, 1H, CH-P), 3.77–3.70 (m, 1H, HC-HN), 1.89–1.87 (m, 1H, *^c^*Hex), 1.82–1.79 (m, 1H, *^c^*Hex), 1.69–1.54 (m, 3H, *^c^*Hex), 1.63 (s, 3H, CH_3_), 1.36–1.11 (m, 5H, *^c^*Hex) ppm; ^13^C {1H} NMR (100 MHz, CDCl_3_) *δ* 171.8 (d, ^3^*J*_PC_ = 9.4 Hz), 164.1 (d, ^3^*J*_PC_ = 10.7 Hz), 132.9, 132.4, 132.2, 132.2, 132.1, 131.9, 131.9, 131.9, 131.8, 131.4, 131.3, 128.9, 128.8, 128.5, 128.5, 128.4, 128.3, 127.0 (d, ^4^*J*_PC_ = 1.6 Hz), 88.7, 71.6 (d, ^1^*J*_PC_ = 81.0 Hz), 48.3, 32.8, 32.7, 25.5, 24.7, 24.6, 21.4 (d, ^3^*J*_PC_ = 6.9 Hz) ppm; ^31^P NMR (160 MHz, CDCl_3_) *δ* 25.6 ppm; ESI-HRMS (CI) *m*/*z* calculated for C_29_H_32_N_2_O_3_P ([M + H]^+^), 487.2151; found 487.2154.

(4S*,5S*)-*N*-Cyclohexyl-4-(diphenylphosphoryl)-5-methyl-2-(3-methylphenyl)-4,5-dihydrooxazole-5-carboxamide **(7b**) (36 mg, 60%) was obtained as a white solid from Joullié–Ugi adduct **4b** (60 mg, 0.12 mmol) and BF_3_·OEt_2_ (18 µL, 0.144 mmol), as described in the general procedure in method A. The crude product was purified via flash column chromatography (SiO_2_, AcOEt/hexane 70:30) to give title compound **7b**. mp 203–205 °C; IR (neat) *v_max_* 3300, 3059, 1660, 1637, 1527, 1194 cm^–1^; ^1^H NMR (400 MHz, CDCl_3_) *δ* 8.17–8.12 (m, 2H, ArH), 7.96–7.94 (m, 2H, ArH), 7.79 (s, 2H, ArH), 7.58–7.35 (m, 8H, ArH), 6.94 (d, ^3^*J*_HH_ = 8.1 Hz, 1H, HC-NH), 5.33 (d, ^2^*J*_PC_ = 6.2 Hz, 1H CH-P), 3.77–3.75 (m, 1H, HC-NH), 2.39 (s, 3H, CH_3_), 1.92–1.81 (m, 2H, *^c^*Hex), 1.73–1.54 (m, 6H, *^c^*Hex + CH_3_), 1.37–1.36 (m, 2H *^c^*Hex), 1.27–1.15 (m, 3H, *^c^*Hex) ppm; ^13^C {1H} NMR (100 MHz, CDCl_3_) *δ* 171.7 (d, ^3^*J*_PC_ = 9.5 Hz), 164.2 (d, ^3^*J*_PC_ = 10.5 Hz), 138.3, 132.9, 132.8, 132.3, 132.1, 131.8, 131.8, 131.7, 131.3, 131.2, 128.9, 128.8, 128.7, 128.4, 128.4, 128.3, 126.8 (d, ^4^*J*_PC_ = 1.6 Hz), 125.5, 88.5, 71.7 (d, ^1^*J*_PC_ = 81 Hz), 48.2, 32.7, 32.7, 25.4, 24.6, 24.5, 21.3, 21.3 ppm; ^31^P NMR (160 MHz, CDCl_3_) *δ* 25.5 ppm; ESI-HRMS (CI) *m*/*z* calculated for C_30_H_34_N_2_O_3_P ([M + H]^+^), 501.2307; found 501.2303.

(4S*,5S*)-*N*-(*tert*-Butyl)-4-(diphenylphosphoryl)-5-methyl-2-phenyl-4,5-dihydrooxazole-5-carboxamide (**7c**) (63 mg, 92%) was obtained as a white solid from Joullié–Ugi adduct **4i** (69 mg 0.15 mmol) and BF_3_·OEt_2_ (22 µL, 0.18 mmol), as described in the general procedure described in method A. The crude product was recrystallized from the diethyl ether–pentane mixture to give **7c**. mp 206–207 °C; IR (neat) *v_max_* 3306, 3075, 2920, 1662, 1629, 1549, 1196, 1118 cm^–1^; ^1^H NMR (400 MHz, CDCl_3_) *δ* 8.15–8.10 (m, 2H, ArH), 7.97–7.90 (m, 4H, ArH), 7.57–7.39 (m, 9H, ArH), 6.87 (s, 1H, NH), 5.32 (d, ^2^*J*_PC_ = 6.2 Hz, 1H, CH-P), 1.61 (s, 3H, CH_3_), 1.33 (s, 9H, *^t^*Bu) ppm; ^13^C {1H} NMR (100 MHz, CDCl_3_) *δ* 171.8 (d, ^3^*J*_PC_ = 9.5 Hz), 164.1 (d, ^3^*J*_PC_ = 10.7 Hz), 132.6 (d, ^1^*J*_PC_ = 66.6 Hz), 132.9, 132.3, 132.1, 132.1, 132.1, 132.0, 131.8, 131.8, 131.8, 131.7, 131.3, 131.2, 128.9, 128.4, 128.5, 128.4, 128.3, 128.3, 127.0, 88.8, 71.4 (d, ^1^*J*_PC_ = 80.7 Hz), 51.4, 28.7, 21.3 (d, ^3^*J*_PC_ = 6.9 Hz) ppm; ^31^P NMR (160 MHz, CDCl_3_) *δ* 25.3 ppm; ESI-HRMS (CI) *m*/*z* calculated for C_27_H_30_N_2_O_3_P ([M + H]^+^), 461.1994; found 461.1995.

(4S*,5S*)-*N*-(*tert*-Butyl)-4-(diphenylphosphoryl)-5-methyl-2-(naphthalen-2-yl)-4,5-dihydrooxazole-5-carboxamide (**7d**) (74 mg, 91%) was obtained as a white solid from Joullié–Ugi adduct **4l** (81 mg, 0.16 mmol) and BF_3_·OEt_2_ (25 µL, 0.20 mmol), as described in the general procedure in method A. The crude product was recrystallized from diethyl ether-pentane mixture to give title compound **7d**. mp 199–202 °C; IR (neat) *v_max_* 3297, 2929, 1667, 1521, 1193, 1117 cm^–1^; ^1^H NMR (400 MHz, CDCl_3_) *δ* 8.45 (s, 1H, ArH), 8.18–8.13 (m, 2H, ArH), 8.06–8.04 (m, 1H ArH), 8.01–7.96 (m, 2H, ArH), 7.92–7.86 (m, 3H, ArH), 7.57–7.42 (m, 8H, ArH), 7.02 (s, 1H, NH), 5.38 (d, ^2^*J*_PH_ = 6.7 Hz, 1H, CH-P), 1.66 (s, 3H, CH_3_), 1.35 (s, 9H, *^t^*Bu) ppm; ^13^C {1H} NMR (100 MHz, CDCl_3_) *δ* 171.7 (d, ^3^*J*_PC_ = 9.1 Hz), 164.3 (d, ^3^*J*_PC_ = 10.9 Hz), 135.1, 133.0, 132.7, 132.3, 132.3, 132.2, 132.0, 132.0, 131.9, 131.8, 131.4, 131.3, 131.2, 129.1, 129.0, 128.9, 128.5, 128.4, 128.1, 127.9, 126.9, 124.7, 124.3 (d, ^4^*J*_PC_ = 1.6 Hz), 89.0, 71.7 (d, ^1^*J*_PC_ = 80.6 Hz), 51.5, 28.7, 21.4 (d, ^3^*J*_PC_ = 6.8 Hz) ppm; ^31^P NMR (160 MHz, CDCl_3_) *δ* 25.4 ppm; ESI-HRMS (CI) *m*/*z* calculated for C_31_H_32_N_2_O_3_P ([M + H]^+^), 511.2151; found 511.2134.

Diethyl ((4S*,5S*)-5-(*tert*-butylcarbamoyl)-5-methyl-2-phenyl-4,5-dihydrooxazol-5-yl)phosphonate (**7e**) (285 mg, 90%) was obtained as a white solid from Joullié–Ugi adduct **5a** (316 mg, 0.80 mmol) and BF_3_·OEt_2_ (296 µL, 2.40 mmol), as described in the general procedure, method B. The crude product was purified via flash column chromatography (SiO_2_, AcOEt/hexane 65:35) to give title compound **7e**. mp 99–102 °C; IR (neat) *v_max_* 3294, 3067, 2975, 1665, 1637, 1538, 1252, 1213, 1074, 1052 cm^–1^; ^1^H NMR (400 MHz, CDCl_3_) *δ* 7.97–7.95 (m, 2H, ArH), 7.52 (d, ^3^*J*_HH_ = 7.4 Hz, 1H, ArH), 7.44–7.41 (m, 2H, ArH), 6.31 (s, 1H, NH), 4.81 (d, ^2^*J*_PC_ = 17.3 Hz, 1H, CH-P), 4.36–4.18 (m, 4H, OCH_2_CH_3_), 1.89 (d, ^4^*J*_PH_ = 0.6, 3H, CH_3_), 1.39–1.34 (m, 6H, OCH_2_CH_3_), 1.31 (s, 9H, *^t^*Bu) ppm; ^13^C {1H} NMR (100 MHz, CDCl_3_) *δ* 172.2 (d, ^3^*J*_PC_ = 14.9 Hz), 163.7 (d, ^3^*J*_PC_ = 12.0 Hz), 132.1, 128.6, 128.3, 127.1 (d, ^4^*J*_PC_ = 2.6 Hz), 87.4, 69.9 (d, ^1^*J*_PC_ = 160.4 Hz), 63.8 (d, ^2^*J*_PC_ = 6.7 Hz), 62.8 (d, ^2^*J*_PC_ = 7.4 Hz), 51.3, 28.7, 20.9 (d, ^3^*J*_PC_ = 6.2 Hz), 16.5 (t, ^3^*J*_PC_ = 5.6 Hz) ppm; ^31^P NMR (160 MHz, CDCl_3_) *δ* 18.5 ppm; ESI-HRMS (CI) *m*/*z* calculated for C_19_H_30_N_2_O_5_P ([M + H]^+^), 397.1892; found 397.1878.

Diethyl ((4S*,5S*)-5-(*tert*-butylcarbamoyl)-2-(4-fluorophenyl)-5-methyl-4,5-dihydrooxazol-4-yl)phosphonate (**7f**) (53 mg, 80%) was obtained as a yellowish oil obtained from Joullié–Ugi adduct **5b** (66 mg, 0.16 mmol) and BF_3_·OEt_2_ (57 µL, 0.48 mmol), as described in the general procedure method B. The crude product was purified via flash column chromatography (SiO_2_, AcOEt/hexane 65:35) to give title compound **7f**. R_f_: 0.70 (AcOEt); IR (neat) *v_max_* 3311, 3067, 2978, 1649, 1587, 1252, 1227, 1040 cm^–1^; ^1^H NMR (400 MHz, CDCl_3_) *δ* 8.00 (dd, ^3^*J*_HH_ = 8.8 Hz, ^4^*J*_HF_ = 5.4 Hz, 2H, ArH), 7.14 (t, ^3^*J*_HH_ = 8.7 Hz, ^3^*J*_HF_ = 8.7 Hz, 2H, ArH), 6.31 (s, 1H, NH), 4.82 (d, ^2^*J*_PC_ = 17.3 Hz, 1H, CH-P), 4.40–4.19 (m, 4H, OCH_2_CH_3_), 1.91 (s, 3H, CH_3_), 1.45–1.35 (m, 6H, OCH_2_CH_3_), 1.34 (s, 9H, *^t^*Bu) ppm; ^13^C {1H} NMR (100 MHz, CDCl_3_) *δ* 171.9 (d, ^3^*J*_PC_ = 14.9 Hz), 165.1 (d, ^1^*J*_CF_ = 253.2 Hz), 163.5 (d, ^3^*J*_PC_ = 12.0 Hz), 130.6, 130.5, 123.2 (t, ^4^*J*_CF_ = 2.9 Hz), 115.9, 115.7, 87.6 (d, ^2^*J*_PC_ = 1.9 Hz), 69.7 (d, ^1^*J*_PC_ = 160.7 Hz), 63.6 (d, ^3^*J*_PC_ = 6.7 Hz), 62.8 (d, ^2^*J*_PC_ = 7.3 Hz), 51.3, 28.6, 20.8 (d, ^3^*J*_PC_ = 6.1 Hz), 16.5 (t, ^3^*J*_PC_ = 5.6 Hz) ppm; ^19^F NMR (376 MHz, CDCl_3_) *δ* –106.8 ppm; ^31^P NMR (160 MHz, CDCl_3_) *δ* 18.4 ppm; ESI-HRMS (CI) *m*/*z* calculated for C_19_H_29_FN_2_O_5_P ([M + H]^+^), 415.1798; found 415.1771.

## 4. Conclusions

In conclusion, we developed a novel strategy to efficiently access to phosphorylated *N*-acylaziridines through the zinc chloride-catalyzed Joullié–Ugi three-component reaction of phosphorus-substituted 2*H*-azirines, carboxylic acids and isocyanides. Most of the JU-3CR proceeded smoothly in THF for a few hours, giving exclusive diastereoselectivity and satisfactory yields. This protocol was applicable to a wide range of substrates, including 2*H*-azirines derived from phosphine oxide and phosphonate, various aromatic and heteroaromatic, aliphatic, acrylic and propargylic acids, and isocyanide with different alkyl substitutions. Even *N*-Fmoc protected amino acids as carboxylic acid partners are well tolerated and phosphorylated aziridine peptidomimetics are achieved in a simple procedure. This strategy for the preparation of phosphorylated *N*-acylaziridines represents a valued method owing to the high degree of diastereoselectivity observed, the high atom economy and the reaction stages. The synthetic potential of this JU-3CR was established with the preparative-scale reaction and useful transformations of the JU-3CR adducts. The regio- and stereospecific ring expansion of *N*-acylaziridines to oxazole derivatives was accomplished in the presence of BF_3_·OEt_2_ as an efficient Lewis acid catalyst.

## Figures and Tables

**Table 1:** Reaction condition optimization ^a^.

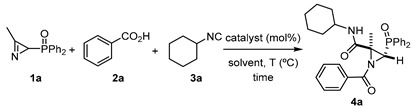					
**Entry**	**Catalyst (mol%)**	**Time (h)**	**T (**°C**)**	**Solvent**	**Yield (%) ^b^**
1			60	THF	10
2	PTSA (25)	1.5	60	THF	14
3	TfOH (10)	1.5	60	THF	35
4	Ti(O*^i^*Pr)_4_ (25)	1	60	THF	0
5	Sc(OTf)_3_ (25)	1	60	THF	0
6	InCl_3_ (30)	1.5	60	THF	0
7	BF_3_·OEt_2_ (25)	1	60	THF	0
8	MgBr_2_ (25)	1.5	60	THF	trace
9	ZnCl_2_·H_2_O (25)	1.5	60	THF	0
10	ZnCl_2_ (25)	1.5	60	THF	60
11	ZnCl_2_ (25)	1	60	MeOH	0
12	ZnCl_2_ (25)	24	60	MeCN	0
13	ZnCl_2_ (25)	24	−10	THF	35
14	ZnCl_2_ (25)	5	rt	THF	75
15	ZnCl_2_ (10)	5	rt	THF	35
16	ZnCl_2_ (30)	5	rt	THF	75

^a^ Unless otherwise noted, reactions were carried out on a 0.5 mmol scale; 2*H*-azirine **1a** (0.5 mmol), carboxylic acid **2a** (1.3 eq.), isocyanide **3a** (1.3 eq.), catalyst (25 mol%) and solvent (0.5 mL). ^b^ Isolated yields.

## Data Availability

The data presented in this study are available in the Supplementary Materials file or on request from the corresponding author (^1^H, ^13^C, ^19^F, ^31^P, 2D-NMR and HRMS spectra).

## Supplementary Materials

The following supporting information can be downloaded at https://www.mdpi.com/article/10.3390/molecules29051023/s1: ^1^H, ^13^C{1H}, ^19^F, ^31^P NMR spectra of synthesized compounds **4**, **5**, **6** and **7**; Figure S1: ORTEP diagram of compound **4i** with thermal displacement parameters drawn at a 50% probability; Table S1: Crystal data and structure refinement for **4i**; Figure S2: ORTEP diagram of compound **7a** with thermal displacement parameters drawn at a 50% probability; Table S2: Crystal data and structure refinement for **7a**.
