# Supplementary material for: Diastereoselective ZnCl2-Mediated Joullié–Ugi Three-Component Reaction for the Preparation of Phosphorylated N-Acylaziridines from 2H-Azirines
Source: Molecules. 2024 Feb 27;29(5):1023. doi: 10.3390/molecules29051023 (PMC10933767; doi:10.3390/molecules29051023)
Supplement: Supplementary file 1 [file molecules-29-01023-s001.zip › molecules-2886840-supplementary.pdf]

Supplementary Materials

Diastereoselective ZnCl2-Mediated Joullié-Ugi Three-Component Reaction for the Preparation of Phosphorylated *N*-Acylaziridines from 2*H*-Azirines

Julene Allende, Iurre Olaizola, Ana M. Ochoa de Retana, Francisco Palacios and Jesús M. de los Santos *

Department of Organic Chemistry I, Faculty of Pharmacy, Lascaray Research Center, University of the Basque Country (UPV/EHU), Paseo de la Universidad 7, 01006 Vitoria, Spain
* Correspondence: jesus.delossantos@ehu.eus; Tel.: +34-945-013-104

Table of contents

1. ^{1}H NMR, ^{13}C NMR, ^{31}P NMR, ^{19}F NMR and 2D HMBC NMR {^{1}H-^{13}C} spectra of compounds........................ S2
2. ORTEP view and X-ray crystallographic statistics for compound **4i** and **7a** S76
3. References S80

1. ^{1}H NMR, ^{13}C NMR, ^{31}P NMR, ^{19}F NMR and 2D HMBC NMR {^{1}H-^{13}C} spectra of compounds

^{1}H NMR (400 MHz, CDCl$_3$) of *N*-Acylaziridine Phosphine Oxide **4a**

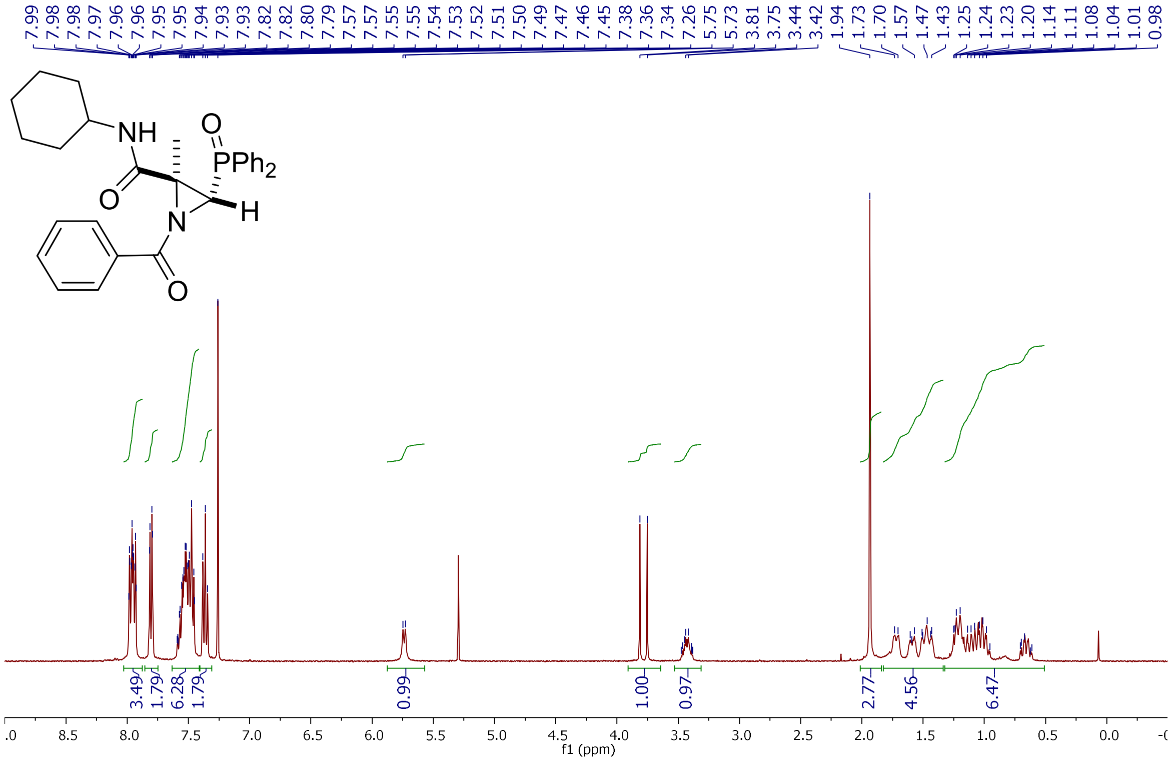

^{13}C {^{1}H} NMR (100 MHz, CDCl$_3$) of *N*-Acylaziridine Phosphine Oxide **4a**

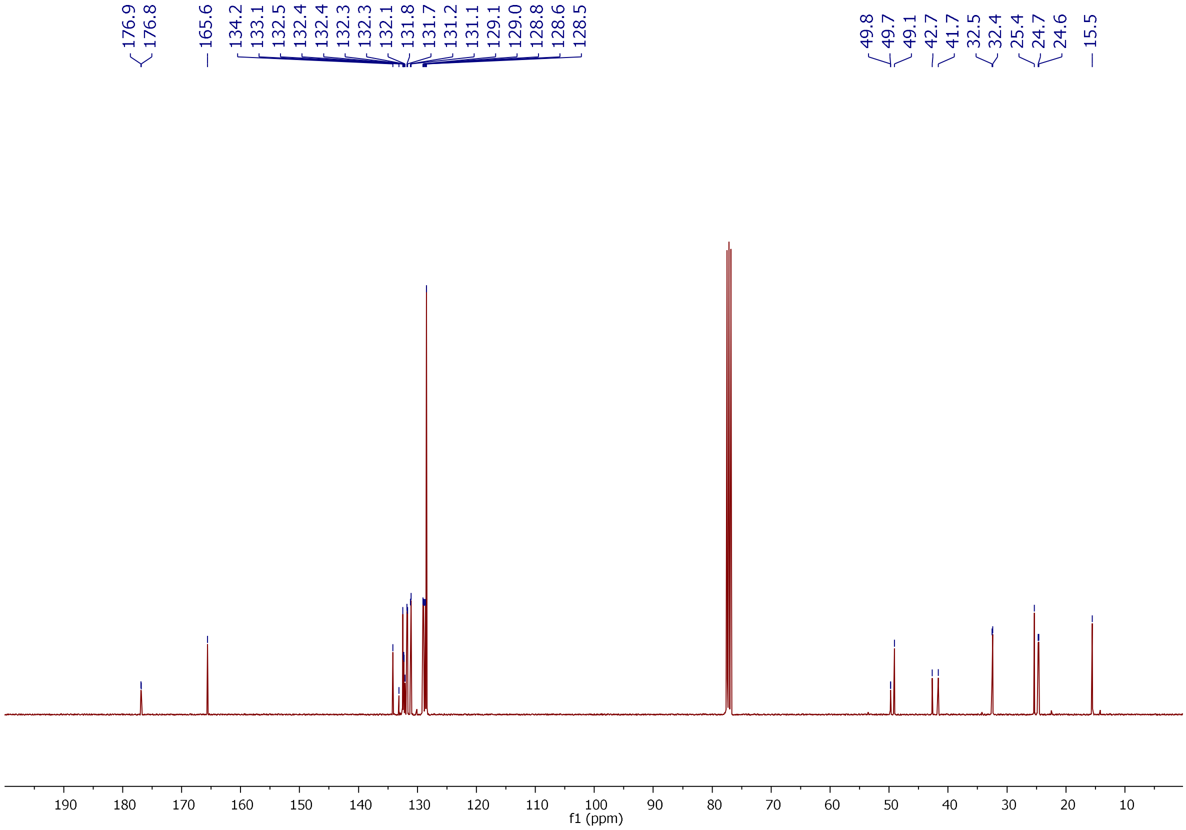

^{31}P (160 MHz, $CDCl_3$) of *N*-Acylaziridine Phosphine Oxide **4a**

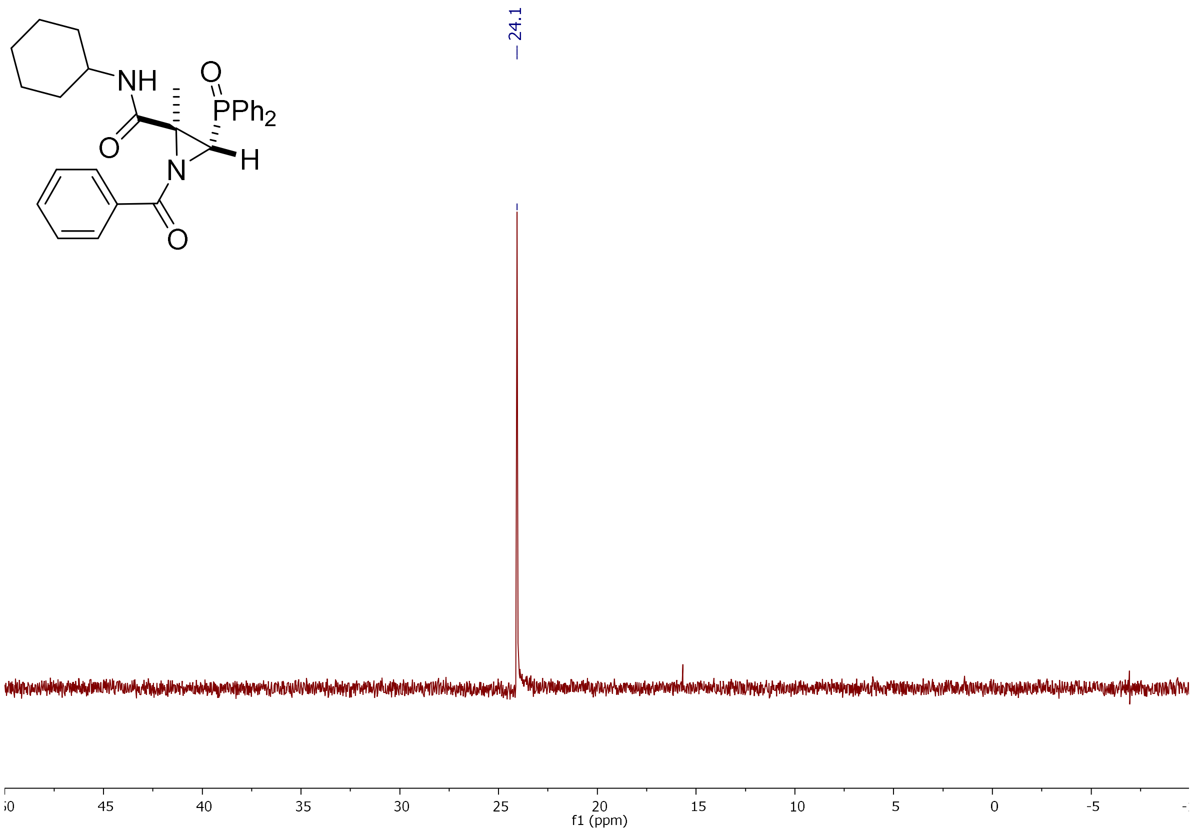

^{1}H-^{13}C HMBC ($CDCl_3$) of *N*-Acylaziridine Phosphine Oxide **4a**

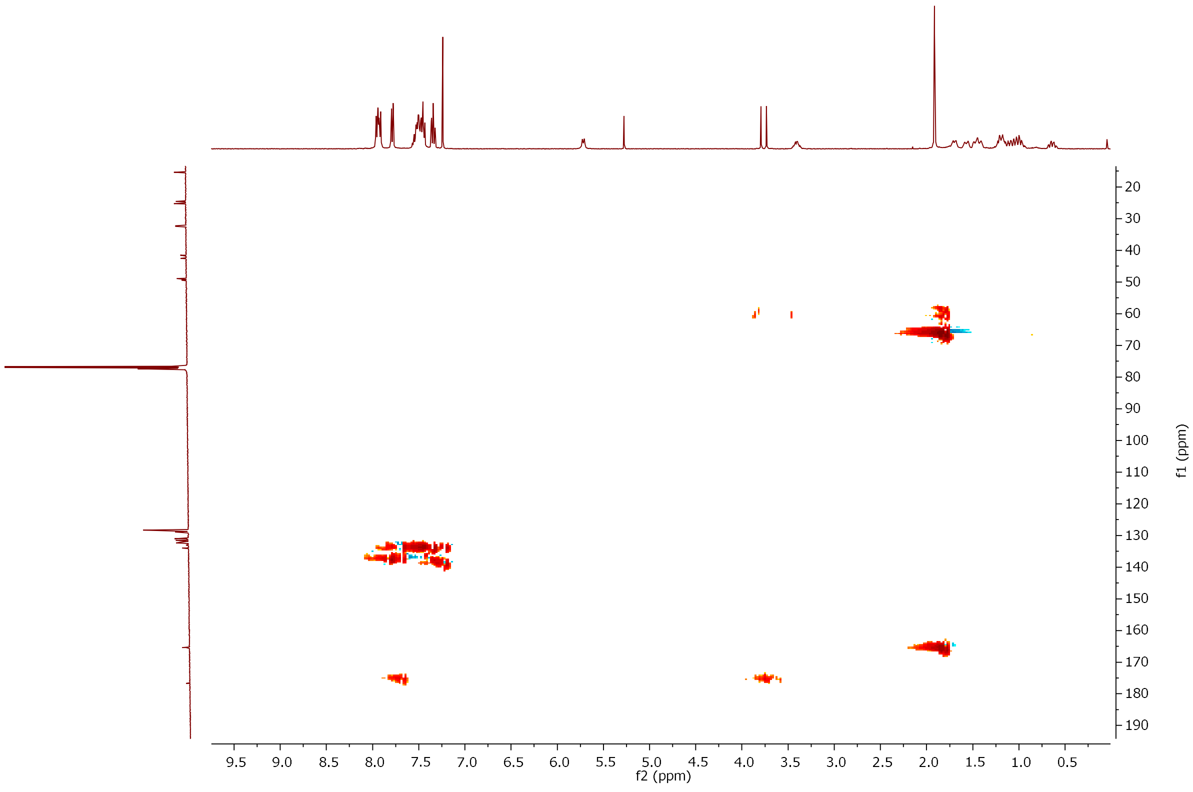

^{1}H NMR (400 MHz, $CDCl_3$) of *N*-Acylaziridine Phosphine Oxide **4b**

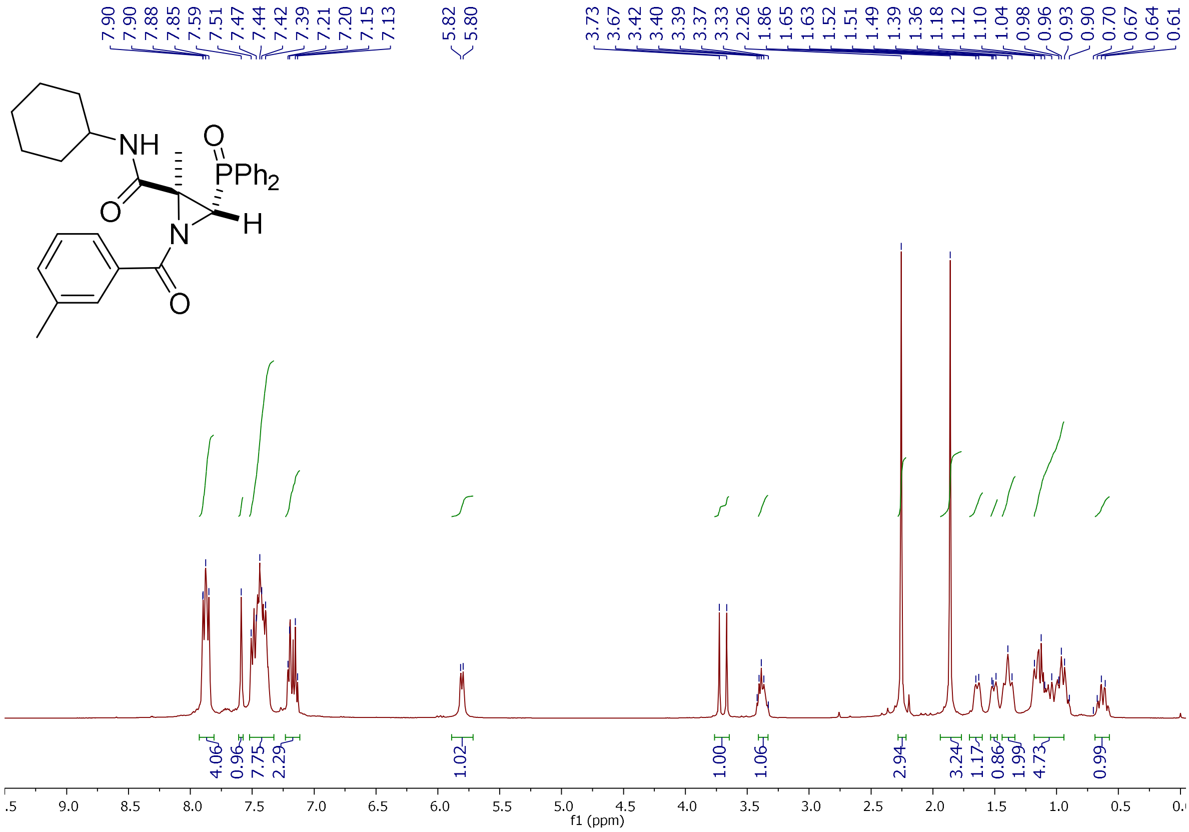

^{13}C {^{1}H} NMR (100 MHz, $CDCl_3$) of *N*-Acylaziridine Phosphine Oxide **4b**

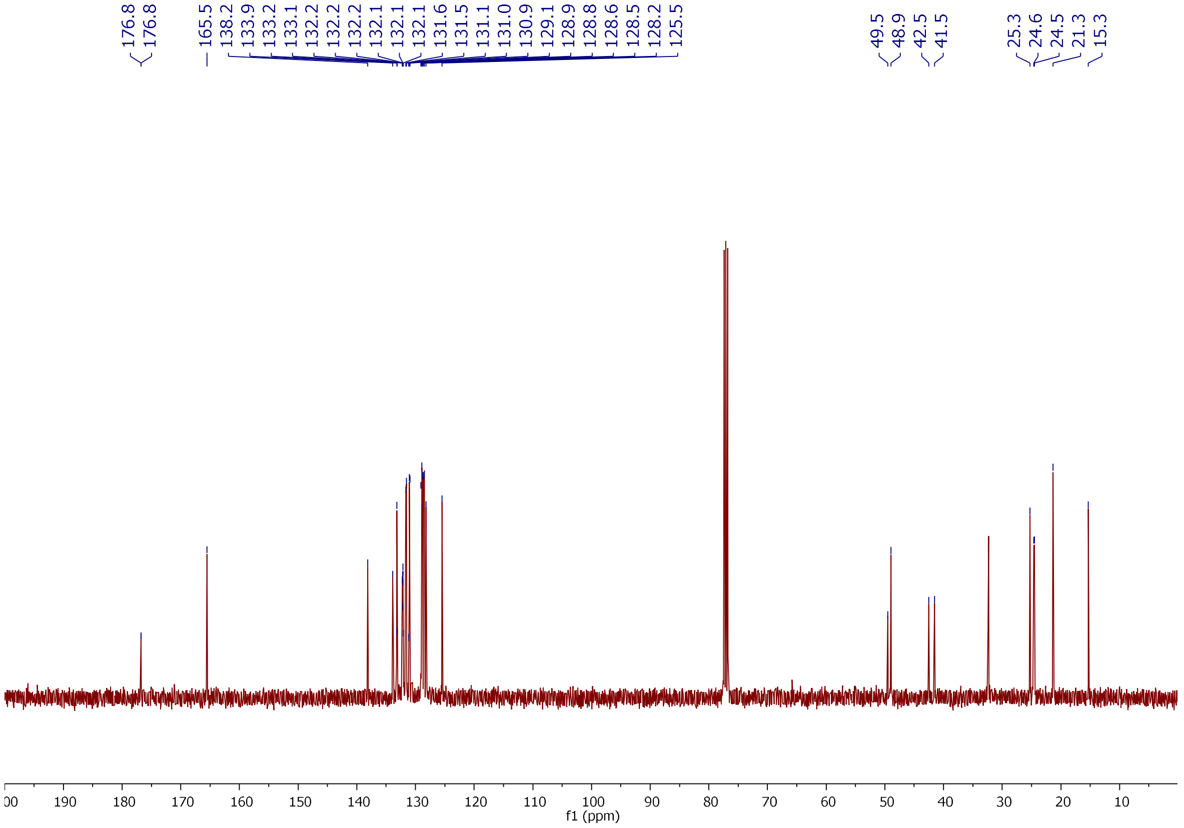

^{31}P (160 MHz, $CDCl_3$) of *N*-Acylaziridine Phosphine Oxide **4b**

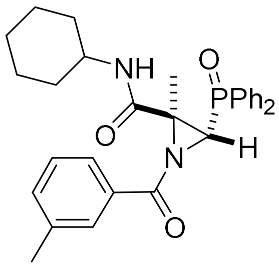

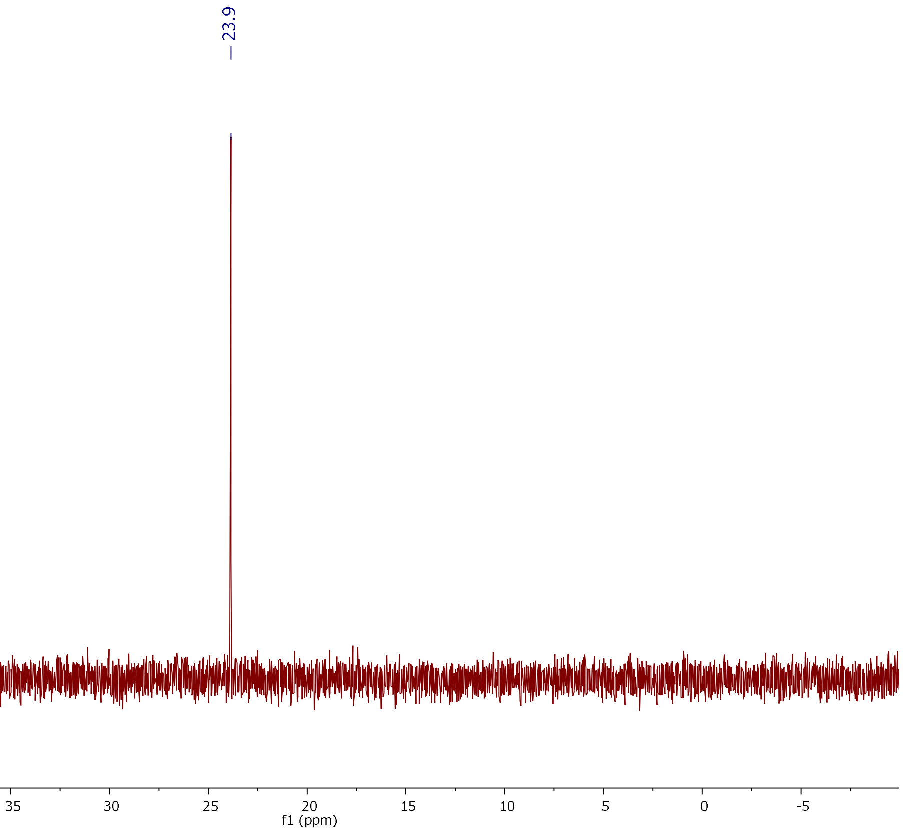

^{1}H NMR (400 MHz, $CDCl_3$) of *N*-Acylaziridine Phosphine Oxide **4c**

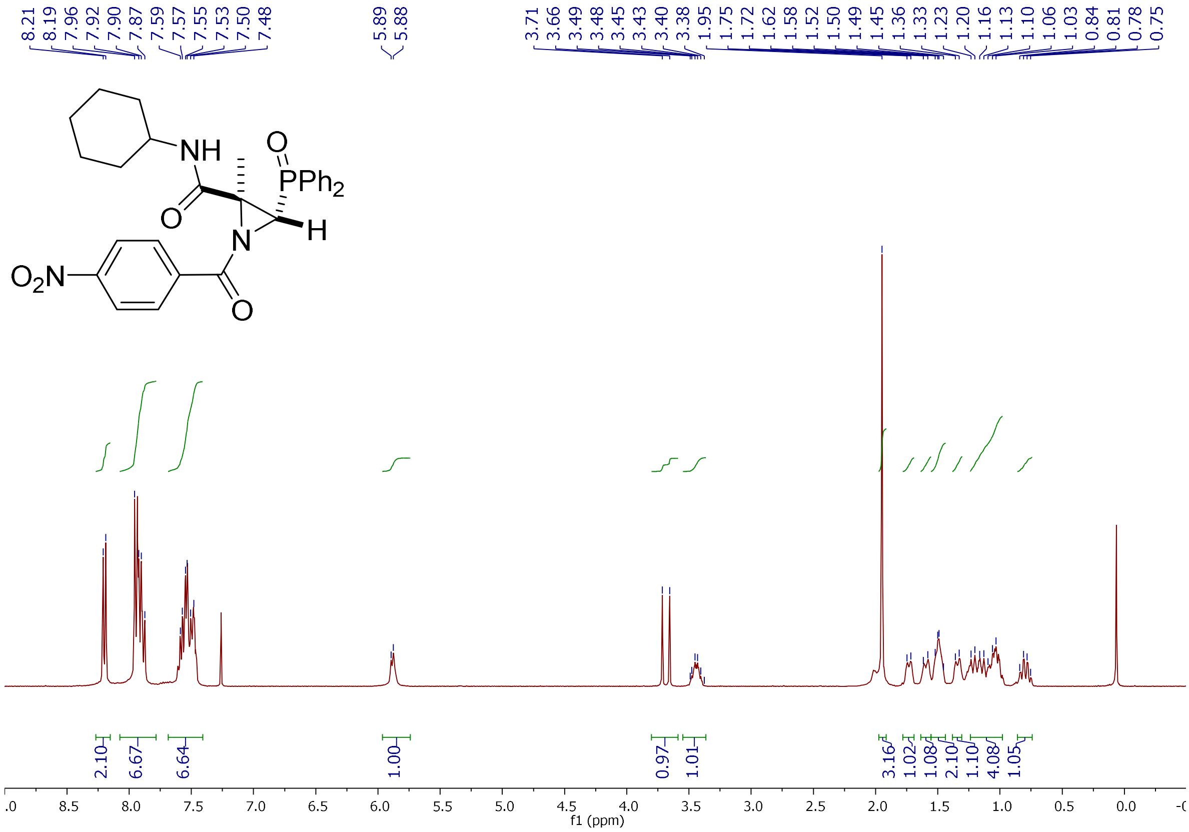

^{13}C {^{1}H} NMR (100 MHz, $CDCl_3$) of *N*-Acylaziridine Phosphine Oxide **4c**

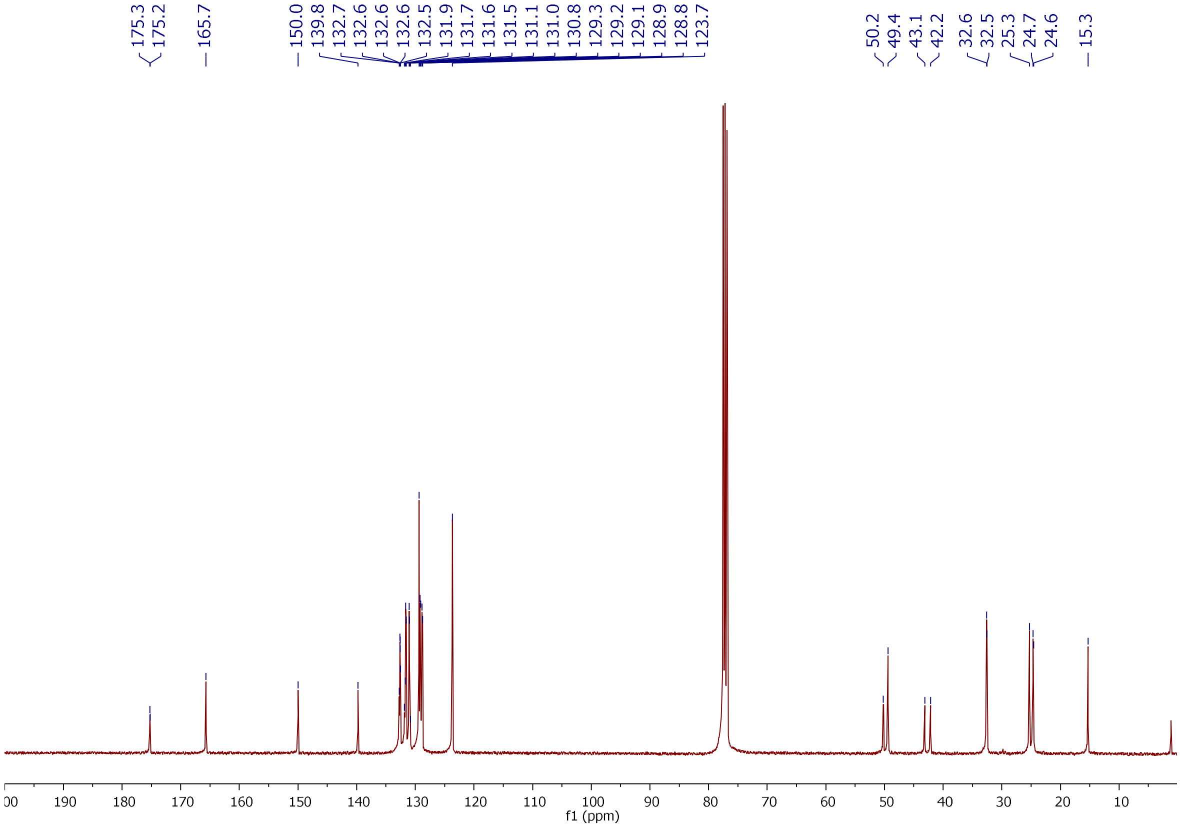

^{31}P (160 MHz, $CDCl_3$) of *N*-Acylaziridine Phosphine Oxide **4c**

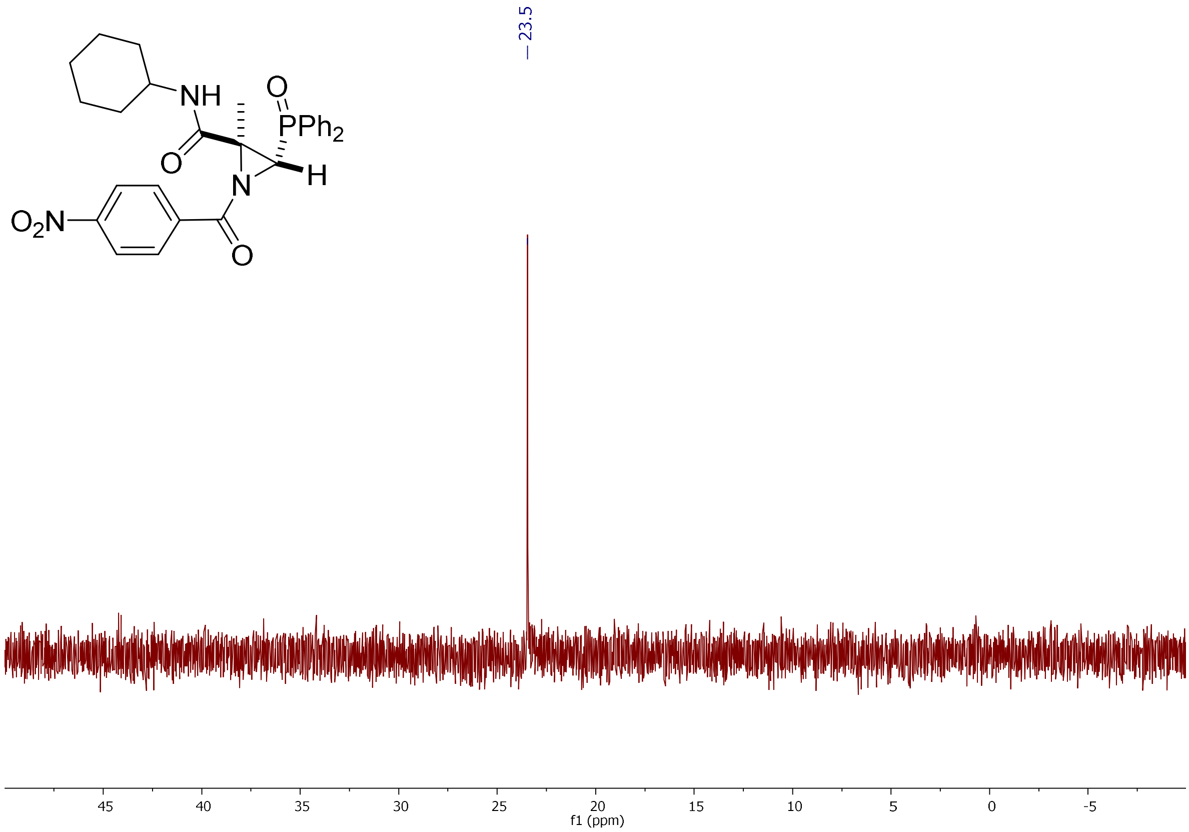

^{1}H NMR (400 MHz, $CDCl_3$) of *N*-Acylaziridine Phosphine Oxide **4d**

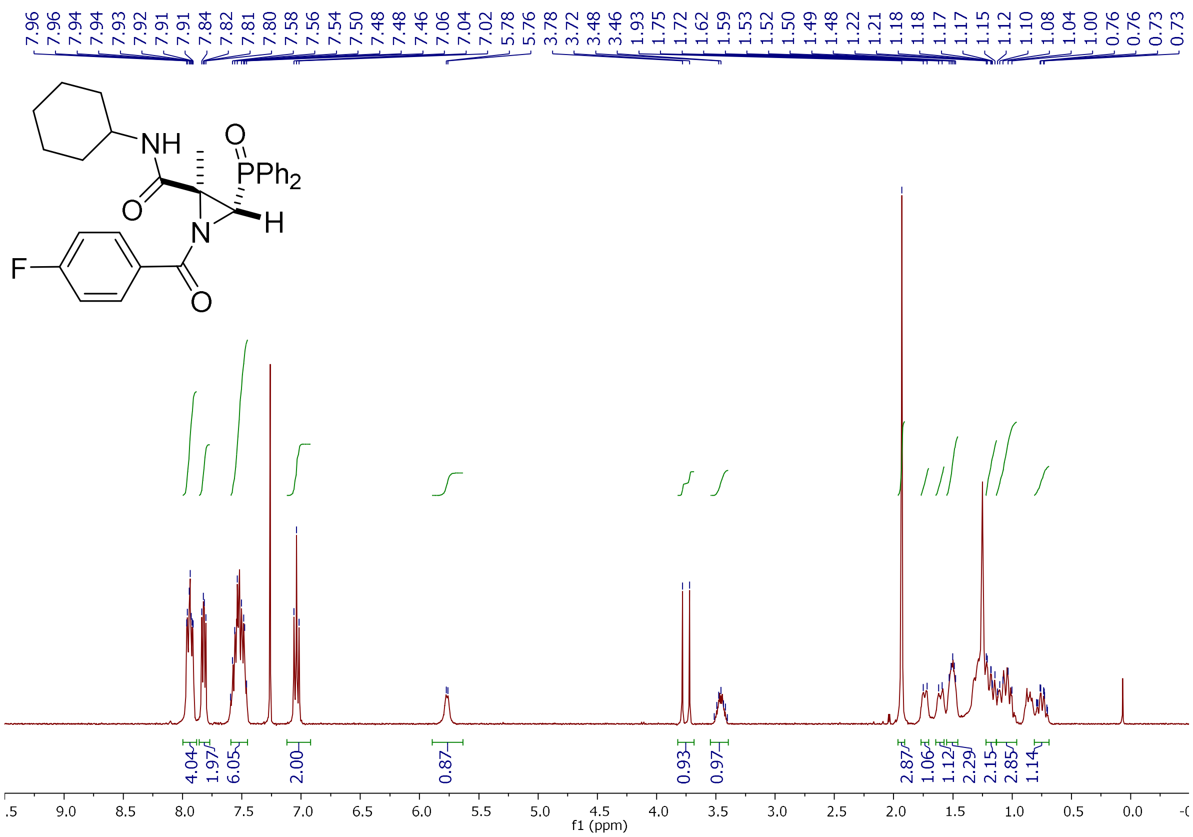

$^{13}C\ \{^{1}H\}$ NMR (100 MHz, $CDCl_3$) of *N*-Acylaziridine Phosphine Oxide **4d**

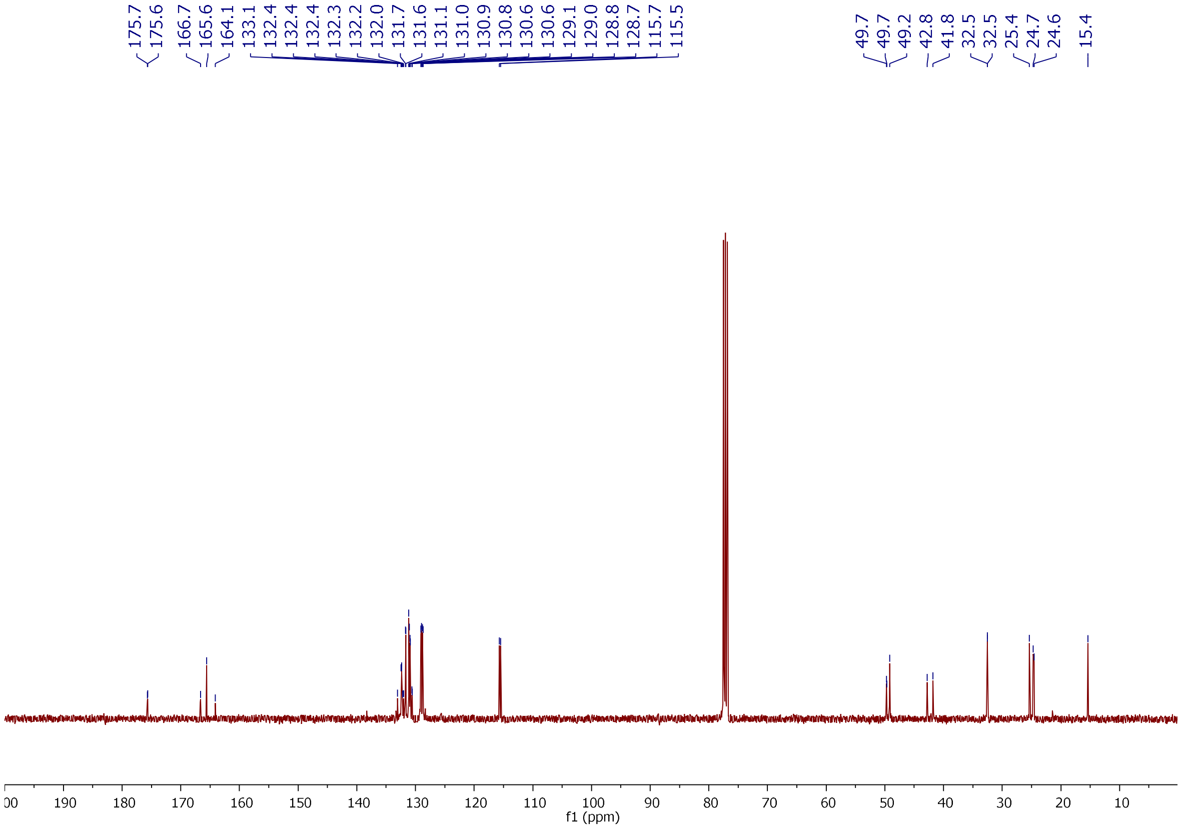

^{31}P (160 MHz, $CDCl_3$) of *N*-Acylaziridine Phosphine Oxide **4d**

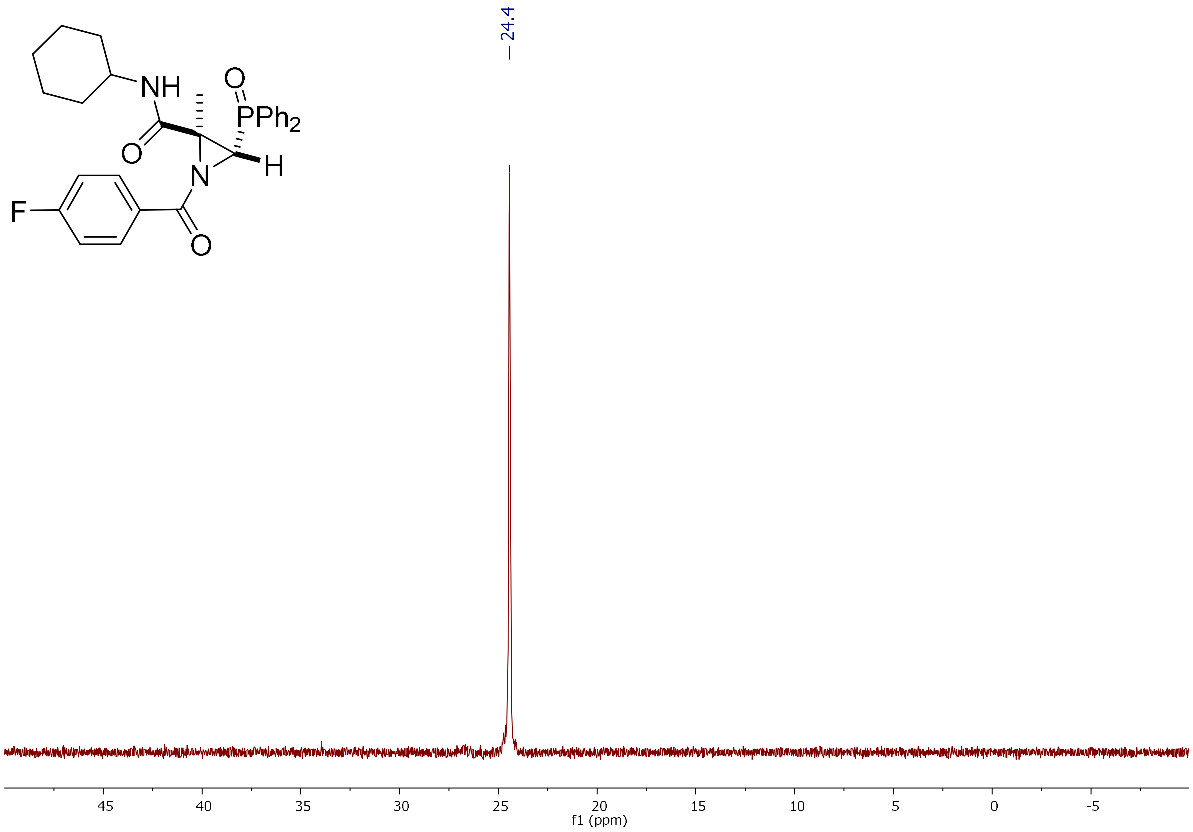

^{19}F (376 MHz, $CDCl_3$) of *N*-Acylaziridine Phosphine Oxide **4d**

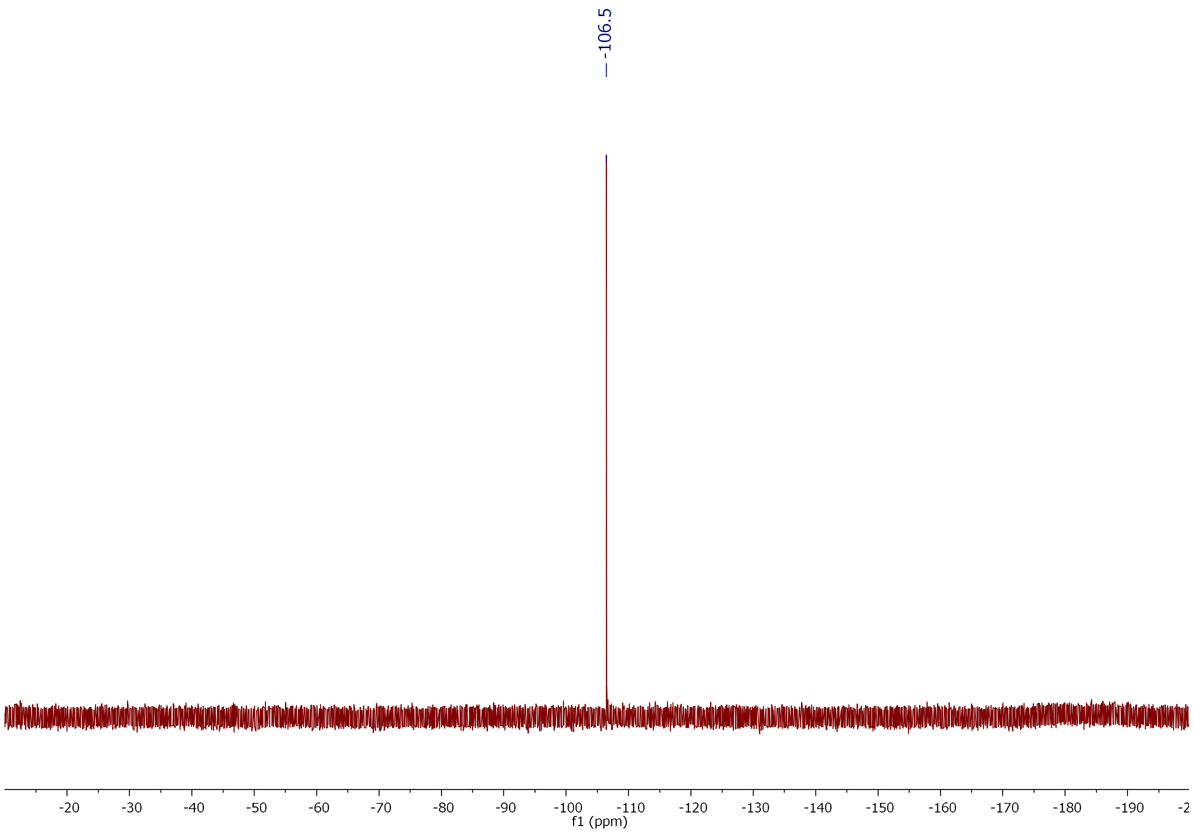

^{1}H NMR (400 MHz, $CDCl_3$) of *N*-Acylaziridine Phosphine Oxide **4e**

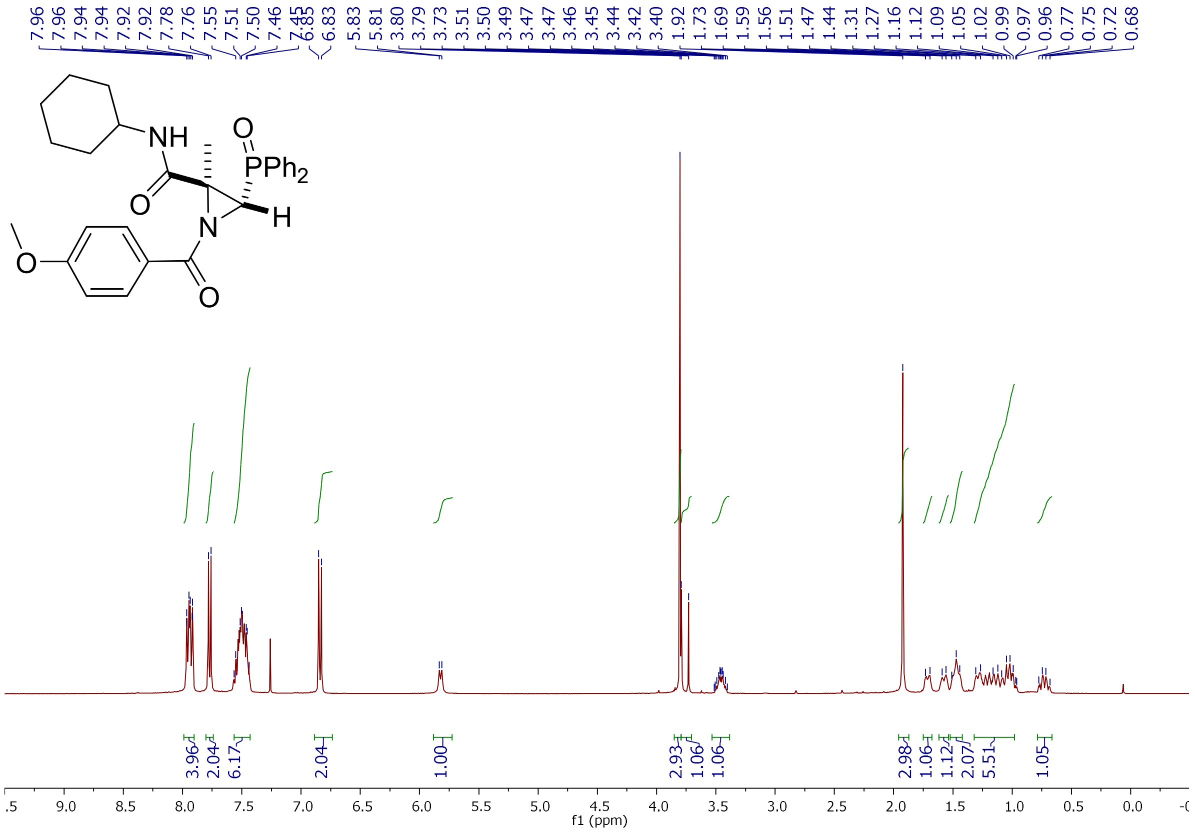

^{13}C {^{1}H} NMR (100 MHz, $CDCl_3$) of *N*-Acylaziridine Phosphine Oxide **4e**

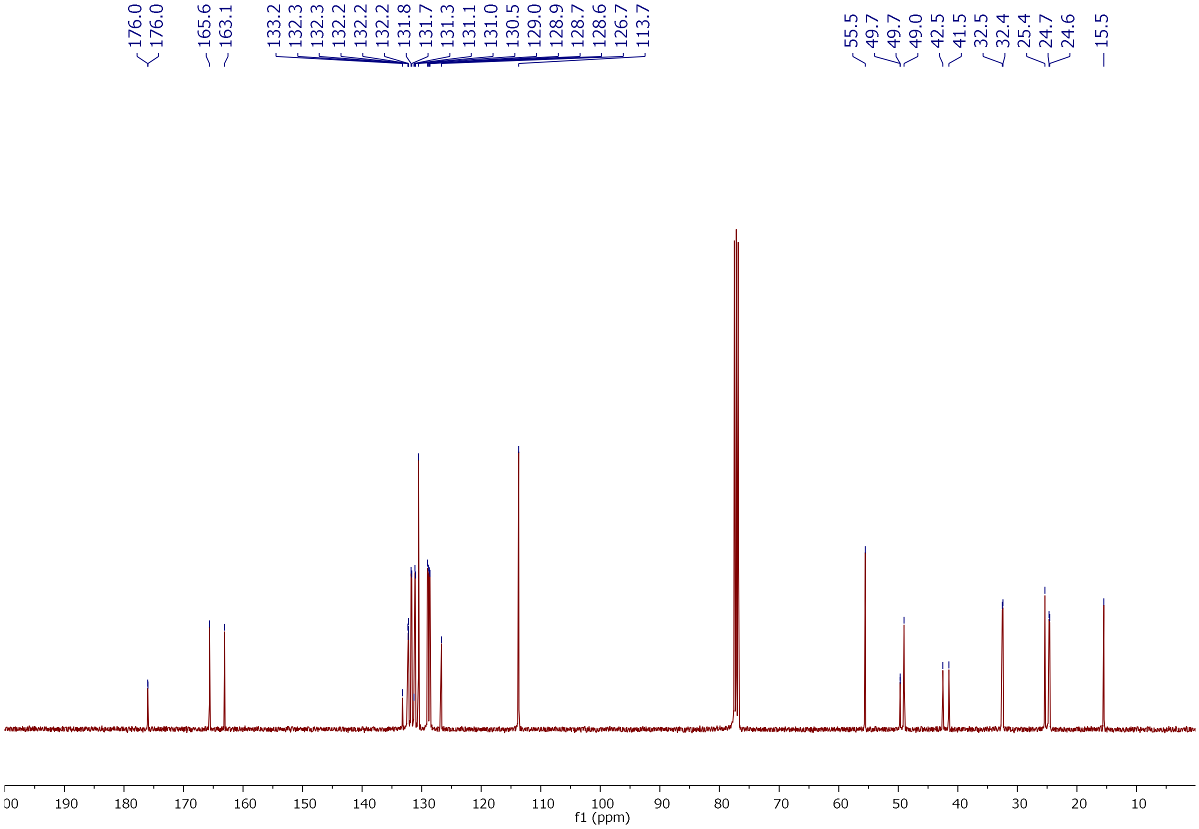

^{31}P (160 MHz, $CDCl_3$) of *N*-Acylaziridine Phosphine Oxide **4e**

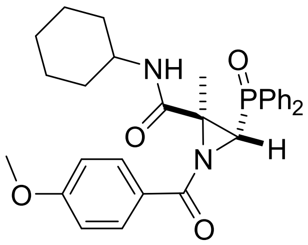

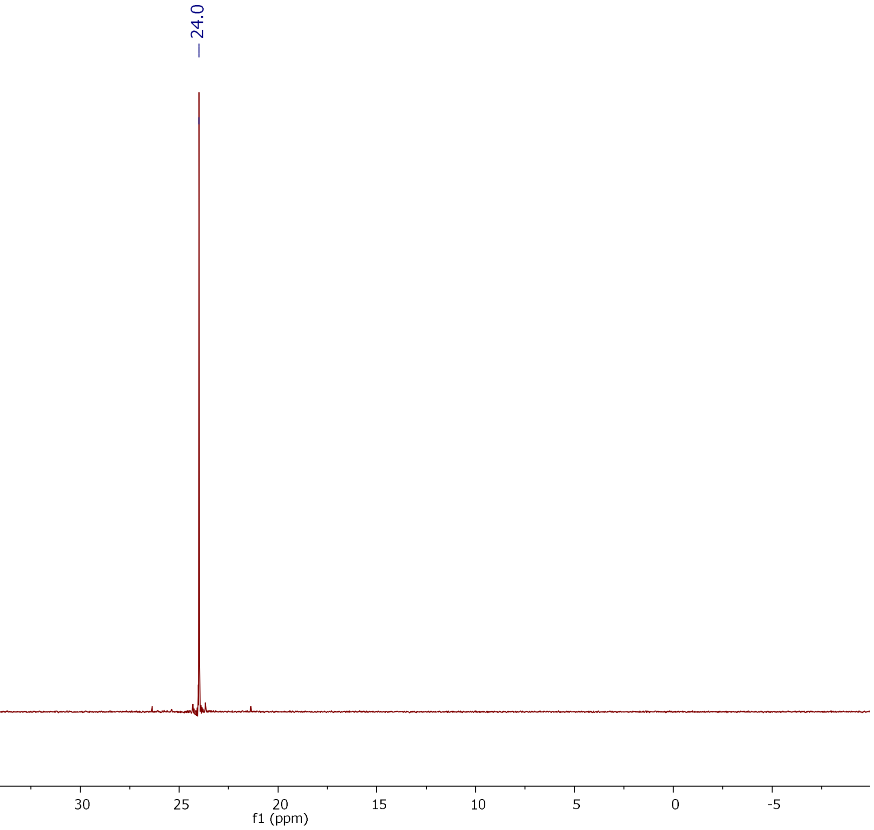

^{1}H NMR (400 MHz, $CDCl_3$) of *N*-Acylaziridine Phosphine Oxide **4f**

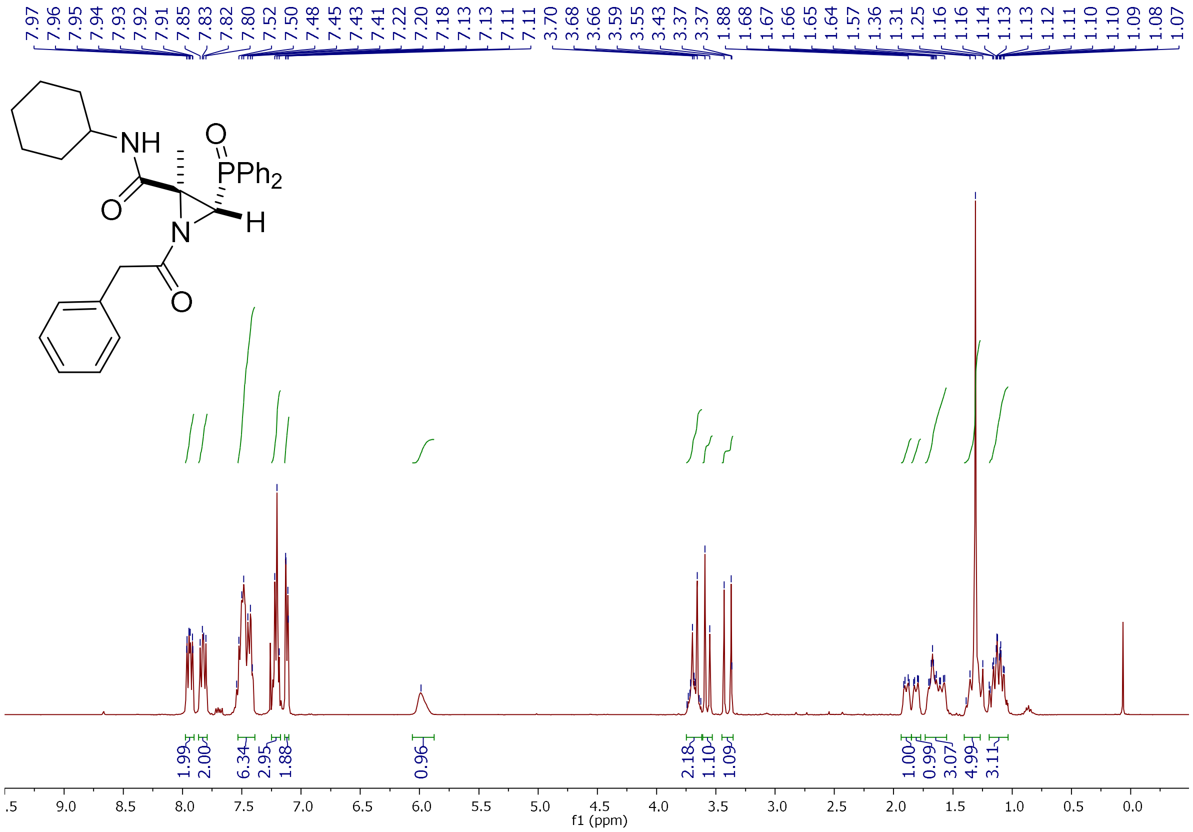

^{13}C {^{1}H} NMR (100 MHz, $CDCl_3$) of *N*-Acylaziridine Phosphine Oxide **4f**

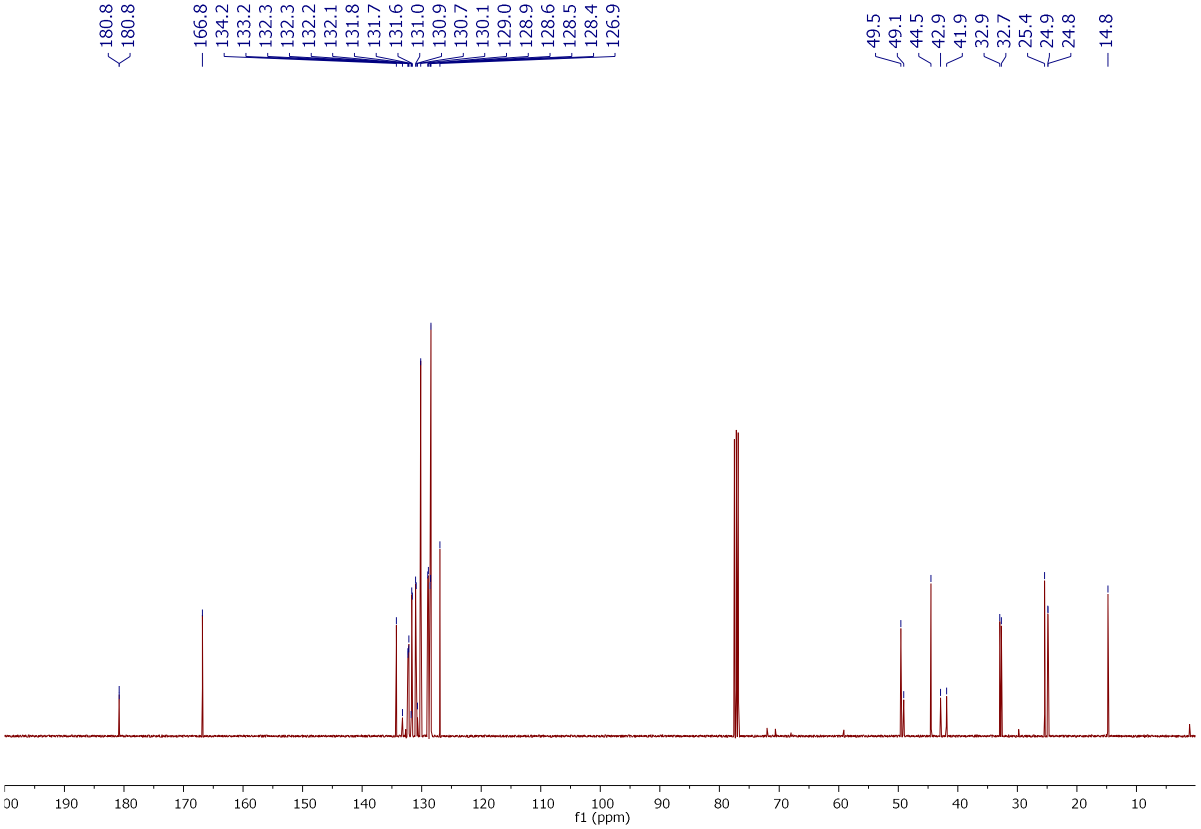

^{31}P (160 MHz, CDCl$_3$) of *N*-Acylaziridine Phosphine Oxide **4f**

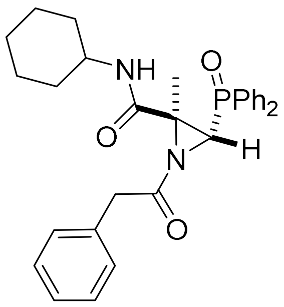

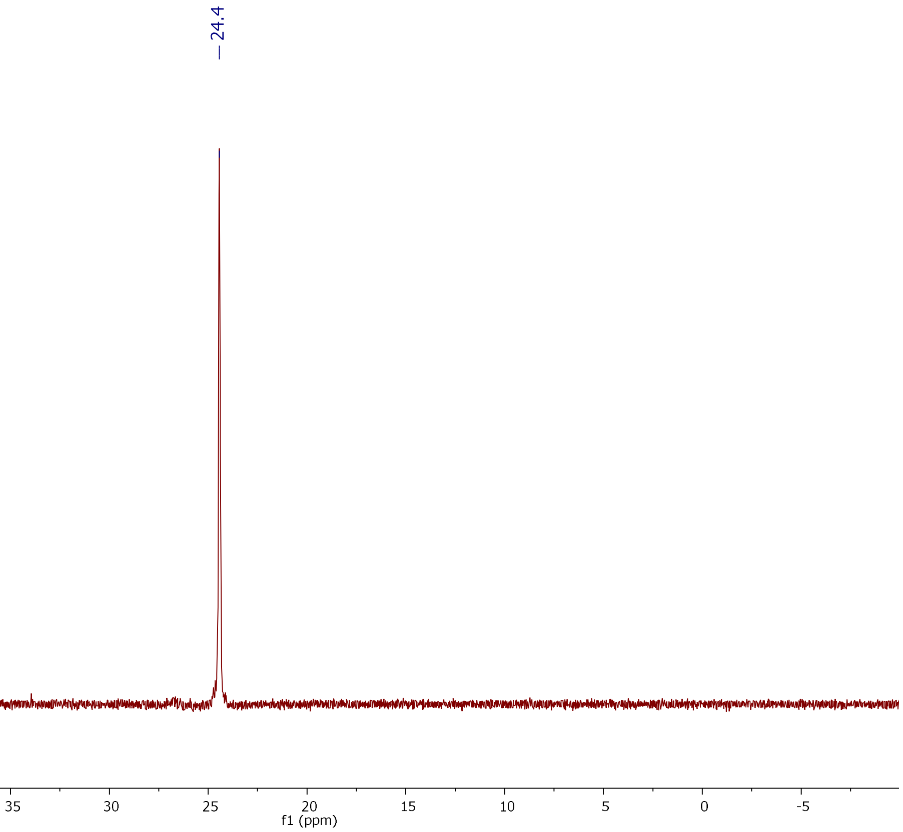

^{1}H NMR (400 MHz, $CDCl_3$) of *N*-Acylaziridine Phosphine Oxide **4g**

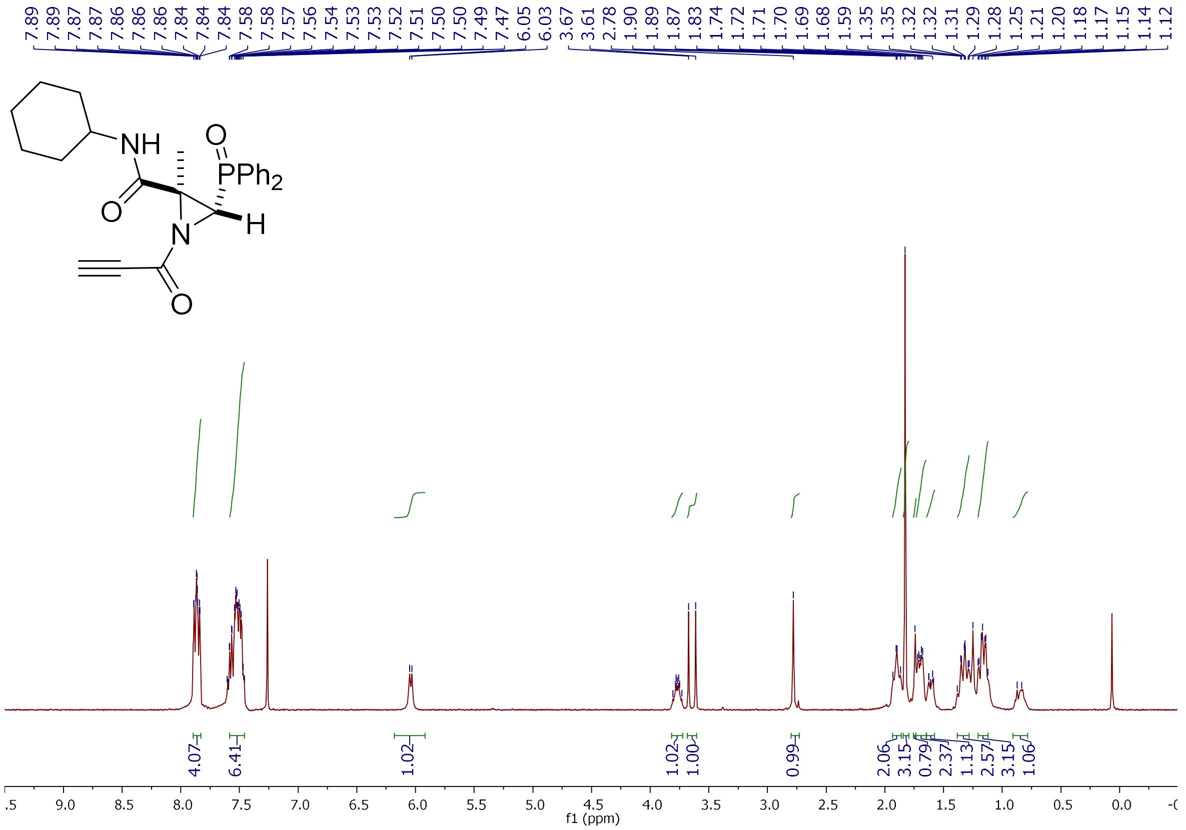

^{13}C {^{1}H} NMR (100 MHz, $CDCl_3$) of *N*-Acylaziridine Phosphine Oxide **4g**

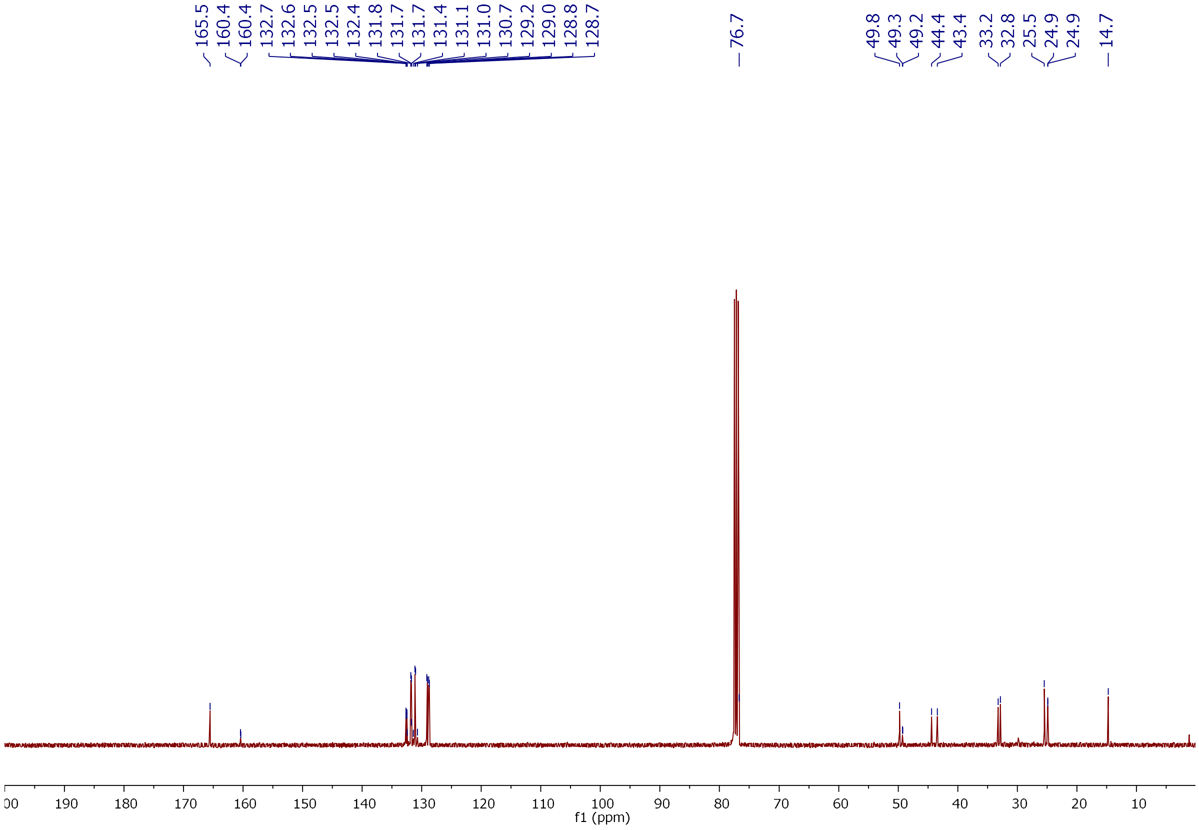

^{31}P (160 MHz, $CDCl_3$) of *N*-Acylaziridine Phosphine Oxide **4g**

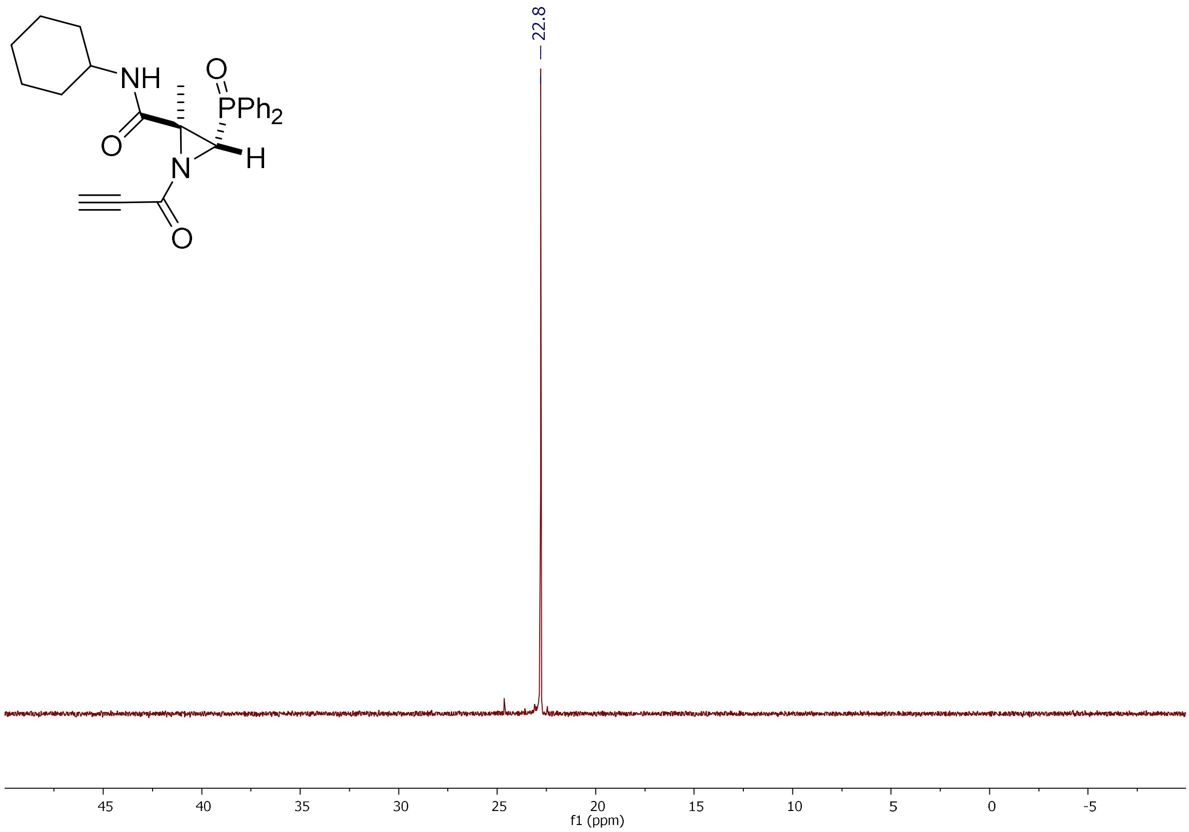

^{1}H NMR (400 MHz, $CDCl_3$) of *N*-Acylaziridine Phosphine Oxide **4h**

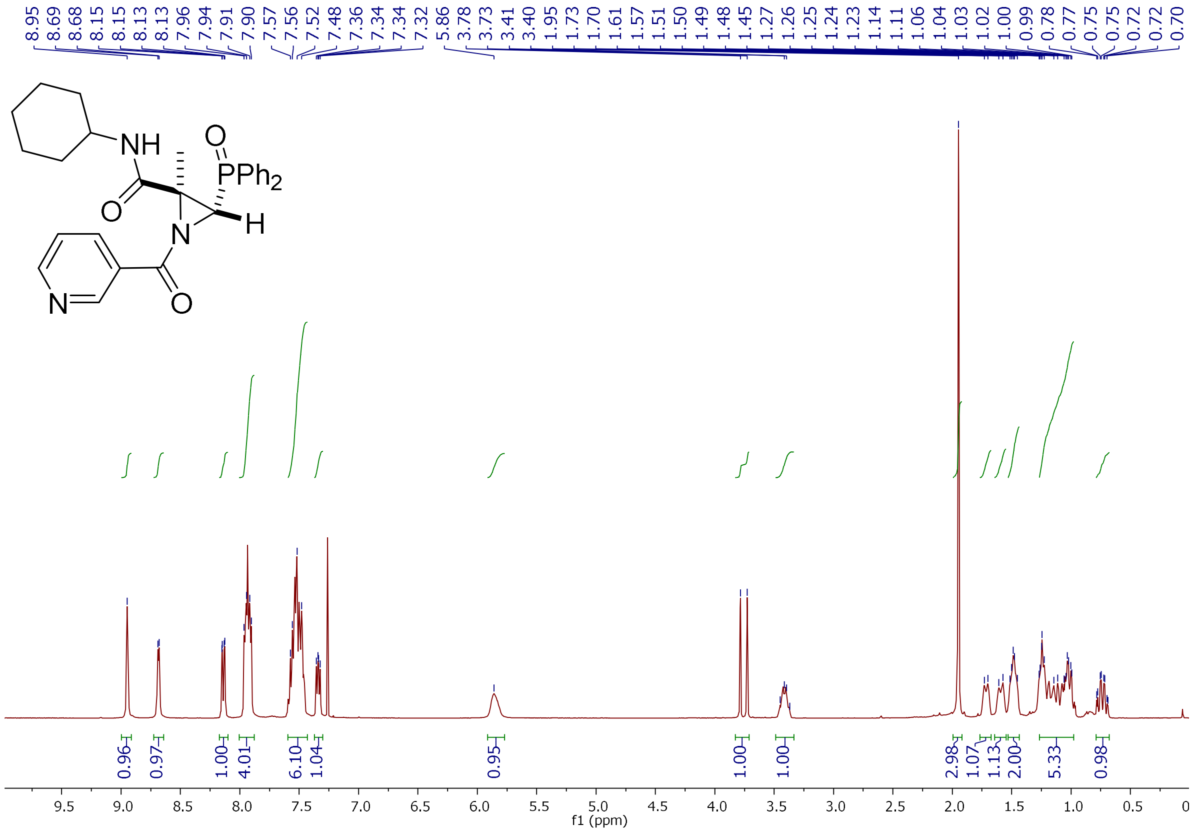

^{13}C {^{1}H} NMR (100 MHz, $CDCl_3$) of *N*-Acylaziridine Phosphine Oxide **4h**

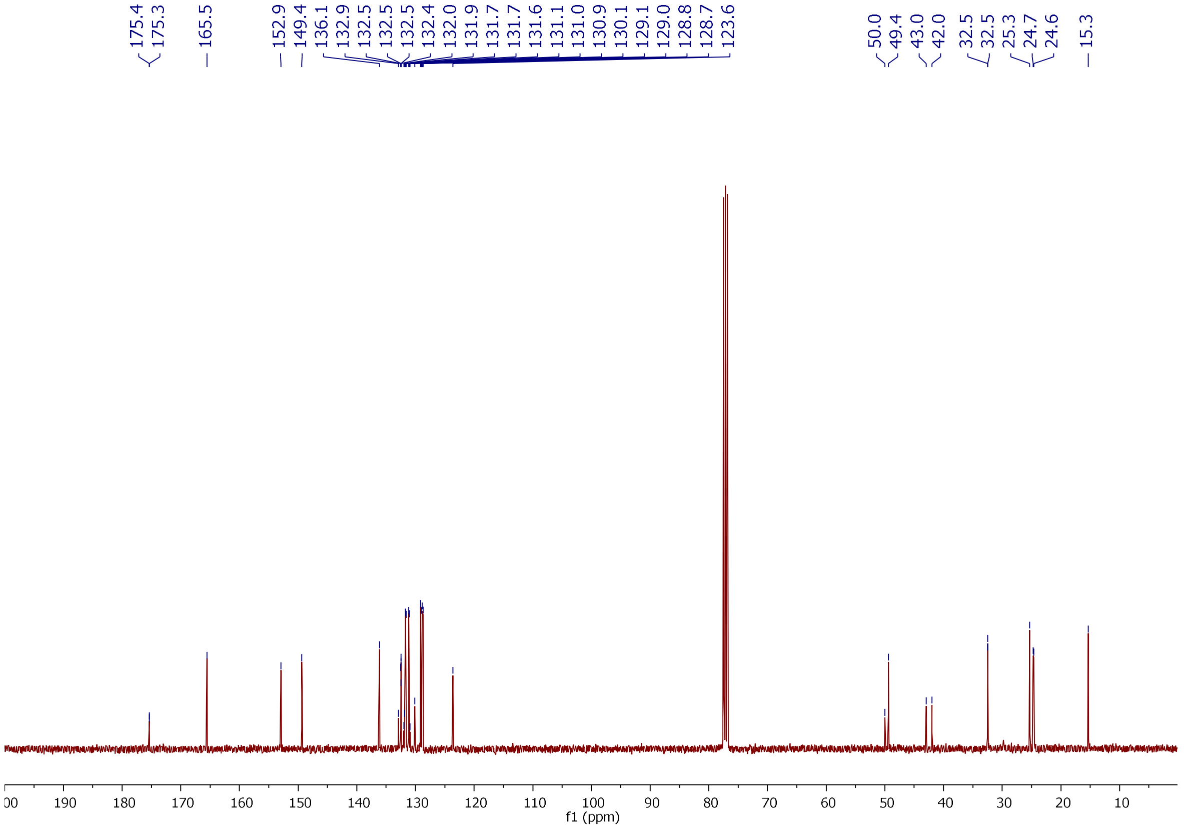

^{31}P (160 MHz, $CDCl_3$) of *N*-Acylaziridine Phosphine Oxide **4h**

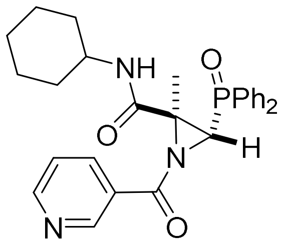

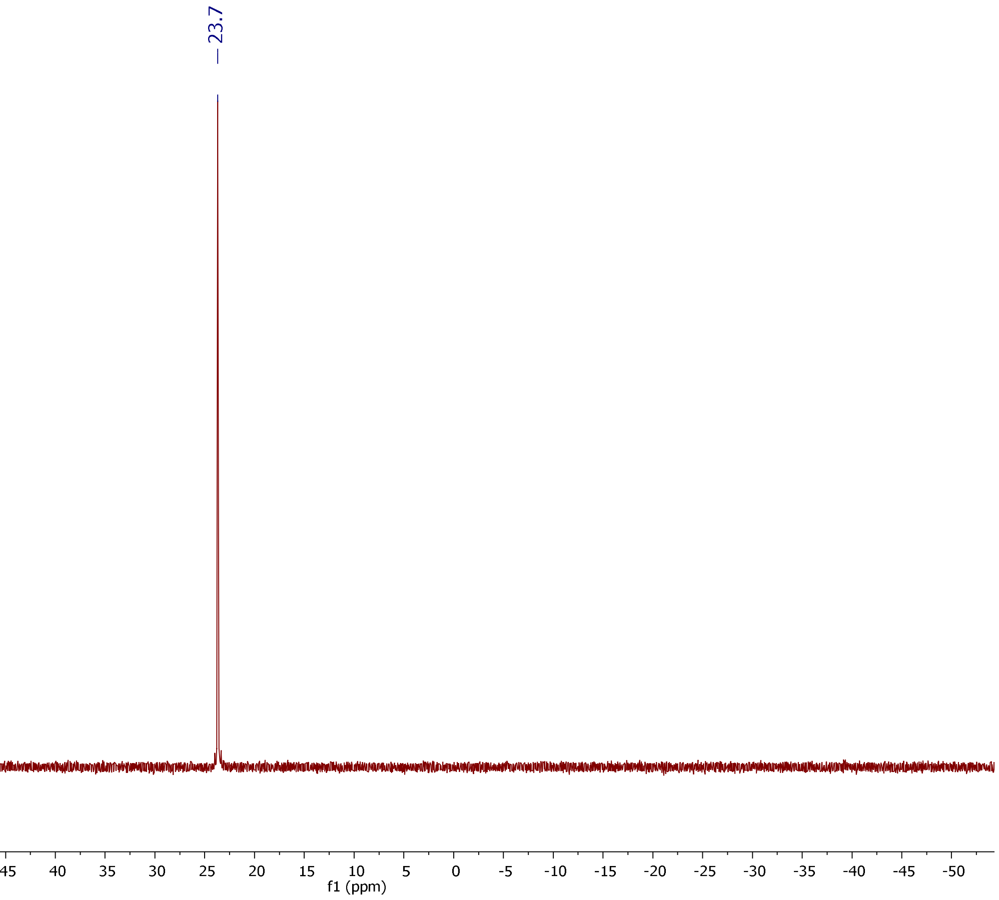

^{1}H NMR (400 MHz, $CDCl_3$) of *N*-Acylaziridine Phosphine Oxide **4i**

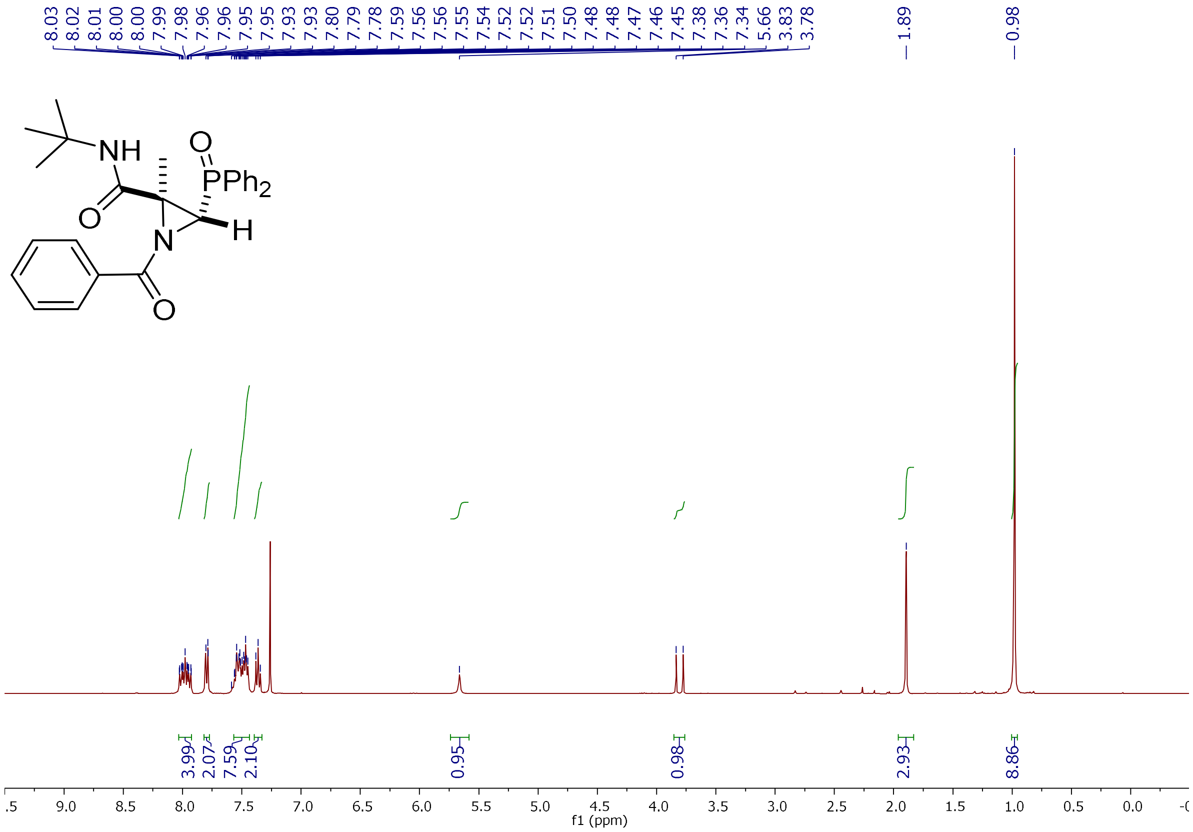

^{13}C {^{1}H} NMR (100 MHz, $CDCl_3$) of *N*-Acylaziridine Phosphine Oxide **4i**

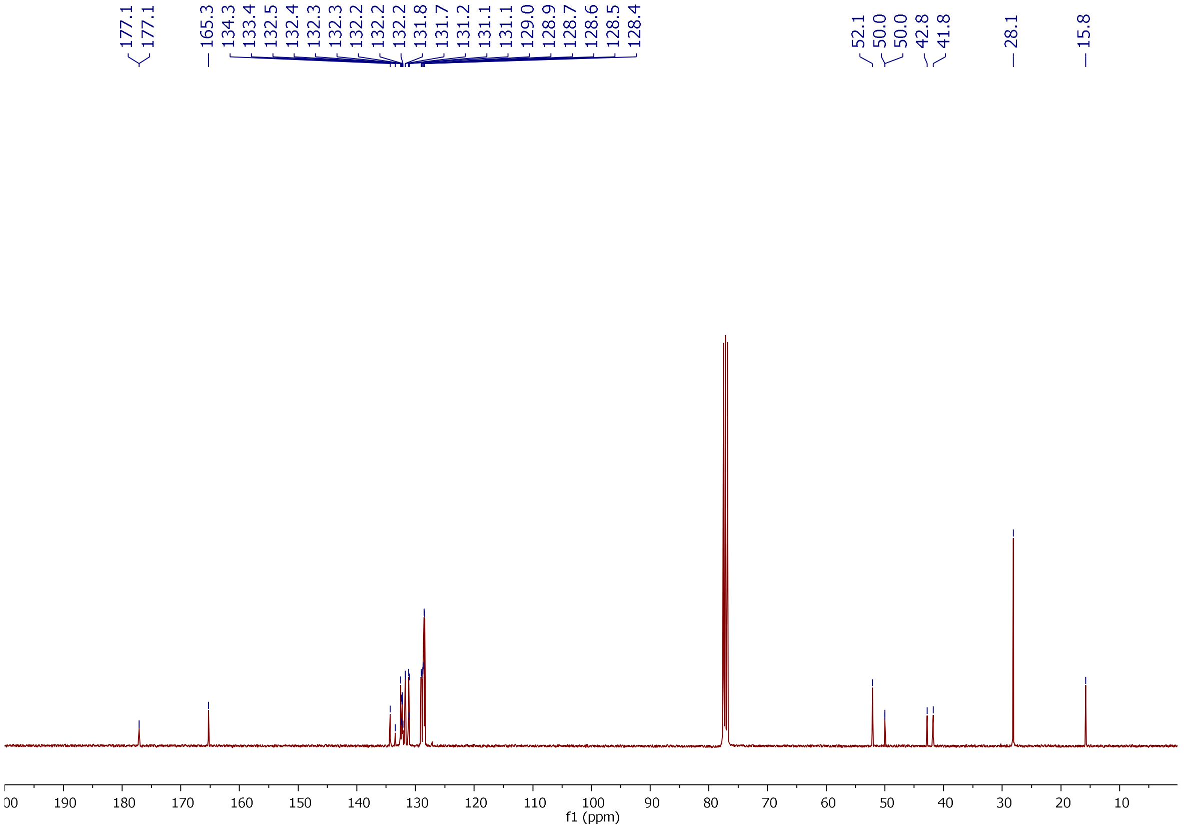

^{31}P (160 MHz, $CDCl_3$) of *N*-Acylaziridine Phosphine Oxide **4i**

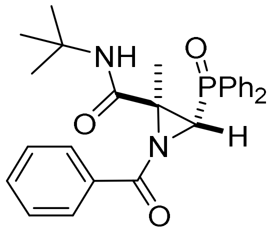

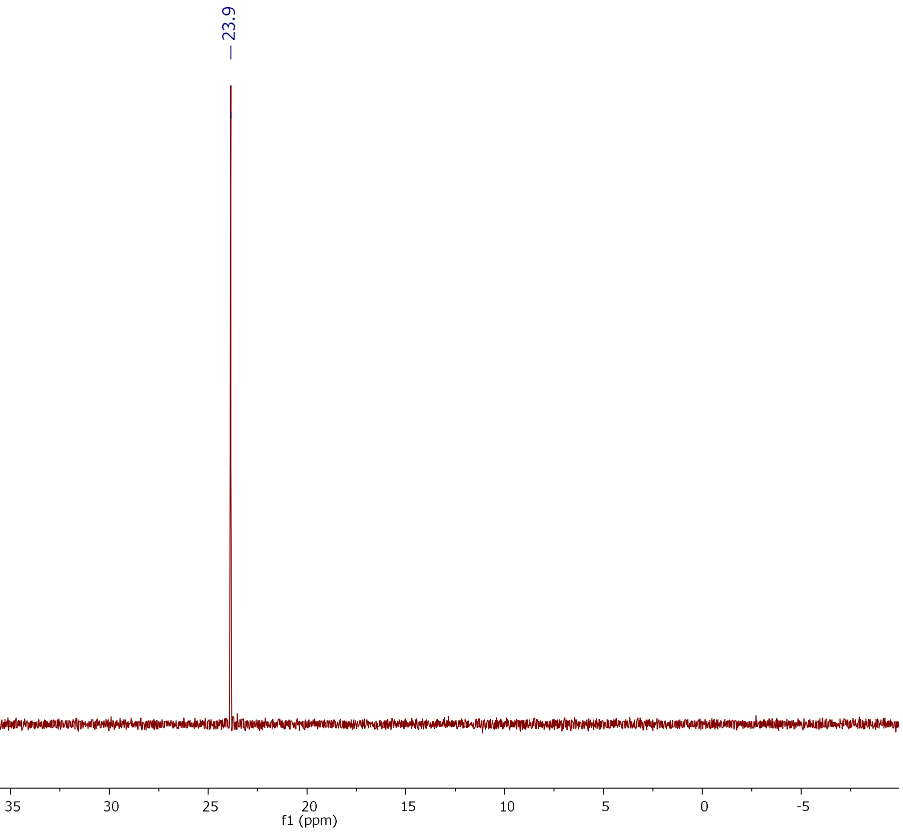

^{1}H NMR (400 MHz, $CDCl_3$) of *N*-Acylaziridine Phosphine Oxide **4j**

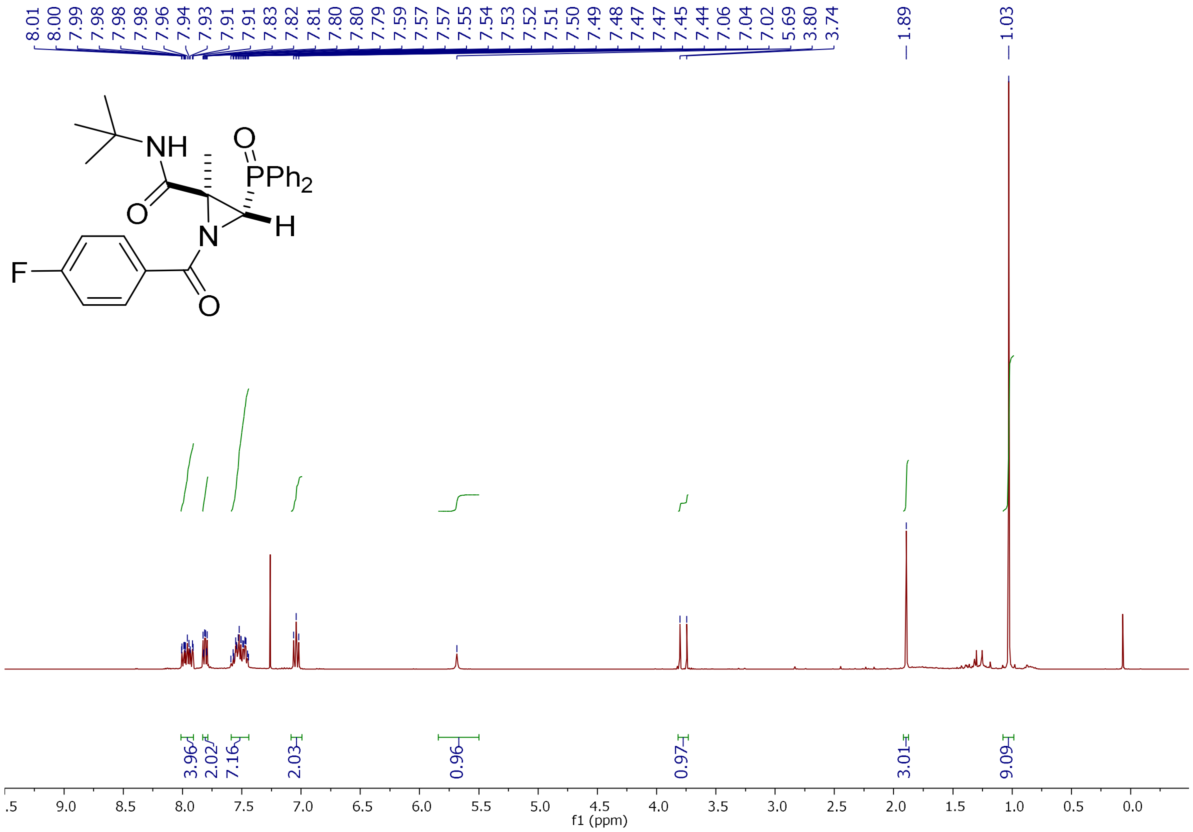

^{13}C {^{1}H} NMR (100 MHz, $CDCl_3$) of *N*-Acylaziridine Phosphine Oxide **4j**

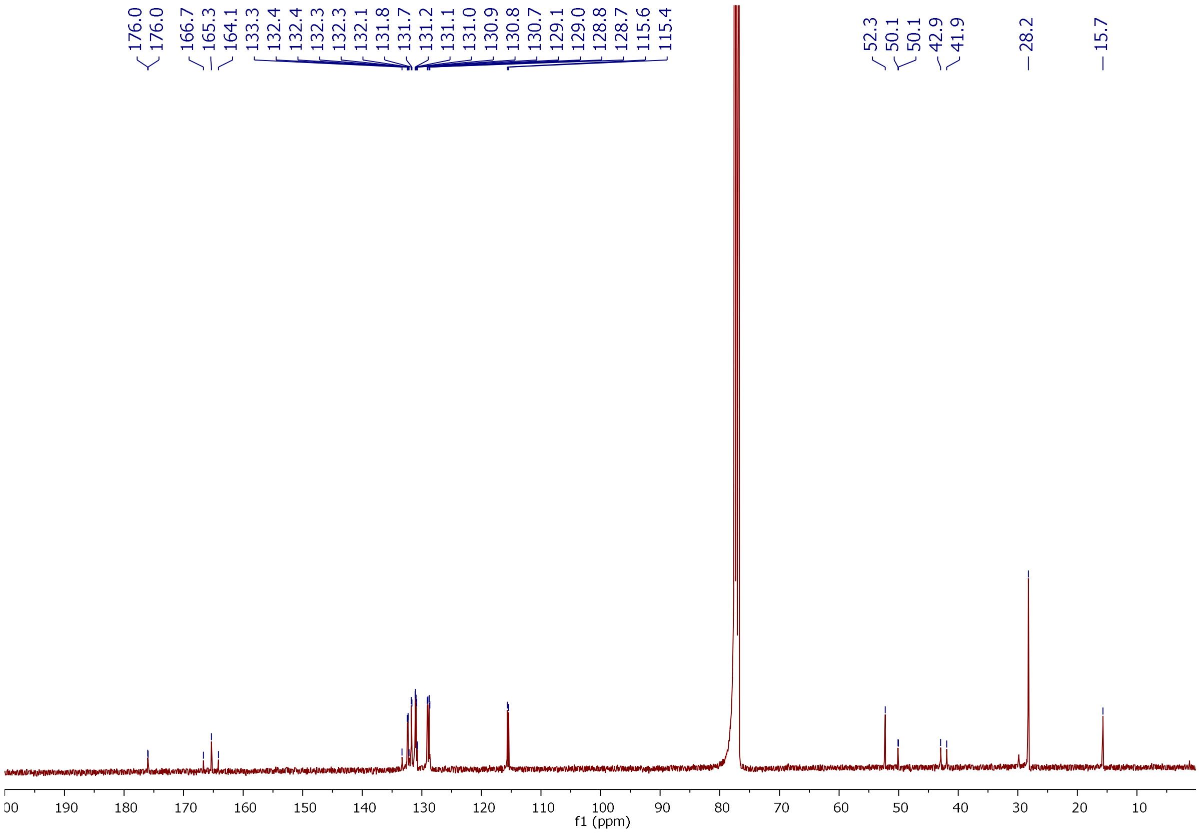

^{31}P (160 MHz, $CDCl_3$) of *N*-Acylaziridine Phosphine Oxide **4j**

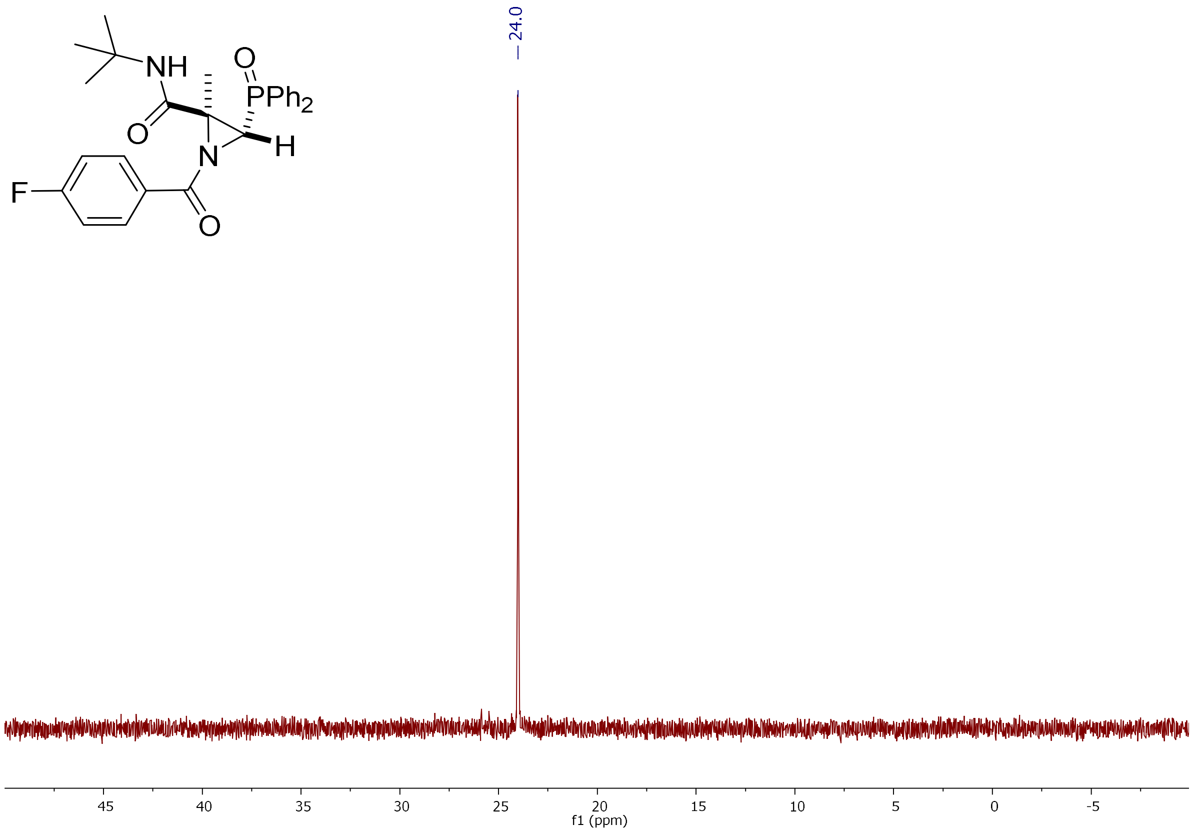

^{19}F (376 MHz, $CDCl_3$) of *N*-Acylaziridine Phosphine Oxide **4j**

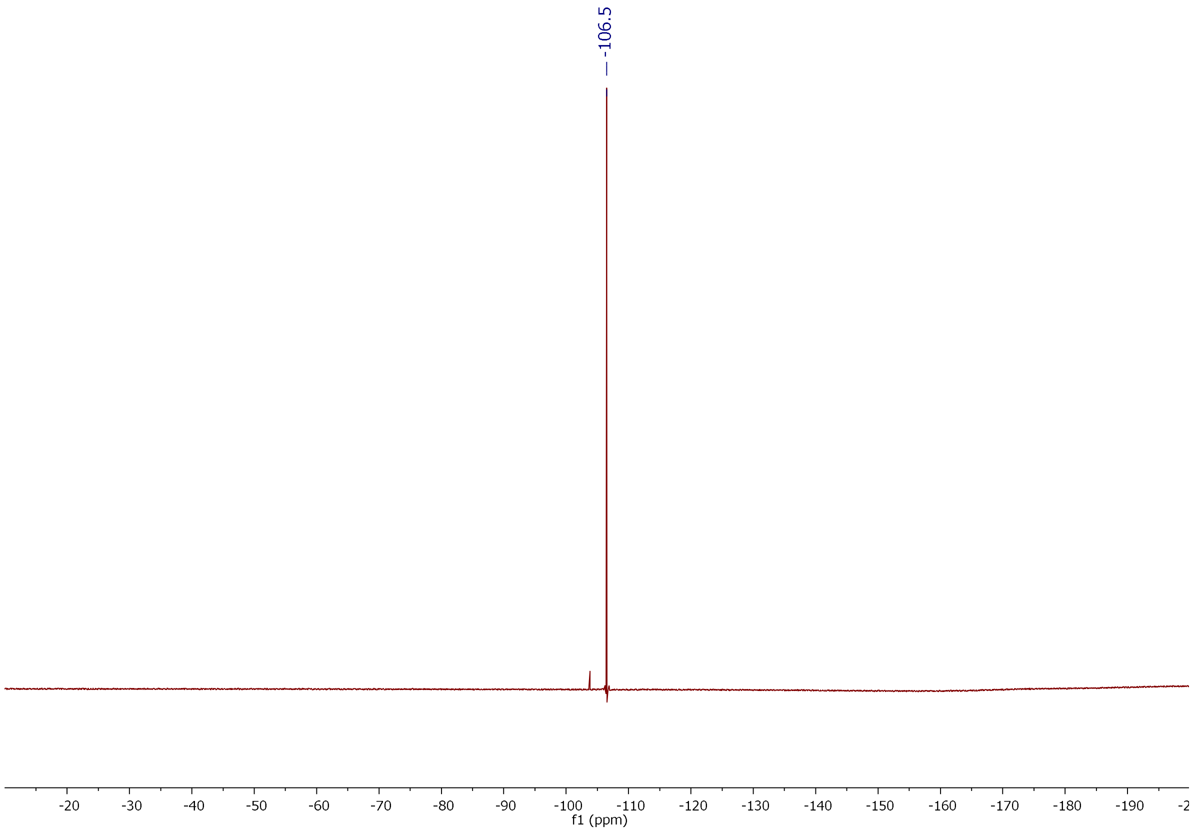

^{1}H NMR (400 MHz, $CDCl_3$) of *N*-Acylaziridine Phosphine Oxide **4k**

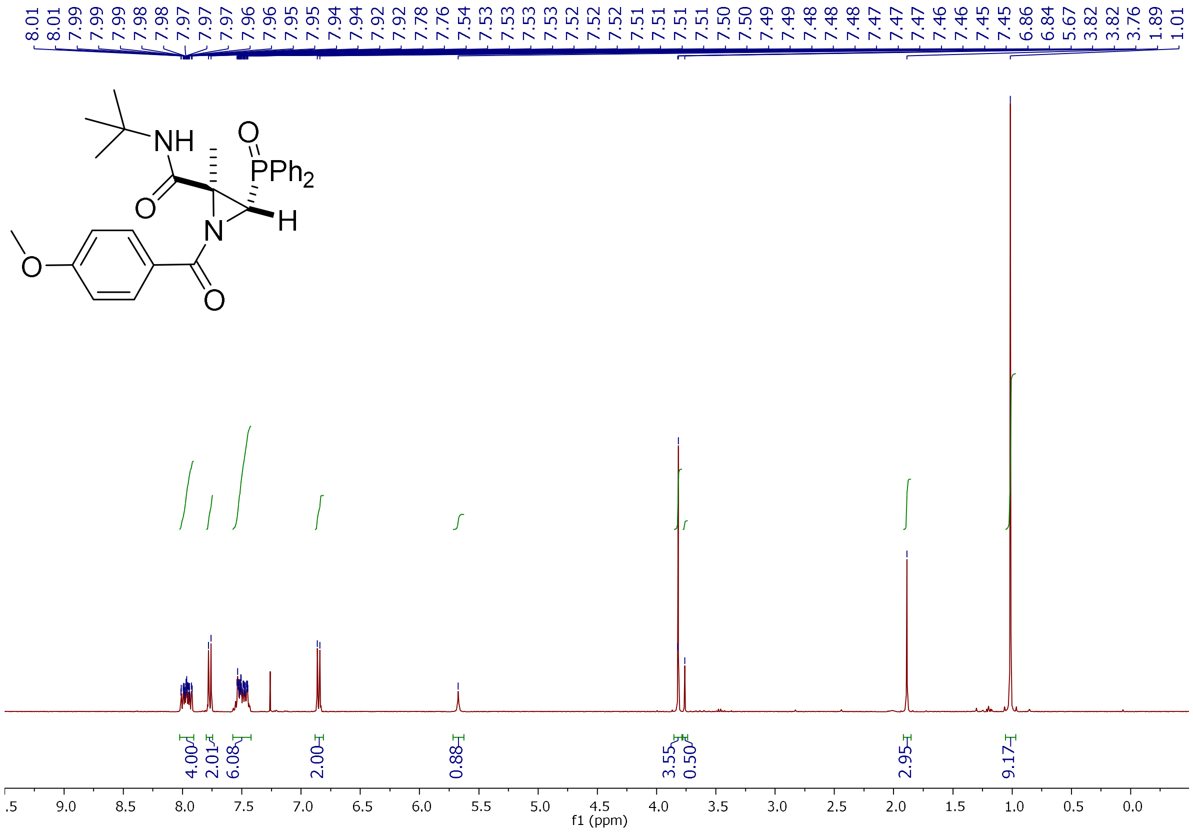

^{13}C {^{1}H} NMR (100 MHz, $CDCl_3$) of *N*-Acylaziridine Phosphine Oxide **4k**

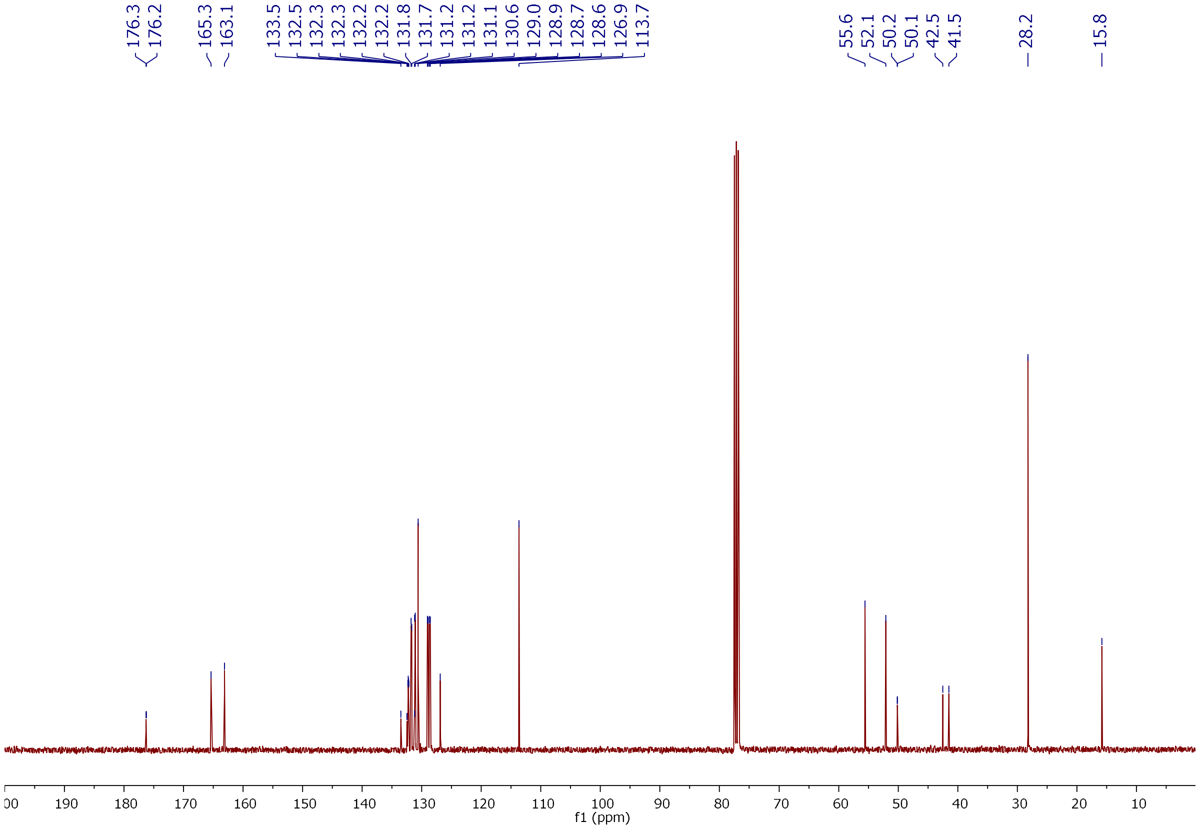

^{31}P (160 MHz, $CDCl_3$) of *N*-Acylaziridine Phosphine Oxide **4k**

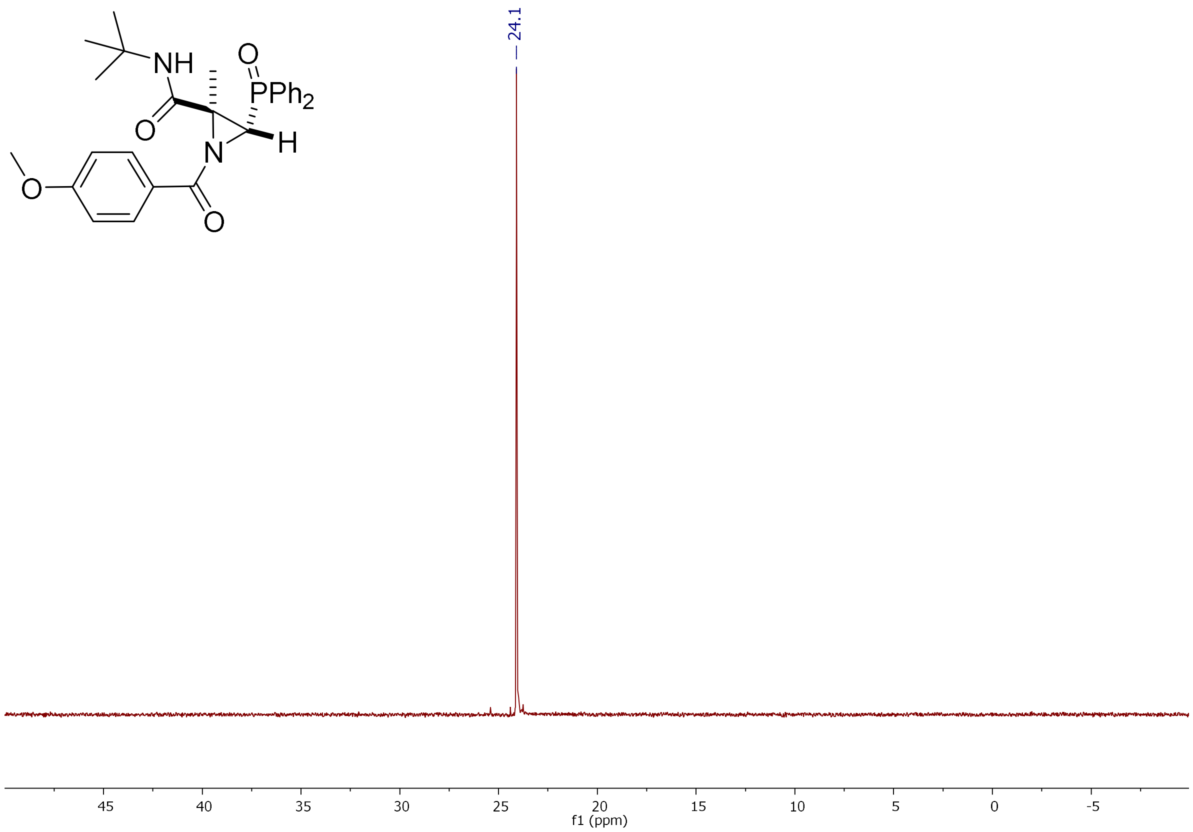

^{1}H NMR (400 MHz, $CDCl_3$) of *N*-Acylaziridine Phosphine Oxide **4l**

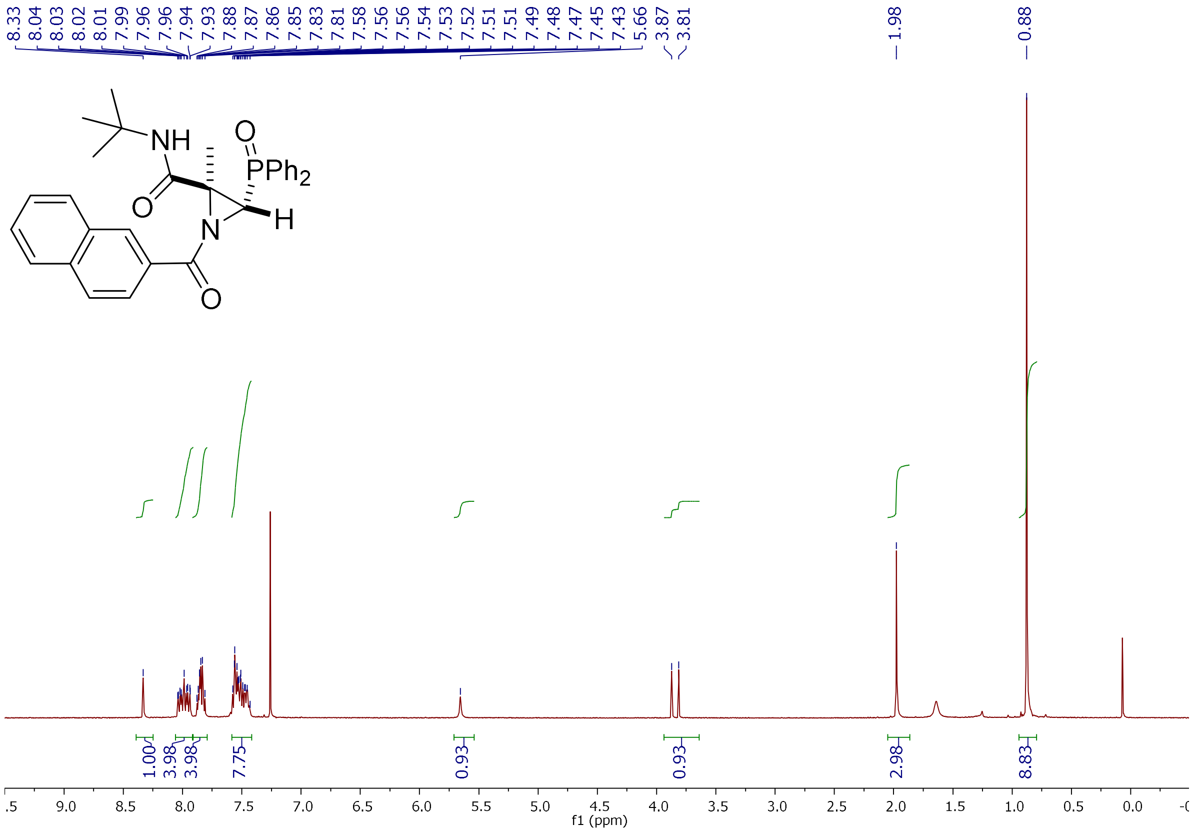

^{13}C {^{1}H} NMR (100 MHz, $CDCl_3$) of *N*-Acylaziridine Phosphine Oxide **4l**

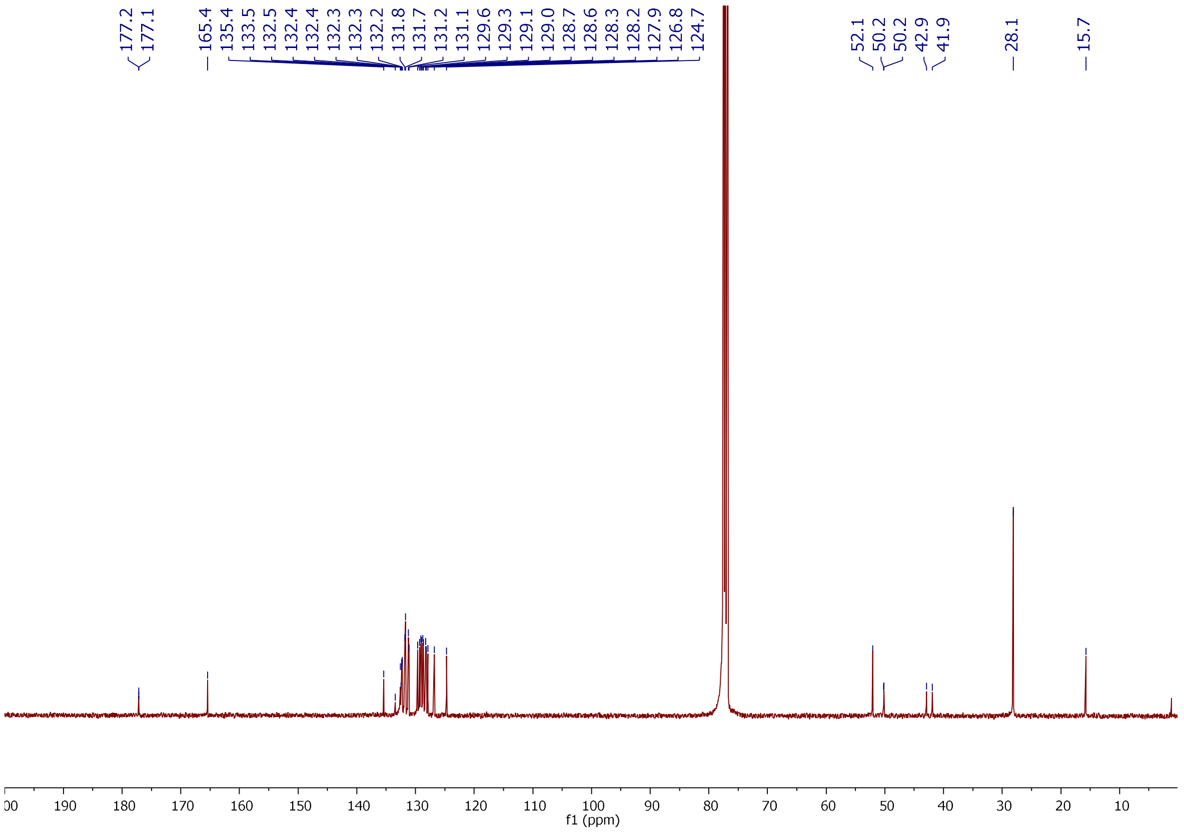

^{31}P (160 MHz, $CDCl_3$) of *N*-Acylaziridine Phosphine Oxide **4l**

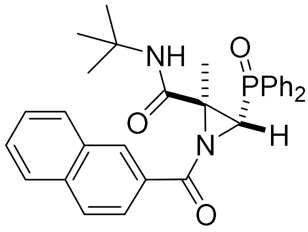

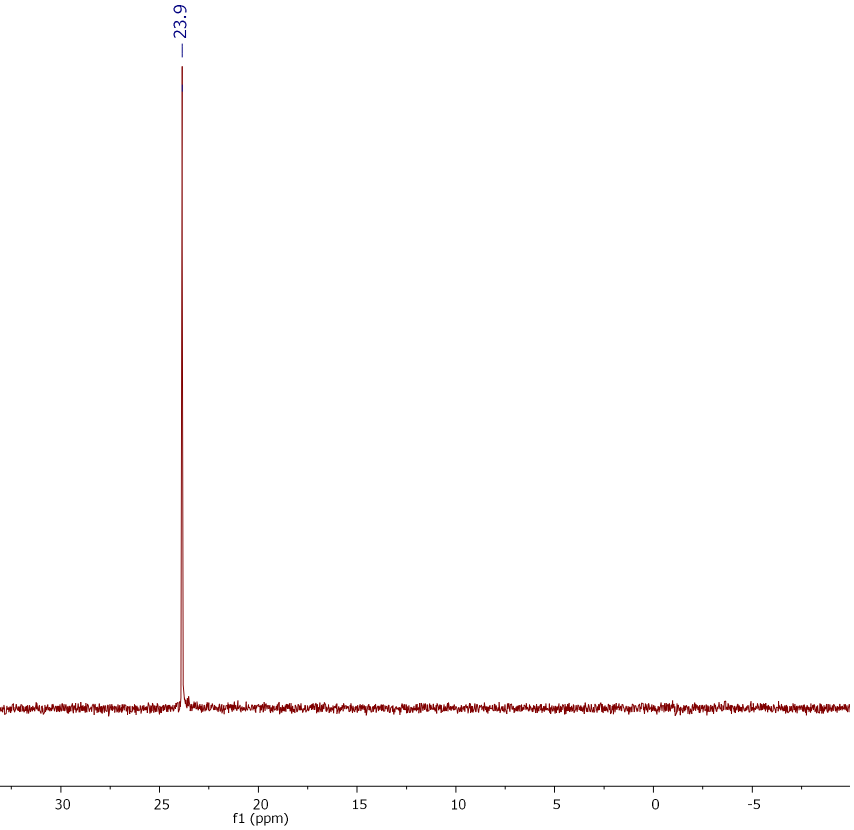

^{1}H NMR (400 MHz, $CDCl_3$) of *N*-Acylaziridine Phosphine Oxide **4m**

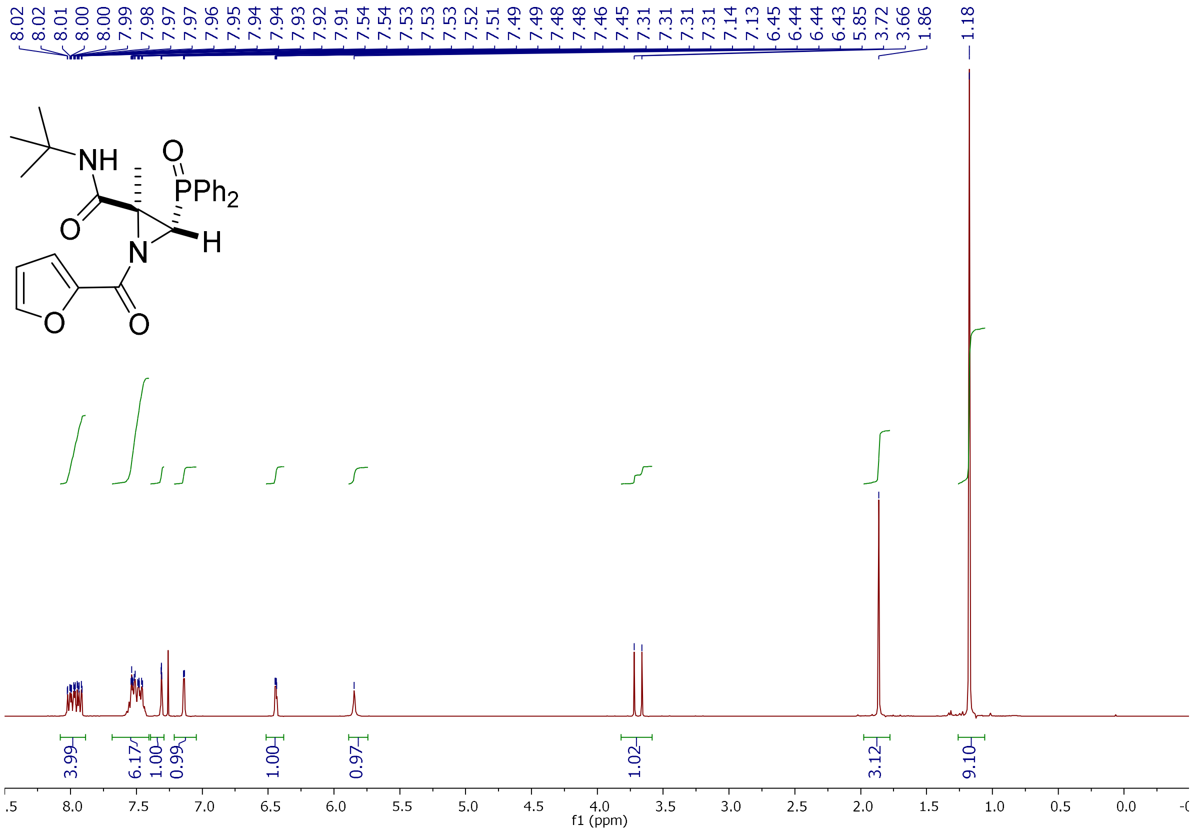

^{13}C {^{1}H} NMR (100 MHz, $CDCl_3$) of *N*-Acylaziridine Phosphine Oxide **4m**

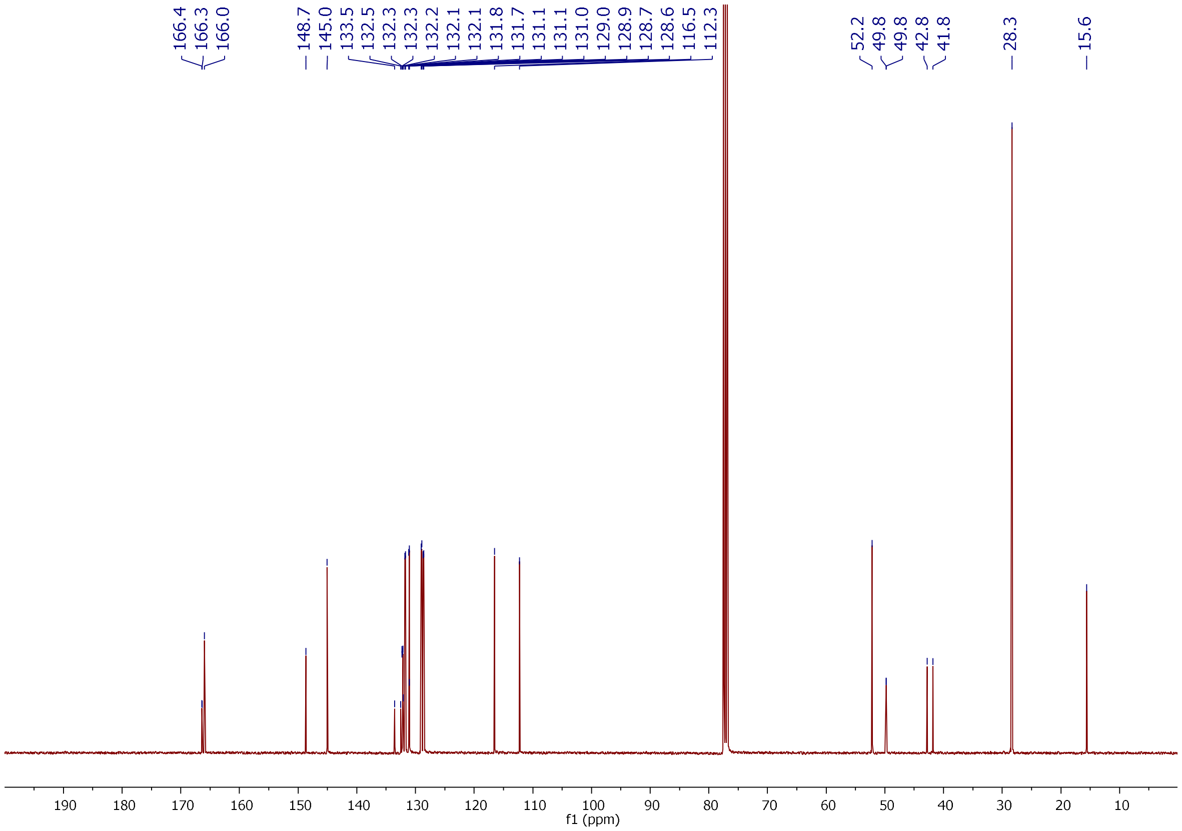

^{31}P (160 MHz, $CDCl_3$) of *N*-Acylaziridine Phosphine Oxide **4m**

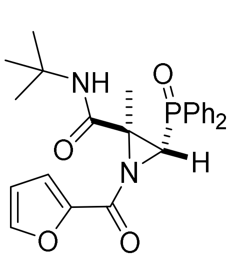

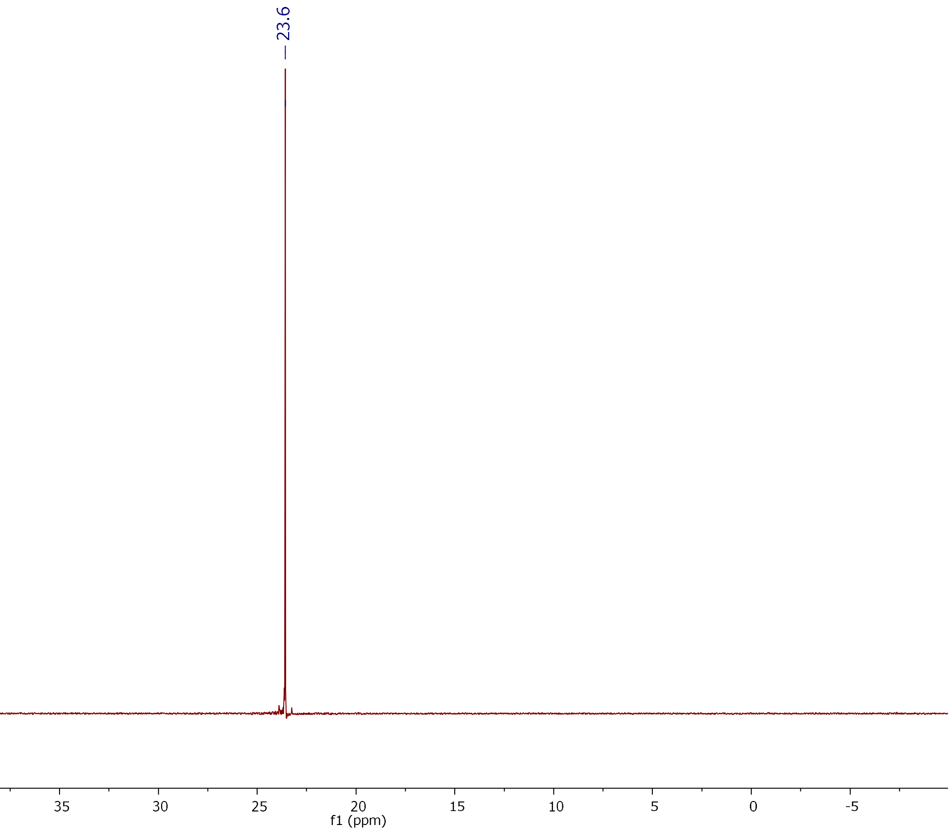

^{1}H NMR (400 MHz, $CDCl_3$) of *N*-Acylaziridine Phosphine Oxide **4n**

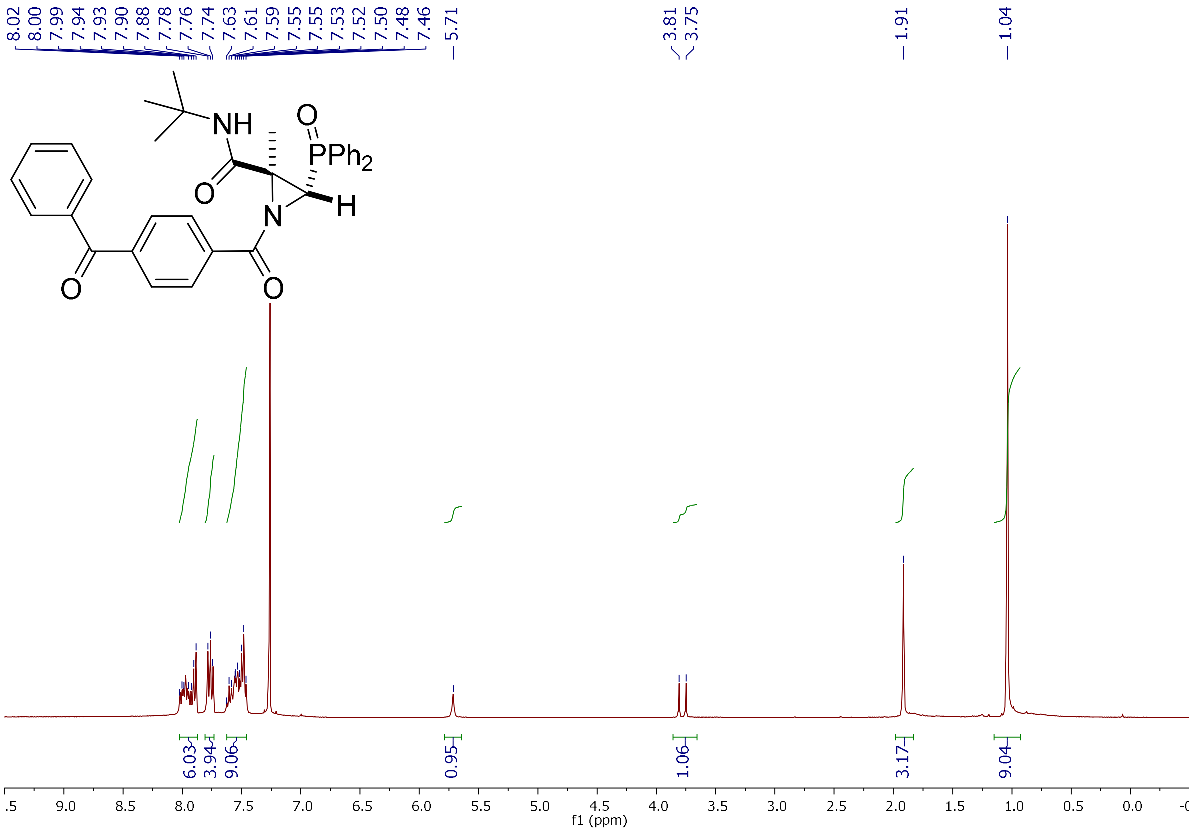

^{13}C {^{1}H} NMR (100 MHz, $CDCl_3$) of *N*-Acylaziridine Phosphine Oxide **4n**

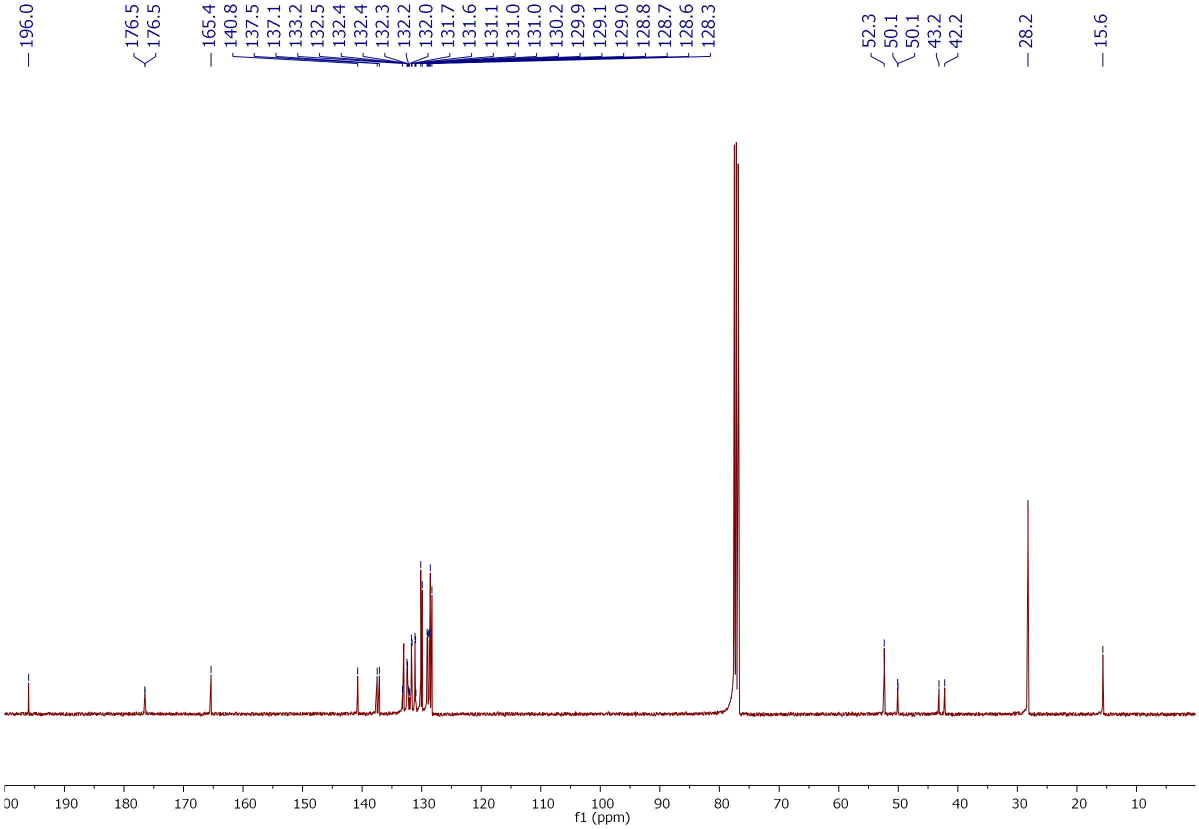

^{31}P (160 MHz, $CDCl_3$) of *N*-Acylaziridine Phosphine Oxide **4n**

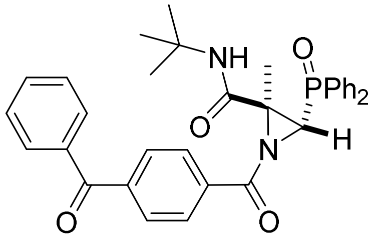

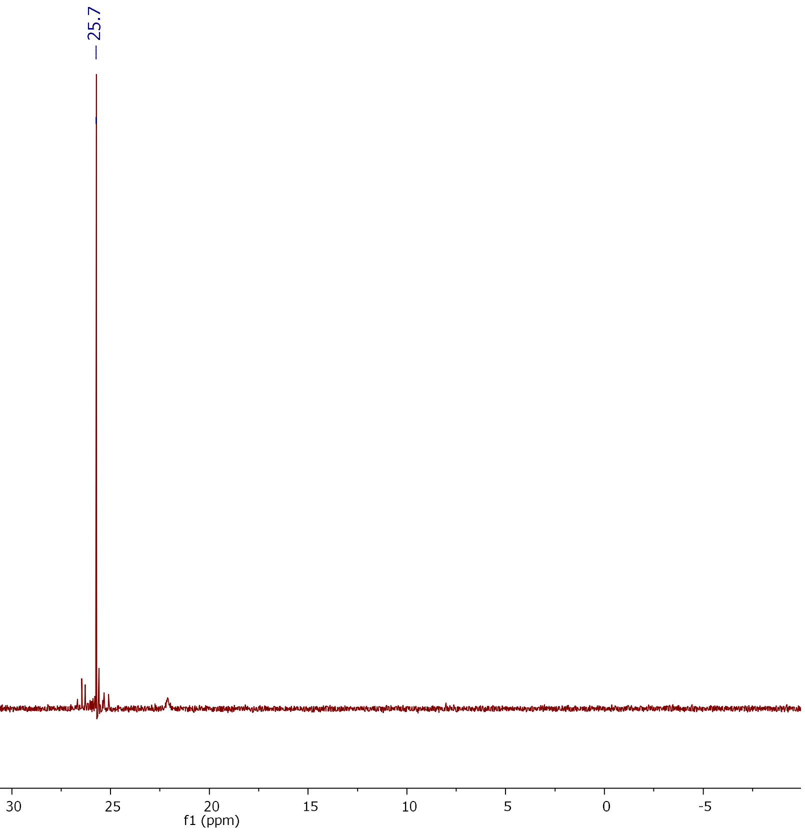

^{1}H NMR (400 MHz, $CDCl_3$) of *N*-Acylaziridine Phosphine Oxide **4o**

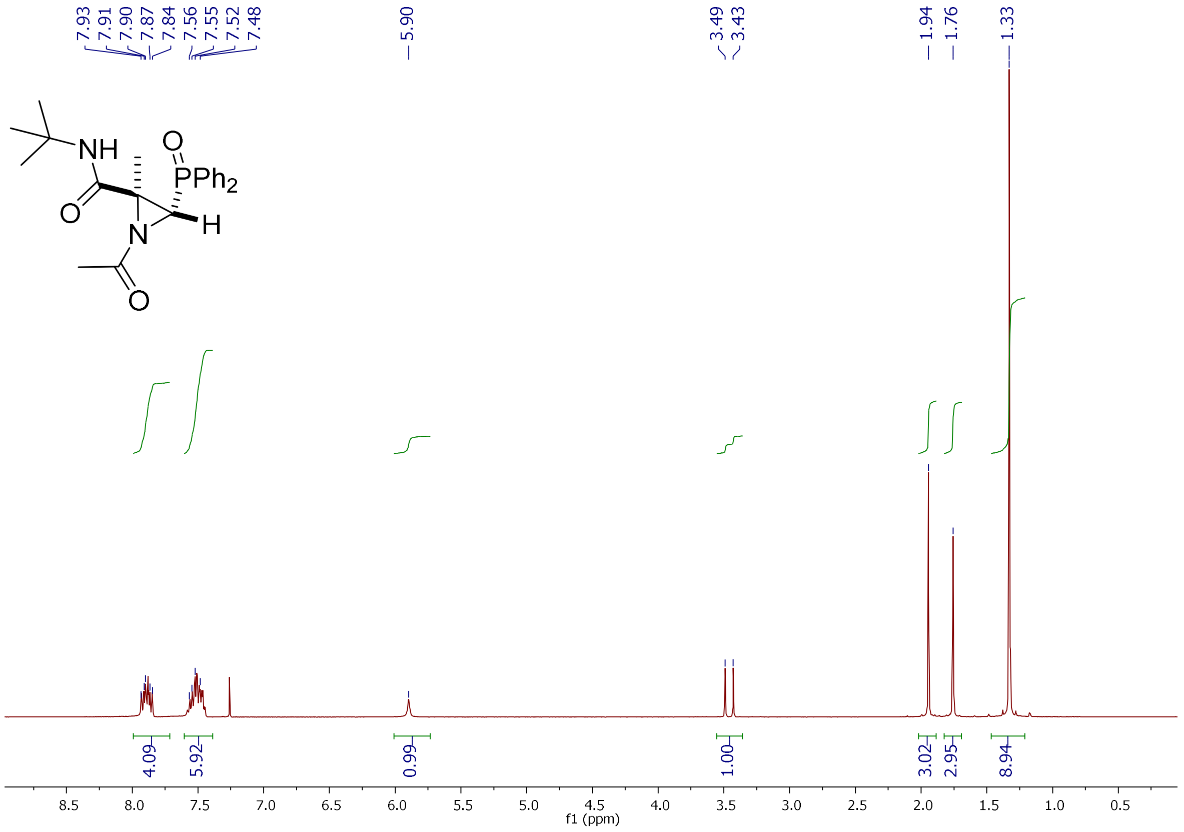

^{13}C {^{1}H} NMR (100 MHz, $CDCl_3$) of *N*-Acylaziridine Phosphine Oxide **4o**

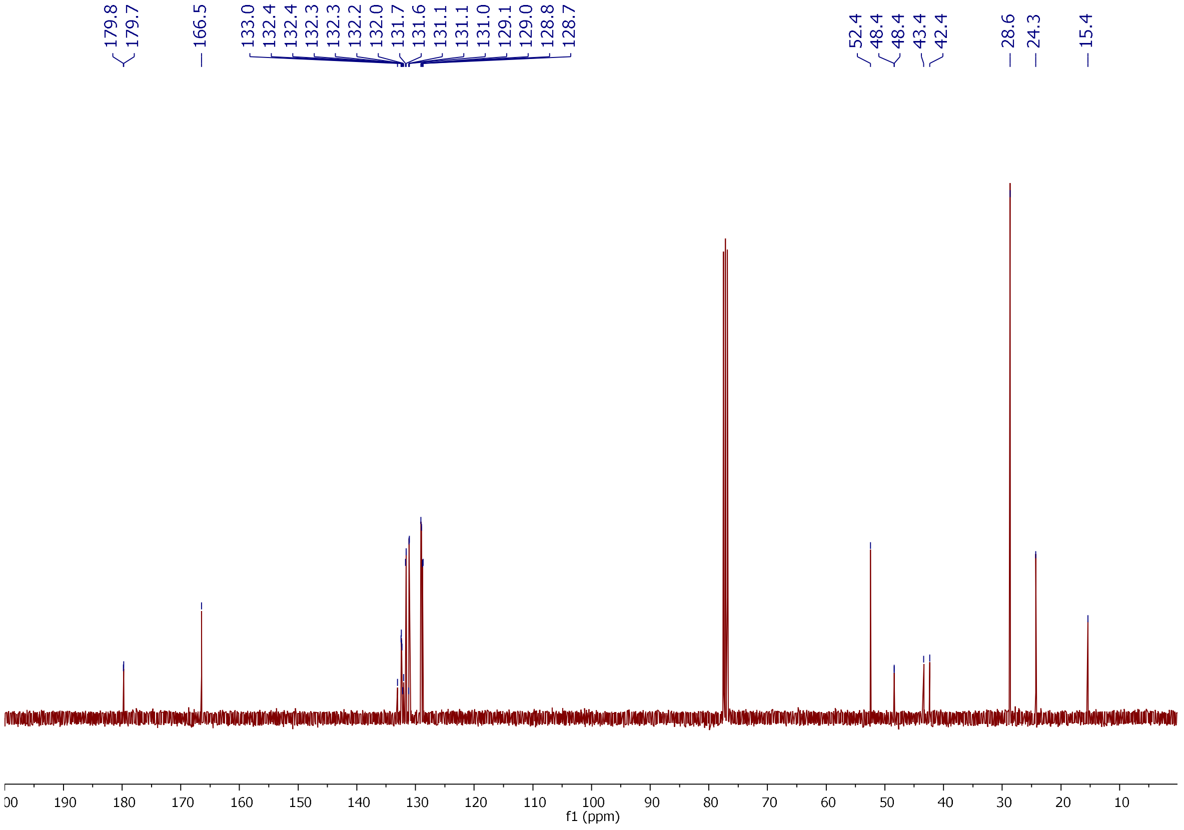

^{31}P (160 MHz, $CDCl_3$) of *N*-Acylaziridine Phosphine Oxide **4o**

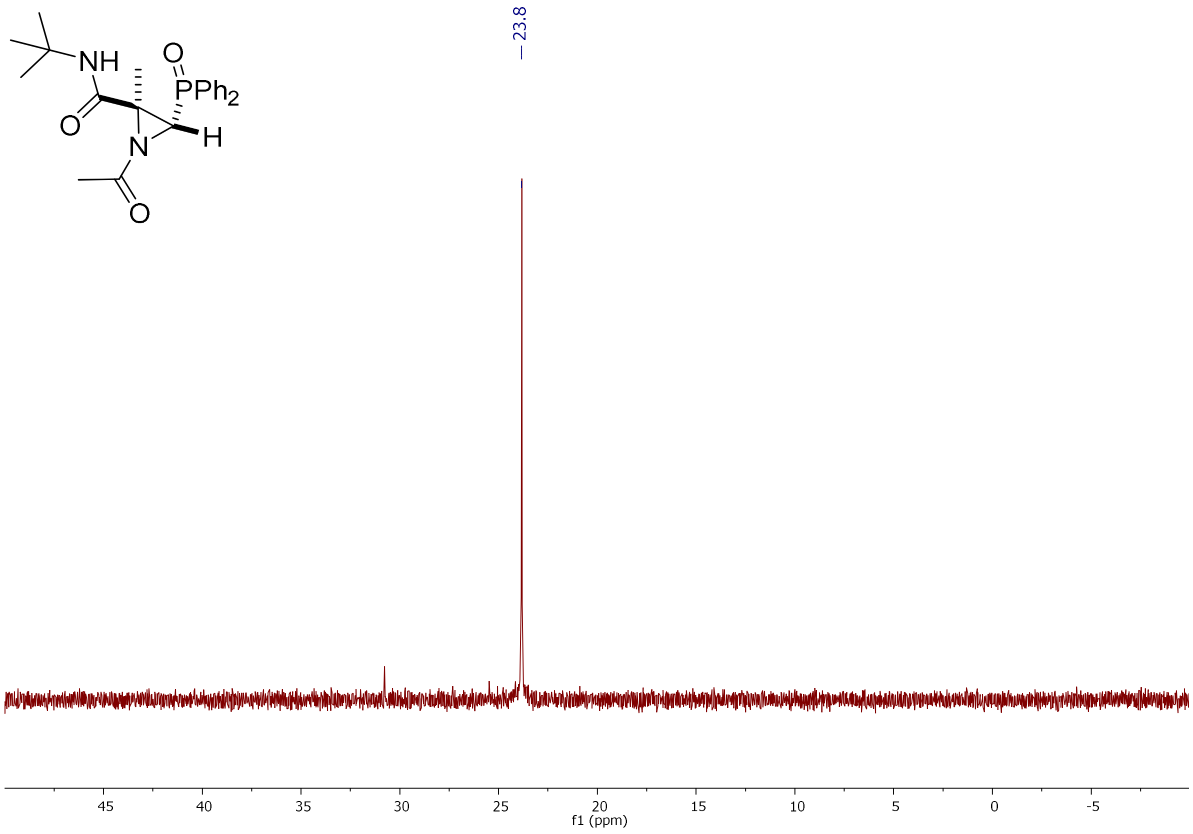

^{1}H NMR (400 MHz, $CDCl_3$) of *N*-Acylaziridine Phosphine Oxide **4p**

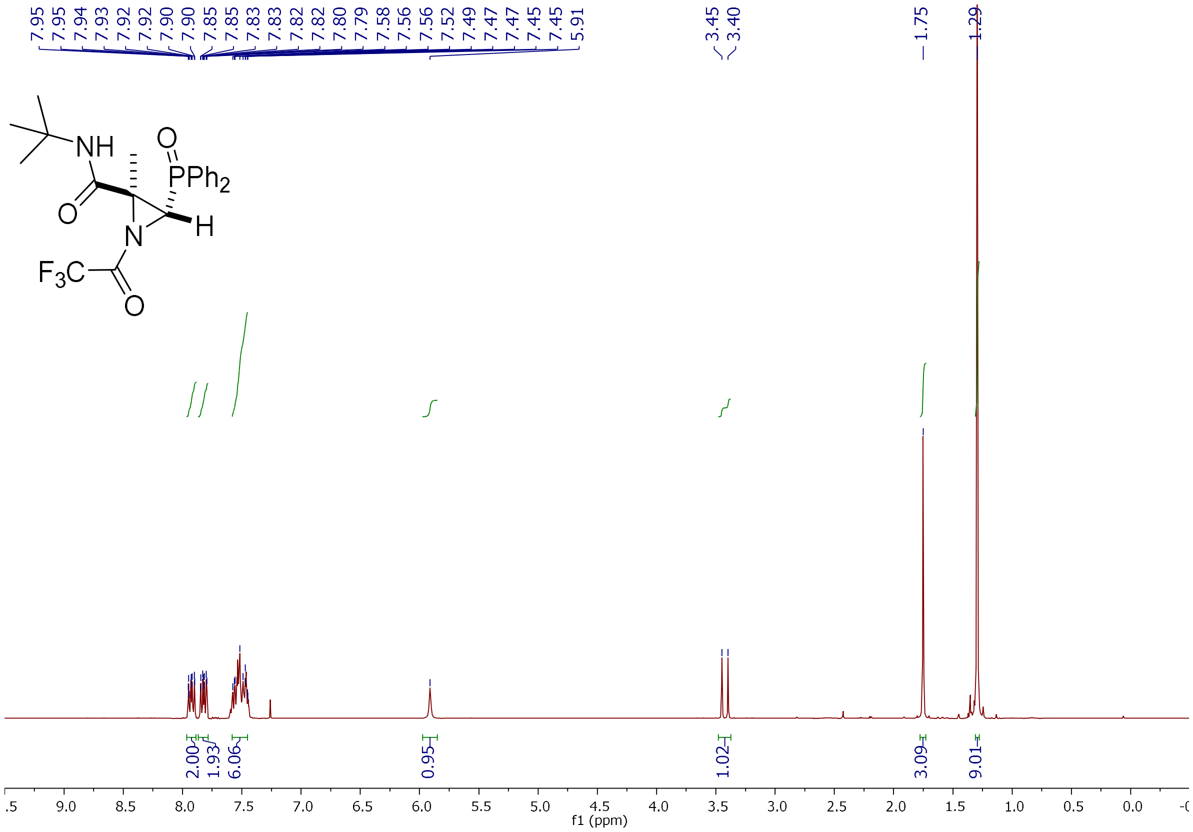

^{13}C {^{1}H} NMR (100 MHz, $CDCl_3$) of *N*-Acylaziridine Phosphine Oxide **4p**

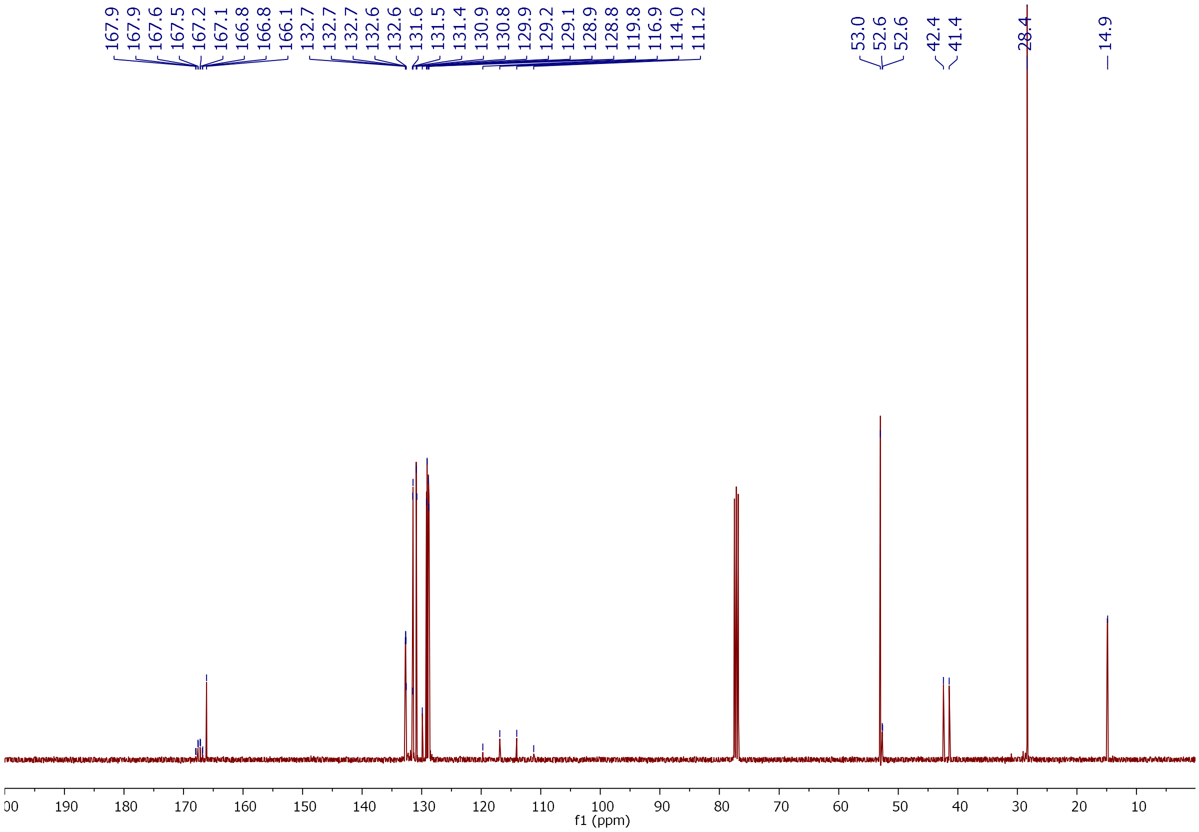

^{31}P (160 MHz, $CDCl_3$) of *N*-Acylaziridine Phosphine Oxide **4p**

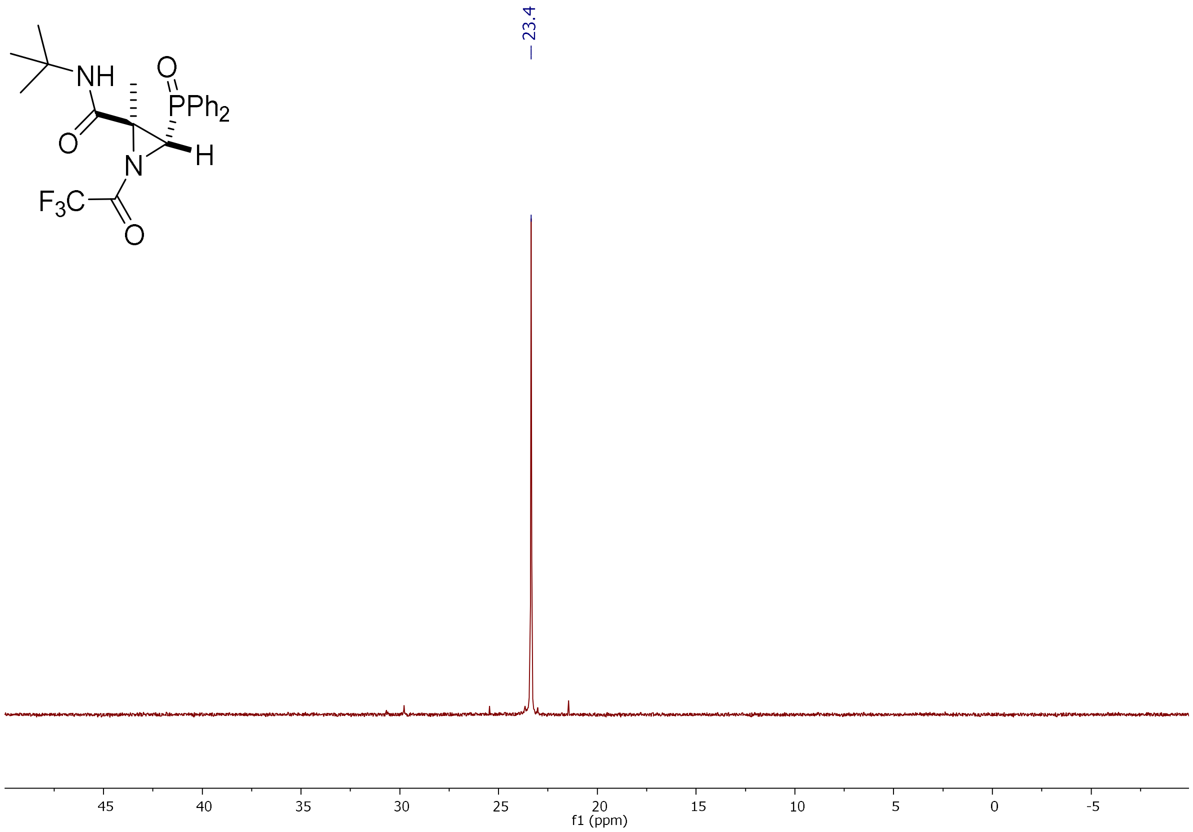

^{19}F (376 MHz, $CDCl_3$) of *N*-Acylaziridine Phosphine Oxide **4p**

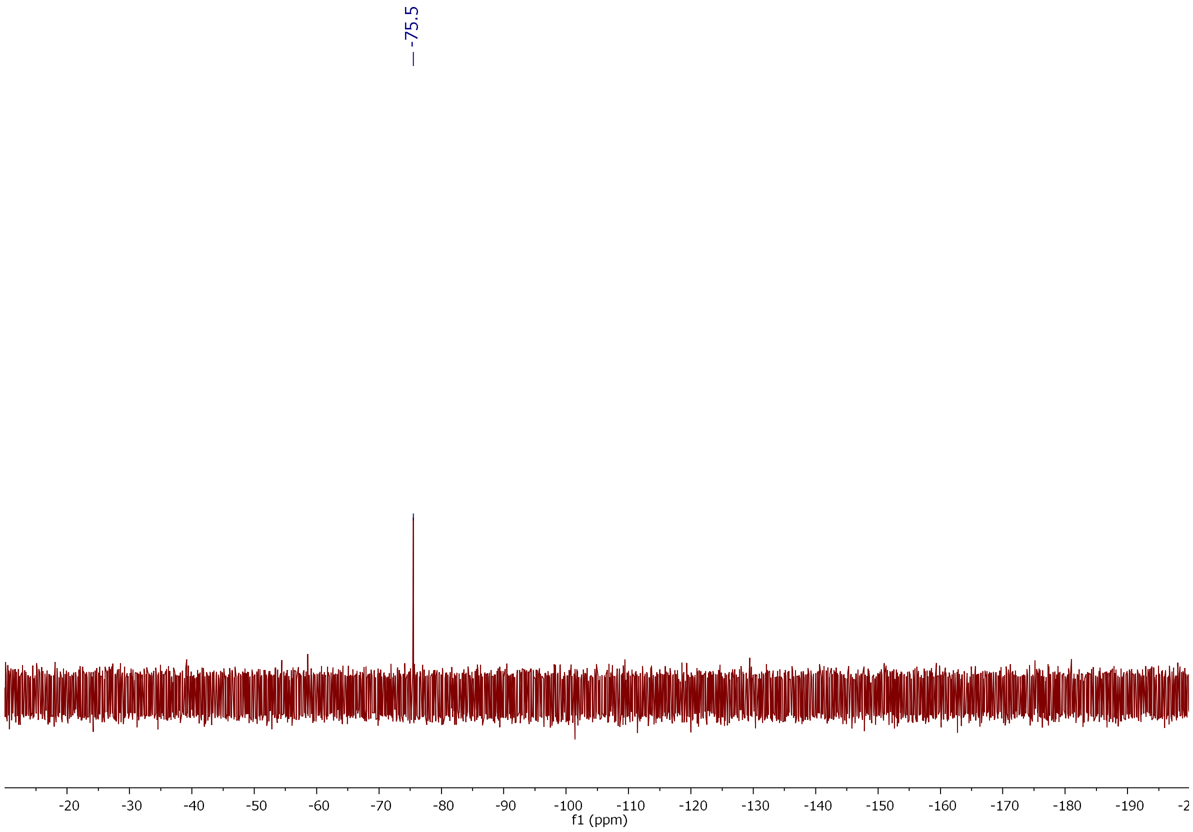

^{1}H NMR (400 MHz, $CDCl_3$) of *N*-Acylaziridine Phosphine Oxide **4q**

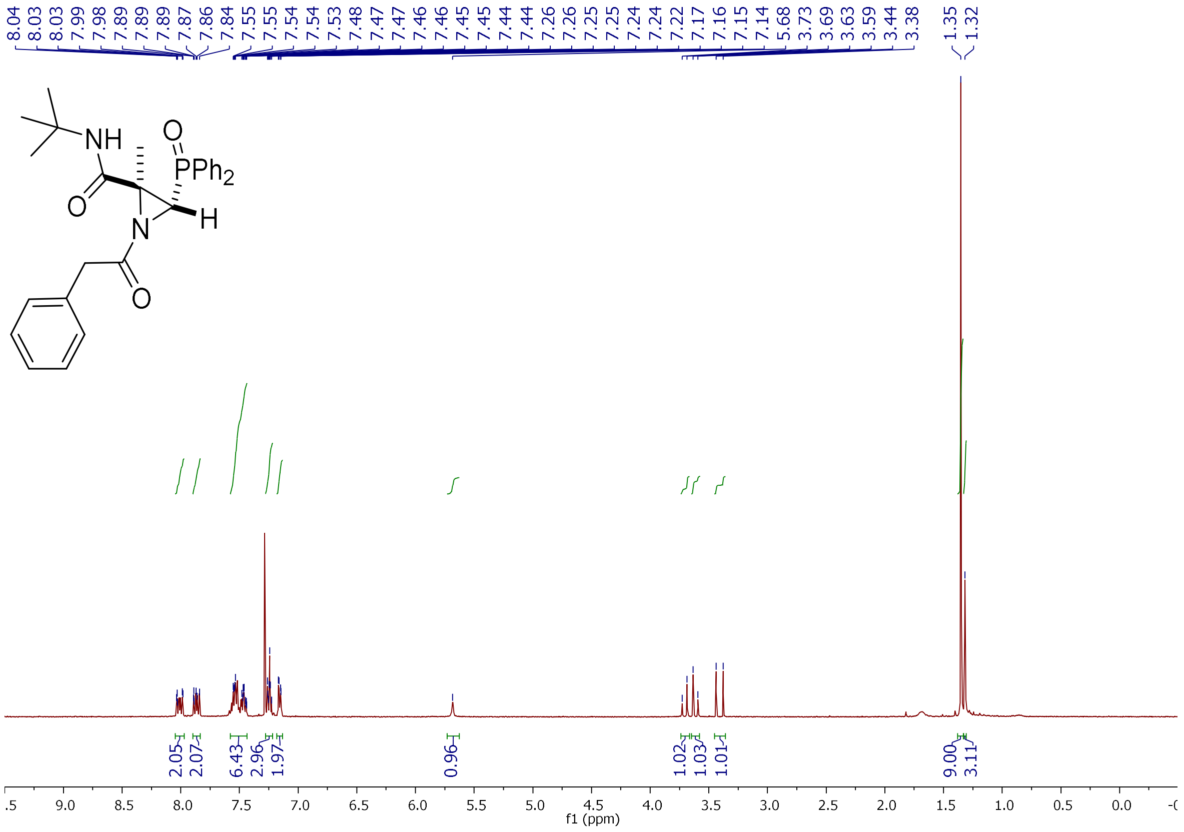

^{13}C {^{1}H} NMR (100 MHz, $CDCl_3$) of *N*-Acylaziridine Phosphine Oxide **4q**

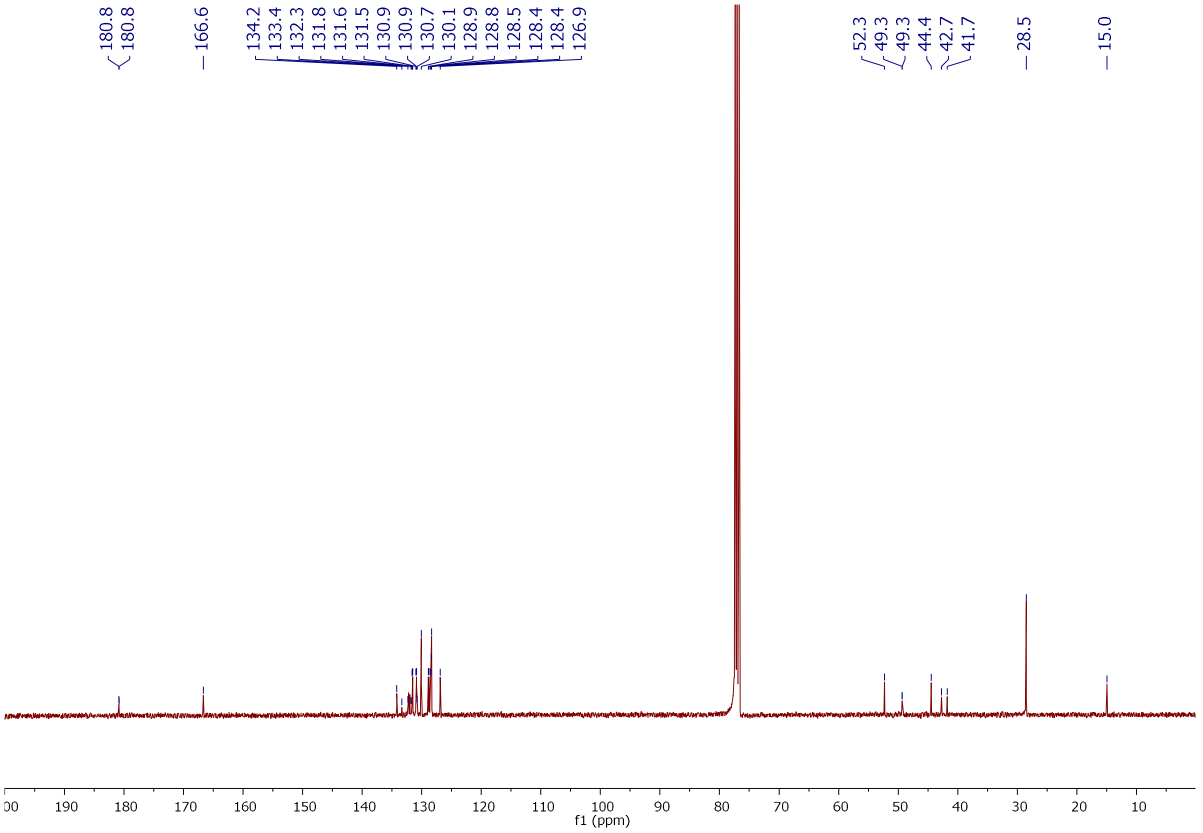

^{31}P (160 MHz, $CDCl_3$) of *N*-Acylaziridine Phosphine Oxide **4q**

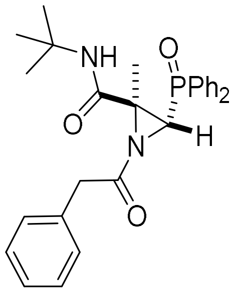

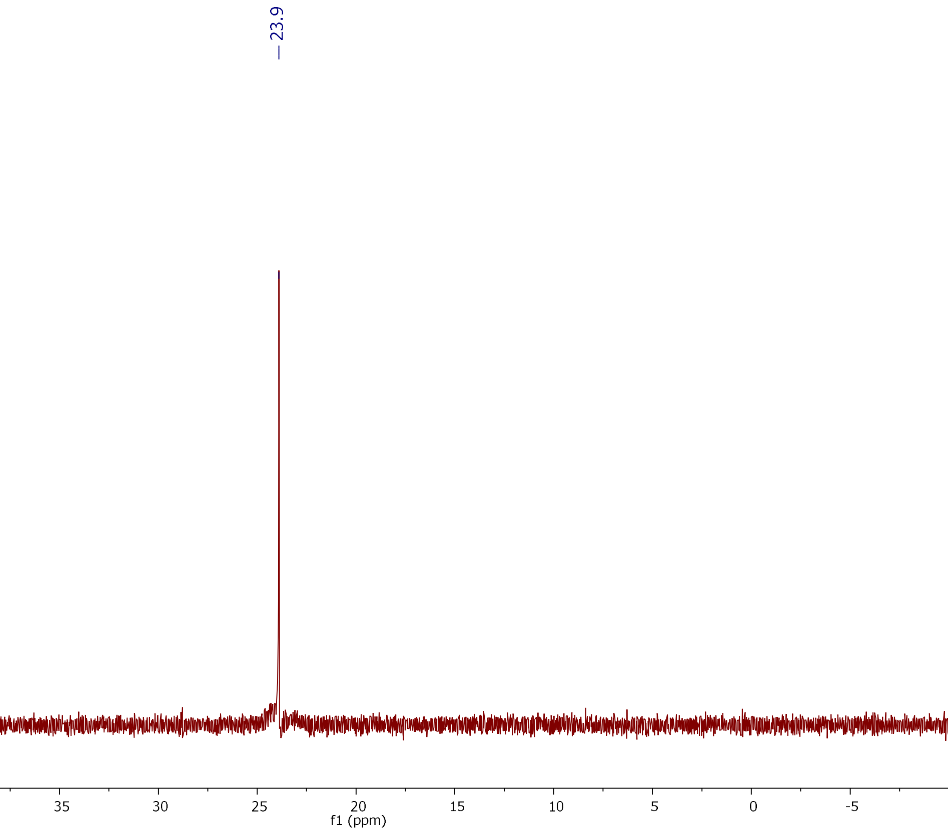

^{1}H NMR (400 MHz, $CDCl_3$) of *N*-Acylaziridine Phosphine Oxide **4r**

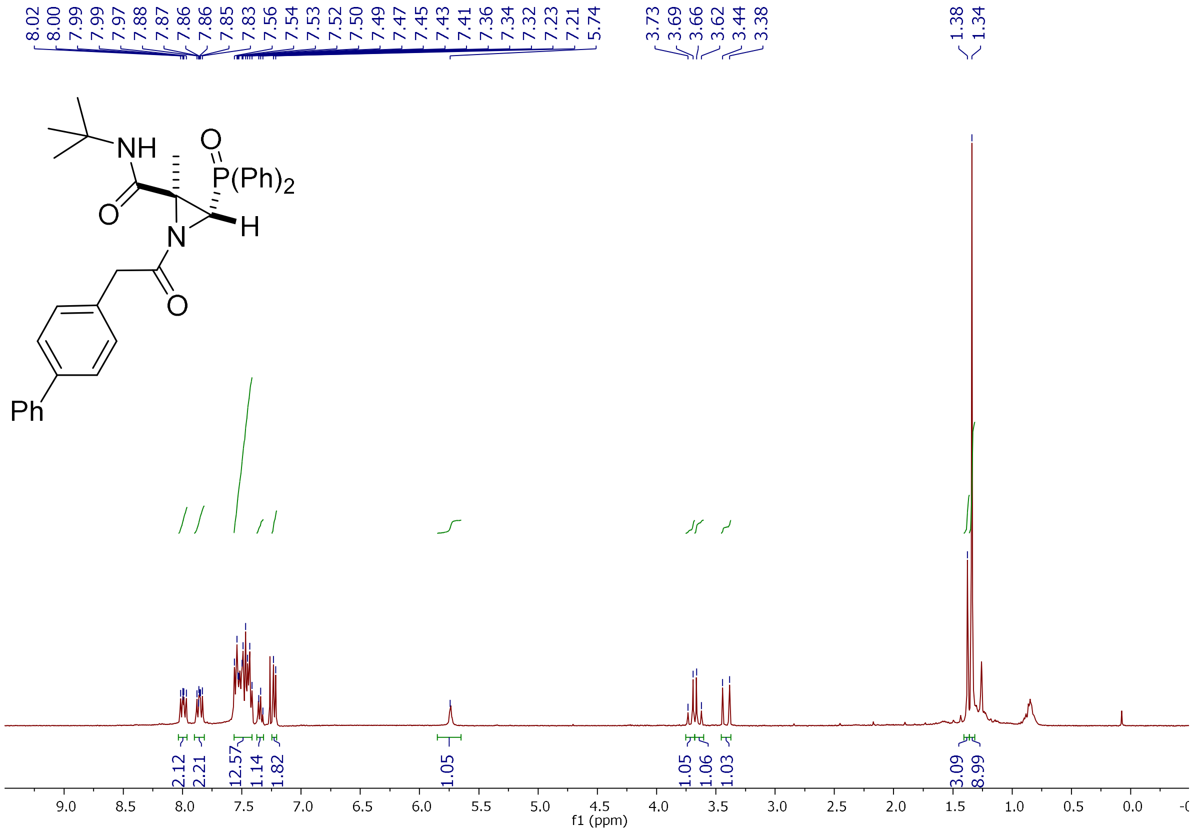

^{13}C {^{1}H} NMR (100 MHz, $CDCl_3$) of *N*-Acylaziridine Phosphine Oxide **4r**

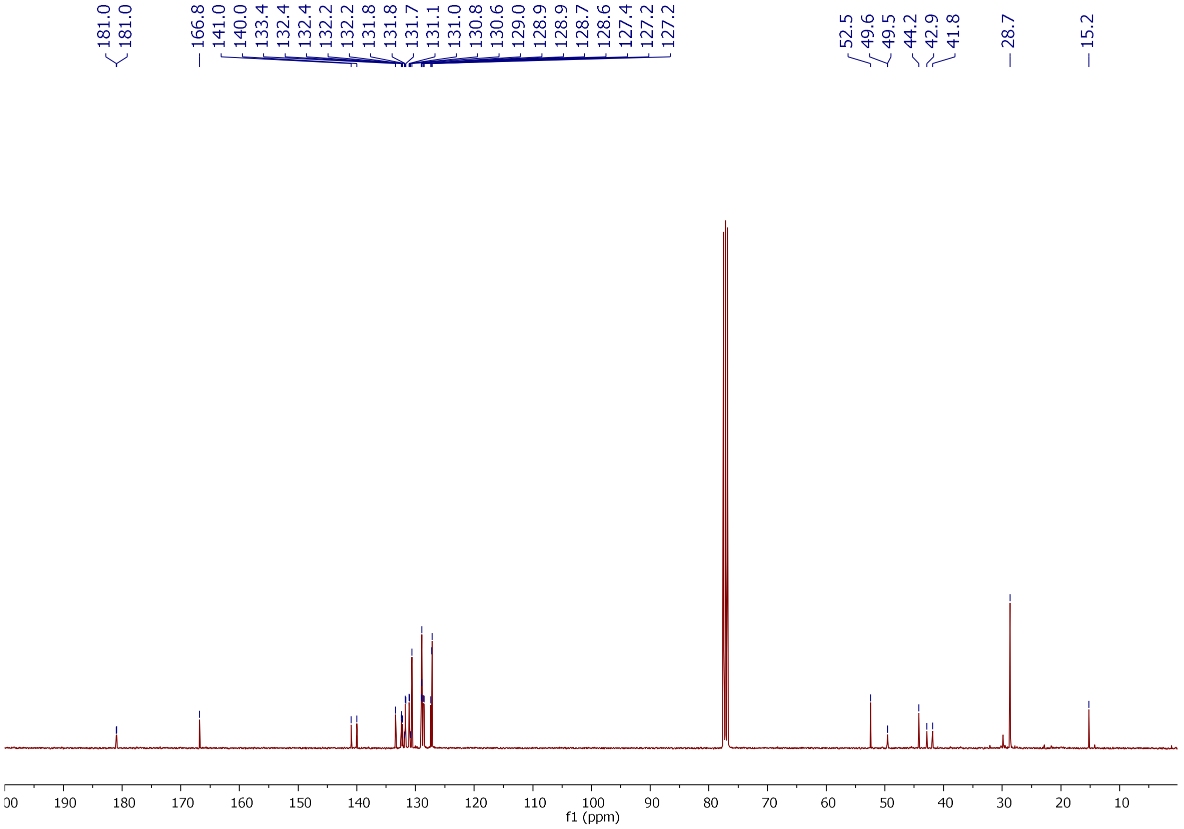

^{31}P (160 MHz, $CDCl_3$) of *N*-Acylaziridine Phosphine Oxide **4r**

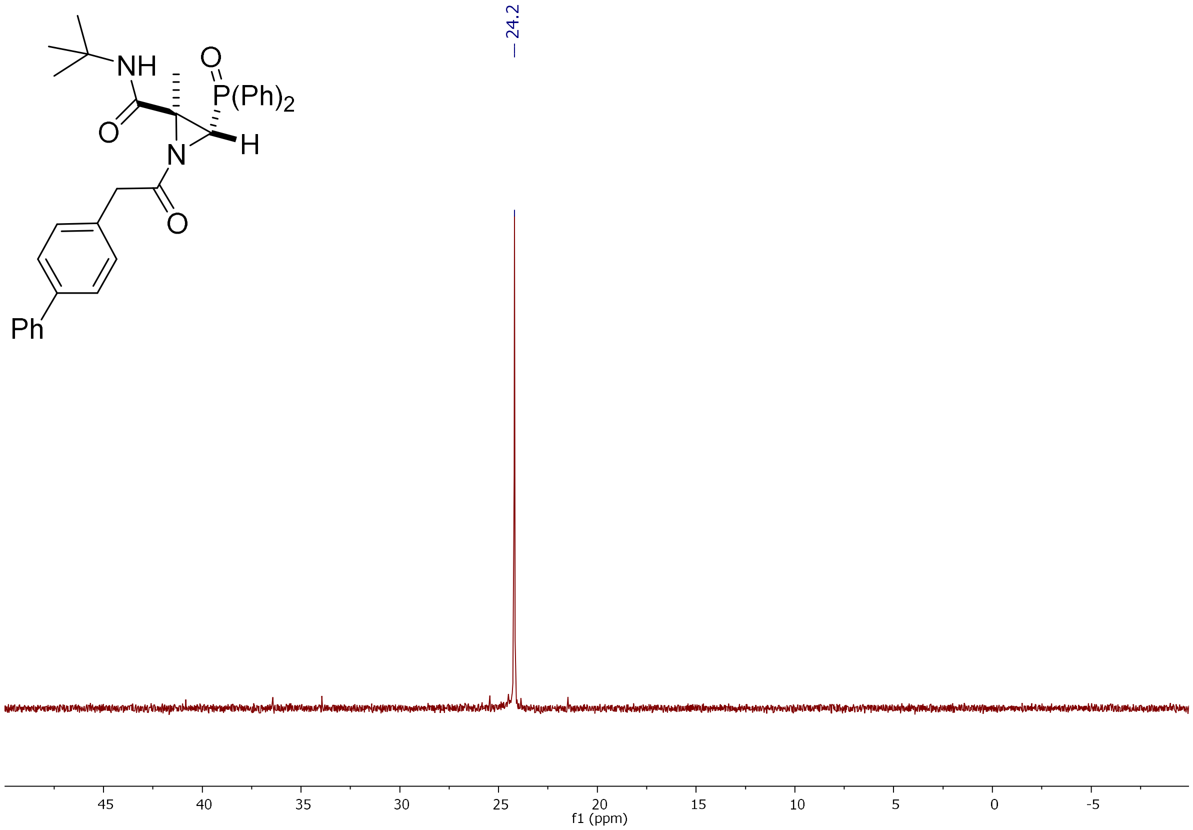

^{1}H NMR (400 MHz, $CDCl_3$) of *N*-Acylaziridine Phosphine Oxide **4s**

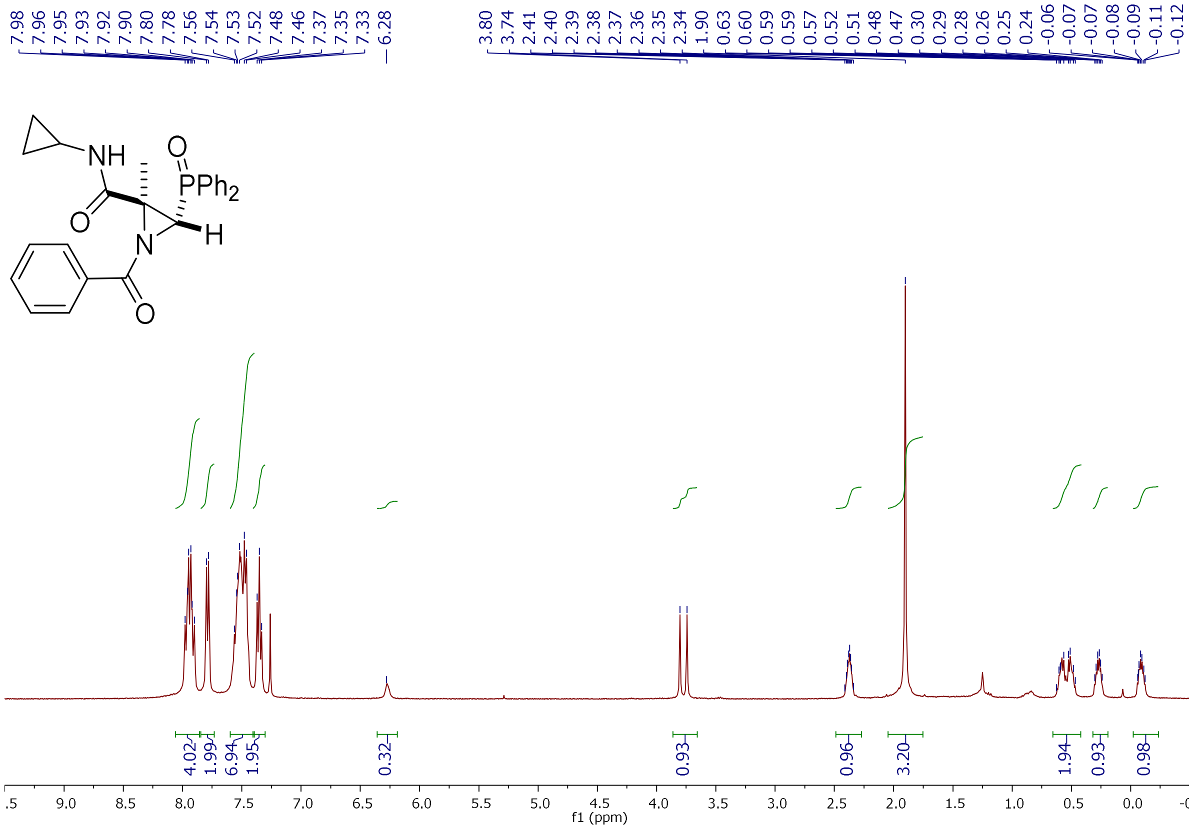

^{13}C {^{1}H} NMR (100 MHz, $CDCl_3$) of *N*-Acylaziridine Phosphine Oxide **4s**

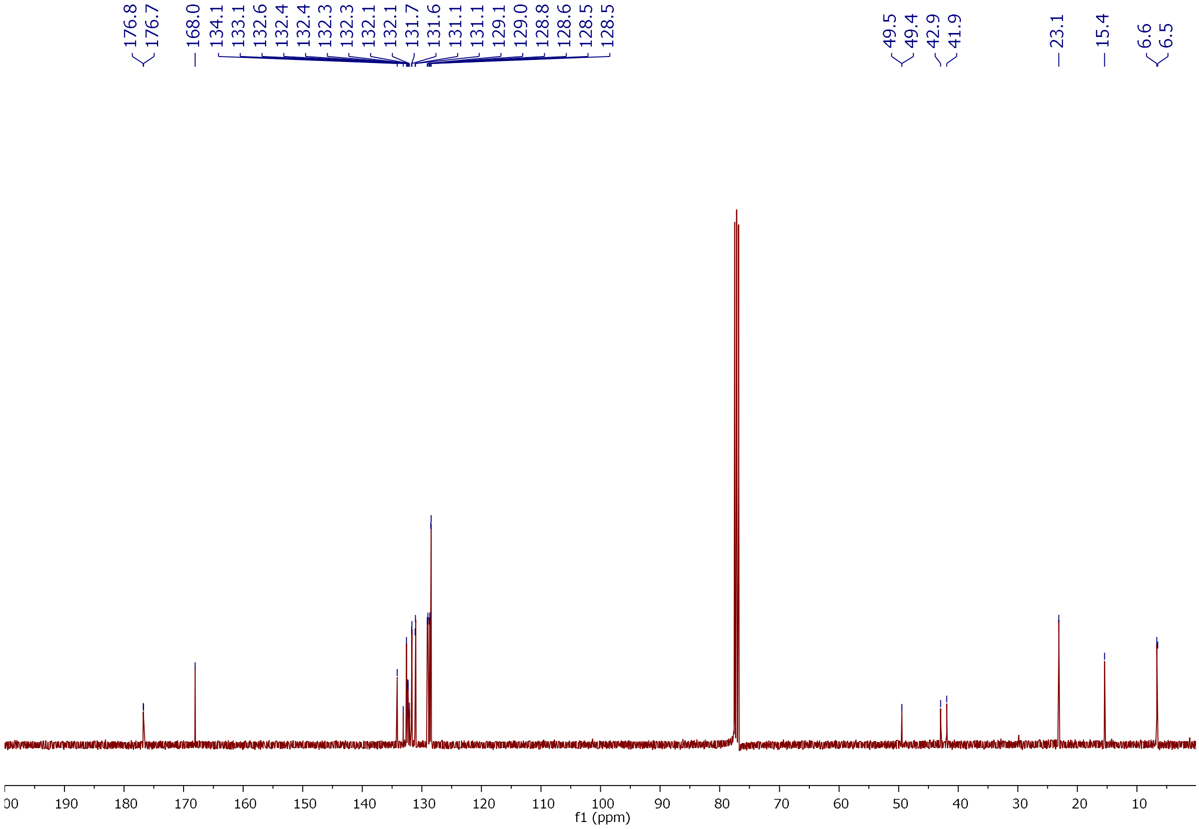

^{31}P (160 MHz, $CDCl_3$) of *N*-Acylaziridine Phosphine Oxide **4s**

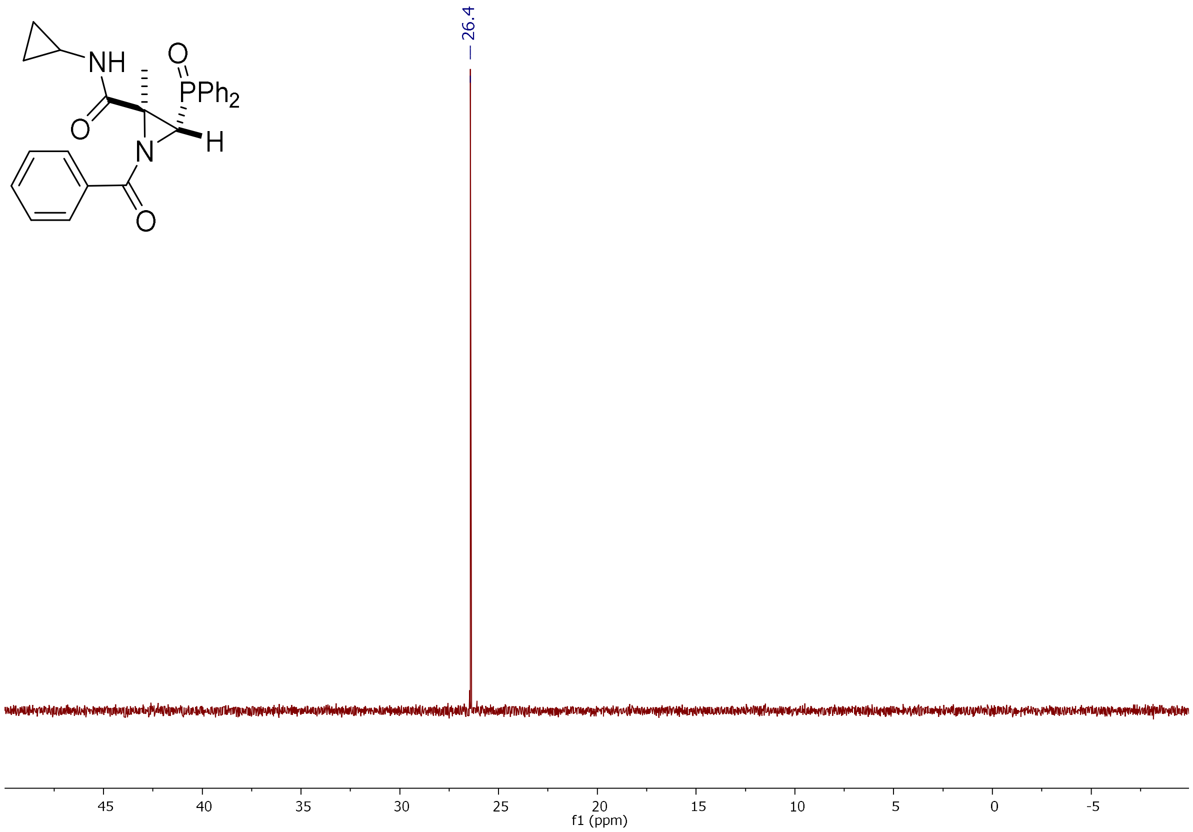

^{1}H NMR (400 MHz, CDCl$_3$) of *N*-Acylaziridine Phosphine Oxide **4t**

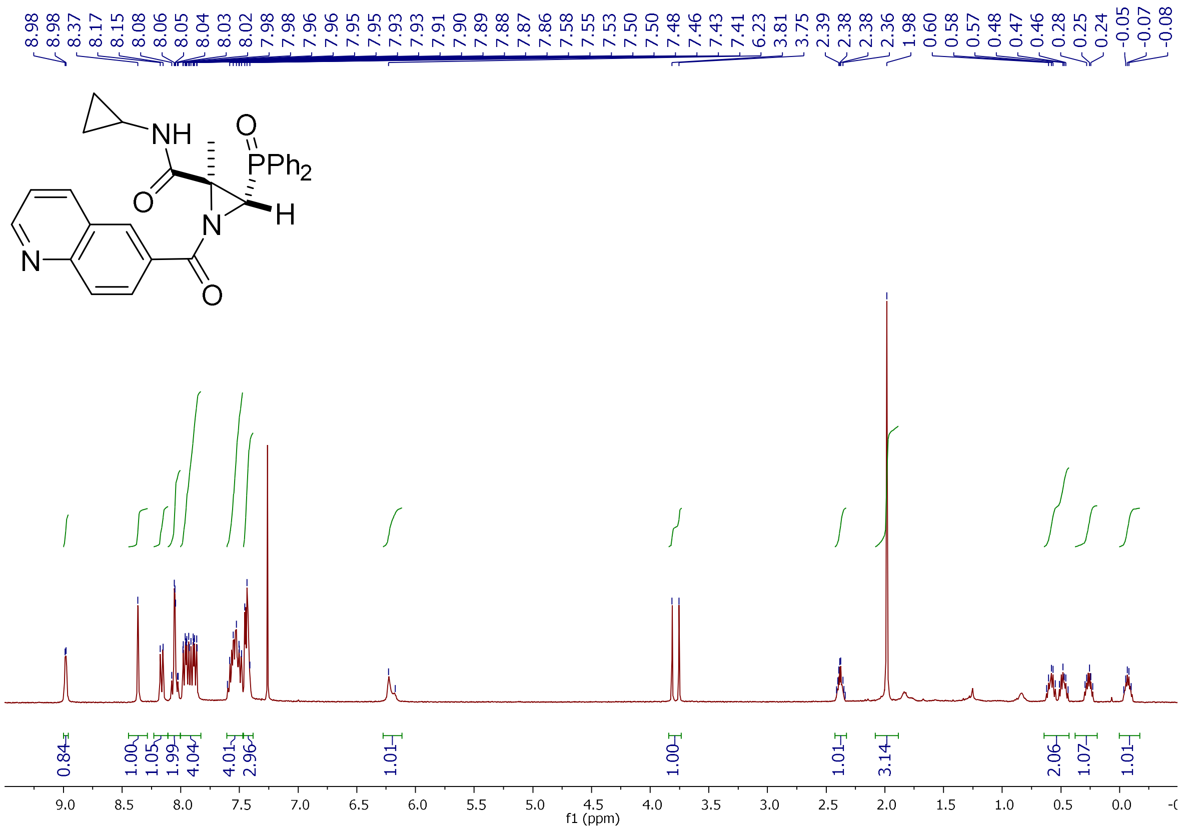

^{13}C {^{1}H} NMR (100 MHz, CDCl$_3$) of *N*-Acylaziridine Phosphine Oxide **4t**

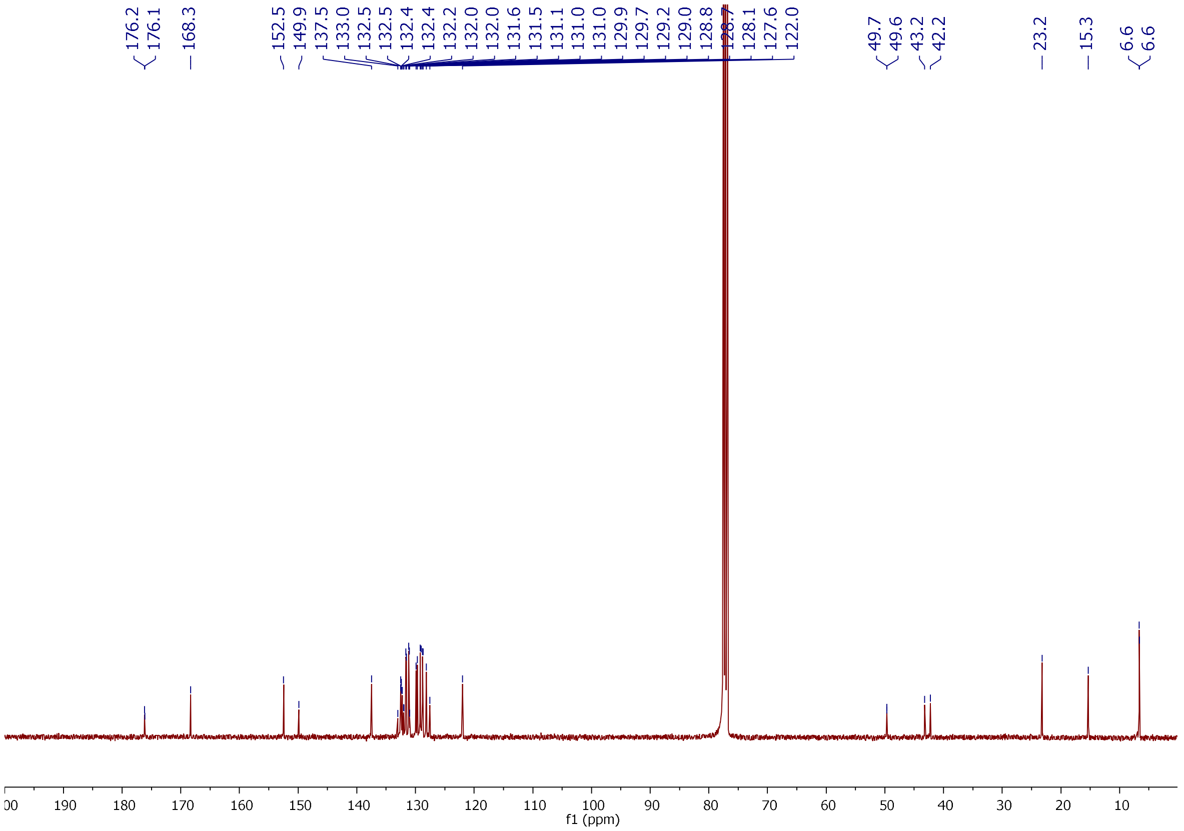

^{31}P (160 MHz, $CDCl_3$) of *N*-Acylaziridine Phosphine Oxide **4t**

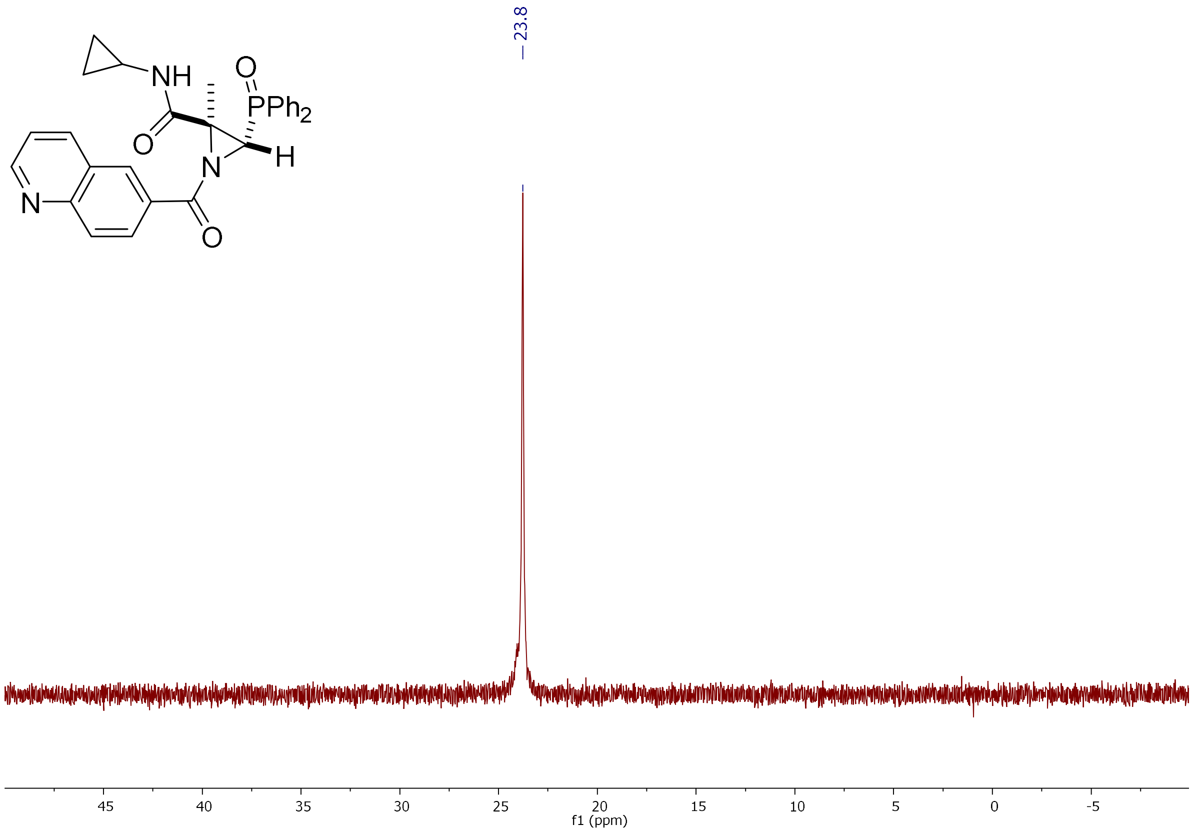

^{1}H NMR (400 MHz, $CDCl_3$) of *N*-Acylaziridine Phosphine Oxide **4u**

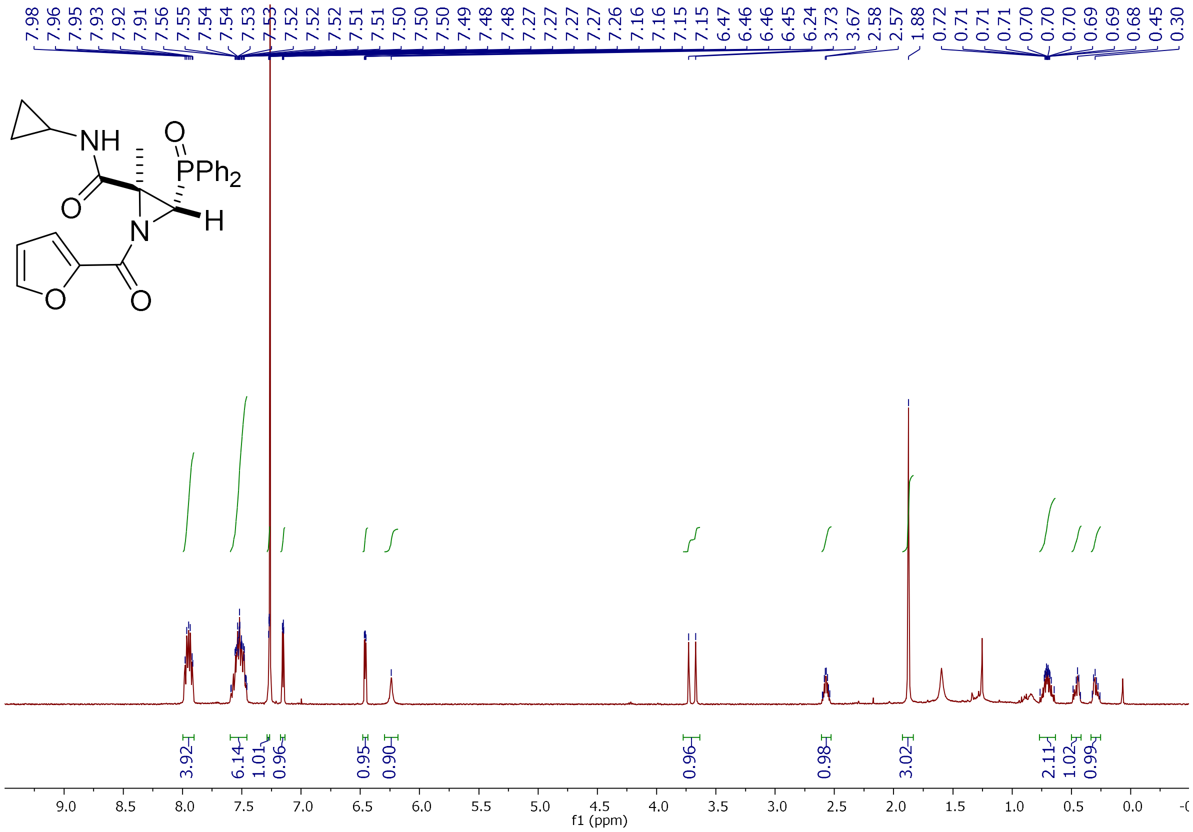

^{13}C {^{1}H} NMR (100 MHz, $CDCl_3$) of *N*-Acylaziridine Phosphine Oxide **4u**

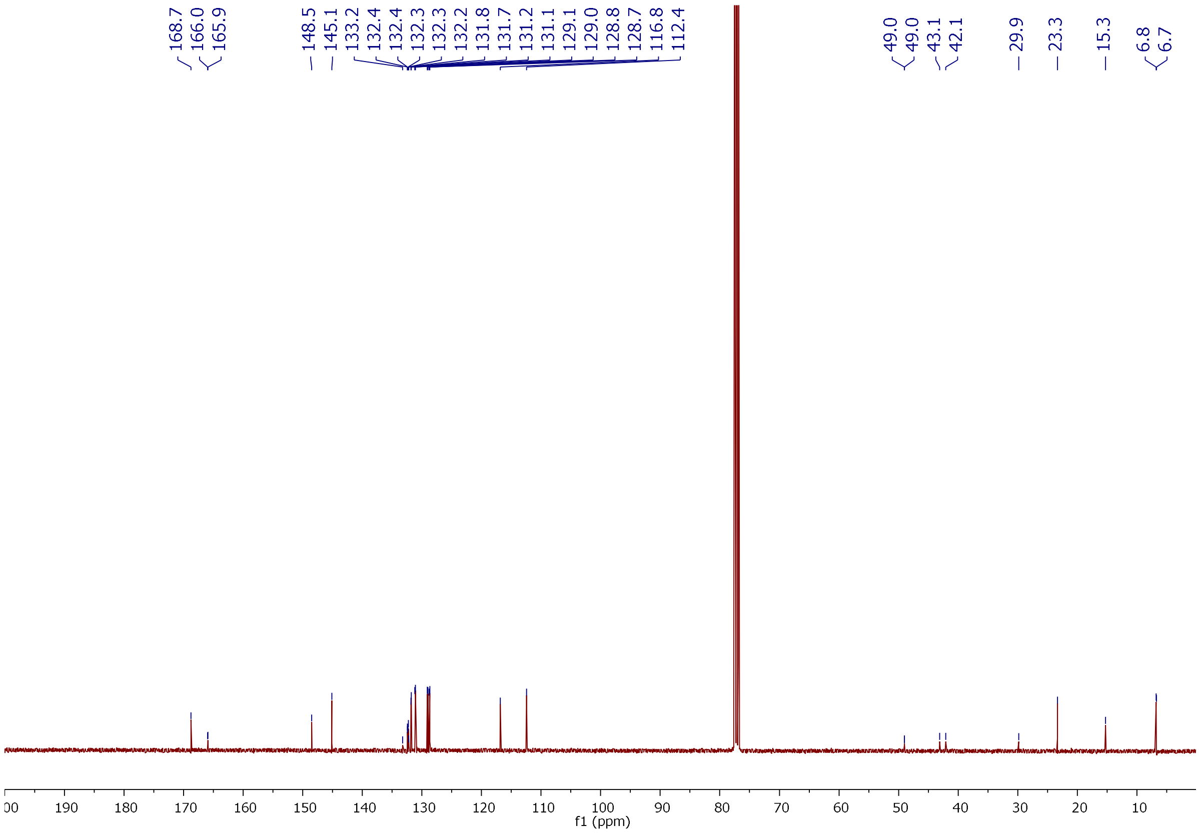

^{31}P (160 MHz, $CDCl_3$) of *N*-Acylaziridine Phosphine Oxide **4u**

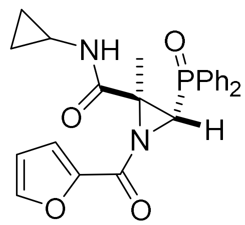

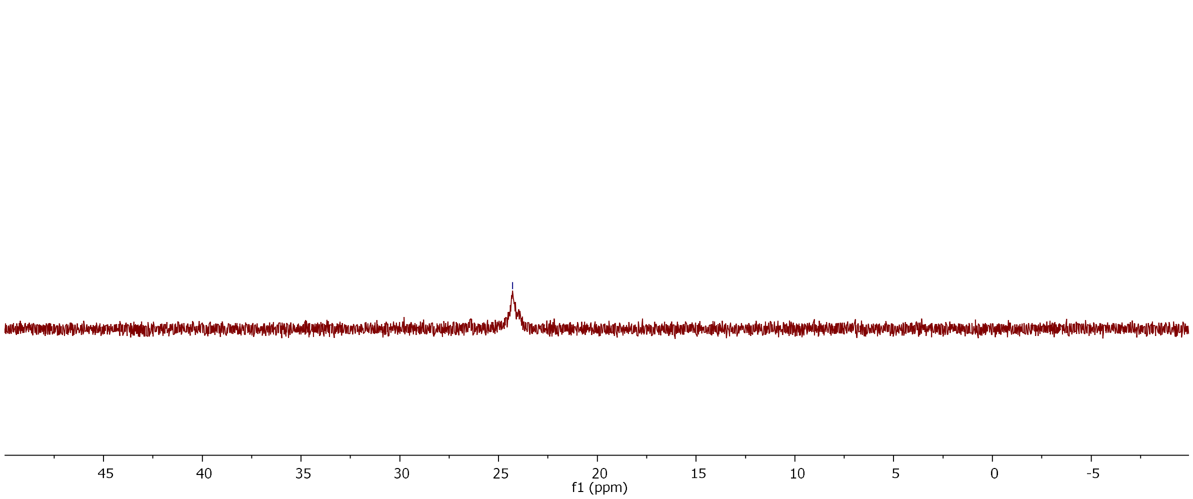

^{1}H NMR (400 MHz, $CDCl_3$) of *N*-Acylaziridine Phosphonate **5a**

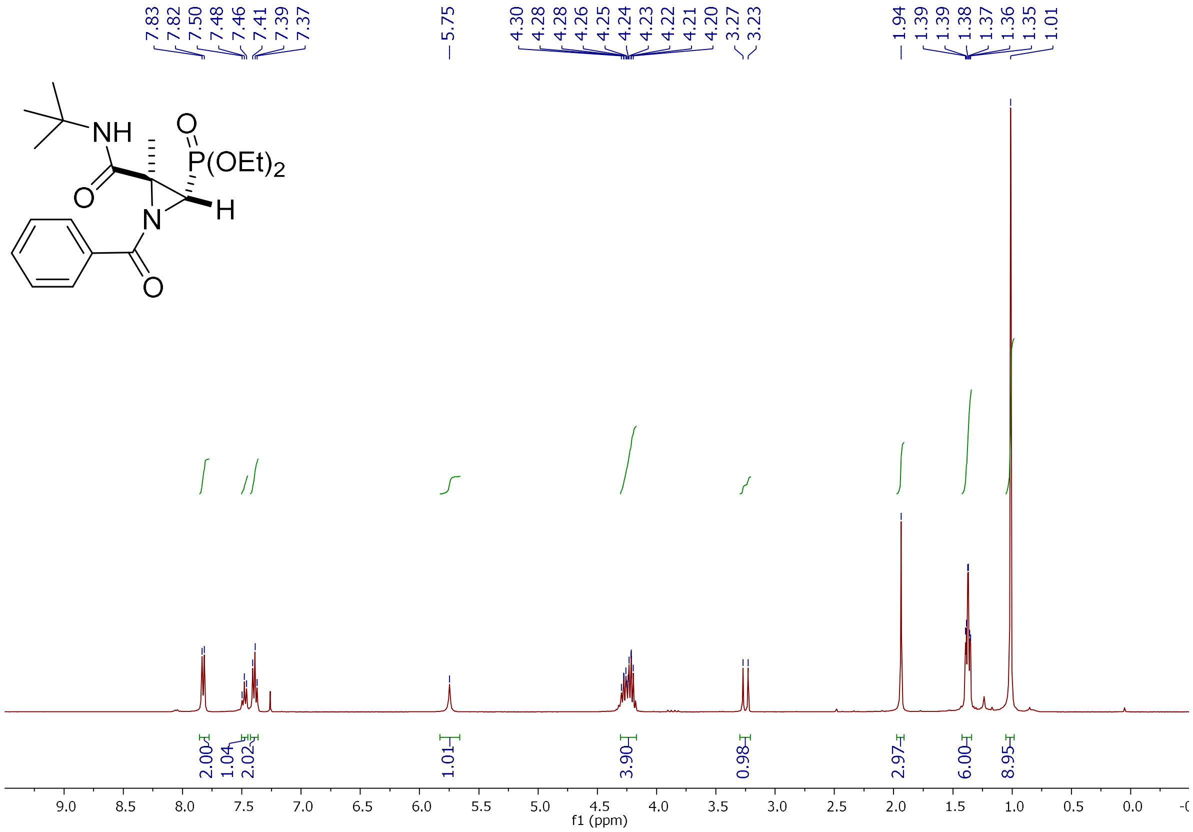

^{13}C {^{1}H} NMR (100 MHz, $CDCl_3$) of *N*-Acylaziridine Phosphonate **5a**

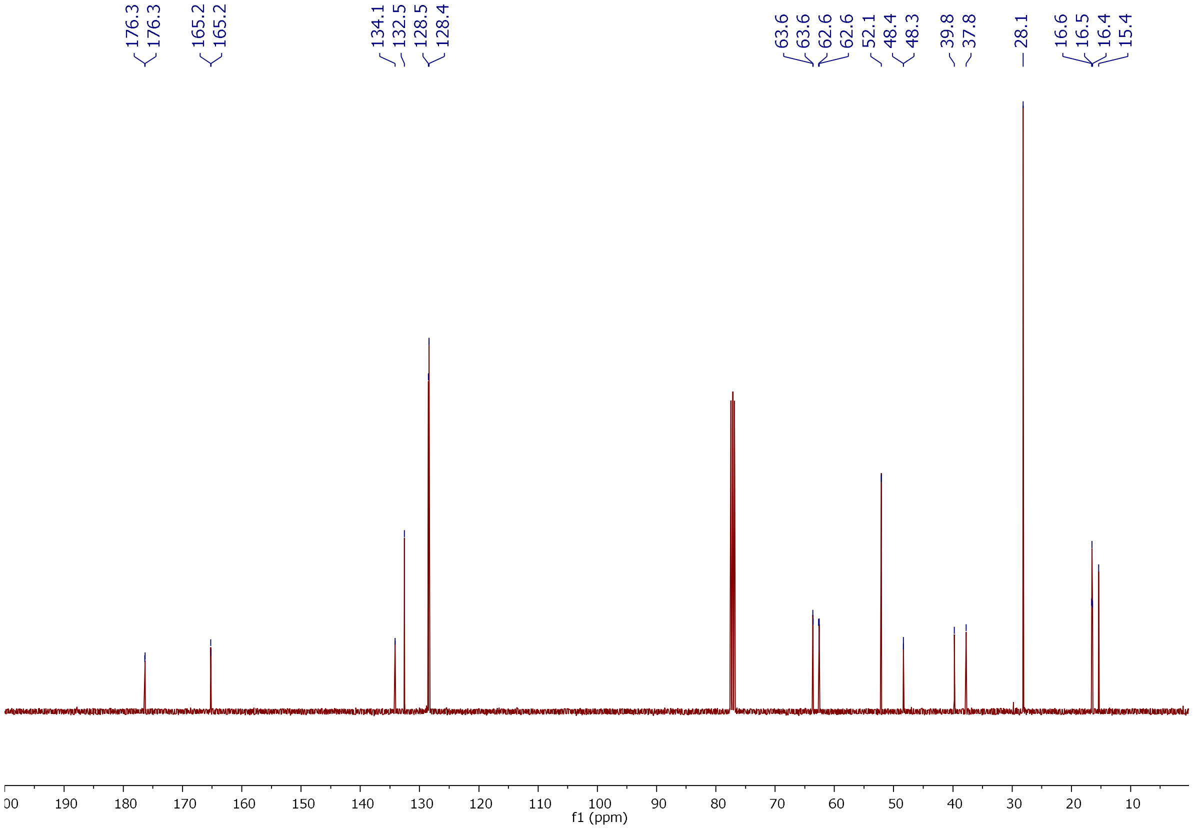

^{31}P (160 MHz, $CDCl_3$) of *N*-Acylaziridine Phosphonate **5a**

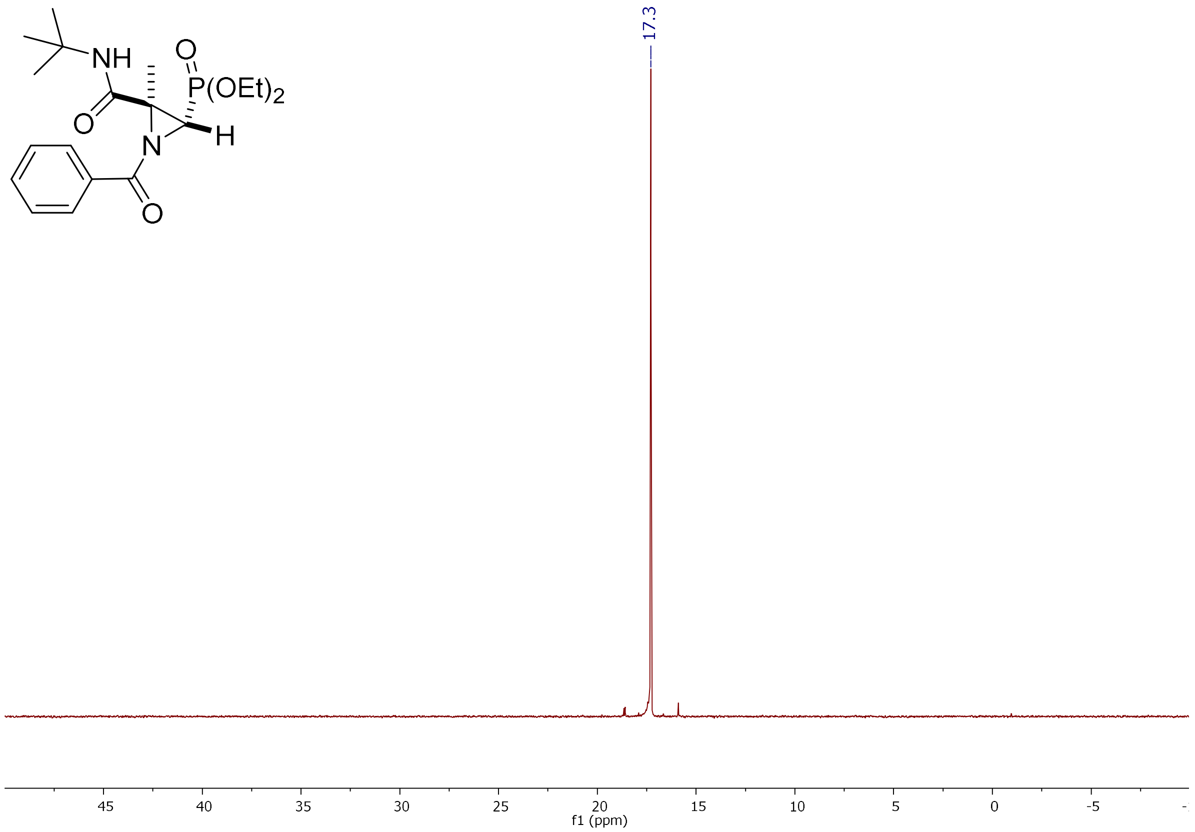

^{1}H NMR (400 MHz, $CDCl_3$) of *N*-Acylaziridine Phosphonate **5b**

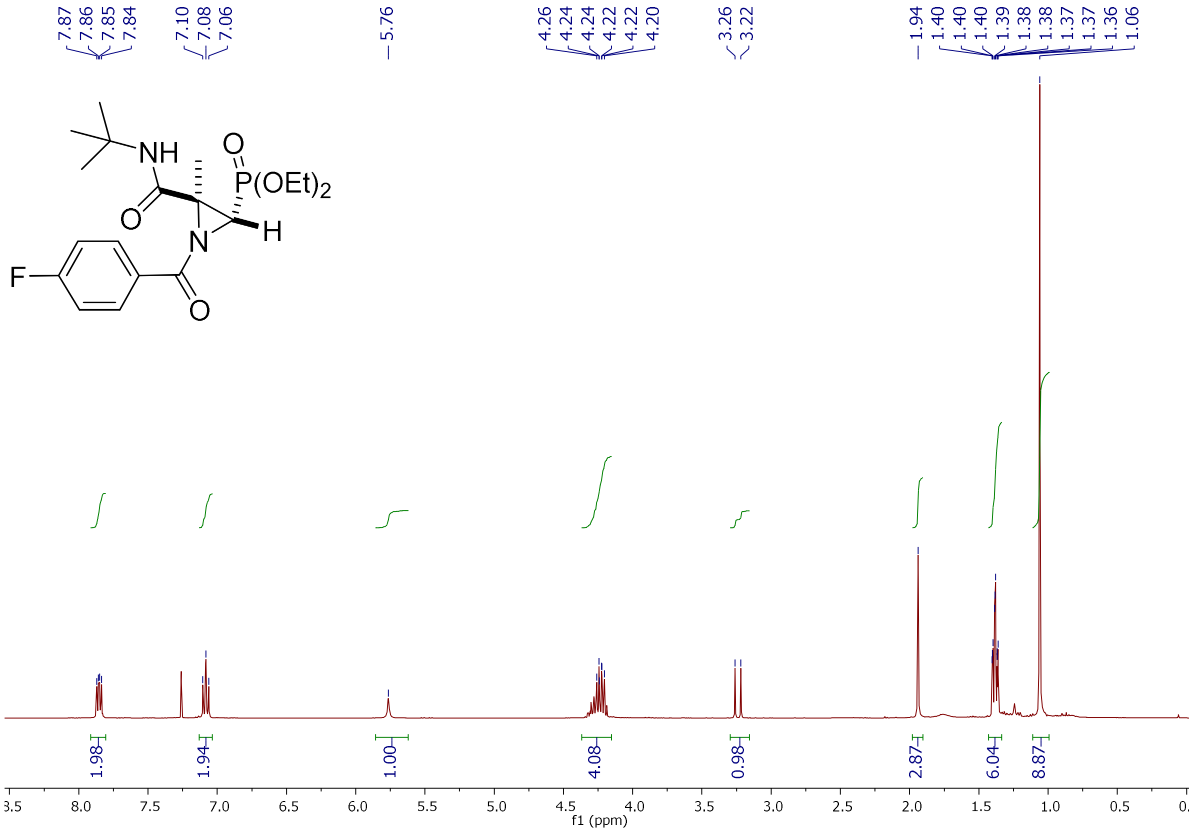

^{13}C {^{1}H} NMR (100 MHz, $CDCl_3$) of *N*-Acylaziridine Phosphonate **5b**

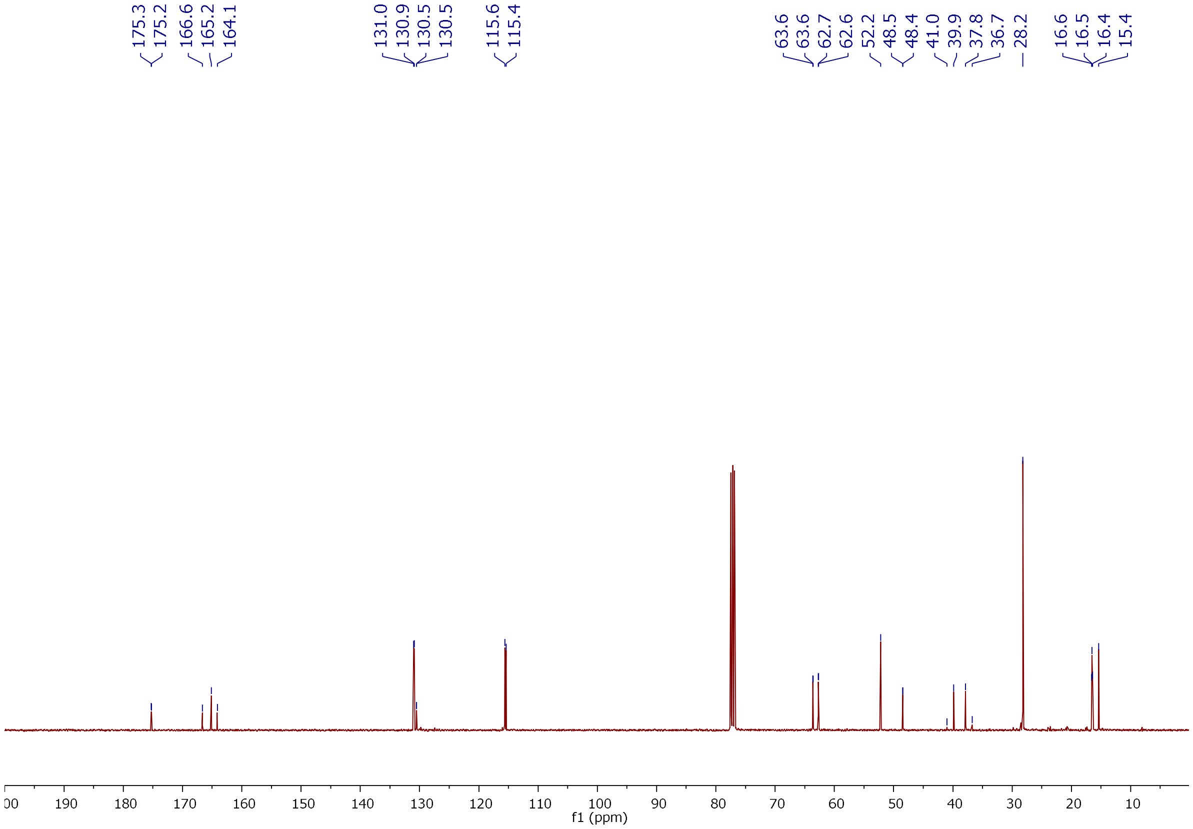

^{31}P (160 MHz, $CDCl_3$) of *N*-Acylaziridine Phosphonate **5b**

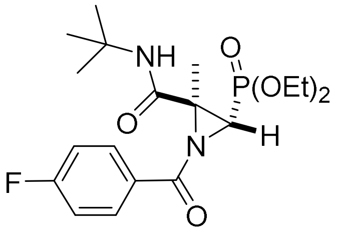

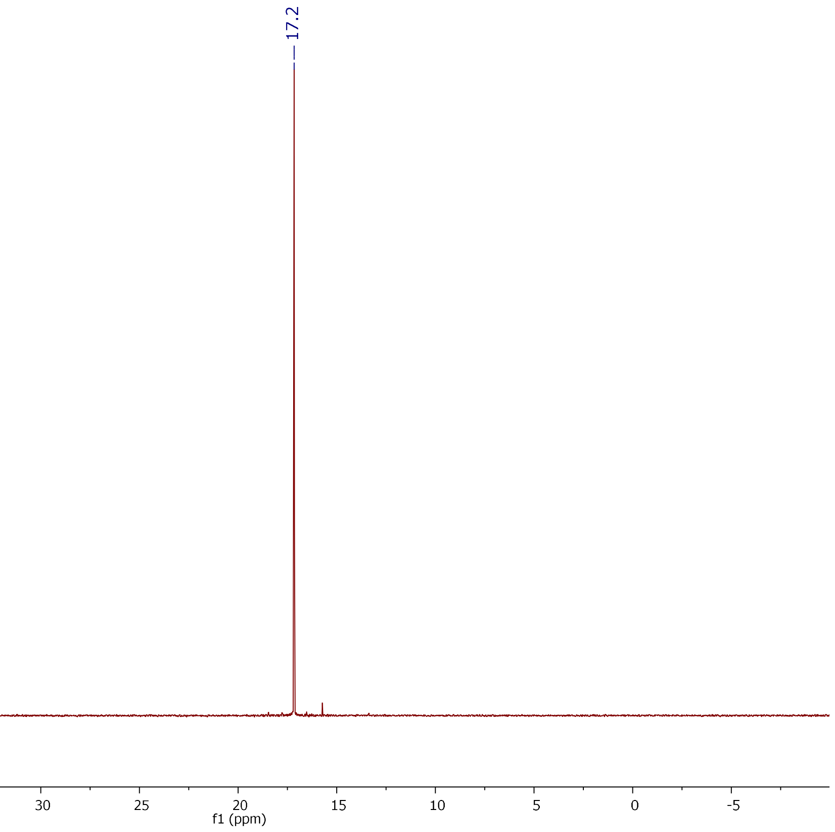

^{19}F (376 MHz, $CDCl_3$) of *N*-Acylaziridine Phosphonate **5b**

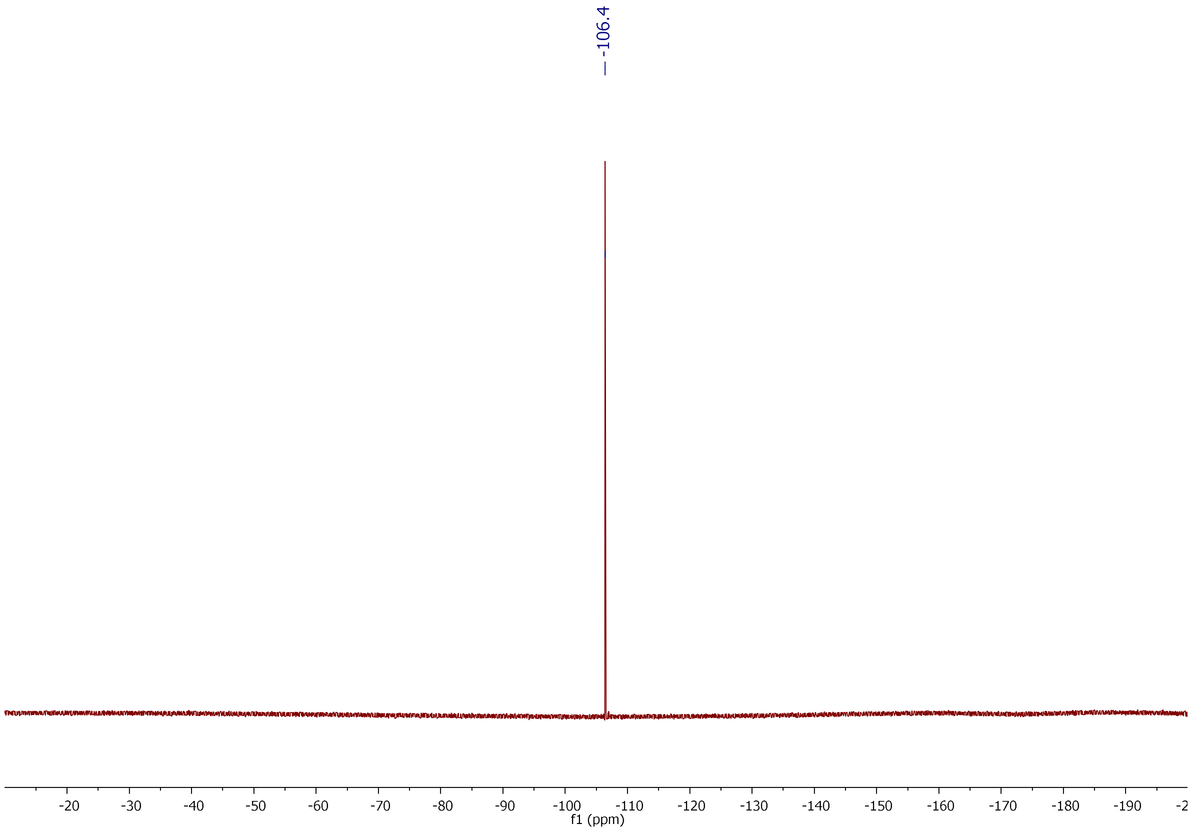

^{1}H NMR (400 MHz, $CDCl_3$) of *N*-Acylaziridine Phosphonate **5c**

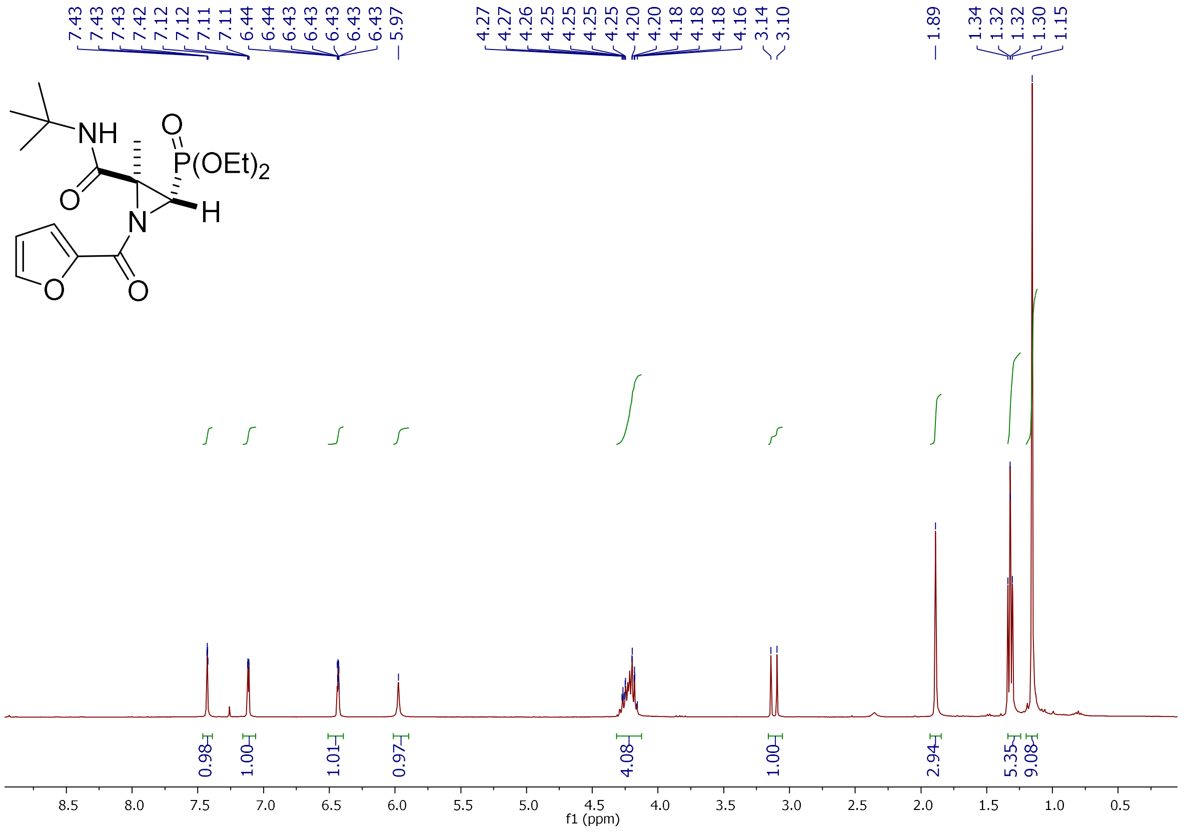

^{13}C {^{1}H} NMR (100 MHz, $CDCl_3$) of *N*-Acylaziridine Phosphonate **5c**

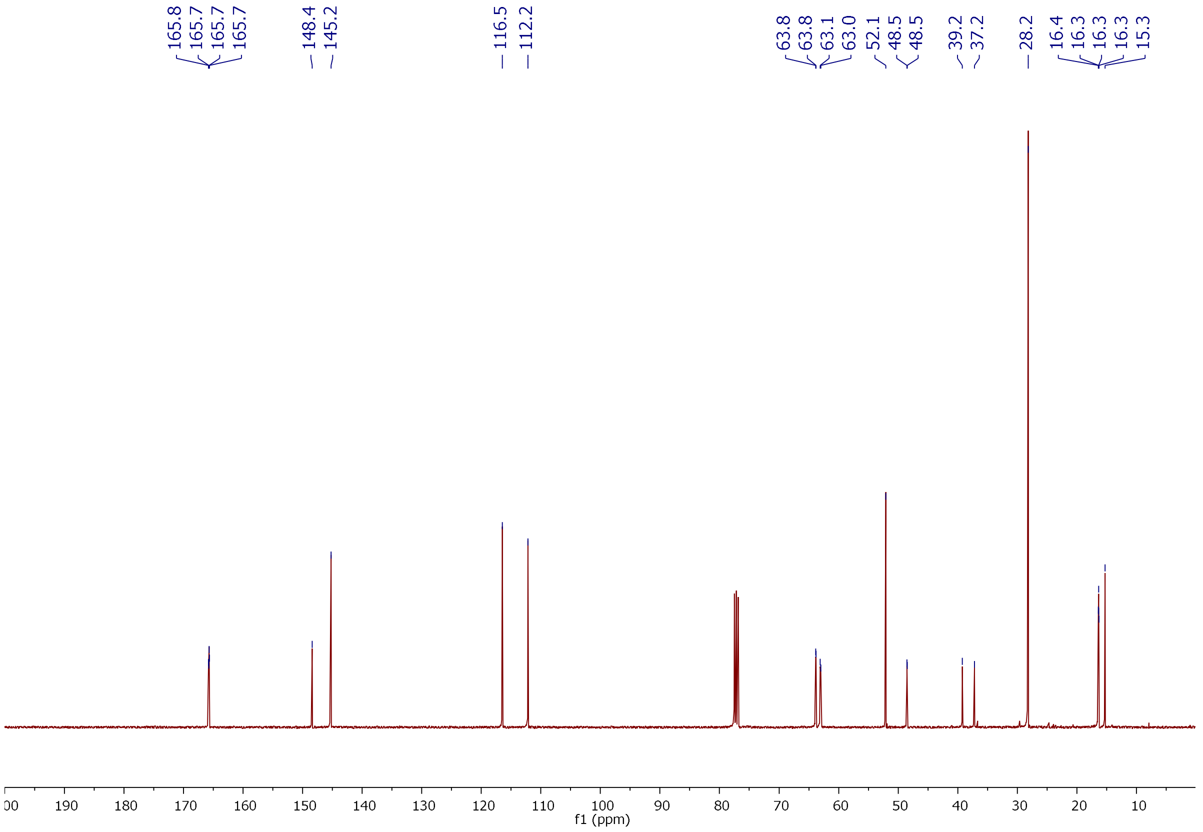

^{31}P (160 MHz, $CDCl_3$) of *N*-Acylaziridine Phosphonate **5c**

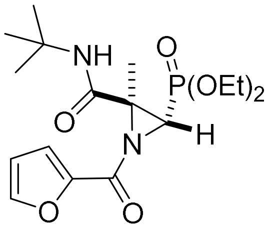

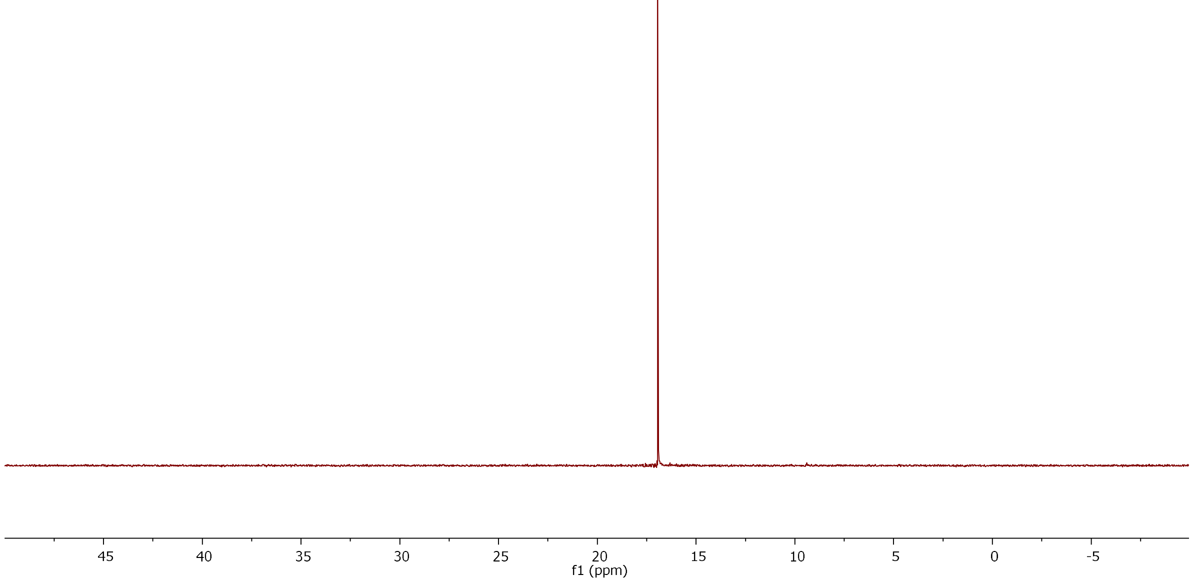

^{1}H NMR (400 MHz, $CDCl_3$) of *N*-Acylaziridine Phosphonate **5d**

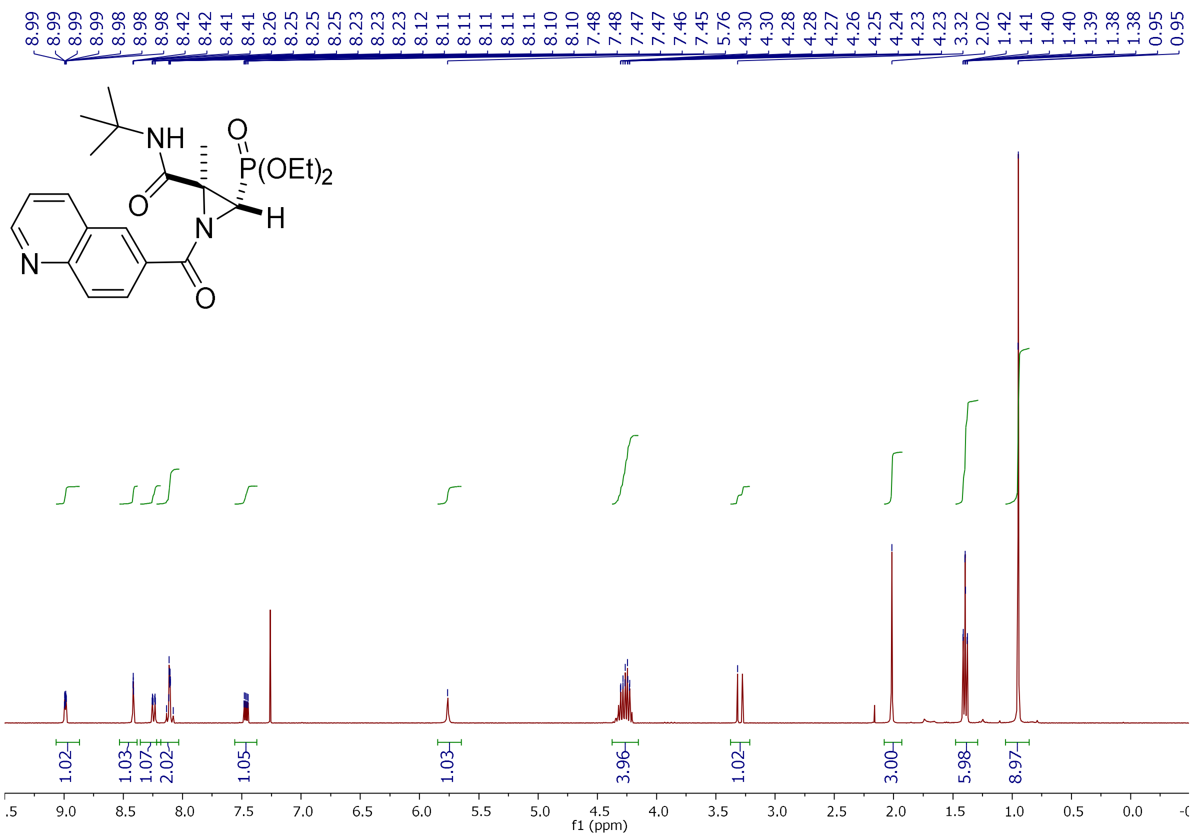

^{13}C {^{1}H} NMR (100 MHz, $CDCl_3$) of *N*-Acylaziridine Phosphonate **5d**

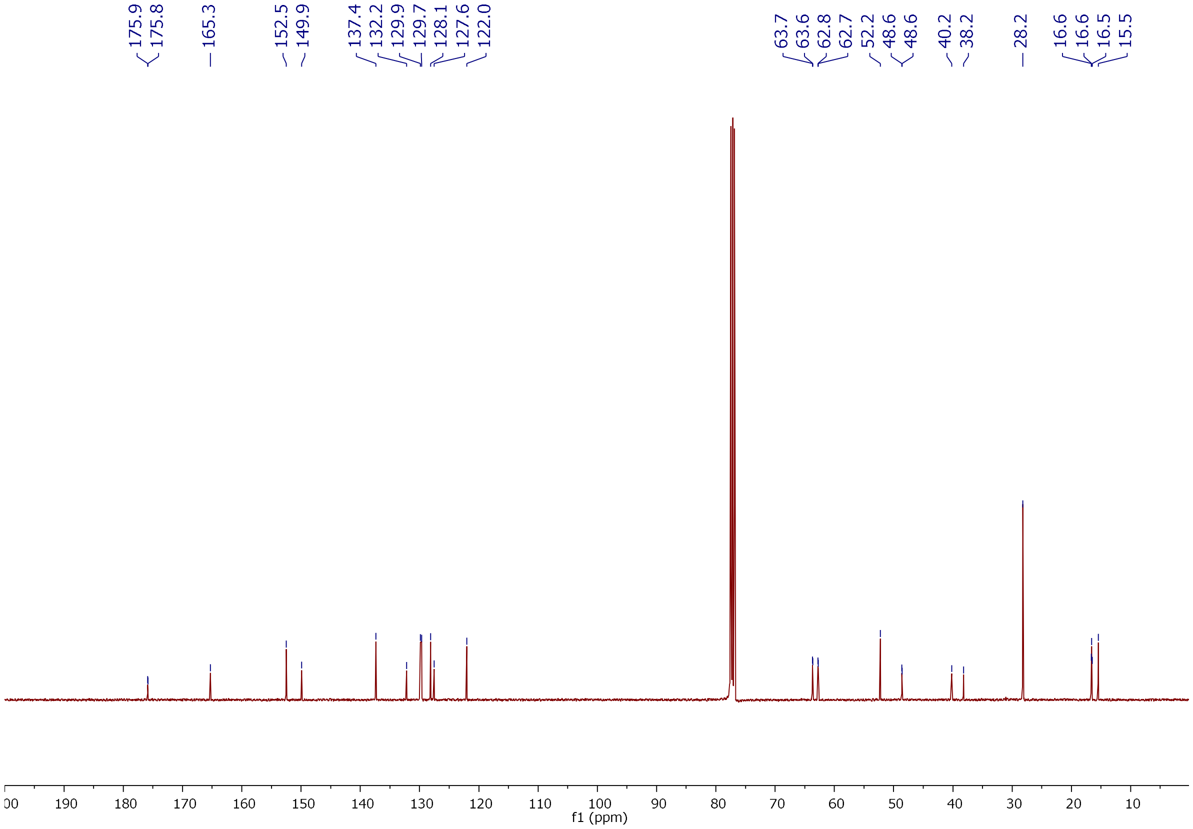

^{31}P (160 MHz, $CDCl_3$) of *N*-Acylaziridine Phosphonate **5d**

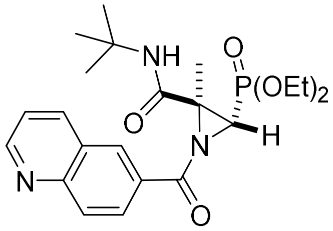

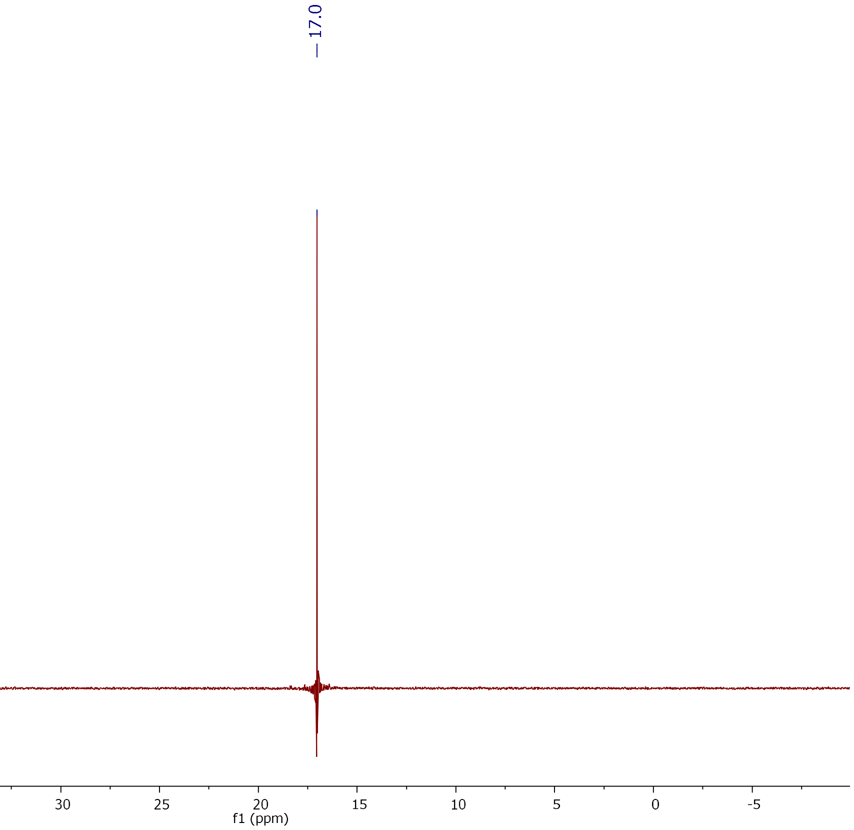

^{1}H NMR (400 MHz, $CDCl_3$) of *N*-Acylaziridine Phosphonate **5e**

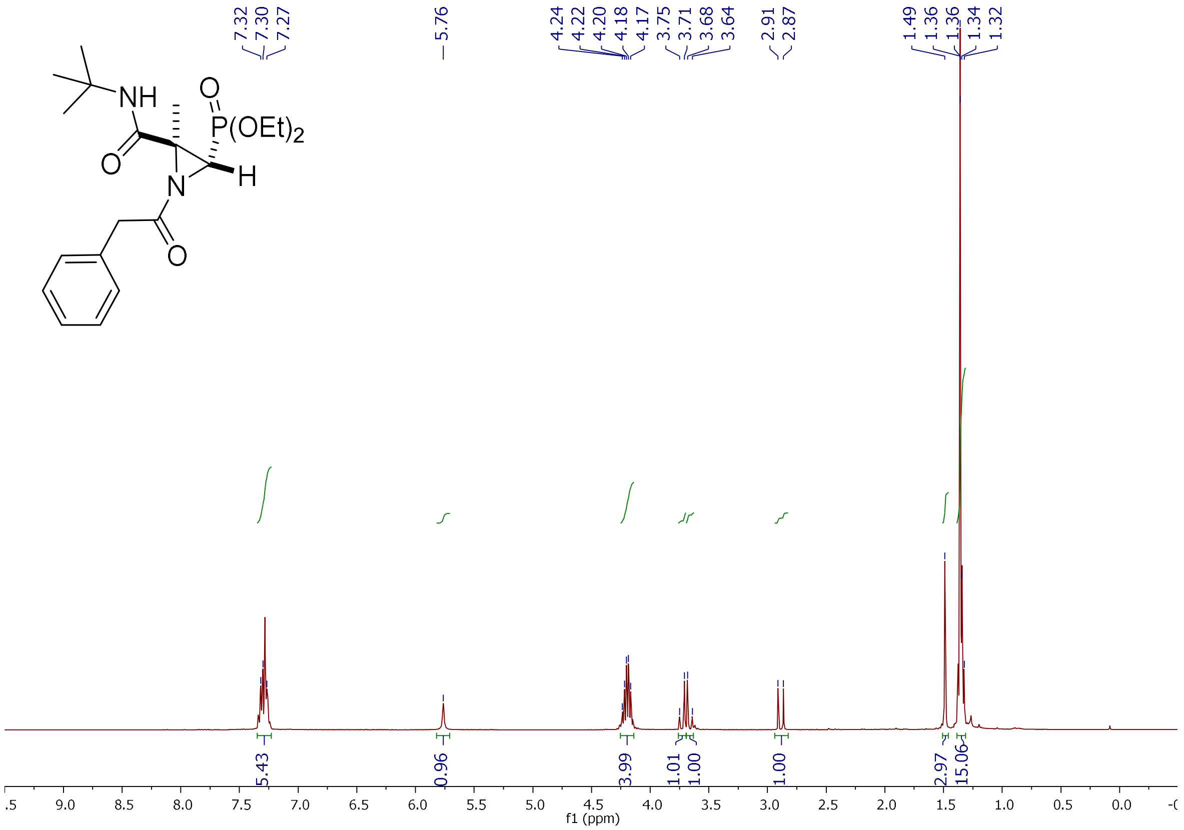

^{13}C {^{1}H} NMR (100 MHz, $CDCl_3$) of *N*-Acylaziridine Phosphonate **5e**

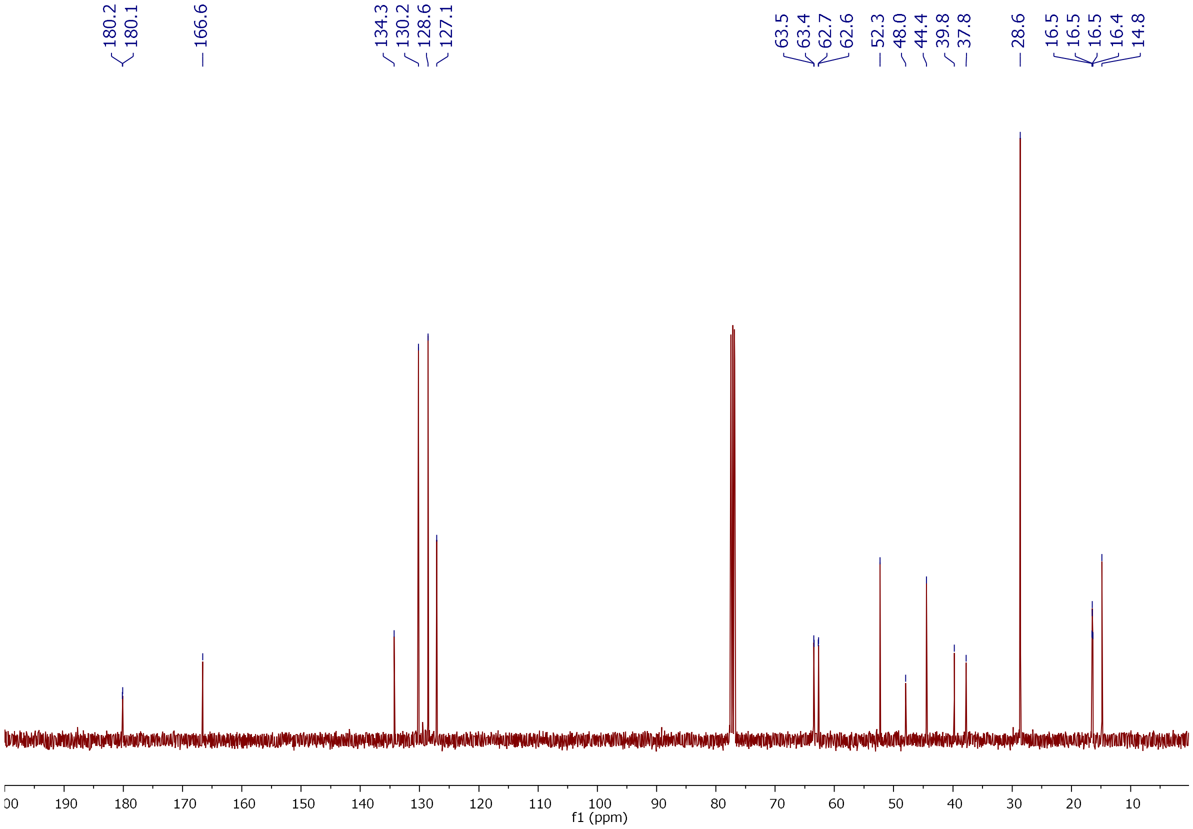

^{31}P (160 MHz, $CDCl_3$) of *N*-Acylaziridine Phosphonate **5e**

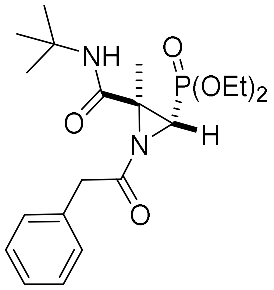

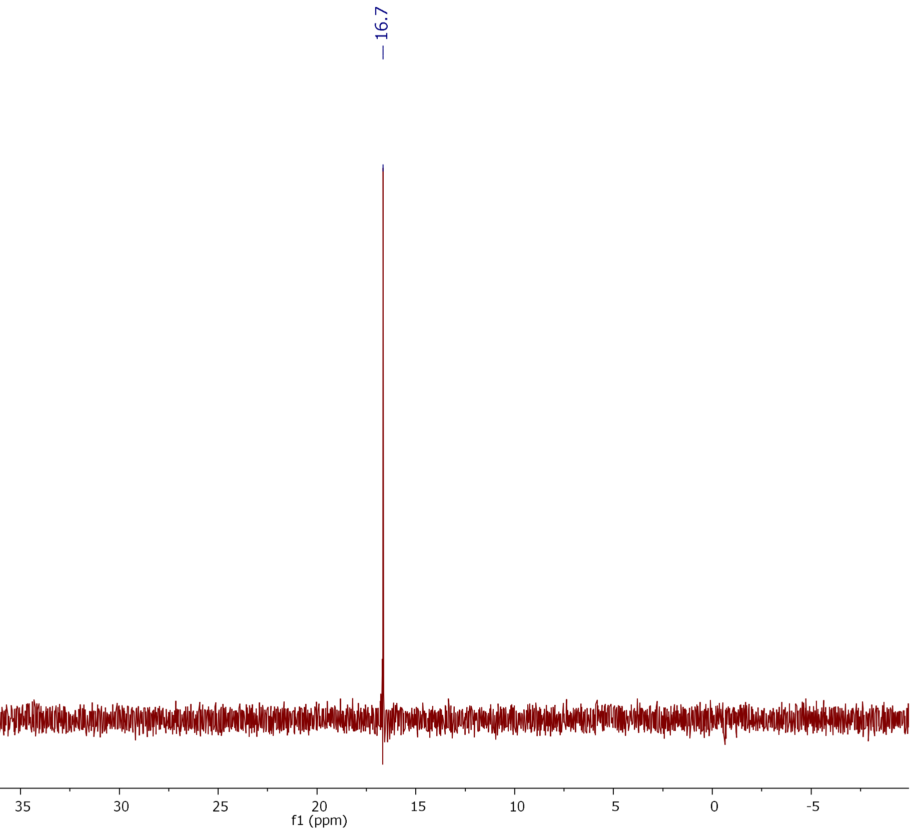

^{1}H NMR (400 MHz, $CDCl_3$) of *N*-Acylaziridine Phosphonate **5f**

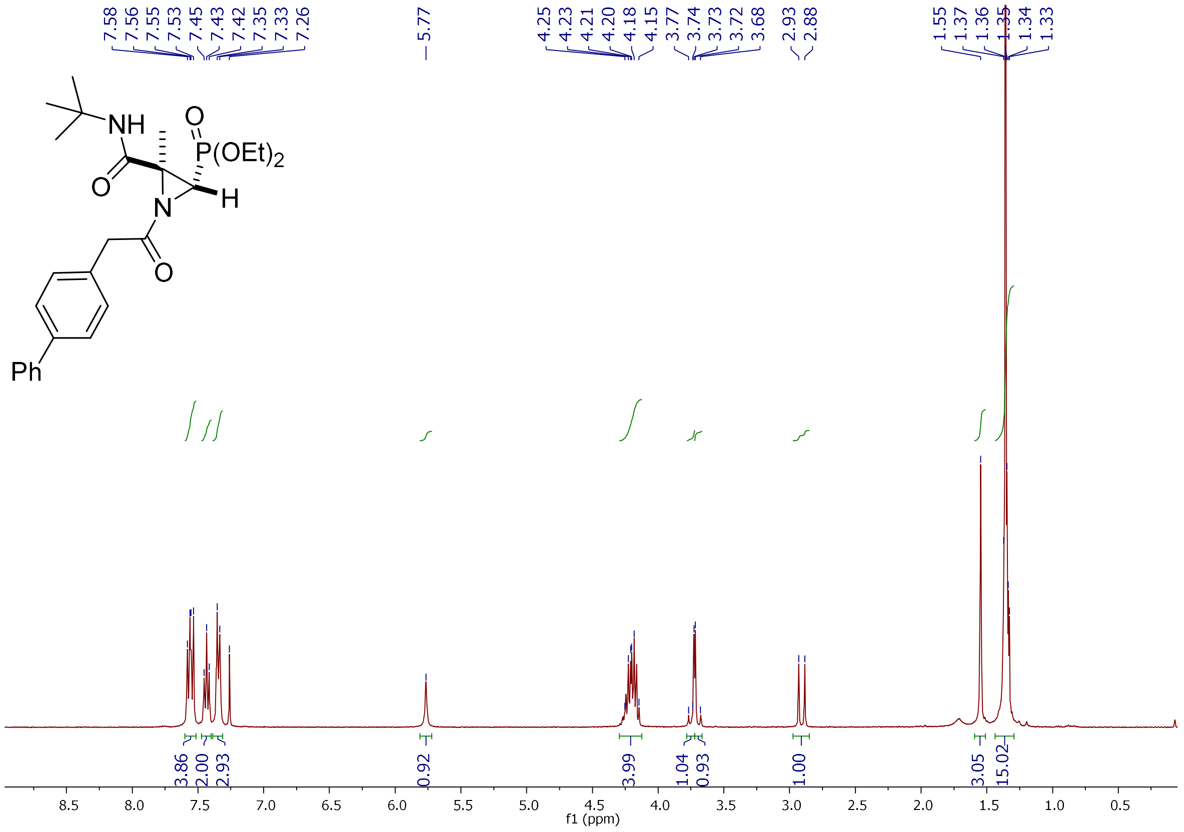

^{13}C {^{1}H} NMR (100 MHz, $CDCl_3$) of *N*-Acylaziridine Phosphonate **5f**

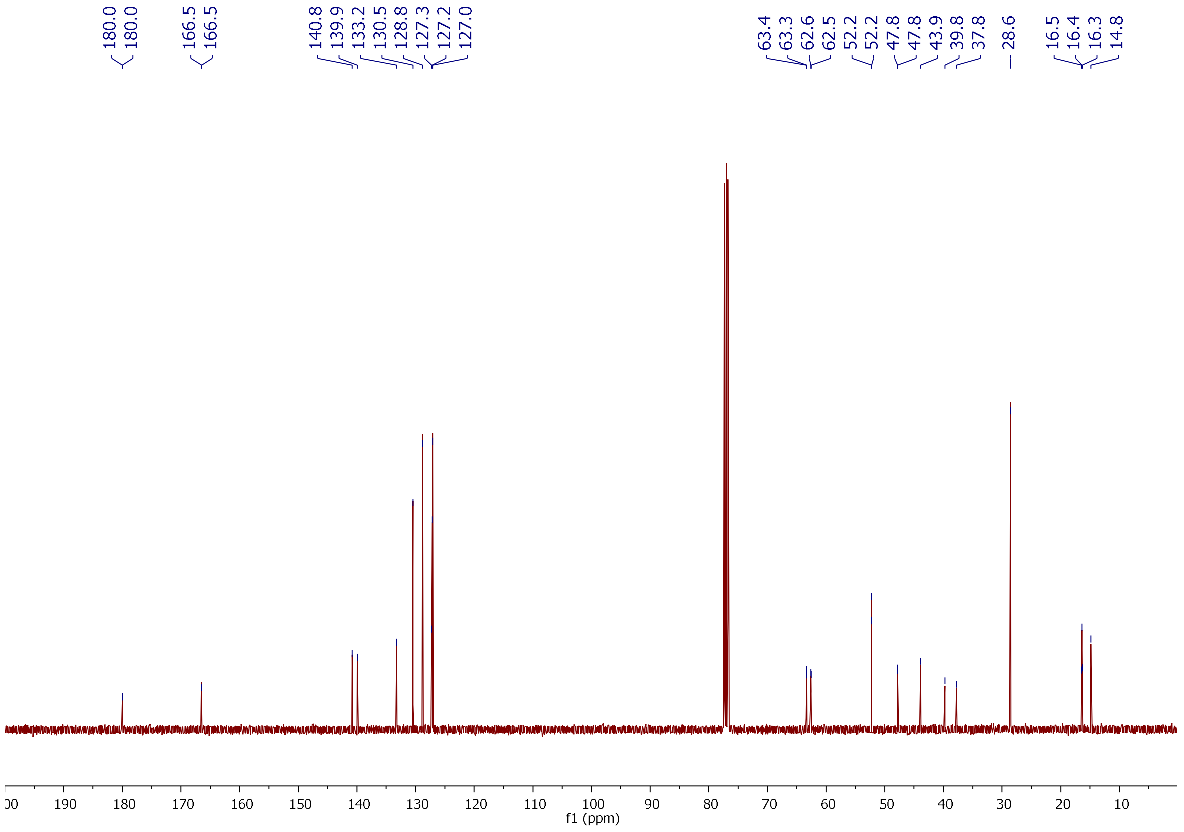

^{31}P (160 MHz, $CDCl_3$) of *N*-Acylaziridine Phosphonate **5f**

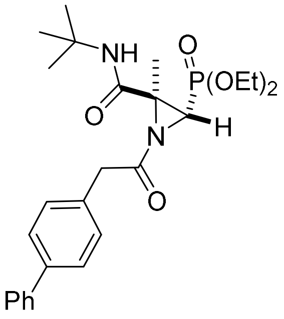

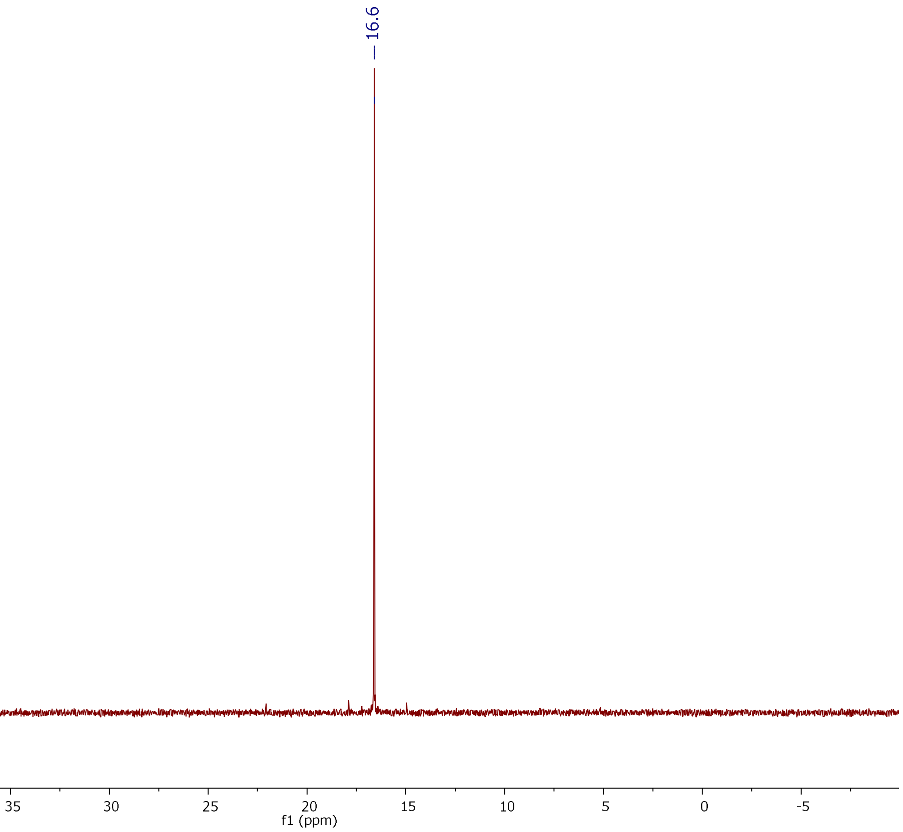

^{1}H NMR (400 MHz, $CDCl_3$) of *N*-Acylaziridine Phosphonate **5g**

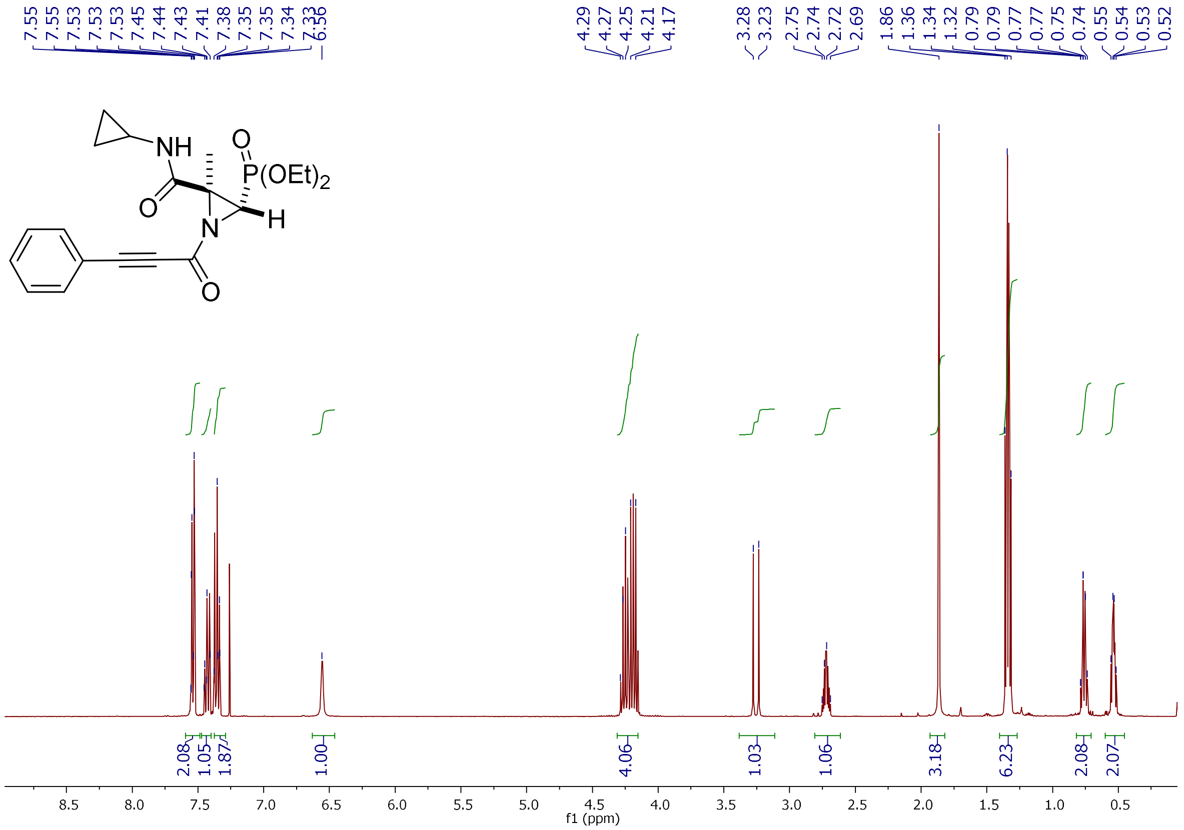

^{13}C {^{1}H} NMR (100 MHz, $CDCl_3$) of *N*-Acylaziridine Phosphonate **5g**

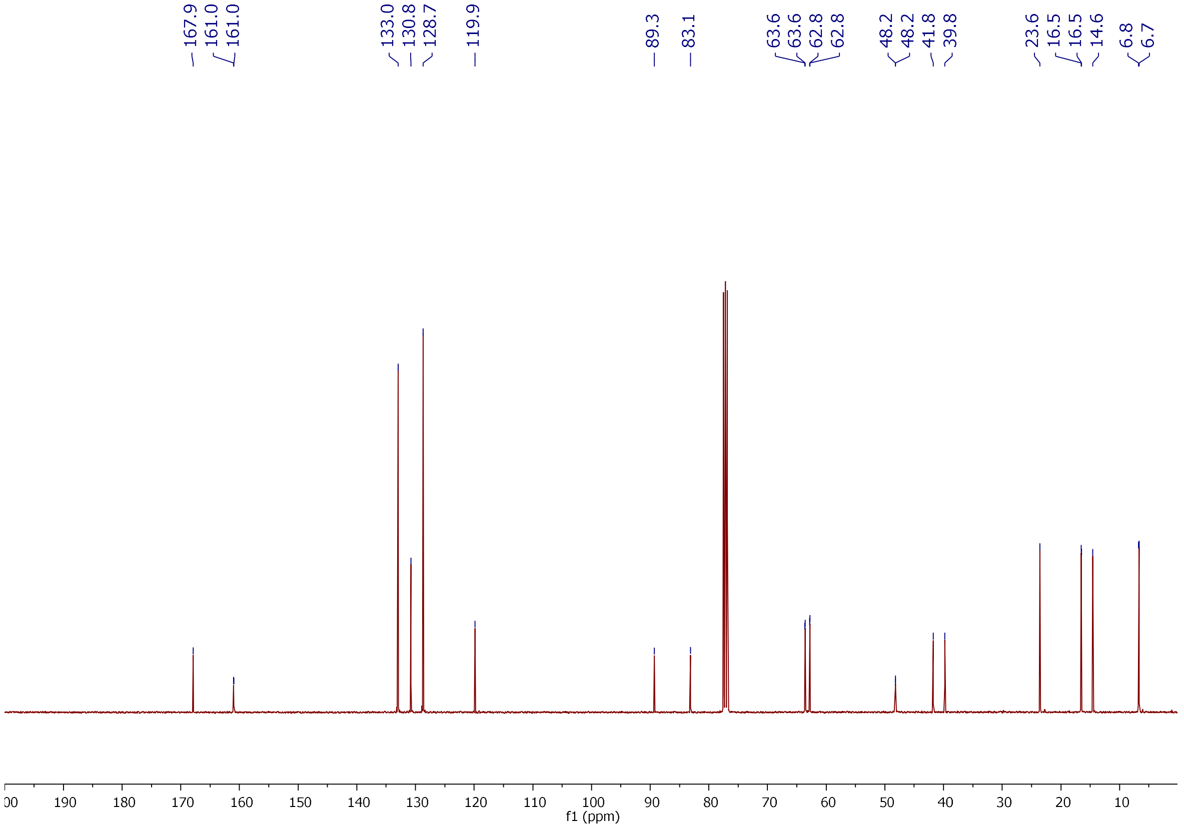

^{31}P (160 MHz, $CDCl_3$) of *N*-Acylaziridine Phosphonate **5g**

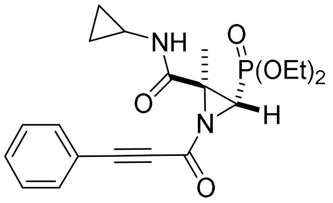

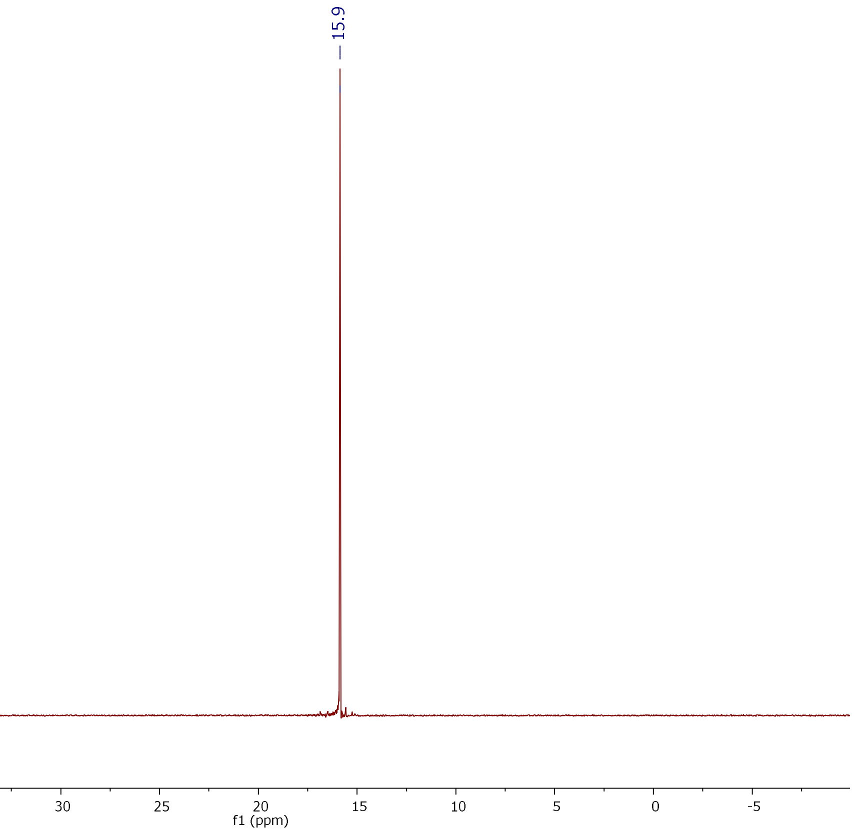

^{1}H NMR (400 MHz, $CDCl_3$) of *N*-Acylaziridine Phosphonate **5h**

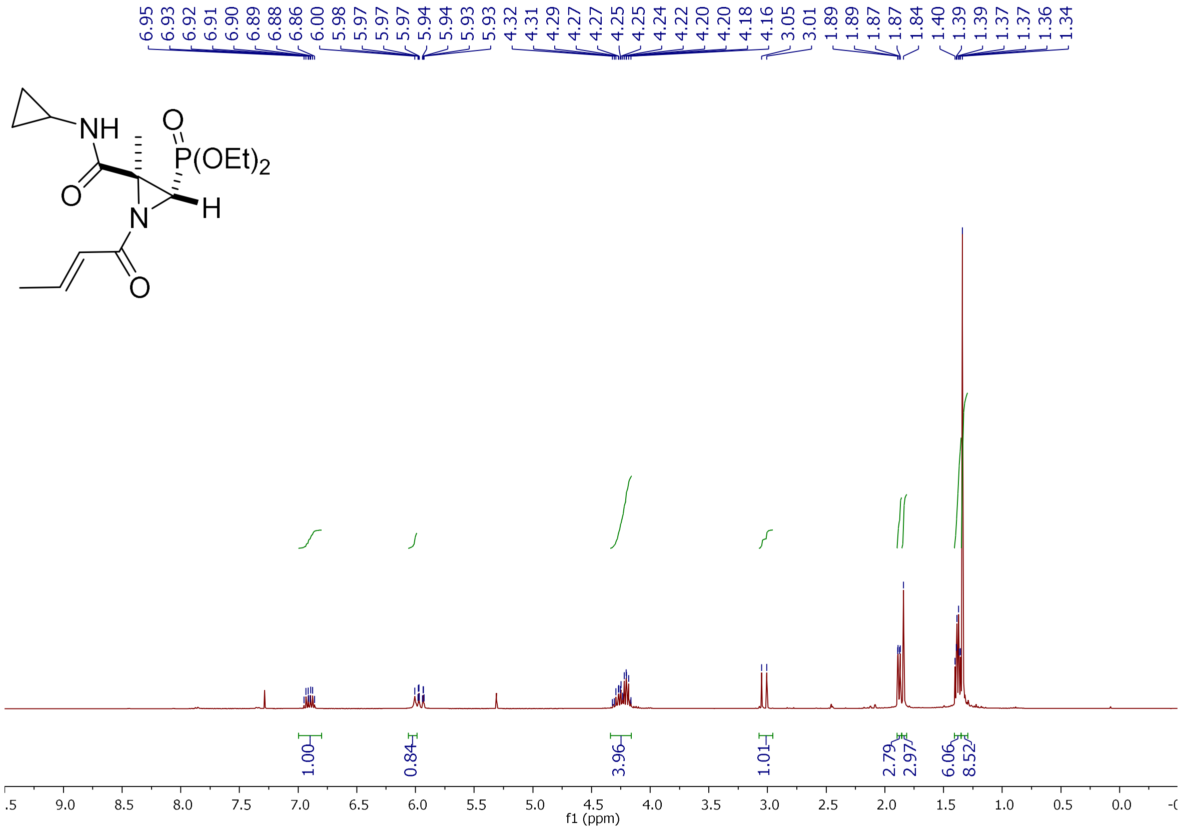

^{13}C {^{1}H} NMR (100 MHz, $CDCl_3$) of *N*-Acylaziridine Phosphonate **5h**

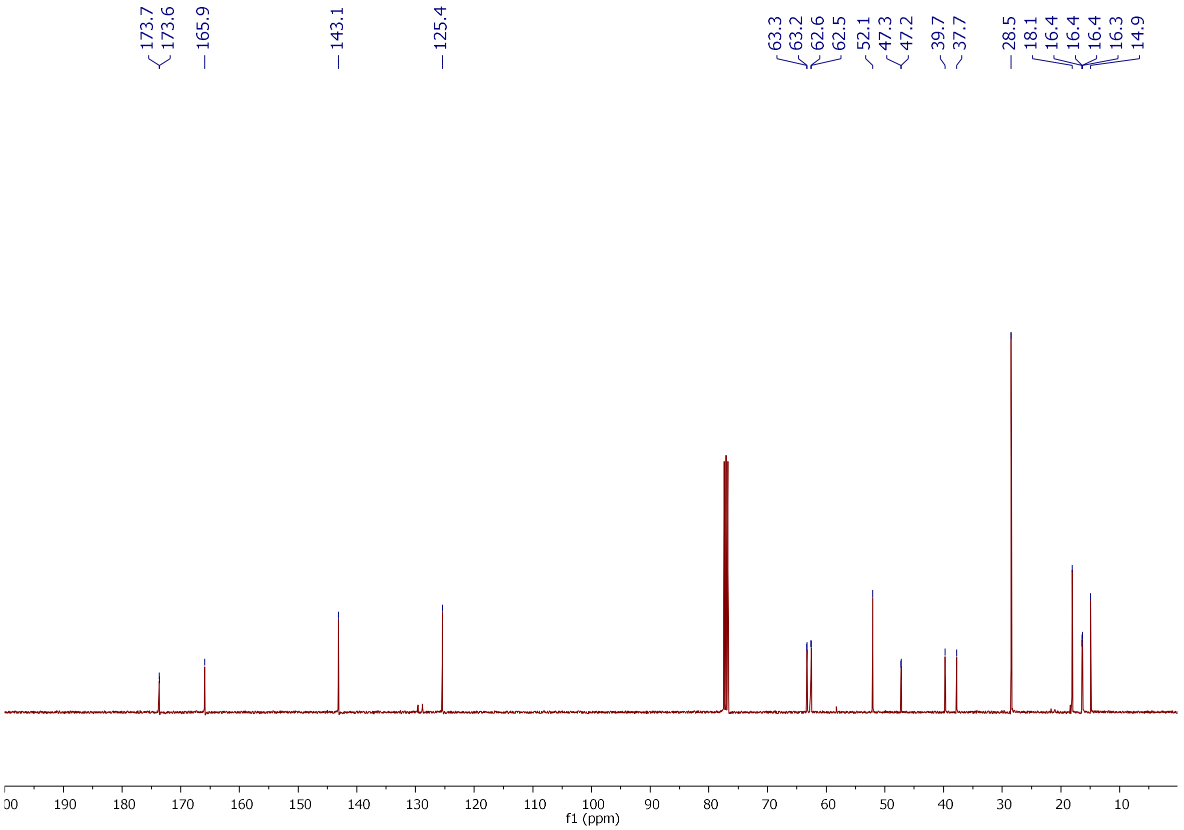

^{31}P (160 MHz, $CDCl_3$) of *N*-Acylaziridine Phosphonate **5h**

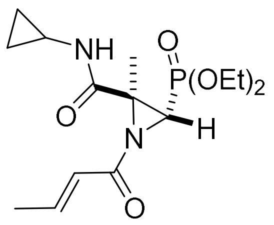

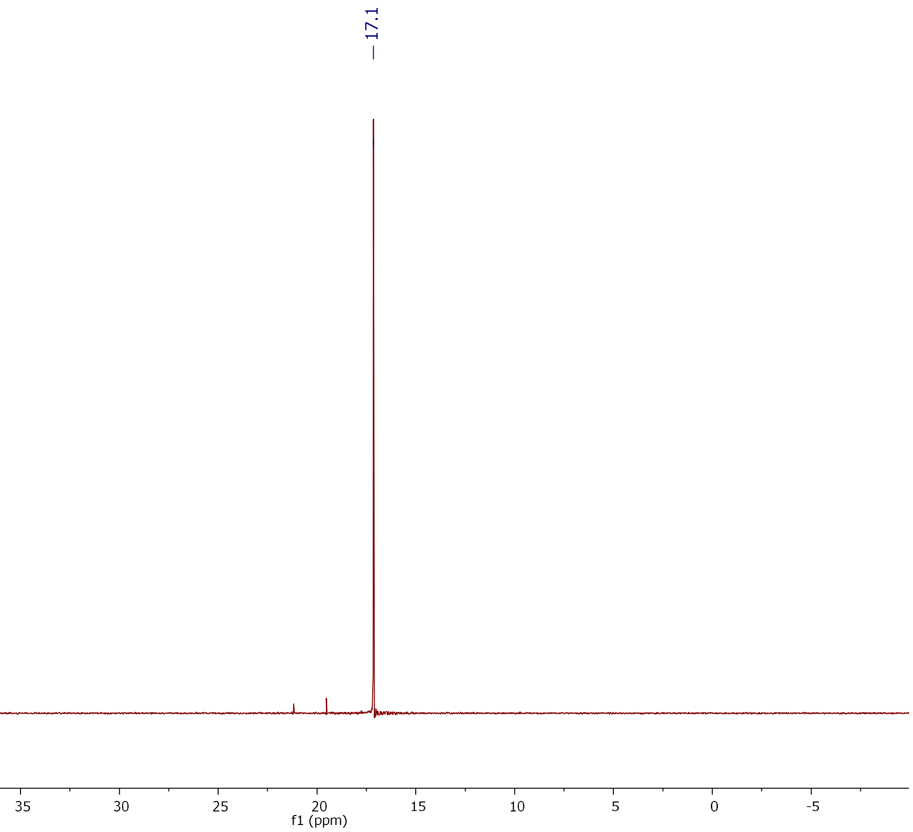

^{1}H NMR (400 MHz, $CDCl_3$) of *N*-Acylaziridine Peptidomimetic **6a**

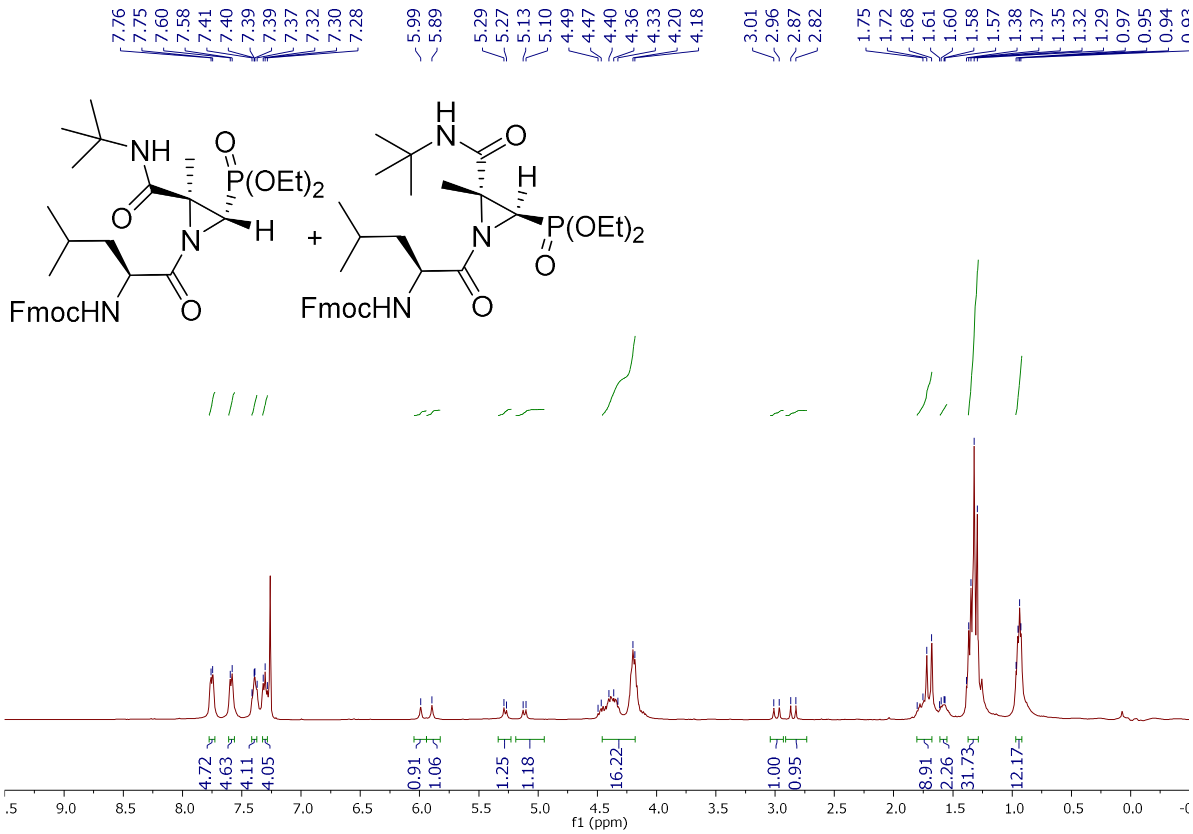

^{13}C {^{1}H} NMR (100 MHz, $CDCl_3$) of *N*-Acylaziridine Peptidomimetic **6a**

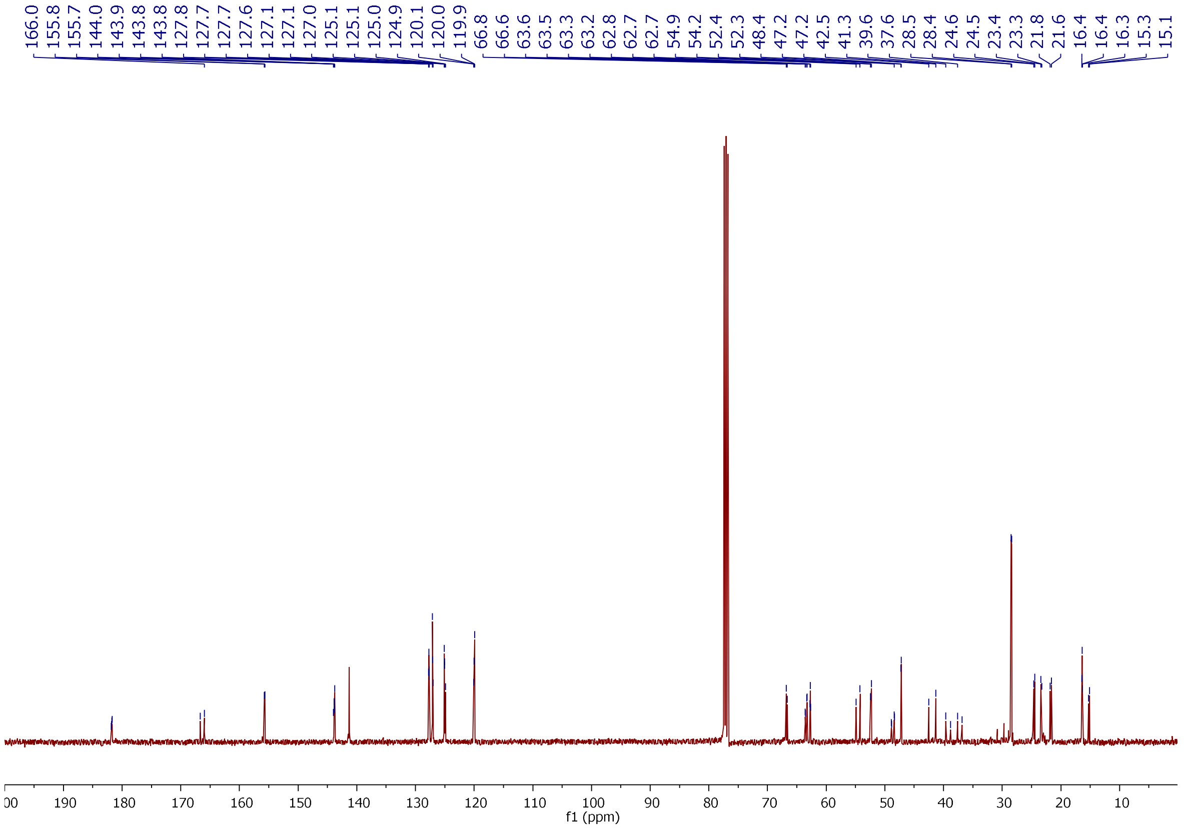

^{31}P (160 MHz, $CDCl_3$) of *N*-Acylaziridine Peptidomimetic **6a**

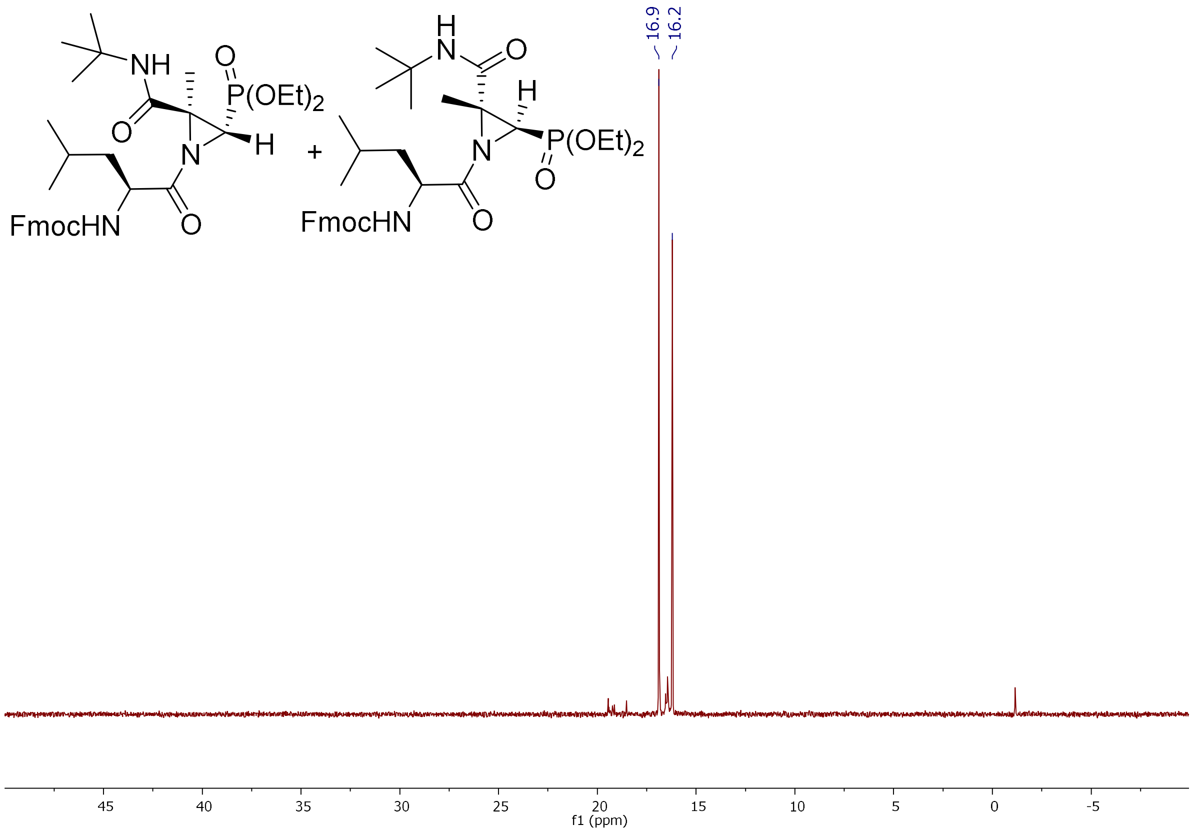

^{1}H NMR (400 MHz, $CDCl_3$) of *N*-Acylaziridine Peptidomimetic **6b**

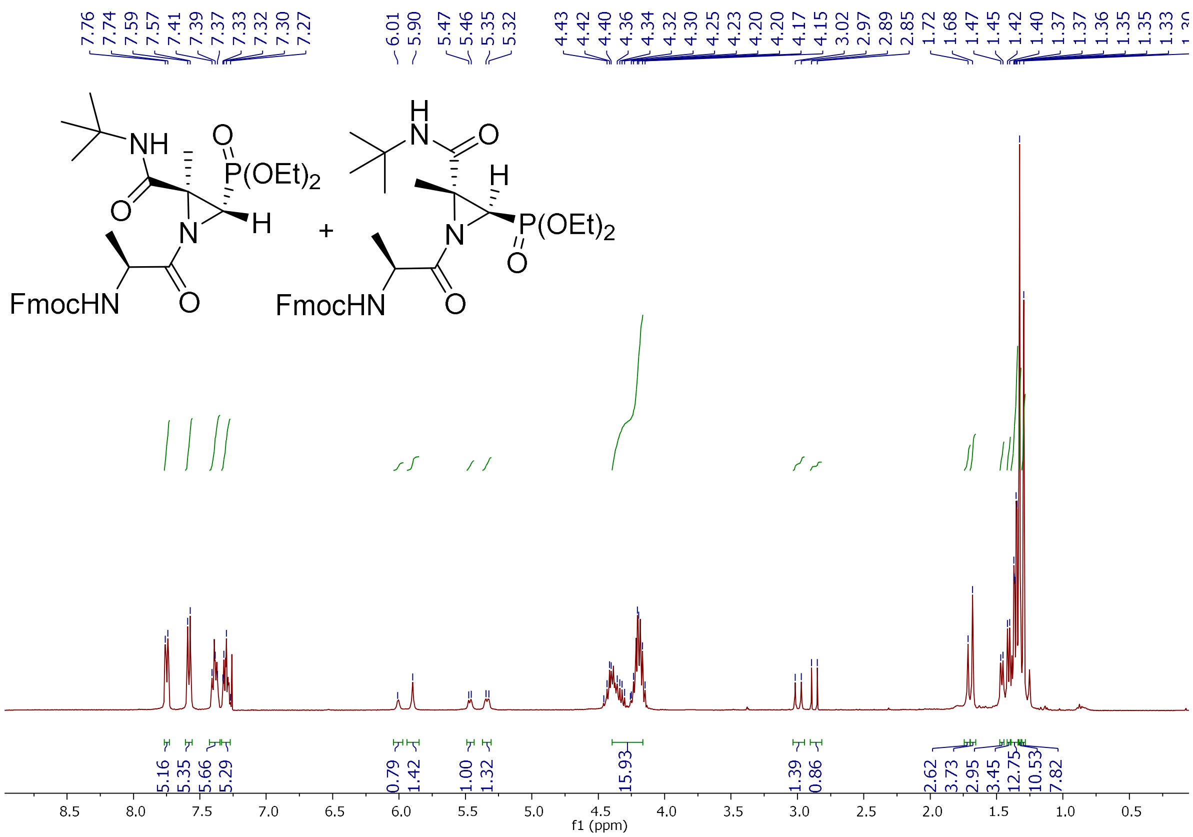

^{13}C {^{1}H} NMR (100 MHz, $CDCl_3$) of *N*-Acylaziridine Peptidomimetic **6b**

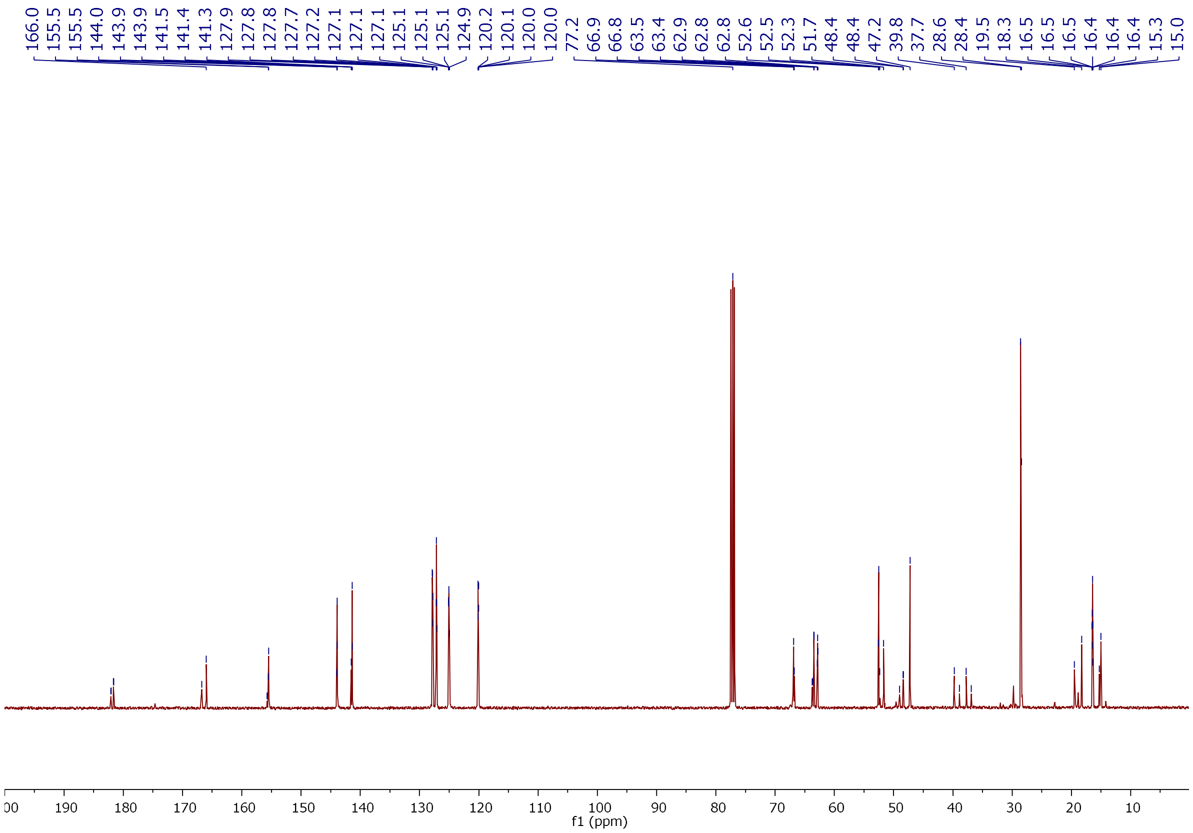

^{31}P (160 MHz, $CDCl_3$) of *N*-Acylaziridine Peptidomimetic **6b**

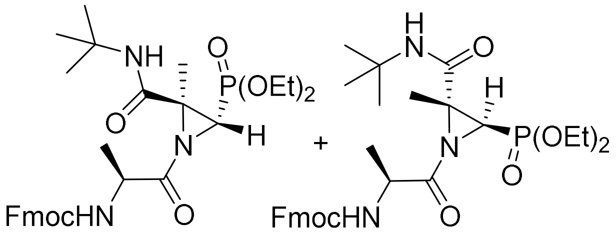

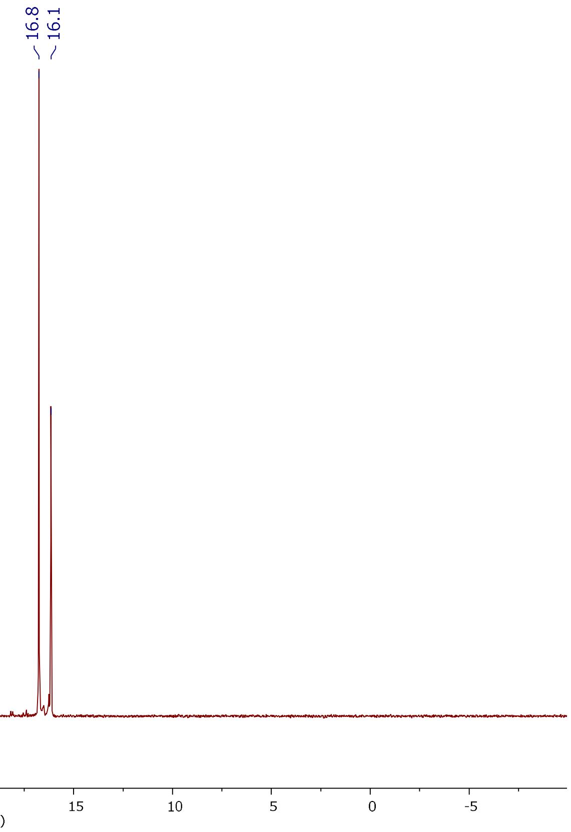

^{1}H NMR (400 MHz, $CDCl_3$) of Oxazole Derivative **7a**

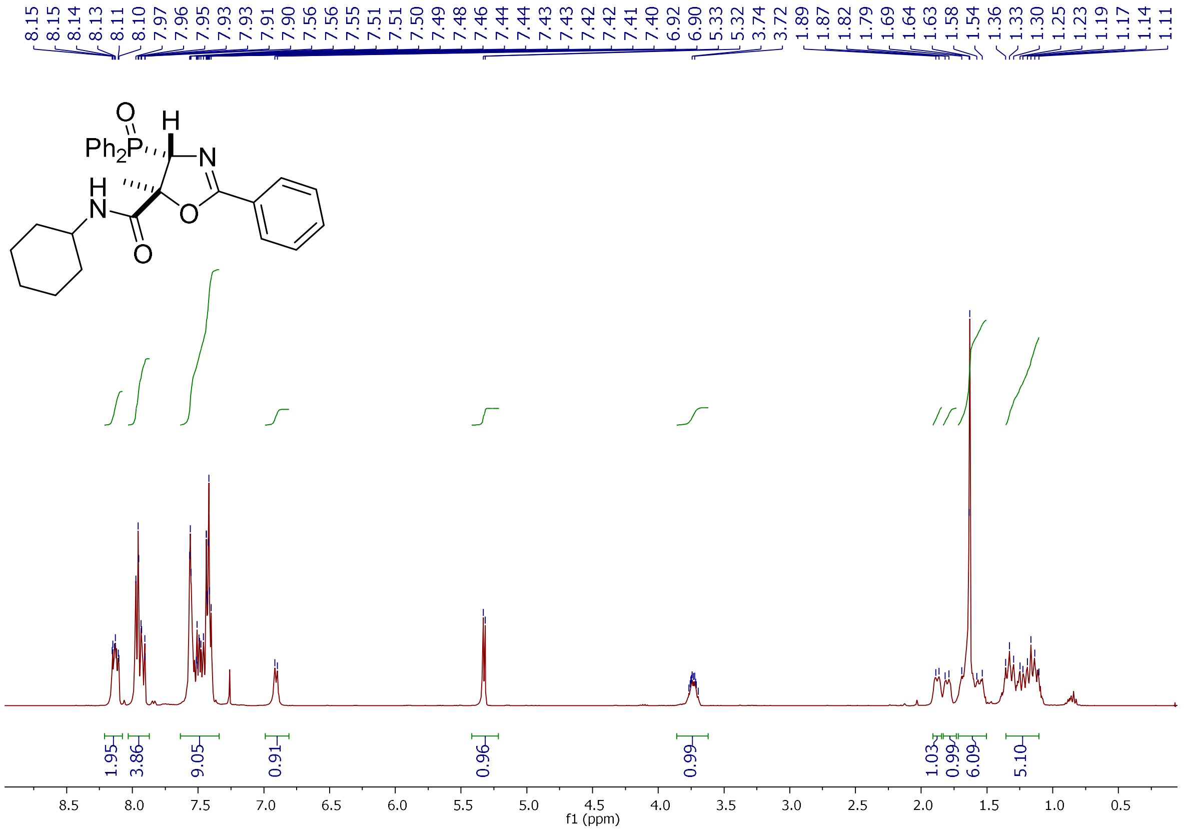

^{13}C {^{1}H} NMR (100 MHz, $CDCl_3$) of Oxazole Derivative **7a**

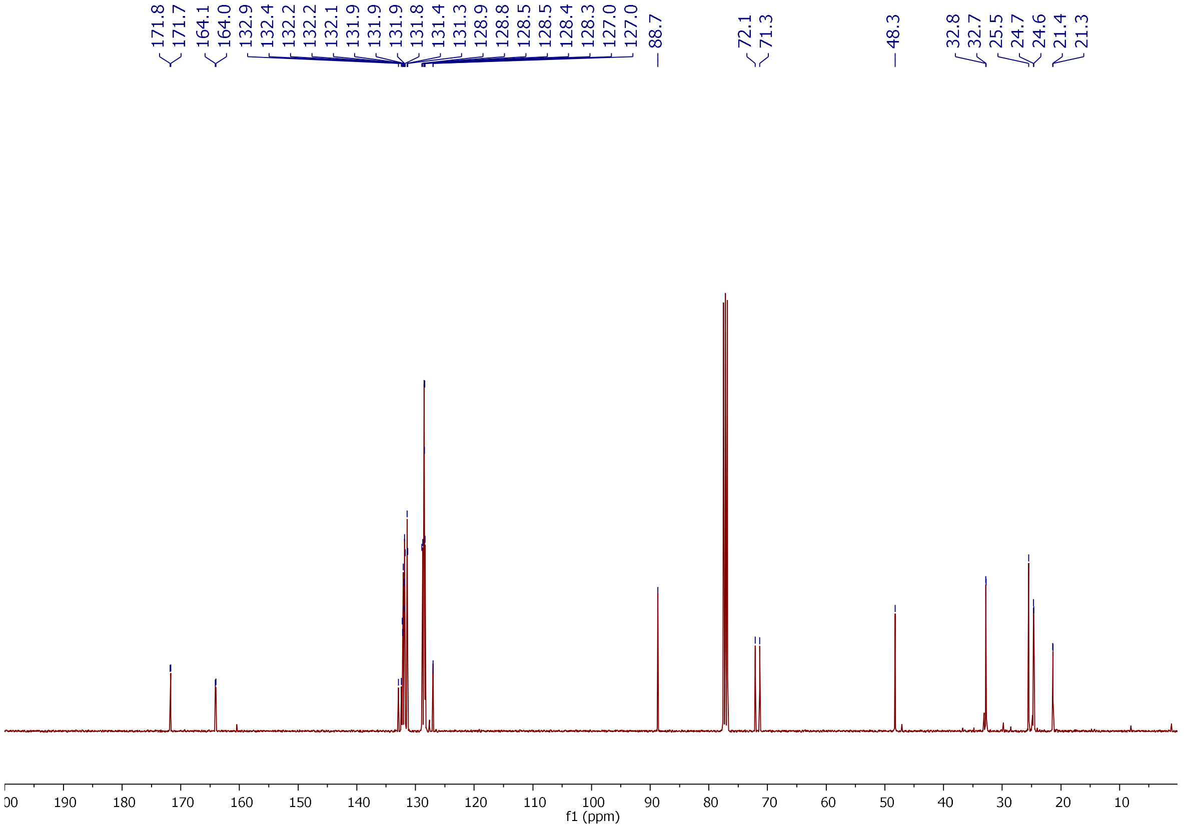

^{31}P (160 MHz, $CDCl_3$) of Oxazole Derivative **7a**

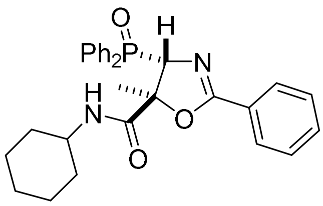

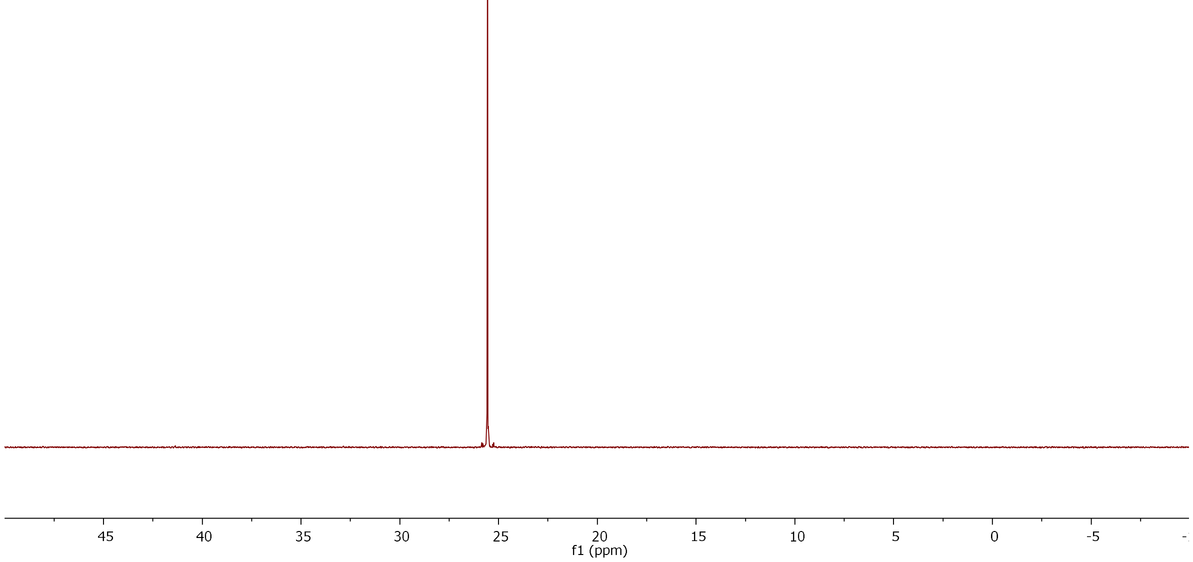

^{1}H NMR (400 MHz, $CDCl_3$) of Oxazole Derivative **7b**

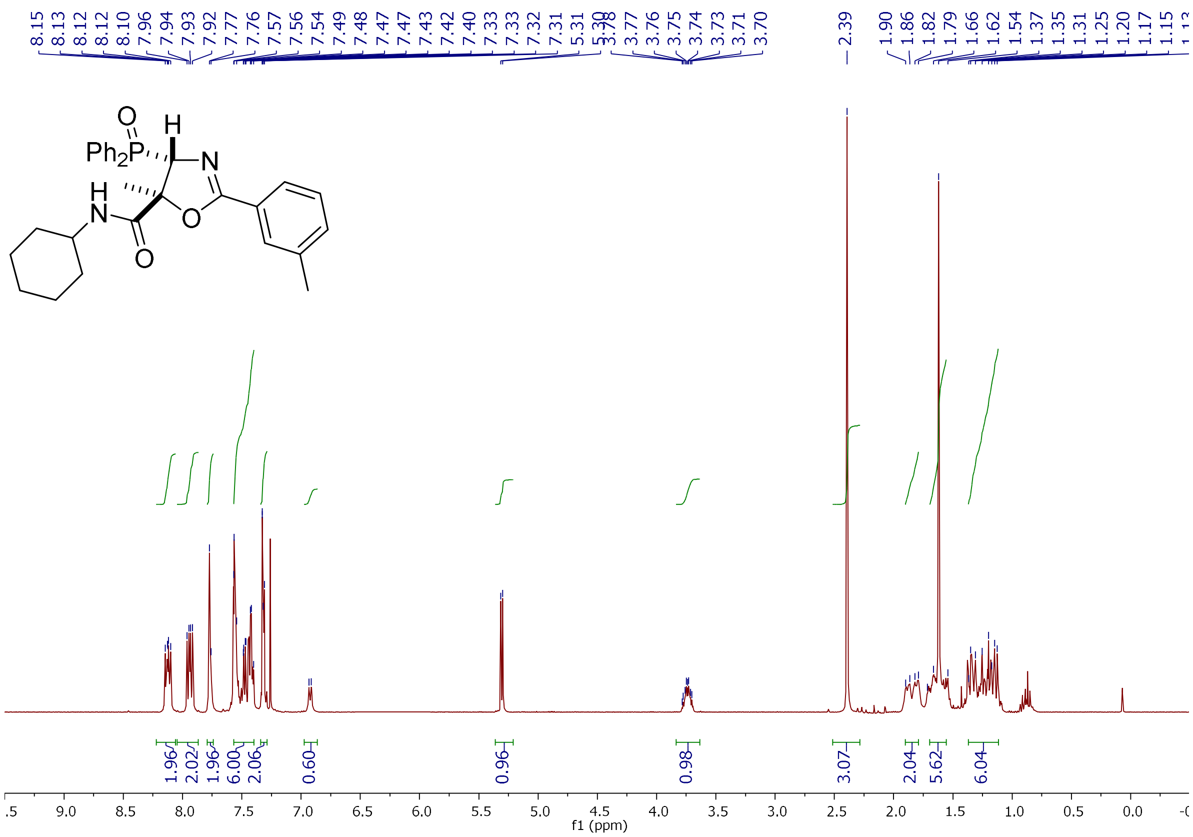

^{13}C {^{1}H} NMR (100 MHz, $CDCl_3$) of Oxazole Derivative **7b**

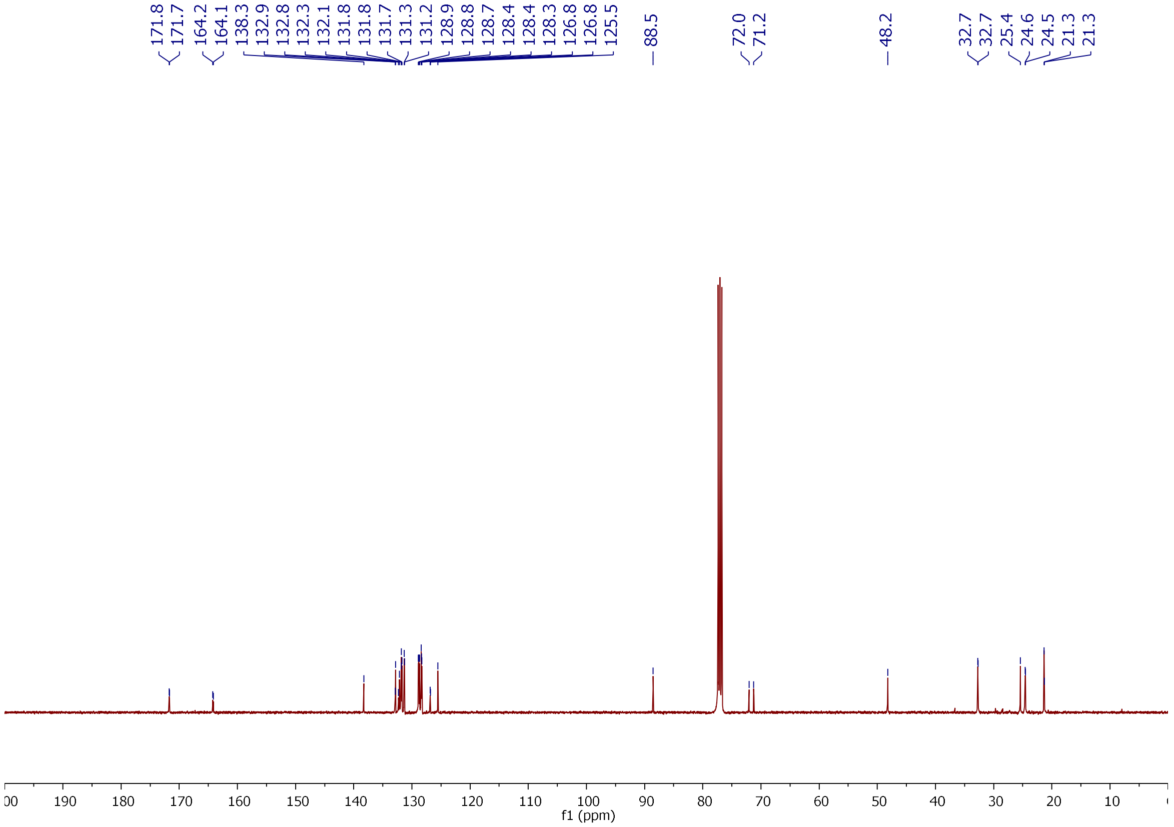

^{31}P (160 MHz, $CDCl_3$) of Oxazole Derivative **7b**

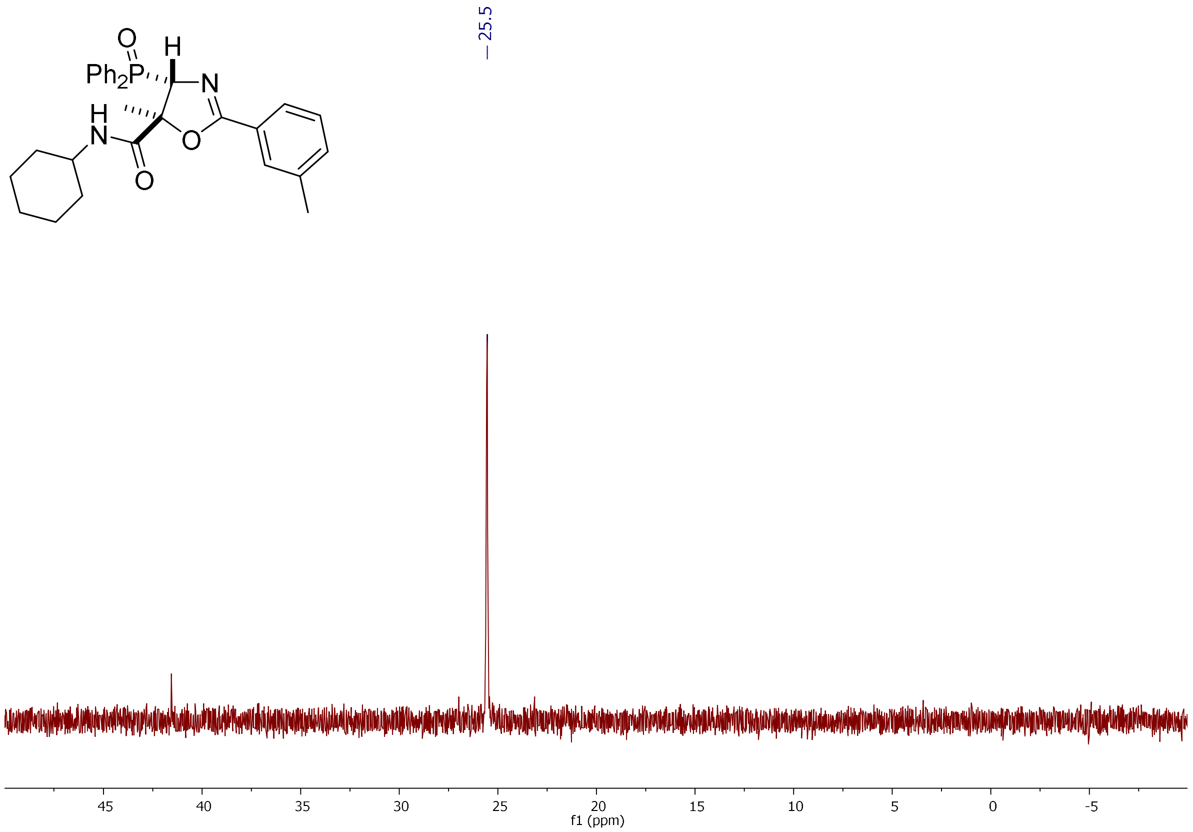

^{1}H NMR (400 MHz, $CDCl_3$) of Oxazole Derivative **7c**

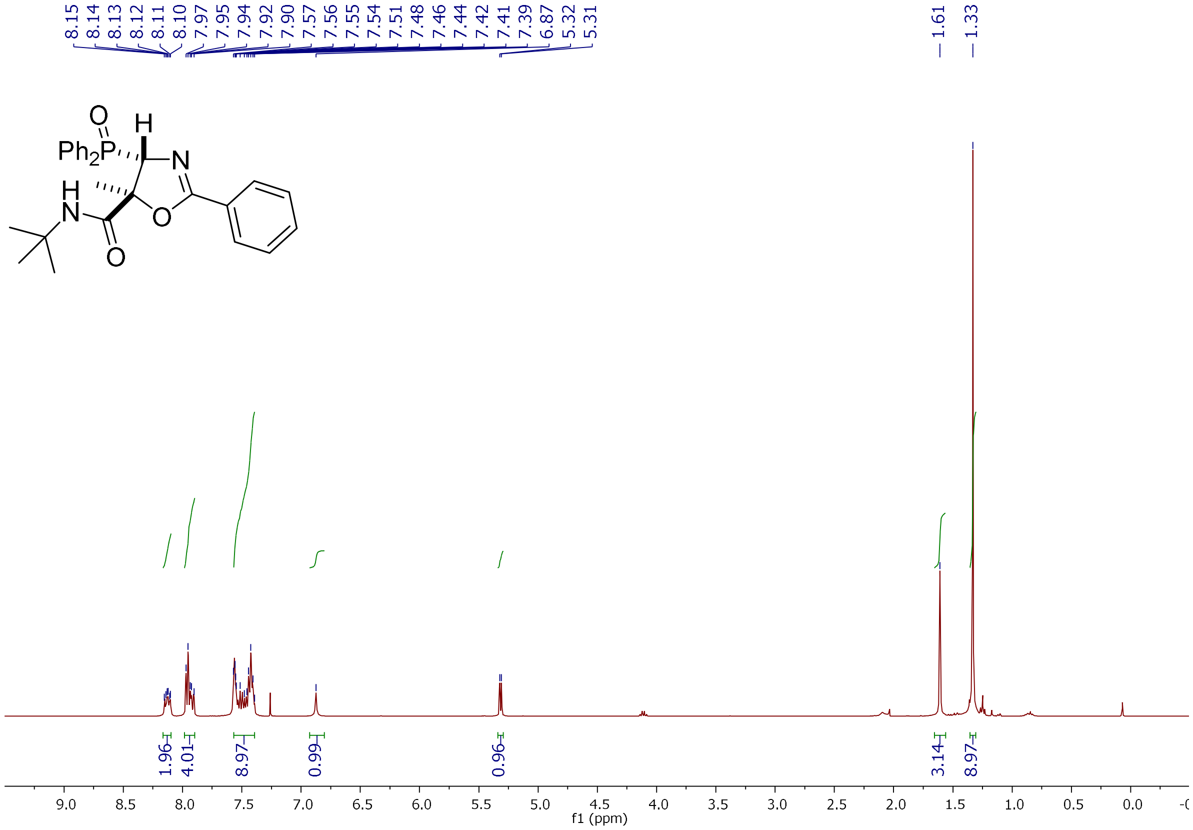

$^{13}C\{^{1}H\}$ NMR (100 MHz, $CDCl_3$) of Oxazole Derivative **7c**

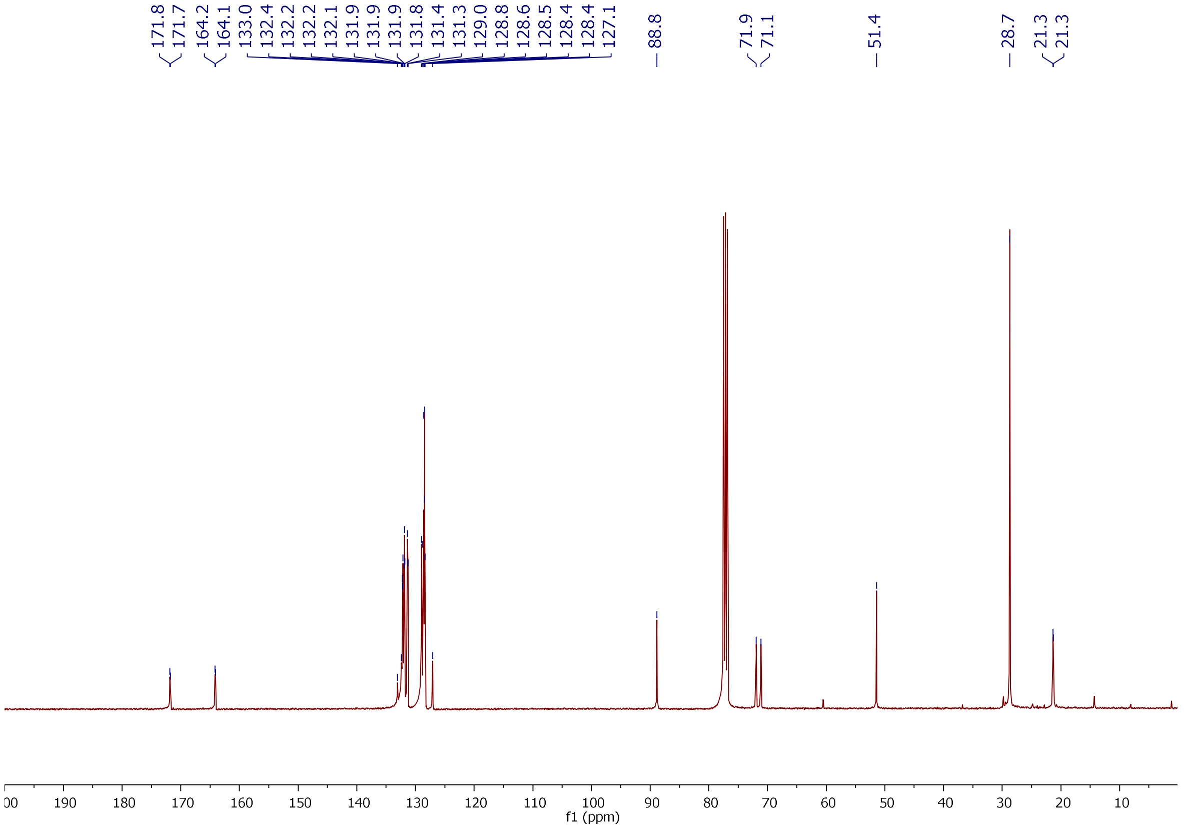

^{31}P (160 MHz, $CDCl_3$) of Oxazole Derivative **7c**

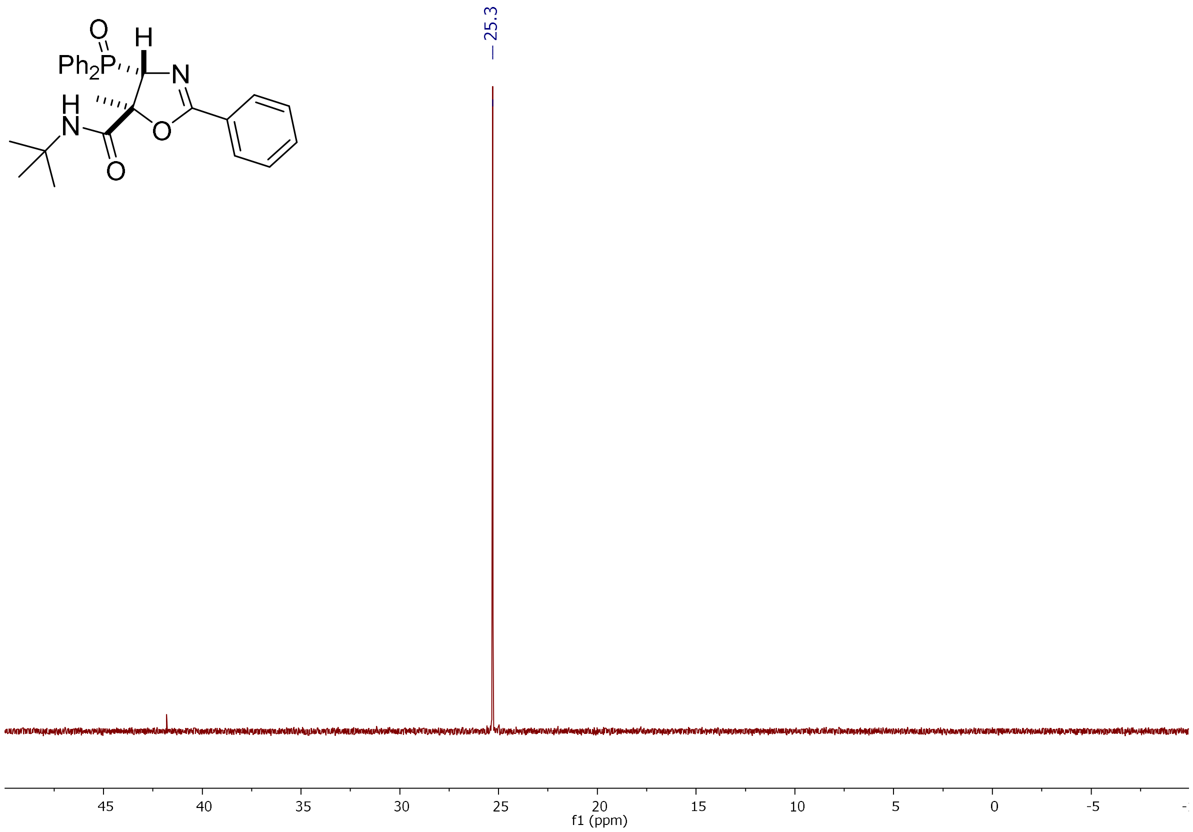

^{1}H NMR (400 MHz, $CDCl_3$) of Oxazole Derivative **7d**

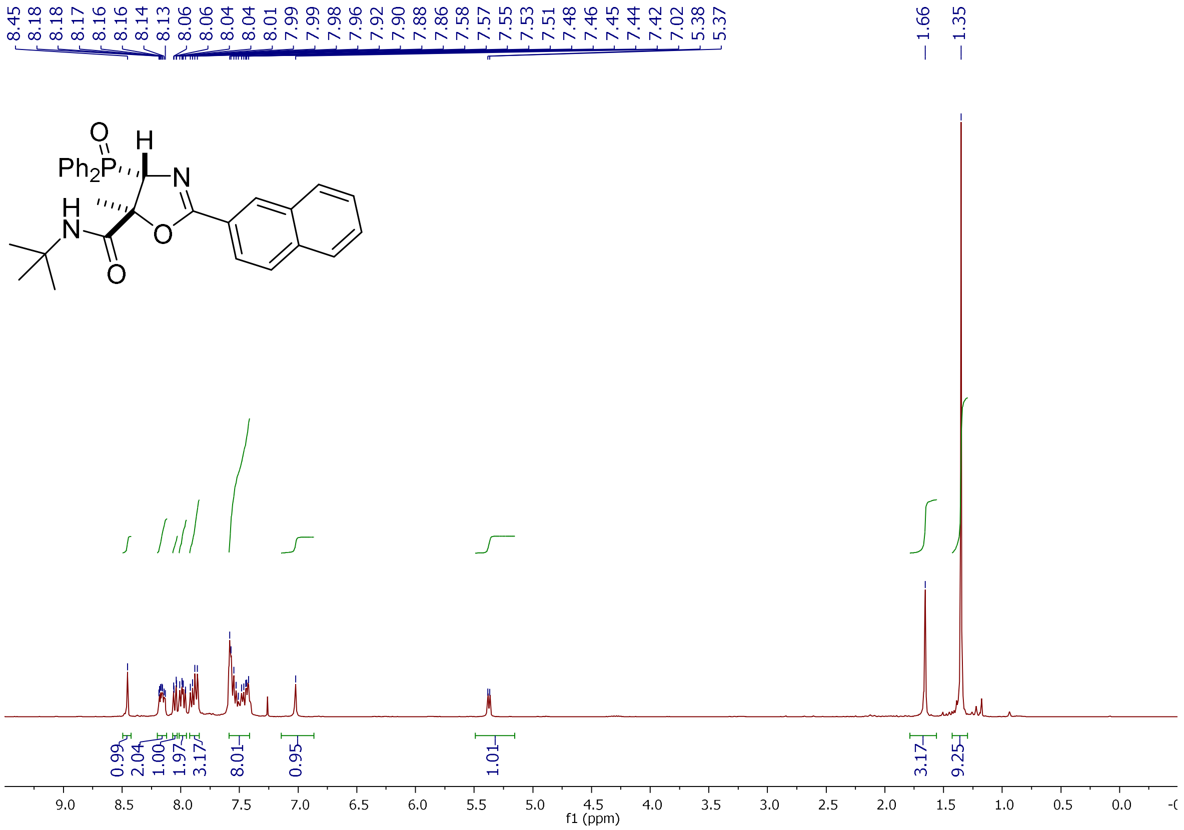

^{13}C {^{1}H} NMR (100 MHz, $CDCl_3$) of Oxazole Derivative **7d**

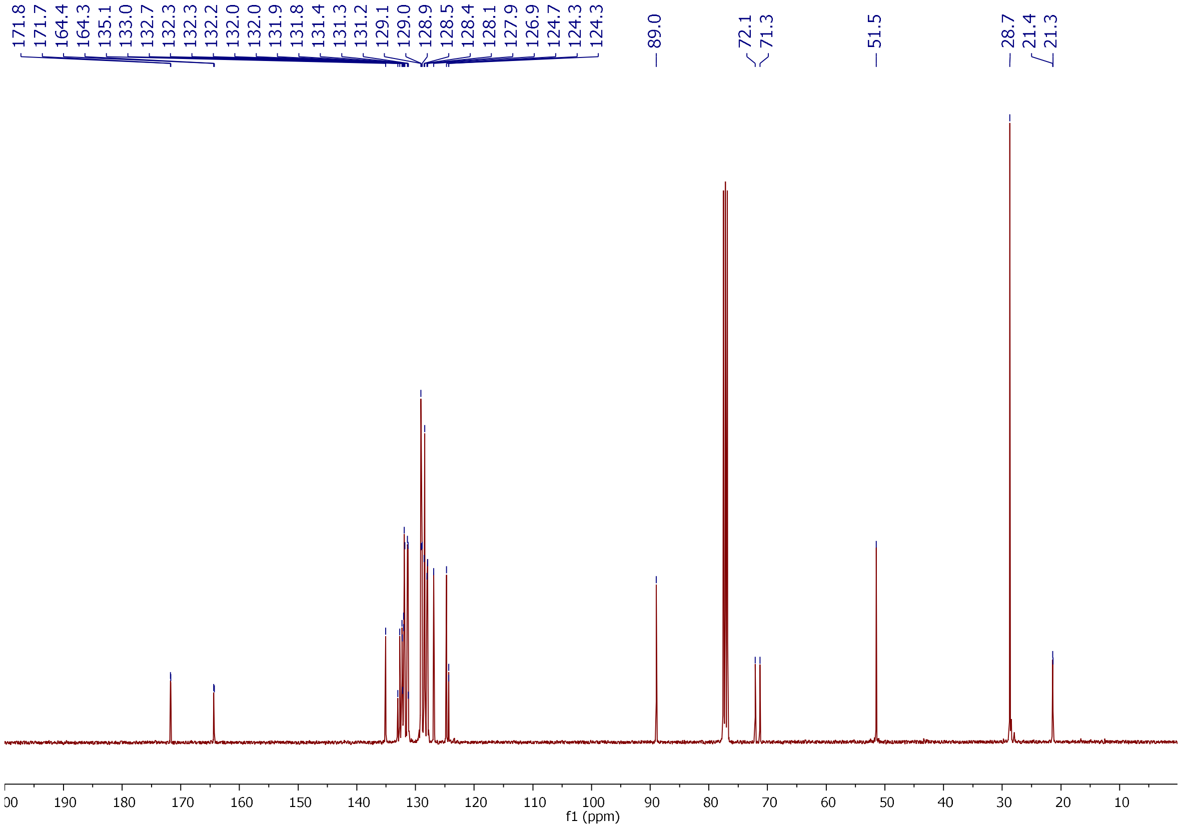

^{31}P (160 MHz, $CDCl_3$) of Oxazole Derivative **7d**

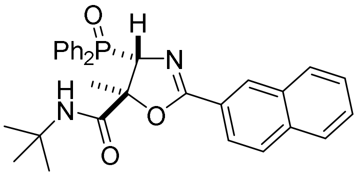

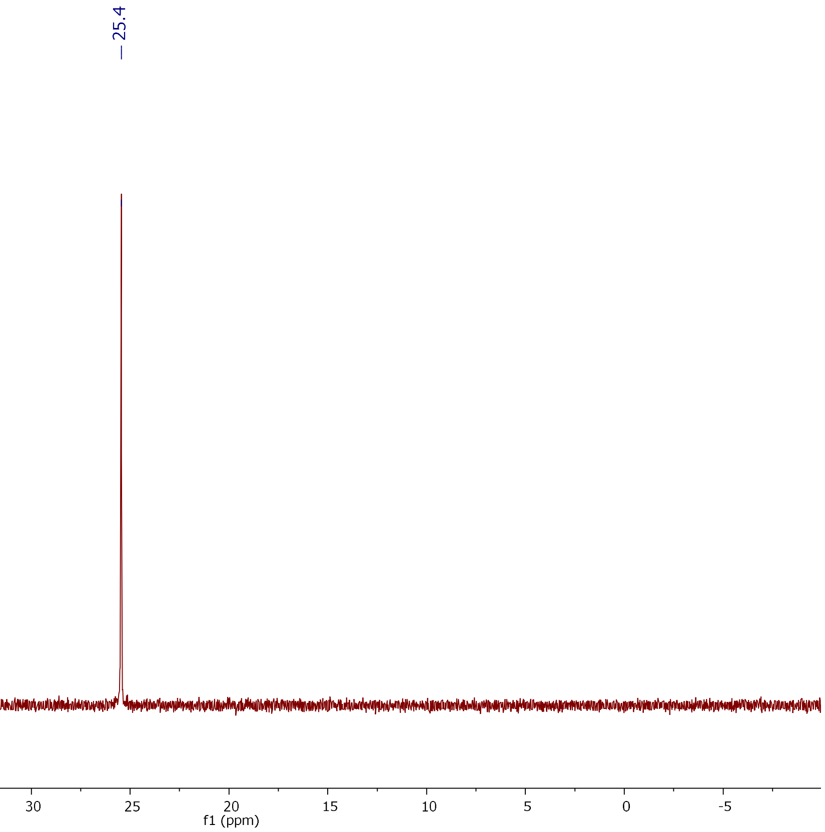

^{1}H NMR (400 MHz, $CDCl_3$) of Oxazole Derivative **7e**

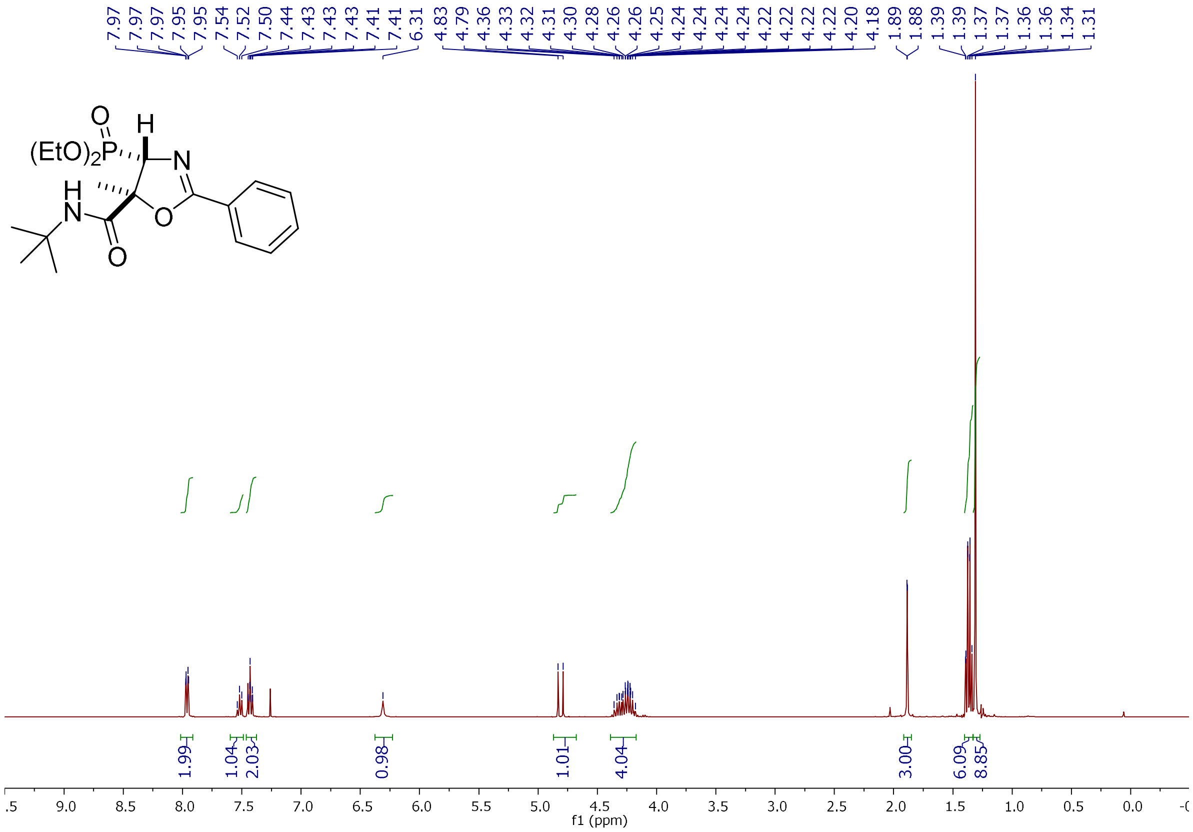

^{13}C {^{1}H} NMR (100 MHz, $CDCl_3$) of Oxazole Derivative **7e**

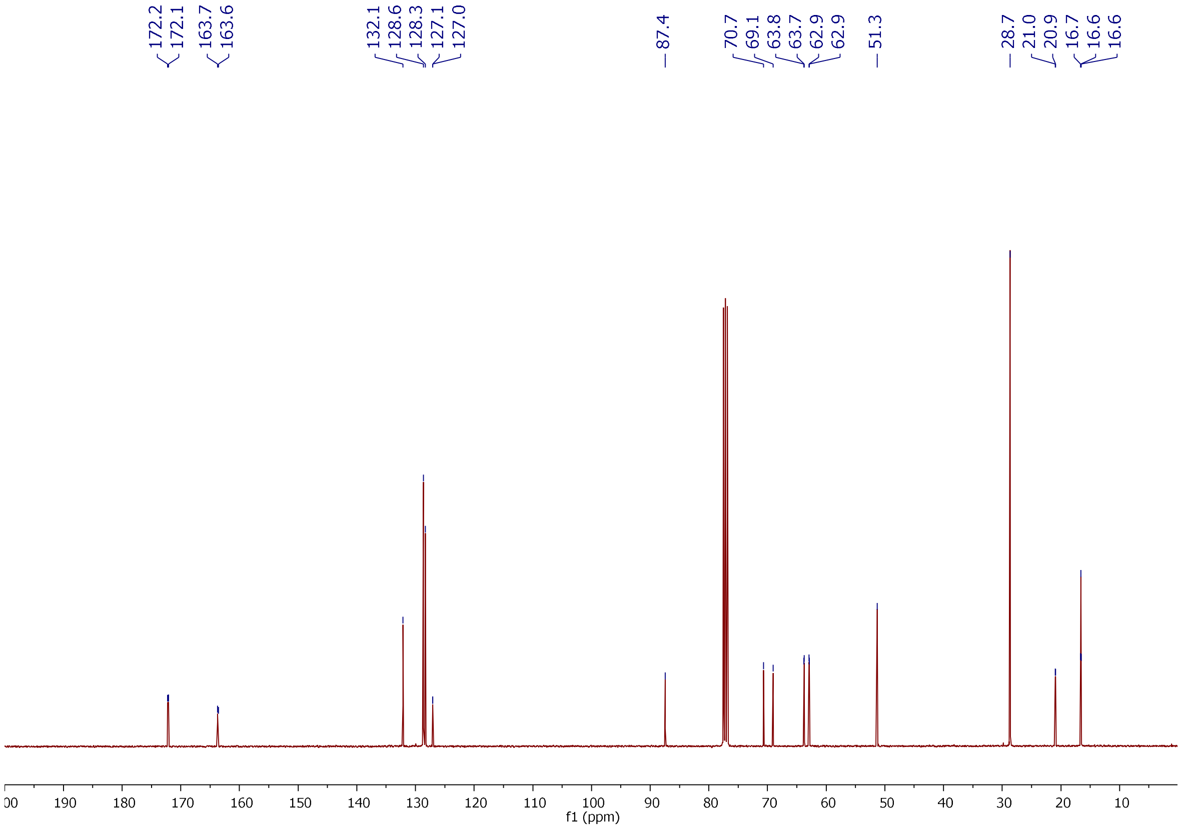

^{31}P (160 MHz, $CDCl_3$) of Oxazole Derivative **7e**

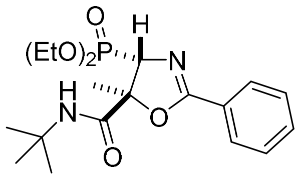

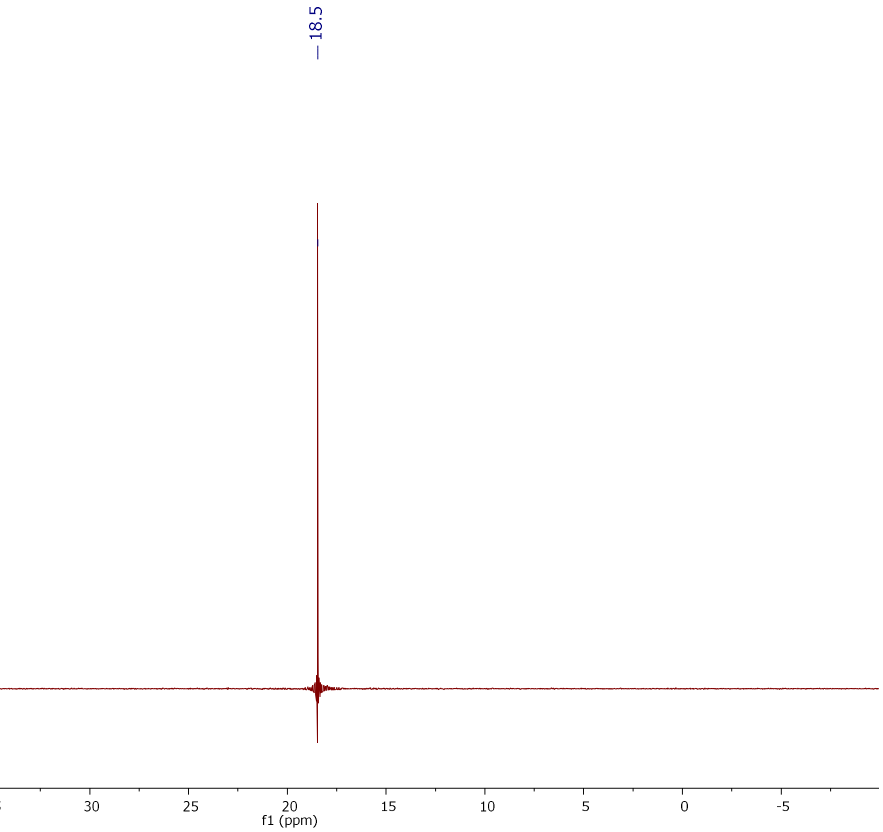

^{1}H NMR (400 MHz, $CDCl_3$) of Oxazole Derivative **7f**

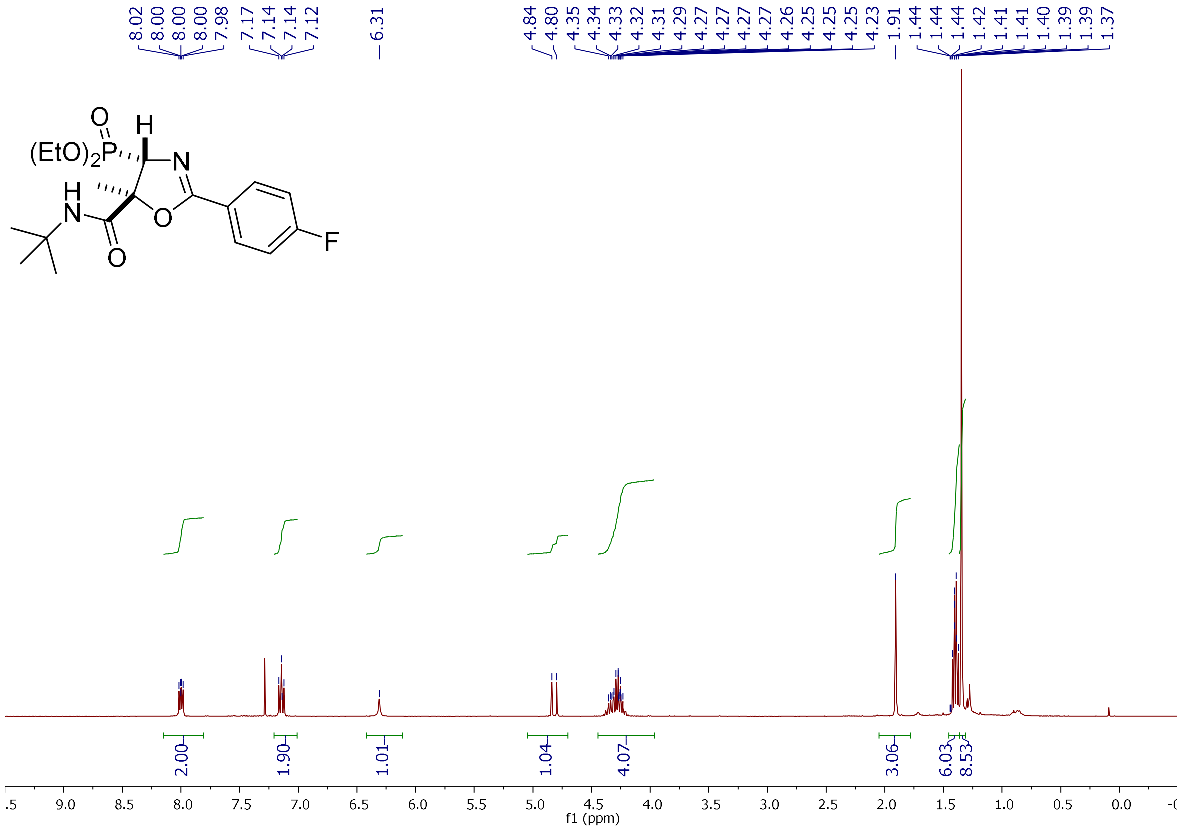

^{13}C {^{1}H} NMR (100 MHz, $CDCl_3$) of Oxazole Derivative **7f**

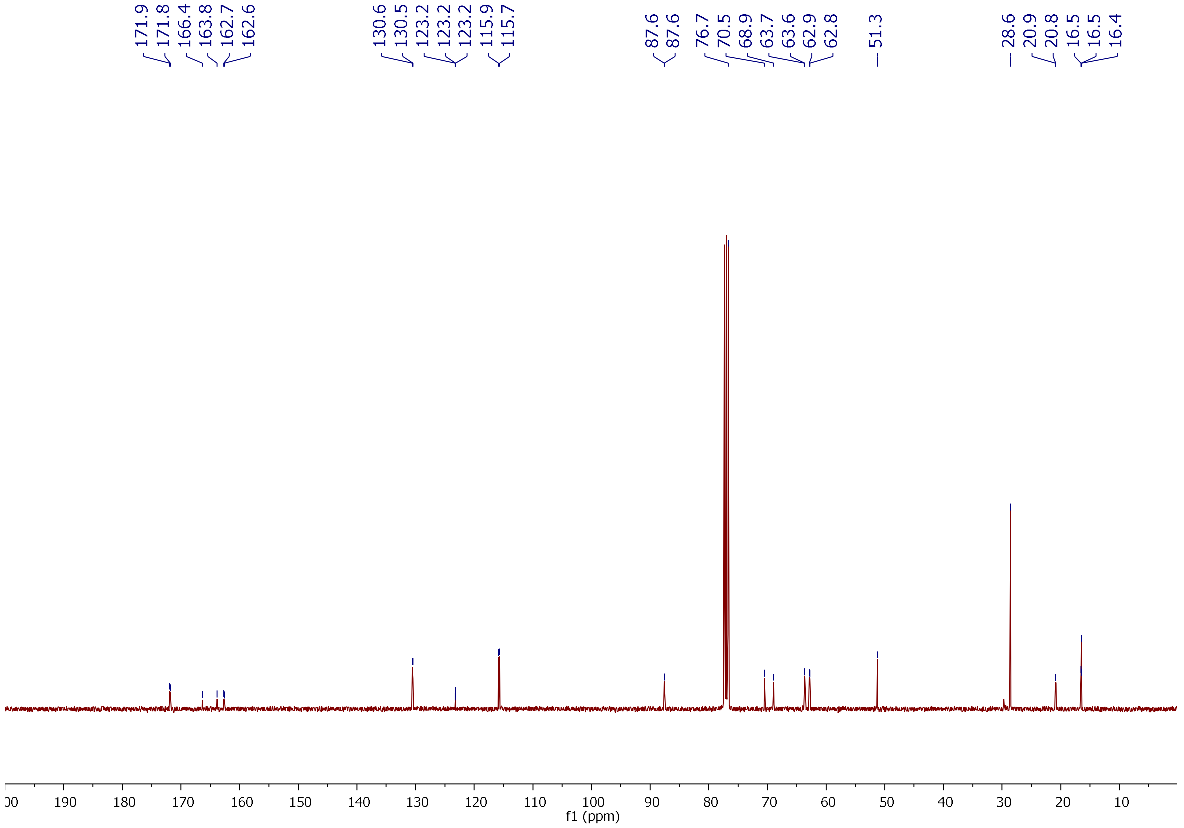

^{31}P (160 MHz, $CDCl_3$) of Oxazole Derivative **7f**

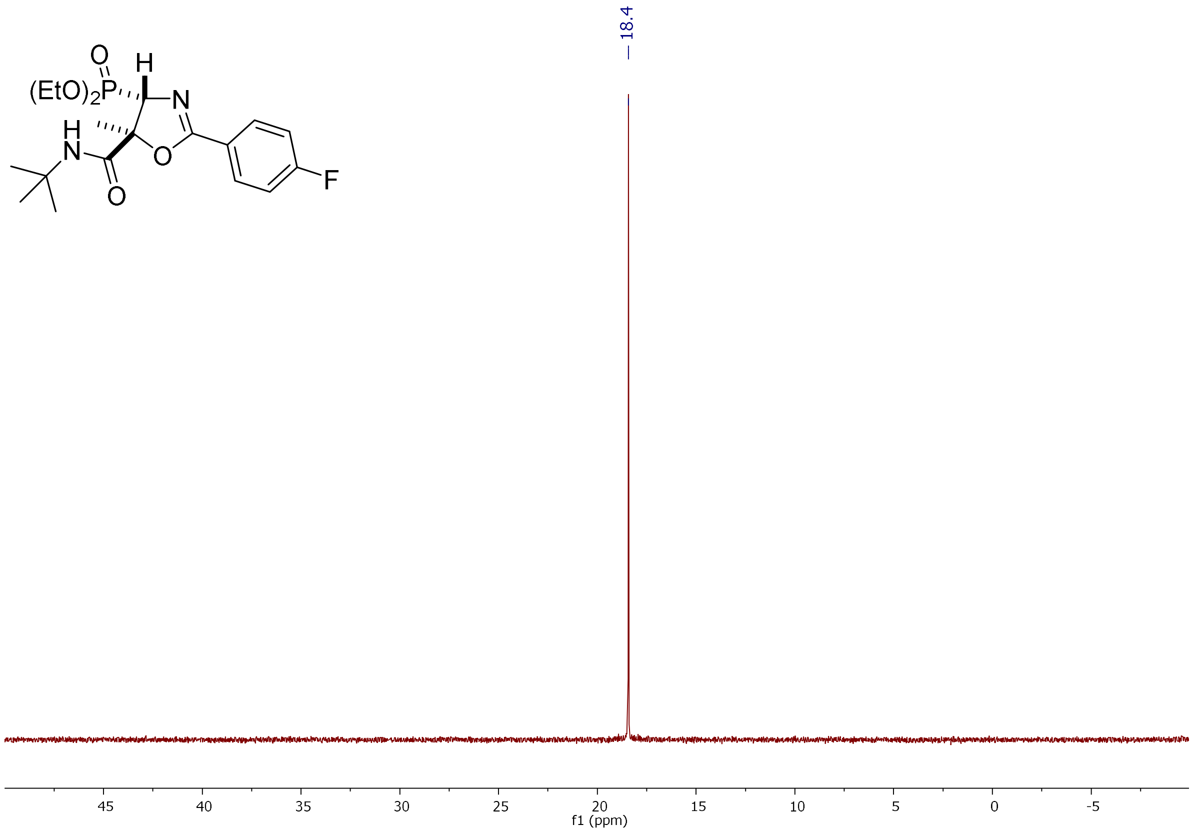

^{19}F (376 MHz, $CDCl_3$) of Oxazole Derivative **7f**

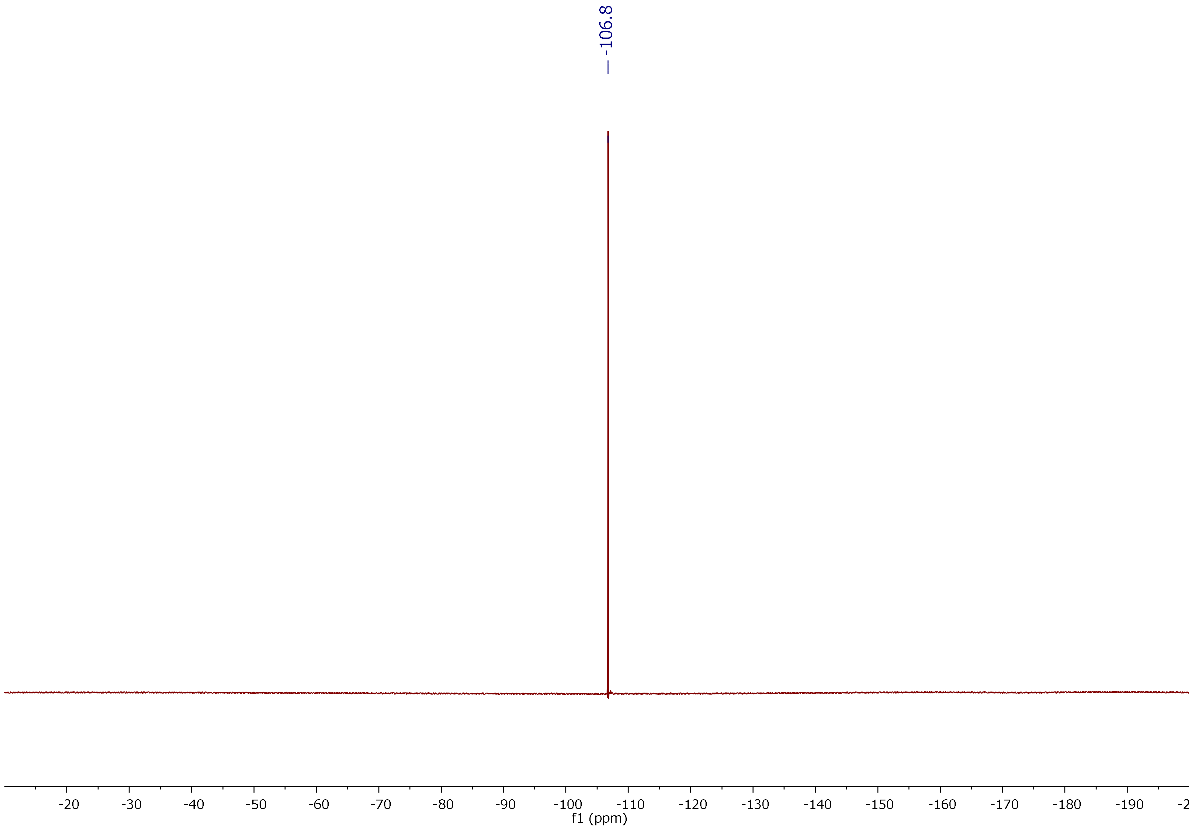

2. ORTEP view and X-ray crystallographic statistics for compounds 4i and 7a

N-Acylaziridine Phosphine Oxide **4i**

(CCDC deposition number 2304039)

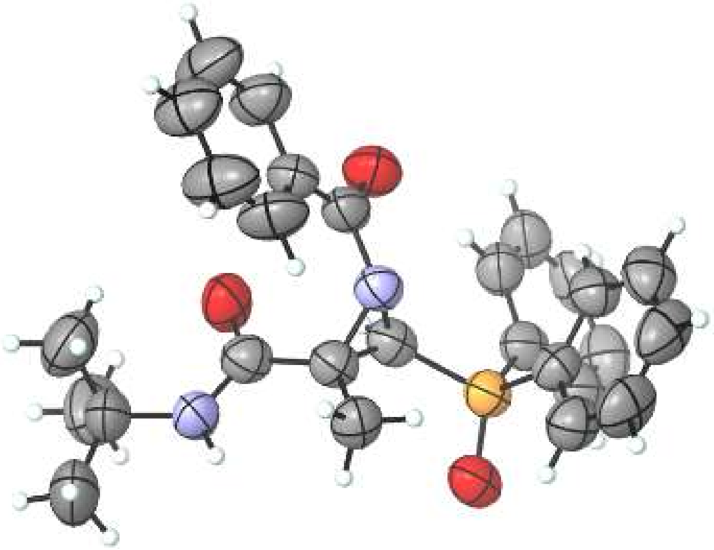

Figure S1. ORTEP diagram of compound **4i** with thermal displacement parameters drawn at 50% probability.

Single crystals of $C_{27}H_{29}N_2O_3P$ **[4i]** were obtained. A suitable crystal was selected and mounted on a SuperNova, Dual, Cu at home/near, HyPix diffractometer. The crystal was kept at 170.01(10) K during data collection. Using Olex2,[1] the structure was solved with the ShelXS[2] structure solution program using Intrinsic Phasing and refined with the ShelXL[3] refinement package using Least Squares minimization.

Table S1. Crystal data and structure refinement for **4i**.

Empirical formula	C27H29N2O3P
Formula weight	460.49
Temperature/K	170.01(10)
Crystal system	monoclinic
Space group	Pc
a/Å	14.3797(5)
b/Å	18.5342(9)
c/Å	9.2763(3)
α/°	90.0
β/°	99.780(3)
γ/°	90.0
Volume/Å^3	2436.35(17)
Z	4
ρcalcg/cm^3	1.255
μ/mm^{-1}	1.247
F(000)	976.0
Crystal size/mm^3	0.255 × 0.133 × 0.118
Radiation	CuKα (λ = 1.54184)
2Θ range for data collection/°	6.238 to 138
Index ranges	-17 ≤ h ≤ 17, -22 ≤ k ≤ 20, -11 ≤ l ≤ 11
Reflections collected	43034
Independent reflections	8983 [R_{int} = 0.1481, R_{sigma} = 0.0972]
Data/restraints/parameters	8983/14/603
Goodness-of-fit on F^2	1.032
Final R indexes [I>=2σ (I)]	R_1 = 0.0819, wR_2 = 0.2127
Final R indexes [all data]	R_1 = 0.1062, wR_2 = 0.2367
Largest diff. peak/hole / e Å^{-3}	0.51/-0.43
Flack parameter	0.04(4)
Bijvoet Pairs Covarage	98%
Hooft y	0.02(3)
P3 false	≤10^{-99}

Oxazole Derivative **7a**

(CCDC deposition number 2304041)

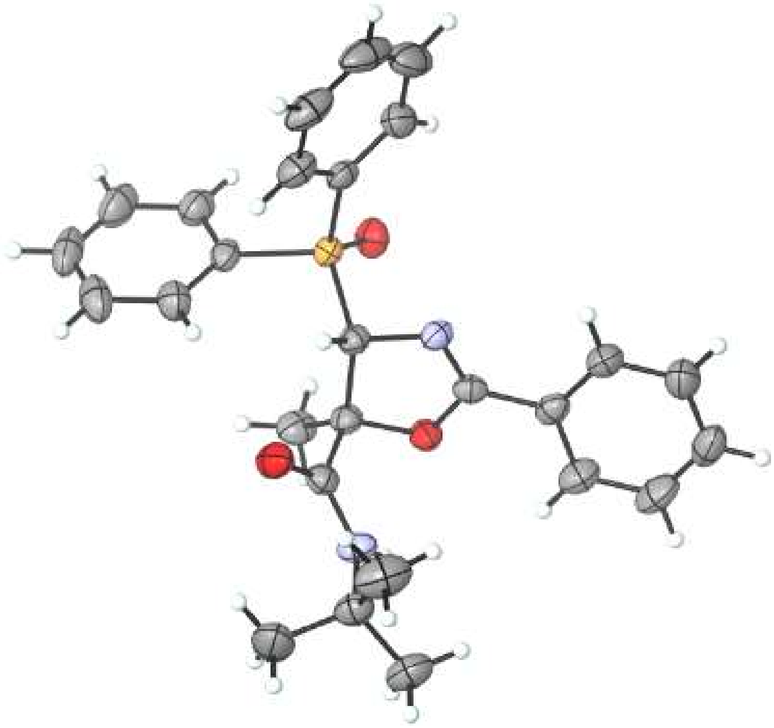

Figure S2. ORTEP diagram of compound **7a** with thermal displacement parameters drawn at 50% probability.

Single crystals of $C_{27}H_{29}N_2O_3P$ **[7a]** were obtained. A suitable crystal was selected and mounted on a SuperNova, Dual, Cu at home/near, HyPix diffractometer. The crystal was kept at 170.01(10) K during data collection. Using Olex2,[1] the structure was solved with the ShelXS[2] structure solution program using Intrinsic Phasing and refined with the ShelXL[3] refinement package using Least Squares minimization.

Table S2. Crystal data and structure refinement for **7a**.

Empirical formula	$C_{27}H_{29}N_2O_3P$
Formula weight	460.49
Temperature/K	170.00(10)
Crystal system	monoclinic
Space group	P21/n
a/Å	12.87200(8)
b/Å	10.12924(7)
c/Å	18.65780(11)
α/°	90.0
β/°	90.2466(5)
γ/°	90.0
Volume/Å^3	2432.65(3)
Z	4
ρcalcg/cm^3	1.257
μ/mm^{-1}	1.247
F(000)	976.0
Crystal size/mm^3	0.559 × 0.26 × 0.126
Radiation	CuKα (λ = 1.54184)
2Θ range for data collection/°	8.328 to 137.96
Index ranges	-15 ≤ h ≤ 15, -12 ≤ k ≤ 12, -22 ≤ l ≤ 22
Reflections collected	44390
Independent reflections	4528 [R_{int} = 0.0611, R_{sigma} = 0.0264]
Data/restraints/parameters	4528/0/302
Goodness-of-fit on F^2	1.044
Final R indexes [I>=2σ (I)]	R_1 = 0.0342, wR_2 = 0.0901
Final R indexes [all data]	R_1 = 0.0356, wR_2 = 0.0914
Largest diff. peak/hole / e Å^{-3}	0.27/-0.43

3. References

[1] Dolomanov, O.V.; Bourhis, L.J.; Gildea, R.J.; Howard, J.A.K.; Puschmann, H. *J. Appl. Cryst.* **2009**, *42*, 339–341.

[2] Sheldrick, G. M. *Acta Cryst*. **2008**, *A64*, 112–122.

[3] Sheldrick, G. M. *Acta Cryst*. **2015**, *C71*, 3–8.
